# Photo-induced ruthenium-catalyzed alkene C–H-arylation at room temperature

**DOI:** 10.1039/d5cc04527d

**Published:** 2025-09-30

**Authors:** Tanumoy Mandal, Stéphane Golling, Sven Trienes, Lutz Ackermann

**Affiliations:** a Wöhler Research Institute for Sustainable Chemistry (WISCh), Georg-August-Universität Göttingen, Tammannstraße 2 37077 Göttingen Germany Lutz.Ackermann@chemie.uni-goettingen.de; b DZHK (German Centre for Cardiovascular Research), Potsdamer Straße 58 10875 Berlin Germany

## Abstract

C–H arylation has surfaced as a powerful tool for molecular sciences, with alkene C–H arylation thus far requiring either high reaction temperatures of 120 °C or stoichiometric amounts of RMgX. In sharp contrast, we herein report on room-temperature C–H arlyations of alkenes by means of ruthenium(ii) catalysis with ample scope. This strategy also enabled late-stage diversification of structurally complex molecules and mechanistic studies provided strong evidence for photo-excitation of a ruthenacycle intermediate.

## Abstract 2

A photo-induced Ru-catalyzed C(sp^2^)–H arylation of heterocycle-tethered alkenes to access to tri-substituted alkenes under mild conditions with broad functional group tolerance, including bio-relevant motifs, is reported.

## Graphical abstract

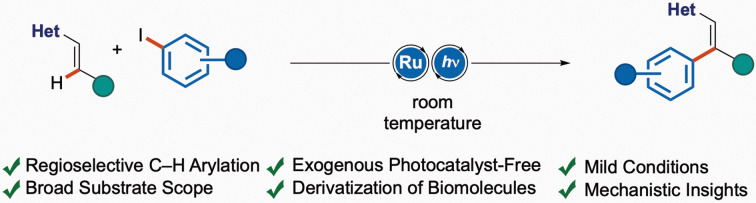

Transition metal catalysis has emerged as a transformative platform for the interconversion of functional groups, enabling the assembly of valuable organic molecules. Particularly, C–H activation represents arguably the most efficient strategy, avoiding lengthy and resource-demanding multi-step syntheses.^1^ The introduction of an aryl motif *via* C–H arylation provided access to distinct molecules by means of ruthenium catalysis,^2^ with various applications to crop protection, polymer chemistry, and drug discovery, among others.^3,4^ While most C(sp^2^)–H functionalizations predominantly focus on arene C–H bonds, the direct activation of vinylic C–H bonds continues to be underdeveloped.^5^ Thus, previous alkene C–H arylations relied as of yet on either high reaction temperatures of 120 °C,^6–10^ or very reactive Grignard reagents in stoichiometric amounts,^11^ thus severely compromising the viable functional group tolerance (Scheme 1A). In sharp contrast, we herein report on the unprecedented C–H-arylation of alkenes at ambient temperature. Key to success was represented by the unique features of light-enabled^12–16^ ruthena(ii)^17^ photoredox catalysis, enabling efficient arylations of alkenes and 1,3-dienes with outstanding levels of chemo-, diastereo- and site-selectivities (Scheme 1B). Late-stage diversifications proved thereby viable at ambient temperature, while detailed mechanistic studies were suggestive of the photo-MLCT-excitation of the key ruthenacycle intermediate.

**Scheme 1:**
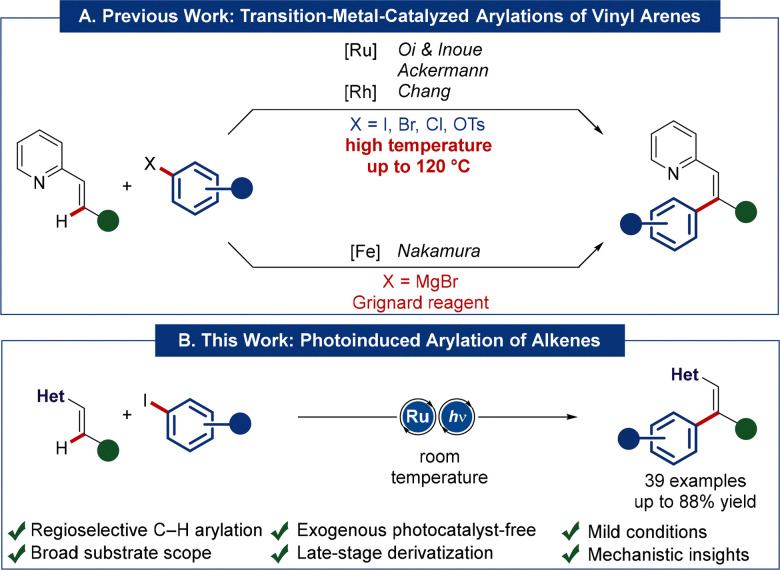
(A) Previous work on directed, transition-metal-catalyzed arylation of alkenes. (B) This work: photo-induced ruthenium-catalyzed C–H-arylation of alkenes.

At the outset of our studies, vinylpyridine 1a and 4-iodoanisole 2a were probed for the photo-induced C(sp^2^)–H-arylation. Gratifyingly, with Na_2_CO_3_ as the base, 10 mol% of [Ru(OAc)_2_(*p*-cymene)] as the catalyst in a mixture of DMA/1,4-dioxane (3 : 1), the corresponding tri-substituted olefin 3a was obtained in 81% yield (Table 1, entry 1). Lower catalytic performances were observed in 1,4-dioxane, DMA, or other solvent mixtures such as DMA/THF in a 3 : 1 ratio (entries 2 and 3). An inferior catalytic efficacy was observed with K_2_CO_3_ or K_3_PO_4_ as the base (entry 4). [Ru(MesCO_2_)_2_(*p*-cymene)] as the catalyst furnished the corresponding product 3a in a satisfying yield of 72% (entry 5). Interestingly, the use of the dimeric [RuCl_2_(*p*-cymene)]_2_ resulted in a lower yield of 32% which can be improved to 79% by adding a catalytic amount of NaOAc (entry 6), highlighting the importance of carboxylate assistance for efficient C–H activation.^18^ However, additional studies indicated a broader applicability and efficacy with [Ru(OAc)_2_(*p*-cymene)] as compared to [RuCl_2_(*p*-cymene)]_2_ in combination with acetate additives (see SI for detailed optimization). Other ruthenium sources, such as Ru_3_(CO)_12_ and RuCl_3_·*n*H_2_O completely failed to give the desired product 3a (Table 1, entry 7). Alternative electrophilic aryl donors proved viable, albeit with reduced yields of the desired product 3a (entry 8). Control experiments reflected the crucial importance of the base and light irradiation (entries 9–11). The essential role of the ruthenium catalyst was also confirmed (entry 12).

**Table 1:** Optimization studies^a^

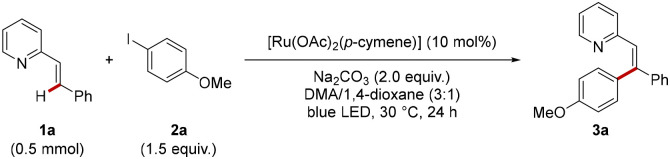		
Entry	Deviation from standard conditions	Yield 3a^b^ (%)
1	None	81
2	1,4-Dioxane or DMA as sole solvent	31/48
3^c^	DMA/THF (3 : 1) as solvent	72
4	K_2_CO_3_ or K_3_PO_4_ as base	74/65
5	[Ru(MesCO_2_)_2_(*p*-cymene)] as catalyst	72
6	[RuCl_2_(*p*-cymene)]_2_ as catalyst	(32), 79^d^
7	RuCl_3_·10H_2_O or Ru_3_(CO)_12_ as catalyst	0^d^/0^d^
8	Ar–Br, Ar–Cl, or Ar–OTf as arylating reagent	44/27/(19)
9	No light	(9)
10	Without Na_2_CO_3_	(12)
11	Under air	0
12	Without [Ru(OAc)_2_(*p*-cymene)]	0

a Reaction conditions: 1a (0.5 mmol), 2a (0.75 mmol), [Ru(OAc)_2_(*p*-cymene)] (10 mol%), Na_2_CO_3_ (2.0 equiv.), N_2_, 30 °C, 24 h, DMA/1,4-dioxane (3 : 1, 1.5 mL), 450 nm blue LED, 30 °C (fan cooling).

b Isolated yield. Yield in parentheses was determined by ^1^H-NMR using CH_2_Br_2_ as the internal standard.

c K_2_CO_3_ (2.0 equiv.) as base.

d 30 mol% NaOAc as additive.

With the optimized reaction conditions for the alkene C–H arylation at room temperature in hand, a viable substrate scope for the photo-induced alkene functionalization was investigated with different aryl iodides. The room-temperature direct arylation proved to be compatible with a large variety of functional groups on electron-rich as well as electron-deficient arenes, including ether (3a), ester (3f, 3h), ketone (3i), and cyano (3j). Halo derivatives were also well tolerated, furnishing the desired products 3l–3n, featuring valuable electrophilic handles. The synthesis potential of our photo-alkene arylation at room temperature was reflected by the efficient late-stage diversification of (−)-menthol (3w), (−)-myrtenol (3x), naproxen (3y), and indomethacin (3z) derivatives (Scheme 2A).

**Scheme 2:**
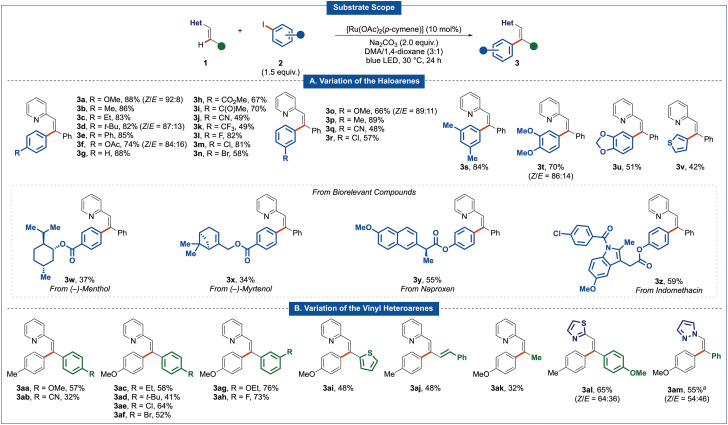
Robustness of the photo-induced C–H activation at room temperature of (A) different aryl iodides and (B) vinyl-heteroarenes. Reaction conditions: 1 (0.5 mmol), aryl iodide 2 (0.75 mmol), [Ru(OAc)_2_(*p*-cymene)] (10 mol%), DMA/1,4-dioxane (1.5 mL), 30 °C (fan cooling). The product was obtained as single isomer. If the product was obtained as a mixture of (*Z*) and (*E*) isomers, the ratio is given in parenthesis. More details can be found in the SI. ^*a* ^Performed on 0.25 mmol scale.

Next, we explored the robustness of the photo-ruthenium-catalyzed C–H arylation at room temperature with a set of representative alkenes 1. Again, a variety of sensitive functional groups, such as cyano- and halo-substituted substrates, were well tolerated. Notably, a diene furnished product 3aj with excellent levels of chemo- and site-selectivities. It is noteworthy that a thiazole also enabled chelation-assisted C–H activation, thereby delivering product 3al in an efficient manner. Additionally, pyrazole was also identified as viable orienting group, furnishing the desired product 3am (Scheme 2B).

Next, we explored the robustness of the photo-ruthenium-catalyzed C–H arylation at room temperature with a set of representative alkenes 1. Again, a variety of sensitive functional groups, such as cyano- and halo-substituted substrates, were well tolerated. Notably, a diene furnished product 3aj with excellent levels of chemo- and site-selectivities. It is noteworthy that a thiazole also enabled chelation-assisted C–H activation, thereby delivering product 3al in an efficient manner. Additionally, pyrazole was also identified as viable orienting group, furnishing the desired product 3am (Scheme 2B).

To gain insights into the catalyst's mode of action, we first probed the performance of independently synthesized cyclometallated complex Ru-I. Interestingly, under photoexcitation, the desired product 3a was obtained in 77% yield, indicating that a cyclometallated intermediate is likely involved. In contrast, a significantly diminished efficacy was observed in the absence of light at ambient temperature or at 30 °C, highlighting the crucial role of light beyond catalyst activation through *p*-cymene decoordination, being suggestive of photo-excitation of a cyclometallated ruthenium species (Scheme 3A). Second, radical scavenger experiments were performed. While BHT and TEMPO led to strongly diminished yields, galvinoxyl and DPPH completely inhibited the reaction, and the corresponding radical adducts were observed by HRMS, supporting the formation of an aryl radical (Scheme 3B). Third, we analyzed the role of the blue LED irradiation by an on/off experiment, showing a significant inhibition of the reaction in the dark. These findings again suggest that continuous light irradiation is required, hence rendering a sole arene decoordination unlikely to be operative (Scheme 3C). Instead, photo-excitation of a ruthenacycle is more likely. Moreover, a quantum yield of 2% renders a radical chain mechanism unlikely to be operative (see SI for details). Additionally, detailed UV/Vis spectroscopy studies (see SI, Fig. S2) support the formation of a new ruthenium species upon light irradiation. Thus, the generation of a cyclometalated intermediate, most likely, plays a key role in the catalytic cycle.

**Scheme 3:**
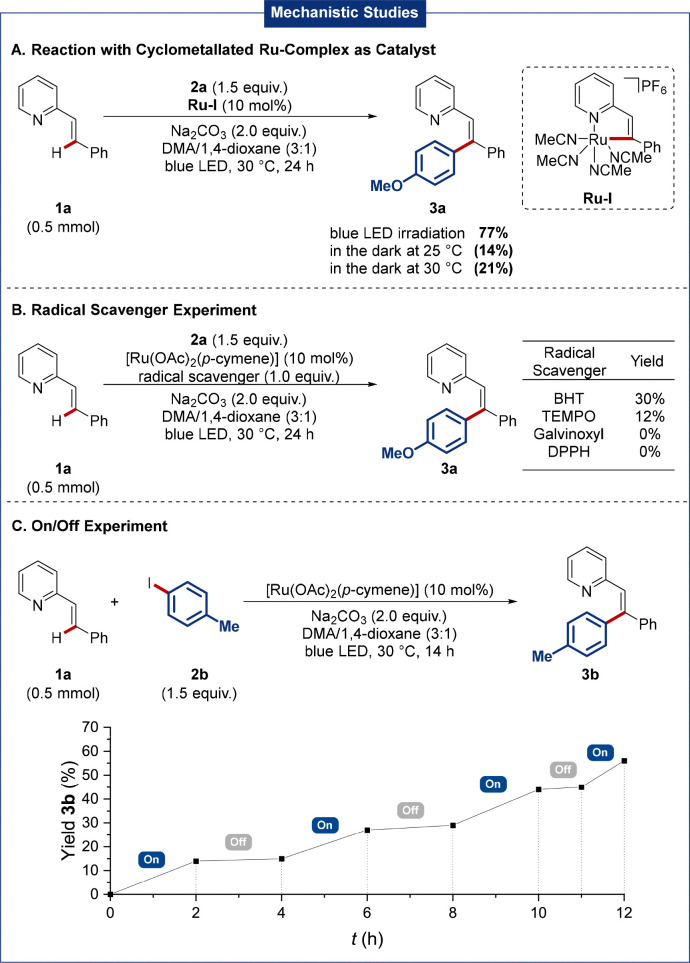
Key mechanistic findings including (A) reactions with a ruthenacycle, (B) radical scavenger experiments, and (C) on/off experiment.

Based on these mechanistic studies and literature precedents,^17^ a plausible mechanism is proposed in Scheme 4. A carboxylate-assisted C–H activation first forms complex A, followed by coordination of the aryl iodide to give intermediate B,^17*c*^ which is excited by light to form singlet species B*via MLCT. Intersystem crossing (ISC) then generates the triplet state B**, enabling SET to generate an aryl radical and complex C. Radical recombination results in complex D, followed by reductive elimination to afford the desired product 3a and complex E after ligand exchange. Afterwards, further C–H activation leads to the regeneration of the active species A.

**Scheme 4:**
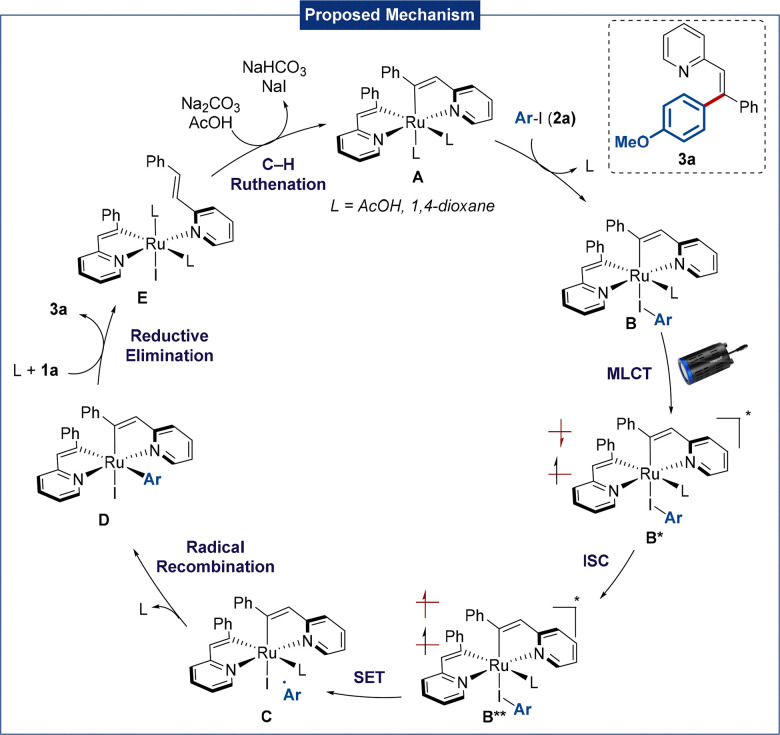
Mechanistic proposal for the ruthenium-catalyzed photoinduced C–H-arylation.

In conclusion, we reported on a photo-induced C–H-arylation of alkenes. Contrary to previous state of the art, the ruthenium catalysis bypasses high temperature or the use of Grignard reagents through photoexcitation and is thereby compatible with a large variety of otherwise sensitive functional groups. Thereby, 39 multi-substituted heterocycle-incorporated olefins were assembled, including several biorelevant derivatives. Mechanistic studies highlighted the crucial role of blue light in this transformation.

The authors gratefully acknowledge generous support by the DZHK, the DFG (Gottfried Wilhelm Leibniz award to L. A.), the ERC Advanced Grant no. 101021358 (L. A.), and DAAD (NAMASTE+) for the financial support (T. M.).

## Conflicts of interest

There are no conflicts to declare.

## Supplementary Material

CC-061-D5CC04527D-s001

## Data Availability

All data associated with this study are available in the article and supplementary information (SI). Supplementary information is available. See DOI: https://doi.org/10.1039/d5cc04527d.
