# Supplementary material for: Photo-induced ruthenium-catalyzed alkene C–H-arylation at room temperature
Source: Chem Commun (Camb). 2025 Sep 30;61(87):16954–7. doi: 10.1039/d5cc04527d (PMC12498270; doi:10.1039/d5cc04527d)
Supplement: CC-061-D5CC04527D-s001 [file CC-061-D5CC04527D-s001.pdf]

Supplementary Information (SI) for ChemComm.
This journal is © The Royal Society of Chemistry 2025

Supporting Information

Photo-Induced Ruthenium-Catalyzed Alkene C–H-Arylation at Room Temperature

Tanumoy Mandal,[a] Stéphane Golling,[a] Sven Trienes[a,b] and Lutz Ackermann*[a,b]

[a] Wöhler Research Institute for Sustainable Chemistry (WISCh), Georg-August-Universität, Tammannstraße 2, 37077 Göttingen (Germany)

[b] DZHK (German Centre for Cardiovascular Research), Potsdamer Straße 58, 10875 Berlin (Germany)

E-mail: Lutz.Ackermann@chemie.uni-goettingen.de
https://www.ackermann.chemie.uni-goettingen.de/

Contents

1 General Remarks **3**

2 General Procedure: Photo-Induced Ruthenium-Catalyzed C–H Arylation **4**

3 Optimization Studies **5**

4 Characterization Data **10**

5 Comparison Chemoselectivity Thermal vs. Photochemical Conditions **30**

6 Comparison Ruthenium-Catalysts **32**

7 Mechanistic Studies **33**

7.1 Reaction with Cyclometallated Ru-Complex as Catalyst 33

7.2 Radical Scavenger Experiments 35

7.3 On/Off Experiment 36

7.4 UV/Vis Spectroscopy 38

7.5 Determination of Quantum Yield 40

8 Isomerization Study **43**

9 References **44**

10 NMR Spectra **45**

1 General Remarks

Catalytic reactions were performed under a N_2 atmosphere using pre-dried glassware and standard Schlenk techniques. The reaction temperature was measured by digital thermometer PCE-T 390, which was in the range of 30 to 35 °C. All the solvents were dried over sodium and freshly distilled under N_2. Alkene starting materials were synthesized according to previously described methods.[1] Other substrates were obtained from commercial sources and were used without further purification. Yields refer to isolated compounds estimated to be >95% pure as determined by ^{1}H-NMR and ^{13}C-NMR. TLC was performed on Merck TLC Silica Gel 60 F254 with detection under UV light at 254 nm. Chromatographic separations were carried out on Merck Geduran SI-60 (0.040–0.063 mm, 230–400 mesh ASTM). IR spectra were recorded on a Bruker FT-IR alpha-P device. EI-MS was recorded on Jeol AccuTOF at 70 eV; ESI-MS was recorded on Bruker Daltonik micrOTOF and maXis. The ratios of mass to charge (*m*/*z*) are reported and the intensity relative to the base peak (I = 100) is given in parenthese. Melting points (m.p.) were measured on Stuart® melting point apparatus SMP3, values are uncorrected. Nuclear magnetic resonance (NMR) spectroscopy was performed at 300, 400 or 500 MHz (^{1}HNMR), 75, 100 or 125 MHz (^{13}C-NMR, APT), 282 or 376 MHz (^{19}F-NMR) and 162 MHz (^{31}P{1H}-NMR) on Bruker Avance III HD 300, Avance III 300, Avance III 400, Avance III HD 400, Avance Neo 400, Avance III HD 500, Varian Unity-300 and Inova 500 instruments. Chemical shifts (δ) are provided in ppm and spectra referenced to non-deuterated solvent signal. Recycling preparative HPLC system from Japan Analytical Industries (LC-92XX II Series, UV and RI Detector) connected to JAIGEL 2HH series column with HPLC grade chloroform.

2 General Procedure: Photo-Induced Ruthenium-Catalyzed C–H Arylation

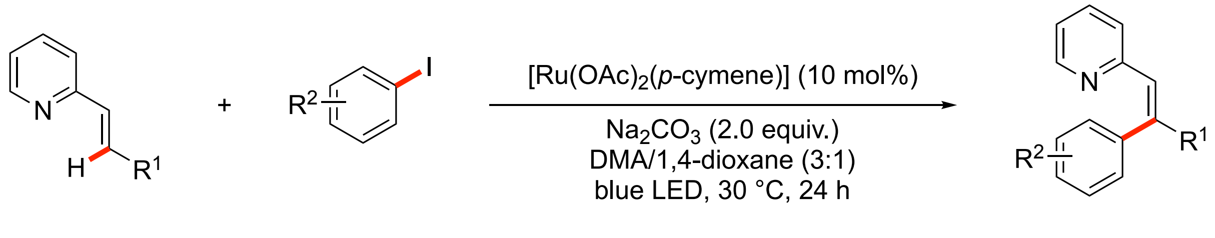

1 (0.5 mmol) **2** (1.5 equiv) **3**

(*E*)-2-Styrylpyridine **1** (0.50 mmol), [Ru(OAc)$_2$(*p*-cymene)] (18 mg, 50.0 µmol, 10 mol%), aryl iodide **2** (0.75 mmol, 1.5 equiv.) [in case of liquid aryl iodide, the derivative was added after evacuation] and Na_2CO_3 (106 mg, 1.00 mmol, 2 equiv.) were placed in a 10 mL vial. The vial was capped with a septum and wrapped with parafilm. The vial was evacuated and purged with N_2 three times. A mixture of DMA and 1,4-dioxane (3:1, 1.5 mL) was then added and the mixture was stirred under visible light irradiation with the wavelength of 450 nm (2 × Kessil A360N, temperature was maintained between 30 °C and 33 °C). After 24 h, the resulting mixture was filtered through a pad of silica gel and washed with diethyl ether. The filtrate was concentrated in vacuo. Purification of the residue by column chromatography (SiO_2, *n*-hexane/EtOAc) yielded arylated product **3**.

3 Optimization Studies

Table S1. Optimization of the solvent.[a]

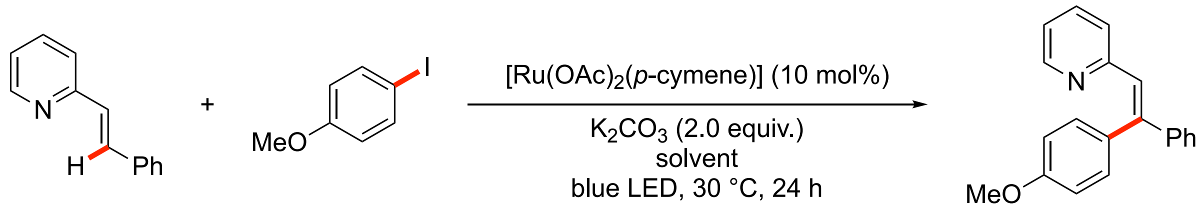

1a **2a** **3a**

Entry	Solvent	Yield
1	1,4-Dioxane	31%
2	DCE	(16%)
3	DMA	48%
4	THF	39%
5	2-Me-THF	37%
6	Toluene	(19%)
7	DMF	31%, (33%)
8	NMP	44%
9	MeCN	27%, (30%)
10	DMSO	n.d.
11	H_2O	(13%)
12	DMA/2-Me-THF (3:1)	57%
13	DMA/2-Me-THF (3:1) + 5 equiv. H_2O	42%
14	DMA/dry THF (from SPS) (3:1)	66%
15	DMA/1,4-dioxane (3:1)	69%
16	DMA/1,4-dioxane (2:1)	65%
17	DMA/1,4-dioxane (1:1)	65%
18	DMA/1,4-dioxane (1:2)	61%
19	DMA/1,4-dioxane (1:3)	58%

20	MeCN/1,4-dioxane (3:1)	(43%)

[a] **1a** (0.5 mmol), **2a** (0.75 mmol), [Ru(OAc)$_2$(*p*-cymene)] (10 mol%), K_2CO_3 (2 equiv.), N_2, 24 h, solvent (2 mL), 450 nm blue LED, isolated yield. Yield in the parentheses was determined by ^{1}H-NMR using CH_2Br_2 as the internal standard.

Table S2. Optimization for the concentration of the reaction.[a]

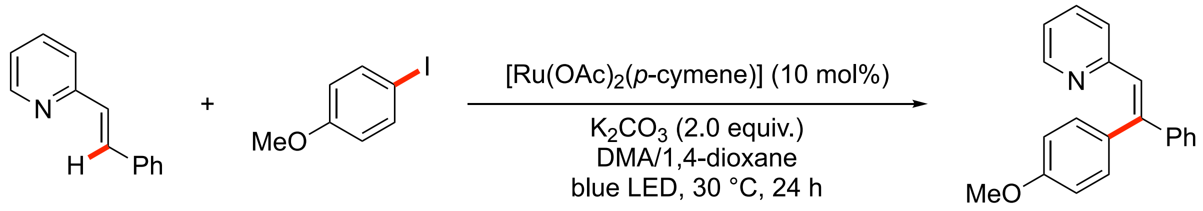

1a **2a** **3a**

Entry	Solvent	Yield
1	DMA/1,4-dioxane (3:1)<br>(2 mL solvent for 0.5 mmol **1a**)	69%
2	DMA/1,4-dioxane (3:1)<br>(1.5 mL solvent for 0.5 mmol **1a**)	74%
3	DMA/dry THF (from SPS) (3:1)<br>(1.5 mL solvent for 0.5 mmol **1a**)	72%
4	DMA/1,4-dioxane (3:1)<br>(1.5 mL solvent for 0.75 mmol **1a**)	68%

[a] **1a** (1 equiv), **2a** (1.5 equiv), [Ru(OAc)$_2$(*p*-cymene)] (10 mol%), K_2CO_3 (2 equiv.), N_2, 24 h, solvent, 450 nm blue LED, isolated yield.

Table S3. Optimization of the catalyst and base.[a]

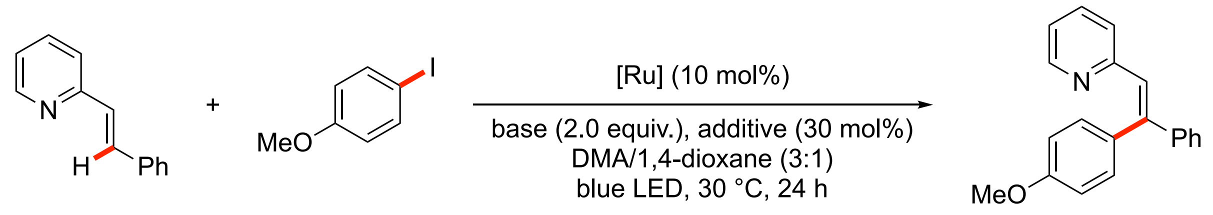

1a **2a** **3a**

Entry	Catalyst	Base	Additive	Yield
1	[Ru(OAc)$_2$(*p*-cymene)]	K_2CO_3	-	74%
2	[Ru(OAc)$_2$(*p*-cymene)]	Li_2CO_3	-	(48%)
**3**	**[Ru(OAc)$_2$(*p*-cymene)]**	**Na_2CO_3**	**-**	**81%**
4	[Ru(OAc)$_2$(*p*-cymene)]	Cs_2CO_3	-	(37%)
5	[Ru(OAc)$_2$(*p*-cymene)]	$NaHCO_3$	-	56% (58%)
6	[Ru(OAc)$_2$(*p*-cymene)]	$KHCO_3$	-	(51%)
7	[Ru(OAc)$_2$(*p*-cymene)]	Na_3PO_4	-	78%
8	[Ru(OAc)$_2$(*p*-cymene)]	K_3PO_4	-	65%
9	[Ru(OAc)$_2$(*p*-cymene)]	Na_2CO_3	PPh_3	n.d.
10	[Ru(MesCO$_2$)$_2$(*p*-cymene)]	Na_2CO_3	-	72%
11	[RuCl$_2$(*p*-cymene)]$_2$	KOAc	-	64%
12	[RuCl$_2$(*p*-cymene)]$_2$	NaOAc	-	73%
13	[RuCl$_2$(*p*-cymene)]$_2$	LiOAc	-	(51%)
14	[RuCl$_2$(*p*-cymene)]$_2$	CsOAc	-	60%%
15	[RuCl$_2$(*p*-cymene)]$_2$	TBAOAc	-	65%
16	[RuCl$_2$(*p*-cymene)]$_2$	$PhCO_2Na$	-	(43%)
17	[RuCl$_2$(*p*-cymene)]$_2$	$AdmCO_2Na$	-	(46%)
18	[RuCl$_2$(*p*-cymene)]$_2$	Na_2CO_3	$MesCO_2H$	53% (54%)
19	[RuCl$_2$(*p*-cymene)]$_2$	Na_2CO_3	(PhO)$_2$P(O)OH	56%
20	[RuCl$_2$(*p*-cymene)]$_2$	Na_2CO_3	(PhO)$_2$P(O)OH + NaOAc	77%
21	[RuCl$_2$(*p*-cymene)]$_2$	Na_2CO_3	NaOAc	79%
22	[RuCl$_2$(*p*-cymene)]$_2$	Na_2CO_3	-	(32%)
23	$RuCl_3\cdot 10H_2O$	Na_2CO_3	NaOAc	n.d.
24	$Ru_3(CO)_{12}$	Na_2CO_3	NaOAc	n.d.
25[b]	[Ru(OAc)$_2$(*p*-cymene)]	Na_2CO_3	-	66%

[a] **1a** (0.5 mmol), **2a** (0.75 mmol), [Ru] species (10 mol%), base (2 equiv.), additive (30 mol%), N_2, 24 h, DMA/1,4-dioxane (3:1, 1.5 mL), 450 nm blue LED, isolated yield. [b] Reaction was carried out using Penn LED photoreactor (blue LED). Yield in the parentheses was determined by ^{1}H-NMR using CH_2Br_2 as the internal standard.

Table S4. Optimization of arylating reagent.[a]

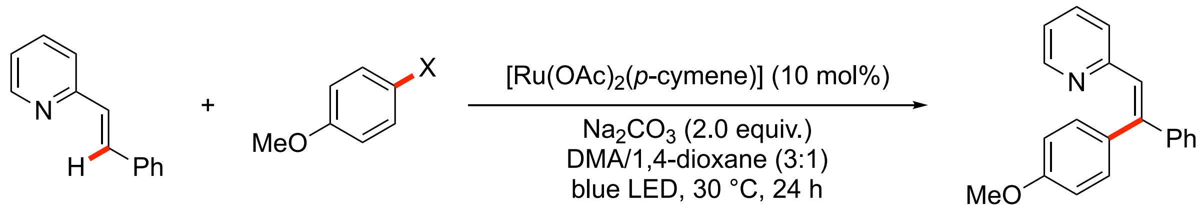

1a **2a** **3a**

Entry	X	Yield
1	I	81%
2	Br	44% (46%)
3	Cl	27%
4	OMs	(16%)
5	OTs	0%
6	OTf	(19%)
7	F	0%

[a] **1a** (0.5 mmol), **2a** (0.75 mmol), [Ru(OAc)$_2$(*p*-cymene)] (10 mol%), Na_2CO_3 (2 equiv.), N_2, 24 h, DMA/1,4-dioxane (3:1, 1.5 mL), 450 nm blue LED, isolated yield. Yield in the parentheses was determined by ^{1}H-NMR using CH_2Br_2 as the internal standard.

Table S5. Control experiments.[a]

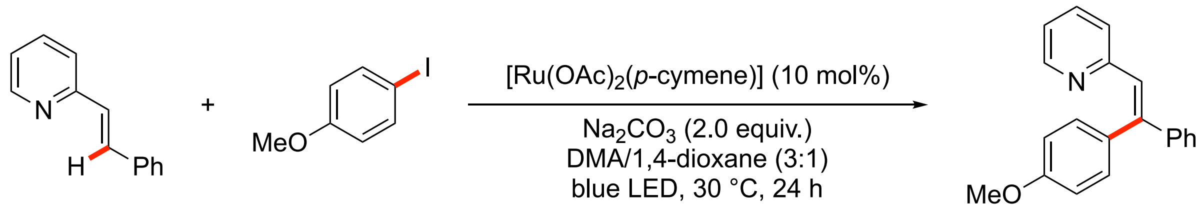

1a **2a** **3a**

Entry	Variation from standard condition	Yield
1	In dark	(9%)
2	Under air	n.d.
3	Without base	(12%)
4	Without catalyst	n.d.
5	$[RuCl_2(p\text{-cymene})]_2$ was used	(32%)
6[b]	**Ru-I**, in dark	(14%)
7[b]	**Ru-II**, in dark	(7%)
8[b]	**Ru-I**, in dark, 30 ºC	(21%)
9[b]	**Ru-I**, under light irradiation	77%
10	**Ru-I**, under light irradiation	(34%)

[a] **1a** (0.5 mmol), **2a** (0.75 mmol), $[Ru(OAc)_2(p\text{-cymene})]$ (10 mol%), base (2 equiv.), additive (30 mol%) N_2, 24 h, DMA/1,4-dioxane (3:1, 1.5 mL), 450 nm blue LED, isolated yield. [b] 30% NaOAc was used as an additive. Yield in the parentheses was determined by ^{1}H-NMR using CH_2Br_2 as the internal standard.

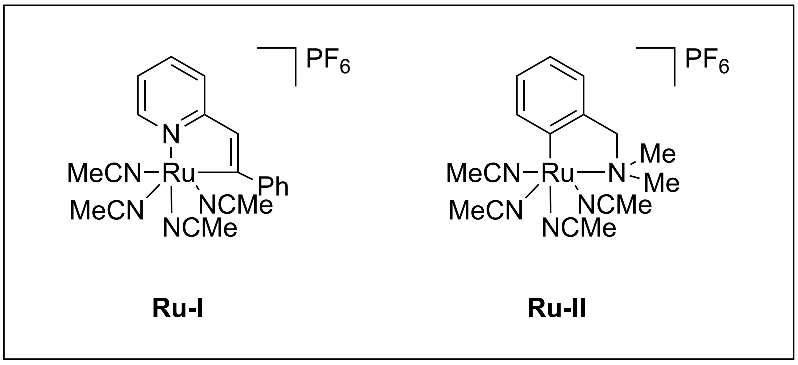

4 Characterization Data

(*Z*)-2-(2-(4-Methoxyphenyl)-2-phenylvinyl)pyridine (3a)

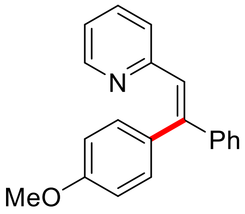

The general procedure was followed using (*E*)-2-styrylpyridine (90.5 mg, 0.50 mmol) and 1-iodo-4-methoxybenzene (176 mg, 0.75 mmol). After 24 h, purification by column chromatography (*n*-hexane/EtOAc 7:1) yielded **3a** (116 mg, 81%) as a pale yellow solid.

Note: The crude product was obtained as a mixture of (*Z*)- and (*E*)-isomer. Analysis by ^{1}H NMR spectroscopy showed a ratio *Z*/*E* = 92:8.

M.p.: 115 °C;

^{1}H NMR (400 MHz, $CDCl_3$): δ = 8.53 (ddd, *J* = 4.9, 1.8, 0.9 Hz, 1H), 7.43 – 7.35 (m, 2H), 7.34 – 7.22 (m, 4H), 7.12 (dd, *J* = 6.2, 2.4 Hz, 3H), 6.96 (ddd, *J* = 7.5, 4.9, 1.1 Hz, 1H), 6.91 – 6.83 (m, 2H), 6.77 (d, *J* = 8.1 Hz, 1H), 3.81 (s, 3H) ppm;

^{13}C NMR (101 MHz, $CDCl_3$): δ = 159.3 (C_q), 156.9 (C_q), 149.3 (CH), 145.5 (C_q), 142.9 (C_q), 135.3 (CH), 132.2 (C_q), 131.4 (CH), 128.4 (CH), 128.2 (CH), 128.0 (CH), 127.9 (CH), 123.7 (CH), 121.0 (CH), 114.1 (CH), 55.2 (CH_3) ppm;

IR (ATR): $\tilde{\nu}$ = 3055, 1606, 1508, 1460, 1243, 1031, 834, 768, 727, 514 cm^{-1};

HR-MS (ESI): *m*/*z* calcd. for $C_{20}H_{18}NO^+$ $[M+H]^+$ 288.1383, found 288.1386.

The spectral data are in accordance with those reported in the literature.[2]

(*Z*)-2-(2-Phenyl-2-(*p*-tolyl)vinyl)pyridine (3b)

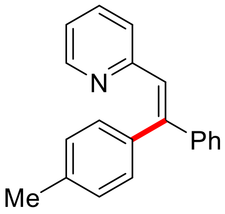

The general procedure was followed using (*E*)-2-styrylpyridine (90.5 mg, 0.50 mmol) and 1-iodo-4-methylbenzene (164 mg, 0.75 mmol). After 24 h, purification by column chromatography (*n*-hexane/EtOAc 9:1) yielded **3b** (117 mg, 86%) as a colorless solid.

M.p.: 90 °C;

^{1}H NMR (400 MHz, $CDCl_3$) δ = 8.59 – 8.50 (m, 1H), 7.42 – 7.35 (m, 2H), 7.34 – 7.26 (m, 4H), 7.16 (d, *J* = 7.3 Hz, 3H), 7.10 (d, *J* = 8.2 Hz, 2H), 7.01 – 6.94 (m, 1H), 6.75 (d, *J* = 8.1 Hz, 1H), 2.39 (s, 3H) ppm;

^{13}C NMR (101 MHz, $CDCl_3$) δ = 156.8 (C_q), 149.3 (CH), 145.9 (C_q), 142.8 (C_q), 137.6 (C_q), 136.9 (C_q), 135.3 (CH), 130.1 (CH), 129.5 (CH), 128.5 (CH), 128.3 (CH), 128.0 (CH), 127.9 (CH), 123.8 (CH), 121.1 (CH), 21.4 (CH_3) ppm;

IR (ATR): $\tilde{\nu}$ = 3051, 1582, 1435, 768, 736, 695, 593, 523, 490 cm^{-1};

HR-MS (ESI): *m*/*z* calcd. for $C_{20}H_{18}N^+$ $[M+H]^+$ 272.1434, found 272.1435.

The spectral data are in accordance with those reported in the literature. [2]

(*Z*)-2-(2-(4-Ethylphenyl)-2-phenylvinyl)pyridine (3c)

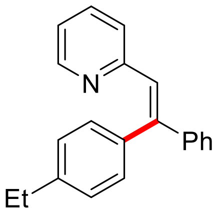

The general procedure was followed using (*E*)-2-styrylpyridine (90.5 mg, 0.50 mmol) and 1-ethyl-4-iodobenzene (174 mg, 0.75 mmol). After 24 h, purification by column chromatography (*n*-hexane/EtOAc 9:1) yielded **3c** (118 mg, 83%) as a pale-yellow sticky oil.

^{1}H NMR (400 MHz, $CDCl_3$) δ = 8.54 (ddd, *J* = 4.8, 1.9, 1.0 Hz, 1H), 7.41 – 7.36 (m, 2H), 7.35 – 7.25 (m, 4H), 7.22 – 7.08 (m, 5H), 6.97 (ddd, J = 7.5, 4.9, 1.1 Hz, 1H), 6.73 (d, *J* = 8.1 Hz, 1H), 2.70 (q, *J* = 7.6 Hz, 2H), 1.28 (t, *J* = 7.6 Hz, 3H) ppm;

^{13}C NMR (101 MHz, $CDCl_3$) δ = 156.9 (C_q), 149.3 (CH), 146.0 (C_q), 143.9 (C_q), 142.8 (C_q), 137.1 (C_q), 135.3 (CH), 130.1 (CH), 128.5 (CH), 128.3 (CH), 128.2 (CH), 128.0 (CH), 127.9 (CH), 123.8 (CH), 121.1 (CH), 28.7 (CH_2), 15.5 (CH_3) ppm;

IR (ATR): $\tilde{\nu}$ = 2928, 1584, 1435, 767, 736, 693, 592, 516, 493 cm^{-1};

HR-MS (ESI): *m*/*z* calcd. for $C_{21}H_{20}N^+$ $[M+H]^+$ 286.1590, found 286.1593.

(*Z*)-2-(2-(4-(*tert*-Butyl)phenyl)-2-phenylvinyl)pyridine (3d)

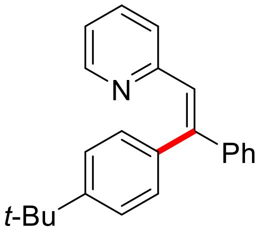

The general procedure was followed using (*E*)-2-styrylpyridine (90.5 mg, 0.50 mmol) and 1-(*tert*-butyl)-4-iodobenzene (195 mg, 0.75 mmol). After 24 h, purification by column chromatography (*n*-hexane/EtOAc 9:1) yielded **3d** (111 mg, 71%) as a pale-yellow sticky oil.

Note: The crude product was obtained as a mixture of (*Z*)- and (*E*)-isomer. Analysis by ^{1}H NMR spectroscopy showed a ratio *Z*/*E* = 87:13.

^{1}H NMR (400 MHz, $CDCl_3$) δ = 8.54 (ddd, *J* = 5.0, 1.8, 1.0 Hz, 1H), 7.44 – 7.24 (m, 8H), 7.17 – 7.08 (m, 3H), 6.98 (ddd, *J* = 7.5, 4.8, 1.1 Hz, 1H), 6.69 (dt, *J* = 8.0, 1.1 Hz, 1H), 1.36 (s, 9H) ppm;

^{13}C NMR (101 MHz, $CDCl_3$) δ = 156.9 (C_q), 151.0 (C_q), 149.3 (CH), 146.0 (C_q), 142.8 (C_q), 136.8 (C_q), 135.3 (CH), 129.8 (CH), 128.6 (CH), 128.3 (CH), 128.1 (CH), 127.9 (CH), 125.7 (CH), 123.8 (CH), 121.2 (CH), 34.8 (C_q), 31.5 (CH_3) ppm;

IR (ATR): $\tilde{\nu}$ = 2960, 1582, 1435, 768, 736, 695, 590, 522, 404 cm^{-1};

HR-MS (ESI): *m*/*z* calcd. for $C_{23}H_{24}N^+$ $[M+H]^+$ 314.1903, found 314.1895.

(*Z*)-2-(2-([1,1'-Biphenyl]-4-yl)-2-phenylvinyl)pyridine (3e)

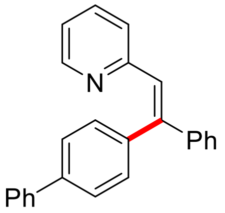

The general procedure was followed using (*E*)-2-styrylpyridine (90.5 mg, 0.50 mmol) and 4-iodo-1,1'-biphenyl (211 mg, 0.75 mmol). After 24 h, purification by column chromatography (*n*-hexane/EtOAc 9:1) yielded **3e** (142 mg, 85%) as a pale-yellow solid.

M.p.: 106 °C;

^{1}H NMR (400 MHz, $CDCl_3$) δ = 8.57 (dd, *J* = 4.9, 1.6 Hz, 1H), 7.73 – 7.57 (m, 4H), 7.52 – 7.26 (m, 12H), 7.21 (s, 1H), 7.09 – 6.97 (m, 1H), 6.84 (d, *J* = 8.0 Hz, 1H) ppm;

^{13}C NMR (101 MHz, $CDCl_3$) δ = 156.7 (C_q), 149.4 (CH), 145.6 (C_q), 142.6 (C_q), 140.6 (C_q), 140.5 (C_q), 139.0 (C_q), 138.0 (CH), 135.5 (CH), 130.7 (CH), 128.9 (CH), 128.4 (CH), 128.2 (CH), 128.0 (CH), 127.6 (CH), 127.4 (CH), 127.0 (CH), 123.9 (CH), 121.3 (CH) ppm;

IR (ATR): $\tilde{\nu}$ = 3052, 1581, 1435, 764, 734, 693, 565, 515, 4405 cm^{-1};

HR-MS (ESI): *m*/*z* calcd. for $C_{25}H_{19}N^+$ $[M+Na]^+$ 356.1410, found 356.1400.

(*Z*)-4-(1-Phenyl-2-(pyridin-2-yl)vinyl)phenyl acetate (3f)

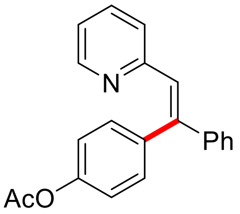

The general procedure was followed using (*E*)-2-styrylpyridine (90.5 mg, 0.50 mmol) and 4-iodophenyl acetate (196 mg, 0.75 mmol). After 24 h, purification by column chromatography (*n*-hexane/EtOAc 4:1 to 3:1) yielded **3f** (101 mg, 64%) as a pale-yellow sticky oil.

Note: The crude product was obtained as a mixture of (*Z*)- and (*E*)-isomer. Analysis by ^{1}H NMR spectroscopy showed a ratio *Z*/*E* = 84:16.

^{1}H NMR (400 MHz, $CDCl_3$) δ = 8.54 – 8.43 (m, 1H), 7.65 – 7.59 (m, 2H), 7.41 (td, *J* = 7.8, 1.8 Hz, 1H), 7.34 (td, *J* = 4.2, 1.7 Hz, 4H), 7.32 – 7.27 (m, 3H), 7.18 (s, 1H), 7.05 (ddd, *J* = 7.6, 4.9, 1.1 Hz, 1H), 6.76 (dt, *J* = 7.9, 1.1 Hz, 1H) 2.29 (s, 3H) ppm;

^{13}C NMR (101 MHz, $CDCl_3$) δ = 169.2 (C_q), 156.4 (C_q), 150.3 (C_q), 149.3 (CH), 144.9 (C_q), 142.3 (C_q), 137.4 (C_q), 135.5 (CH), 131.3 (CH), 129.0 (CH), 128.3 (CH), 128.2 (CH), 127.9 (CH), 123.8 (CH), 121.9 (CH), 121.3 (CH), 21.2 (CH_3) ppm;

IR (ATR): $\tilde{\nu}$ = 3053, 1762, 1583, 1434, 1189, 908, 852, 770, 735, 695, 585, 406 cm^{-1};

HR-MS (ESI): *m*/*z* calcd. for $C_{21}H_{18}NO_2^+$ $[M+H]^+$ 316.1332, found 316.1334.

2-(2,2-Diphenylvinyl)pyridine (3g)

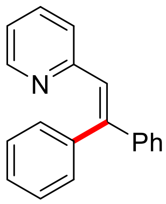

The general procedure was followed using (*E*)-2-styrylpyridine (90.5 mg, 0.50 mmol) and iodobenzene (153 mg, 0.75 mmol). After 24 h, purification by column chromatography (*n*-hexane/EtOAc 9:1) yielded **3g** (113 mg, 88%) as a white solid.

M.p.: 191 °C;

^{1}H NMR (400 MHz, $CDCl_3$) δ = 8.54 (ddd, *J* = 4.9, 1.9, 1.0 Hz, 1H), 7.40 – 7.32 (m, 7H), 7.32 – 7.27 (m, 2H), 7.26 – 7.18 (m, 2H), 7.19 (s, 1H), 6.98 (ddd, *J* = 7.5, 4.9, 1.1 Hz, 1H), 6.69 (dt, *J* = 8.1, 1.1 Hz, 1H) ppm;

^{13}C NMR (101 MHz, $CDCl_3$) δ = 156.7 (C_q), 149.4 (CH), 145.9 (C_q), 142.5 (C_q), 140.0 (C_q), 135.4 (CH), 130.2 (CH), 128.8 (CH), 128.7 (CH), 128.3 (CH), 128.1 (CH), 127.9 (CH), 127.8 (CH), 123.8 (CH), 121.3 (CH) ppm;

IR (ATR): $\tilde{\nu}$ = 3053, 1586, 1435, 992, 770, 698, 598, 521, 404 cm^{-1};

HR-MS (ESI): *m*/*z* calcd. for $C_{19}H_{16}N^+$ $[M+H]^+$ 258.1277, found 258.1279.

The spectral data are in accordance with those reported in the literature.[2]

Methyl (*Z*)-4-(1-phenyl-2-(pyridin-2-yl)vinyl)benzoate (3h)

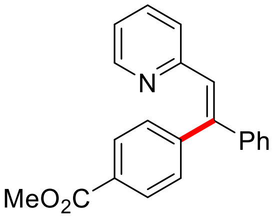

The general procedure was followed using (*E*)-2-styrylpyridine (90.5 mg, 0.50 mmol) and methyl 4-iodobenzoate (197 mg, 0.75 mmol). After 24 h, purification by column chromatography (*n*-hexane/EtOAc 4:1) yielded **3h** (105 mg, 67%) as a brown liquid.

^{1}H NMR (400 MHz, $CDCl_3$) δ = 8.57 – 8.46 (m, 1H), 8.07 – 7.94 (m, 2H), 7.33 – 7.27 (m, 8H), 7.19 (s, 1H), 7.00 (ddd, *J* = 7.5, 4.9, 1.1 Hz, 1H), 6.70 (d, *J* = 8.1 Hz, 1H), 3.93 (s, 3H) ppm;

^{13}C NMR (101 MHz, $CDCl_3$) δ = 167.0 (C_q), 156.2 (C_q), 149.5 (CH), 145.1 (C_q), 144.9 (C_q), 142.0 (C_q), 135.6 (CH), 130.4 (CH), 130.1 (CH), 129.5 (C_q), 129.4 (CH), 128.5 (CH), 128.4 (CH), 127.9 (CH), 124.0 (CH), 121.6 (CH), 52.3 (CH_3) ppm;

IR (ATR): $\tilde{\nu}$ = 3053, 1719, 1583, 1435, 1277, 1106, 772, 702, 577, 520, 405 cm^{-1};

HR-MS (ESI): *m*/*z* calcd. for $C_{21}H_{18}NO_2^+$ $[M+H]^+$ 316.1332, found 316.1336.

(*Z*)-1-(4-(1-Phenyl-2-(pyridin-2-yl)vinyl)phenyl)ethan-1-one (3i)

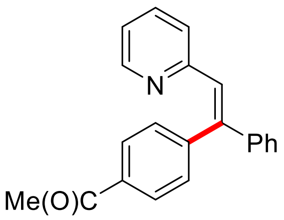

The general procedure was followed using (*E*)-2-styrylpyridine (90.5 mg, 0.50 mmol) and 1-(4-iodophenyl)ethan-1-one (185 mg, 0.75 mmol). After 24 h, purification by column chromatography (*n*-hexane/EtOAc 4:1) yielded **3i** (110 mg, 74%) as a pale-yellow solid.

M.p.: 117 °C;

^{1}H NMR (400 MHz, $CDCl_3$) δ = 8.52 (dd, *J* = 5.0, 1.6 Hz, 1H), 7.93 (d, *J* = 8.1 Hz, 2H), 7.36 – 7.30 (m, 8H), 7.19 (s, 1H), 7.01 (dd, *J* = 7.5, 4.9 Hz, 1H), 6.72 (d, *J* = 8.0 Hz, 1H), 2.62 (s, 3H) ppm;

^{13}C NMR (101 MHz, $CDCl_3$) δ = 197.9 (C_q), 156.1 (C_q), 149.5 (CH), 145.4 (C_q), 144.9 (C_q), 142.0 (C_q), 136.4 (C_q), 135.7 (CH), 130.7 (CH), 129.5 (CH), 128.8 (CH), 128.5 (CH), 128.4 (CH), 127.9 (CH), 124.0 (CH), 121.6 (CH), 26.8 (CH_3) ppm;

IR (ATR): $\tilde{\nu}$ = 3053, 1681, 1605, 1433, 1263, 827, 766, 696, 521, 404 cm^{-1};

HR-MS (ESI): *m*/*z* calcd. for $C_{21}H_{17}NONa^+$ $[M+Na]^+$ 300.1202, found 300.1204.

The spectral data are in accordance with those reported in the literature. [2]

(*Z*)-4-(1-Phenyl-2-(pyridin-2-yl)vinyl)benzonitrile (3j)

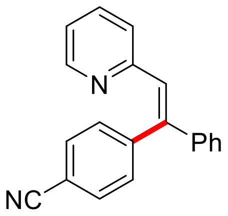

The general procedure was followed using (*E*)-2-styrylpyridine (90.5 mg, 0.50 mmol) and 4-iodobenzonitrile (173 mg, 0.75 mmol). After 24 h, purification by column chromatography (*n*-hexane/EtOAc 6:1 to 3:1) yielded **3j** (69 mg, 49%) as a pale-yellow solid.

M.p.: 108 °C;

^{1}H NMR (400 MHz, $CDCl_3$) δ = 8.54 – 8.43 (m, 1H), 7.65 – 7.59 (m, 2H), 7.41 (td, *J* = 7.8, 1.8 Hz, 1H), 7.34 (td, *J* = 4.2, 1.7 Hz, 4H), 7.32 – 7.27 (m, 3H), 7.18 (s, 1H), 7.05 (ddd, *J* = 7.6, 4.9, 1.1 Hz, 1H), 6.76 (dt, *J* = 7.9, 1.1 Hz, 1H) ppm;

^{13}C NMR (101 MHz, $CDCl_3$) δ = 155.7 (C_q), 149.6 (CH), 145.4 (C_q), 144.3 (C_q), 141.6 (C_q), 135.9 (CH), 132.5 (CH), 131.3 (CH), 129.8 (CH), 128.7 (CH), 128.6 (CH) (CH), 127.9 (CH), 124.2 (CH), 121.9 (CH), 118.9 (C_q), 111.6 (C_q) ppm;

IR (ATR): $\tilde{\nu}$ = 3053, 2224, 1583, 1436, 1151, 853, 768, 696, 589, 520, 413 cm^{-1};

HR-MS (ESI): *m*/*z* calcd. for $C_{20}H_{15}N_2^+$ $[M+H]^+$ 283.1230, found 283.1232.

(*Z*)-2-(2-Phenyl-2-(4-(trifluoromethyl)phenyl)vinyl)pyridine (3k)

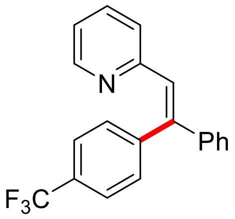

The general procedure was followed using (*E*)-2-styrylpyridine (90.5 mg, 0.50 mmol) and 1-iodo-4-(trifluoromethyl)benzene (204 mg, 0.75 mmol). After 24 h, purification by column chromatography (*n*-hexane/EtOAc 11:1 to 9:1) yielded **3k** (79 mg, 49%) as a pale-yellow sticky oil.

^{1}H NMR (400 MHz, $CDCl_3$) δ = 8.53 (d, *J* = 4.8 Hz, 1H), 7.60 (d, *J* = 8.1 Hz, 2H), 7.41 – 7.30 (m, 8H), 7.20 (s, 1H), 7.03 (ddd, *J* = 7.5, 4.9, 1.2 Hz, 1H), 6.72 (d, *J* = 8.0 Hz, 1H) ppm;

^{13}C NMR (101 MHz, $CDCl_3$) δ = 156.1 (C_q), 149.6 (CH), 144.5 (C_q) 143.9 (C_q), 141.9 (C_q), 135.7 (CH), 130.7 (CH), 129.9 (C_q, q, $^2J_{C–F}$ = 32.8 Hz), 129.1 (CH), 128.6 (CH), 128.5 (CH), 127.9 (CH), 125.5 (CH, q, $^3J_{C–F}$ = 3.7 Hz), 123.9 (C_q, q, $^1J_{C–F}$ = 232.4 Hz), 123.0 (CH), 121.5 (CH) ppm;

^{19}F NMR (377 MHz, $CDCl_3$) δ = -62.43 ppm;

IR (ATR): $\tilde{\nu}$ = 3055, 1582, 1463, 1320, 1163, 1059, 851, 767, 694, 520, 404 cm^{-1};

HR-MS (ESI): *m*/*z* calcd. for $C_{20}H_{15}F_3N^+$ $[M+H]^+$ 326.1151, found 326.1155.

The spectral data are in accordance with those reported in the literature.[2]

(*Z*)-2-(2-(4-Fluorophenyl)-2-phenylvinyl)pyridine (3l)

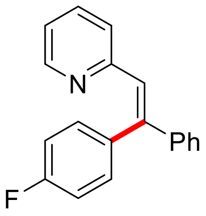

The general procedure was followed using (*E*)-2-styrylpyridine (90.5 mg, 0.50 mmol) and 1-fluoro-4-iodobenzene (167 mg, 0.75 mmol). After 24 h, purification by column chromatography (*n*-hexane/EtOAc 9:1) yielded **3l** (113 mg, 82%) as a white solid.

M.p.: 108 °C;

^{1}H NMR (400 MHz, $CDCl_3$) δ = 8.54 (ddd, *J* = 5.0, 1.8, 1.0 Hz, 1H), 7.33 (ddt, *J* = 10.4, 5.2, 2.0 Hz, 6H), 7.22 – 7.13 (m, 3H), 7.07 – 6.97 (m, 3H), 6.73 (d, *J* = 8.1 Hz, 1H) ppm;

^{13}C NMR (101 MHz, $CDCl_3$) δ = 162.5 (C_q, d, $^1J_{C–F}$ = 249.2 Hz), 156.5 (C_q), 149.5 (CH), 144.8 (C_q), 142.5 (C_q), 135.8 (C_q), 135.5 (CH), 132.0 (CH, d, $^3J_{C–F}$ = 8.0 Hz), 129.0 (CH), 128.4 (CH), 128.3 (CH), 127.9 (CH), 123.8 (CH), 121.4 (CH), 115.8 (CH, d, $^2J_{C–F}$ = 21.4 Hz) ppm;

^{19}F NMR (282 MHz, CDCl3) δ = -113.9 ppm;

IR (ATR): $\tilde{\nu}$ = 3053, 1586, 1500, 1435, 1221, 1153, 822, 768, 694, 521, 498, 405 cm^{-1};

HR-MS (ESI): *m*/*z* calcd. for $C_{19}H_{15}FN^+$ $[M+H]^+$ 276.1183, found 276.1183.

The spectral data are in accordance with those reported in the literature.[2]

(*Z*)-2-(2-(4-Chlorophenyl)-2-phenylvinyl)pyridine (3m)

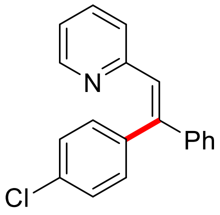

The general procedure was followed using (*E*)-2-styrylpyridine (90.5 mg, 0.50 mmol) and 1-chloro-4-iodobenzene (178 mg, 0.75 mmol). After 24 h, purification by column chromatography (*n*-hexane/EtOAc 9:1) yielded **3m** (96 mg, 57%) as a pale-yellow solid.

M.p.: 139 °C;

^{1}H NMR (400 MHz, $CDCl_3$) δ = 8.53 (ddd, *J* = 4.8, 1.8, 0.9 Hz, 1H), 7.38 – 7.26 (m, 8H), 7.14 (d, *J* = 8.7 Hz, 3H), 7.01 (ddd, *J* = 7.6, 4.8, 1.1 Hz, 1H), 6.76 (d, *J* = 8.0 Hz, 1H) ppm;

^{13}C NMR (101 MHz, $CDCl_3$) δ = 156.3 (C_q), 149.5 (CH), 144.6 (C_q), 142.2 (C_q), 138.4 (C_q), 135.6 (CH), 133.8 (C_q), 131.7 (CH), 129.1 (CH), 129.0 (CH), 128.4 (CH), 128.3 (CH), 127.8 (CH), 123.9 (CH), 121.5 (CH) ppm;

IR (ATR): $\tilde{\nu}$ = 3052, 1579, 1487, 1431, 1089, 1016, 821, 754, 695, 578, 482 cm^{-1};

HR-MS (ESI): *m*/*z* calcd. for $C_{19}H_{15}ClN^+$ $[M+H]^+$ 292.0888, found 292.0876.

(*Z*)-2-(2-(4-Bromophenyl)-2-phenylvinyl)pyridine (3n)

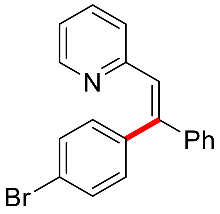

The general procedure was followed using (*E*)-2-styrylpyridine (90.5 mg, 0.50 mmol) and 1-bromo-4-iodobenzene (213 mg, 0.75 mmol). After 24 h, purification by column chromatography (*n*-hexane/EtOAc 9:1) yielded **3n** (96 mg, 57%) as a pale-yellow solid.

M.p.: 128 °C;

^{1}H NMR (400 MHz, $CDCl_3$) δ = 8.53 (ddd, *J* = 4.8, 1.8, 0.9 Hz, 1H), 7.51 – 7.42 (m, 2H), 7.39 – 7.29 (m, 6H), 7.15 (s, 1H), 7.12 – 7.04 (m, 2H), 7.02 (ddd, *J* = 7.6, 4.9, 1.1 Hz, 1H), 6.76 (dt, *J* = 8.1, 1.1 Hz, 1H) ppm;

^{13}C NMR (101 MHz, $CDCl_3$) δ = 156.3 (C_q), 149.5 (CH), 144.7 (C_q), 142.2 (C_q), 139.0 (C_q), 135.6 (CH), 132.1 (CH), 132.0 (CH), 129.1 (CH), 128.5 (CH), 128.4 (CH), 127.9 (CH), 123.9 (CH), 122.0 (C_q), 121.5 (CH) ppm;

IR (ATR): $\tilde{\nu}$ = 3052, 1586, 1489, 1435, 1260, 1070, 1010, 820, 742, 696, 575, 490 cm^{-1};

HR-MS (ESI): *m*/*z* calcd. for $C_{19}H_{15}BrN^+$ $[M+H]^+$ 336.0382, found 336.0375.

(*Z*)-2-(2-(3-Methoxyphenyl)-2-phenylvinyl)pyridine (3o)

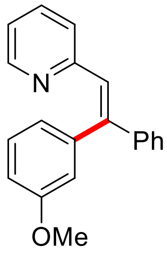

The general procedure was followed using (*E*)-2-styrylpyridine (90.5 mg, 0.50 mmol) and 1-iodo-3-methoxybenzene (175 mg, 0.75 mmol). After 24 h, purification by column chromatography (*n*-hexane/EtOAc 9:1) yielded **3o** (95 mg, 66%) as a brown sticky oil.

Note: The crude product was obtained as a mixture of (*Z*)- and (*E*)-isomer. Analysis by ^{1}H NMR spectroscopy showed a ratio *Z*/*E* = 89:11.

^{1}H NMR (400 MHz, $CDCl_3$) δ = 8.64 – 8.31 (m, 1H), 7.41 – 7.37 (m, 2H), 7.35 – 7.24 (m, 5H), 7.17 (s, 1H), 7.02 – 6.96 (m, 1H), 6.90 (dd, J = 8.3, 2.6 Hz, 1H), 6.83 – 6.78 (m, 1H), 6.77 – 6.71 (m, 2H), 3.71 (d, *J* = 0.8 Hz, 3H) ppm;

^{13}C NMR (101 MHz, $CDCl_3$) δ = 160.0 (C_q), 156.6 (C_q), 149.3, 145.7 (C_q), 142.3 (C_q), 141.3 (C_q), 135.4 (CH), 129.9 (CH), 128.7 (CH), 128.3 (CH), 128.1 (CH), 127.8 (CH), 123.8 (CH), 122.6 (CH), 121.3 (CH), 115.2 (CH), 113.8 (CH), 55.3 (CH_3) ppm;

IR (ATR): $\tilde{\nu}$ = 3054, 1572, 1456, 1423, 1229, 877, 768, 693, 555, 457 cm^{-1};

HR-MS (ESI): *m*/*z* calcd. for $C_{20}H_{18}NO^+$ $[M+H]^+$ 288.1383, found 288.1387.

The spectral data are in accordance with those reported in the literature.[2]

(*Z*)-2-(2-Phenyl-2-(*m*-tolyl)vinyl)pyridine (3p)

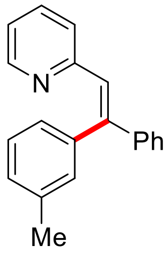

The general procedure was followed using (*E*)-2-styrylpyridine (90.5 mg, 0.50 mmol) and 1-iodo-3-methylbenzene (164 mg, 0.75 mmol). After 24 h, purification by column chromatography (*n*-hexane/EtOAc 9:1) yielded **3p** (121 mg, 89%) as a colorless solid.

M.p.: 98 °C;

^{1}H NMR (400 MHz, $CDCl_3$) δ = 8.55 (ddd, *J* = 4.9, 1.9, 1.0 Hz, 1H), 7.43 – 7.38 (m, 2H), 7.37 – 7.21 (m, 5H), 7.19 – 7.14 (m, 2H), 7.03 (dd, *J* = 8.8, 2.0 Hz, 2H), 6.98 (ddd, *J* = 7.5, 4.8, 1.1 Hz, 1H), 6.72 (d, *J* = 8.1 Hz, 1H), 2.31 (s, 3H) ppm;

^{13}C NMR (101 MHz, $CDCl_3$) δ = 156.7 (C_q), 149.3 (CH), 146.0 (C_q), 142.6 (C_q), 139.9 (C_q), 138.5 (C_q), 135.3 (CH), 130.6 (CH), 128.7 (CH), 128.6 (CH), 128.5 (CH), 128.3 (CH), 128.0 (CH), 127.8 (CH), 127.2 (CH), 123.7 (CH), 121.2 (CH), 21.5 (CH_3) ppm;

IR (ATR): $\tilde{\nu}$ = 3051, 1586, 1435, 1261, 889, 766, 694, 592, 520, 405 cm^{-1};

HR-MS (ESI): *m*/*z* calcd. for $C_{20}H_{17}NNa^+$ $[M+Na]^+$ 294.1253, found 294.1258.

(*Z*)-3-(1-Phenyl-2-(pyridin-2-yl)vinyl)benzonitrile (3q)

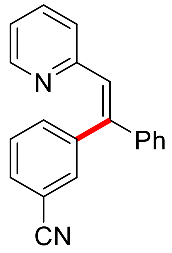

The general procedure was followed using (*E*)-2-styrylpyridine (90.5 mg, 0.50 mmol) and 3-iodobenzonitrile (173 mg, 0.75 mmol). After 24 h, purification by column chromatography (*n*-hexane/EtOAc 4:1) yielded **3q** (68 mg, 48%) as pale yellow solid.

M.p.: 121 °C;

^{1}H NMR (300 MHz, $CDCl_3$) δ = 8.52 – 8.43 (m, 1H), 7.62 (ddd, *J* = 5.2, 3.6, 1.6 Hz, 1H), 7.51 (q, *J* = 1.3 Hz, 1H), 7.47 – 7.25 (m, 8H), 7.18 (s, 1H), 7.05 (ddd, *J* = 7.5, 4.9, 1.3 Hz, 1H), 6.76 (d, *J* = 8.0 Hz, 1H) ppm;

^{13}C NMR (101 MHz, $CDCl_3$) δ = 155.7 (C_q), 149.6 (CH), 143.7 (C_q), 141.7 (C_q), 141.6 (C_q), 135.9 (CH), 135.0 (CH), 133.9 (CH), 131.4 (CH), 129.8 (CH), 129.5 (CH), 128.7 (CH), 127.9 (CH), 124.2 (CH), 121.9 (CH), 118.7 (C_q), 112.9 (C_q) ppm;

IR (ATR): $\tilde{\nu}$ = 3056, 2229, 1586, 1468, 1266, 769, 699, 556, 517, 405 cm^{-1};

HR-MS (ESI): *m*/*z* calcd. for $C_{20}H_{15}N_2^+$ $[M+H]^+$ 283.1230, found 283.1234.

(*Z*)-2-(2-(3-Chlorophenyl)-2-phenylvinyl)pyridine (3r)

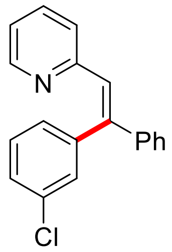

The general procedure was followed using (*E*)-2-styrylpyridine (90.5 mg, 0.50 mmol) and 1-chloro-3-iodobenzene (179 mg, 0.75 mmol). After 24 h, purification by column chromatography (*n*-hexane/EtOAc 9:1) yielded **3r** (85 mg, 59%) as a pale-yellow oil.

^{1}H NMR (400 MHz, $CDCl_3$) δ = 8.51 (ddd, *J* = 4.9, 1.9, 1.0 Hz, 1H), 7.35 – 7.28 (m, 7H), 7.25 – 7.21 (m, 1H), 7.19 (t, *J* = 1.8 Hz, 1H), 7.14 (s, 1H), 7.07 (dt, *J* = 7.5, 1.4 Hz, 1H), 6.99 (ddd, *J* = 7.5, 4.9, 1.1 Hz, 1H), 6.71 (dt, *J* = 8.1, 1.1 Hz, 1H) ppm;

^{13}C NMR (101 MHz, $CDCl_3$) δ = 156.2 (C_q), 149.5 (CH), 144.4 (C_q), 142.0 (C_q), 141.9 (C_q), 135.6 (CH), 134.7 (C_q), 130.2 (CH), 130.1 (CH), 129.4 (CH), 128.6 (CH), 128.5 (CH), 128.4 (CH), 128.1 (CH), 127.8 (CH), 123.9 (CH), 121.6 (CH) ppm;

IR (ATR): $\tilde{\nu}$ = 3054, 1584, 1465, 1433, 1152, 1083, 765, 700, 521, 405 cm^{-1};

HR-MS (ESI): *m*/*z* calcd. for $C_{19}H_{14}ClNNa^+$ $[M+Na]^+$ 314.0707, found 314.0708.

(*Z*)-2-(2-(3,5-Dimethylphenyl)-2-phenylvinyl)pyridine (3s)

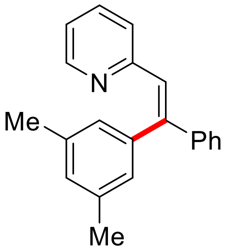

The general procedure was followed using (*E*)-2-styrylpyridine (90.5 mg, 0.50 mmol) and 1-iodo-3,5-dimethylbenzene (174 mg, 0.75 mmol). After 24 h, purification by column chromatography (*n*-hexane/EtOAc 9:1) yielded **3s** (124 mg, 87%) as a white solid.

M.p.: 133 °C;

^{1}H NMR (400 MHz, $CDCl_3$) δ = 8.73 – 8.27 (m, 1H), 7.42 – 7.36 (m, 2H), 7.35 – 7.27 (m, 4H), 7.15 (s, 1H), 7.03 – 6.95 (m, 2H), 6.87 – 6.82 (m, 2H), 6.73 (dt, *J* = 8.2, 1.1 Hz, 1H), 2.27 (s, 6H) ppm;

^{13}C NMR (101 MHz, $CDCl_3$) δ = 156.8 (C_q), 149.3 (CH), 146.2 (C_q), 142.7 (C_q), 139.8 (C_q), 138.4 (C_q), 135.3 (CH), 129.5 (CH), 128.5 (CH), 128.3 (CH), 128.0 (CH), 127.8 (CH), 127.7 (CH), 123.7 (CH), 121.2 (CH), 21.4 (CH_3) ppm;

IR (ATR): $\tilde{\nu}$ = 3025, 1583, 1462, 771, 742, 696, 571, 524, 404 cm^{-1};

HR-MS (ESI): *m*/*z* calcd. for $C_{21}H_{20}N^+$ $[M+H]^+$ 286.1590, found 286.1594.

(*Z*)-2-(2-(3,4-Dimethoxyphenyl)-2-phenylvinyl)pyridine (3t)

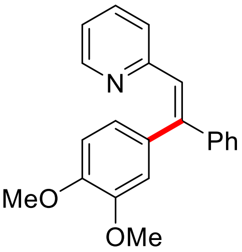

The general procedure was followed using (*E*)-2-styrylpyridine (90.5 mg, 0.50 mmol) and 4-iodo-1,2-dimethoxybenzene (198 mg, 0.75 mmol). After 24 h, purification by column chromatography (*n*-hexane/EtOAc 4:1) yielded **3t** (95 mg, 60%) as a brown sticky oil.

Note: The crude product was obtained as a mixture of (*Z*)- and (*E*)-isomer. Analysis by ^{1}H NMR spectroscopy showed a ratio *Z*/*E* = 86:14.

^{1}H NMR (400 MHz, $CDCl_3$) δ = 8.53 (ddd, *J* = 5.0, 1.8, 1.0 Hz, 1H), 7.41 – 7.36 (m, 2H), 7.35 – 7.28 (m, 4H), 7.11 (s, 1H), 6.98 (ddd, *J* = 7.5, 4.8, 1.1 Hz, 1H), 6.84 (d, *J* = 8.2 Hz, 1H), 6.78 – 6.73 (m, 2H), 6.69 (d, *J* = 1.9 Hz, 1H), 3.90 (s, 3H), 3.69 (s, 3H) ppm;

^{13}C NMR (101 MHz, $CDCl_3$) δ = 156.9 (C_q), 149.3 (CH), 149.1 (C_q), 148.7 (C_q), 145.7 (C_q), 142.7 (C_q), 135.4 (CH), 132.3 (C_q), 128.4 (CH), 128.3 (CH), 128.2 (CH), 127.9 (CH), 123.8 (CH), 122.8 (CH), 121.2 (CH), 113.3 (CH), 111.3 (CH), 55.9 (CH_3), 55.9 (CH_3) ppm;

IR (ATR): $\tilde{\nu}$ = 3000, 1581, 1512, 1465, 1254, 1135, 1028, 769, 698, 518, 407 cm^{-1};

HR-MS (ESI): *m*/*z* calcd. for $C_{21}H_{20}NO_2^+$ $[M+H]^+$ 318.1489, found 318.1476.

(*Z*)-2-(2-(Benzo[*d*][1,3]dioxol-5-yl)-2-phenylvinyl)pyridine (3u)

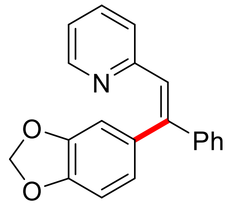

The general procedure was followed using (*E*)-2-styrylpyridine (90.5 mg, 0.50 mmol) and 5-iodobenzo[*d*][1,3]dioxole (186 mg, 0.75 mmol). After 24 h, purification by column chromatography (*n*-hexane/EtOAc 8:1) yielded **3u** (77 mg, 51%) as a pale-yellow oil.

^{1}H NMR (400 MHz, $CDCl_3$) δ = 8.64 (ddd, *J* = 4.9, 1.9, 1.0 Hz, 1H), 7.50 – 7.38 (m, 6H), 7.20 (s, 1H), 7.15 – 7.08 (m, 1H), 6.94 (dt, *J* = 8.0, 1.1 Hz, 1H), 6.88 (dd, *J* = 7.7, 0.6 Hz, 1H), 6.82 – 6.76 (m, 2H), 6.09 (s, 2H) ppm;

^{13}C NMR (101 MHz, $CDCl_3$) δ = 156.7 (C_q), 149.3 (CH), 148.1 (C_q), 147.4 (C_q), 145.6 (C_q), 142.7 (C_q), 135.6 (CH), 133.6 (C_q), 128.6 (CH), 128.34 (CH), 128.2 (CH), 127.9 (CH), 123.9 (CH), 123.9 (CH), 121.3 (CH), 110.6 (CH), 108.8 (CH), 101.2 (CH_2) ppm;

IR (ATR): $\tilde{\nu}$ = 3025, 1583, 1462, 771, 742, 696, 571, 524, 404 cm^{-1};

HR-MS (ESI): *m*/*z* calcd. for $C_{20}H_{16}NO_2^+$ $[M+H]^+$ 302.1176, found 302.1179.

(*Z*)-2-(2-Phenyl-2-(thiophen-3-yl)vinyl)pyridine (3v)

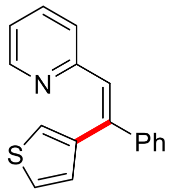

The general procedure was followed using (*E*)-2-styrylpyridine (90.5 mg, 0.50 mmol) and 3-iodothiophene (158 mg, 0.75 mmol). After 24 h, purification by column chromatography (*n*-hexane/EtOAc 9:1) yielded **3v** (62 mg, 42%) as a pale-brown solid.

M.p.: 91 °C;

^{1}H NMR (400 MHz, $CDCl_3$) δ = 8.56 (ddd, *J* = 4.9, 1.9, 1.0 Hz, 1H), 7.40 (ddd, *J* = 8.2, 4.9, 2.0 Hz, 3H), 7.36 – 7.30 (m, 4H), 7.13 (s, 1H), 7.09 (dd, *J* = 3.0, 1.3 Hz, 1H), 7.03 (ddd, *J* = 7.5, 4.9, 1.1 Hz, 1H), 6.89 (dd, *J* = 4.9, 1.2 Hz, 1H), 6.83 (dt, *J* = 8.1, 1.1 Hz, 1H) ppm;

^{13}C NMR (101 MHz, $CDCl_3$) δ = 156.7 (C_q), 149.3 (CH), 142.3 (C_q), 140.6 (C_q), 139.9 (C_q), 135.6 (CH), 129.3 (CH), 129.2 (CH), 128.4 (CH), 128.4 (CH), 128.3 (CH), 127.7 (CH), 125.9 (CH), 125.2 (CH), 123.6 (CH), 121.4 (CH) ppm;

IR (ATR): $\tilde{\nu}$ = 3054, 1608, 1585, 1466, 1436, 893, 766, 692, 516, 433 cm^{-1};

HR-MS (ESI): *m*/*z* calcd. for $C_{17}H_{13}NSNa^+$ $[M+Na]^+$ 286.0661, found 286.0670.

The spectral data are in accordance with those reported in the literature.[2]

(1*R*,2*S*,5*R*)-2-Isopropyl-5-methylcyclohexyl 4-((*Z*)-1-phenyl-2-(pyridin-2-yl)vinyl) benzoate (3w)

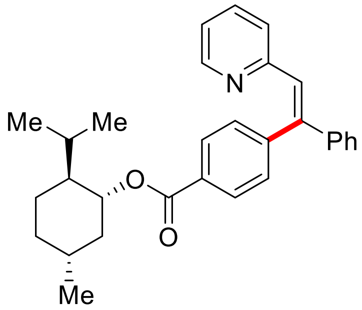

The general procedure was followed using (*E*)-2-styrylpyridine (90.5 mg, 0.50 mmol) and (1*R*,2*S*,5*R*)-2-isopropyl-5-methylcyclohexyl 4-iodobenzoate (290 mg, 0.75 mmol). After 24 h, purification by column chromatography (*n*-hexane/EtOAc 7:1) yielded **3w** (82 mg, 37%) as a pale-yellow sticky liquid.

^{1}H NMR (400 MHz, $CDCl_3$) δ = 8.53 (ddd, *J* = 4.9, 1.9, 1.0 Hz, 1H), 8.12 – 7.89 (m, 2H), 7.38 – 7.29 (m, 6H), 7.33 – 7.25 (m, 2H), 7.19 (s, 1H), 7.01 (ddd, *J* = 7.5, 4.9, 1.2 Hz, 1H), 6.74 (dt, *J* = 8.1, 1.1 Hz, 1H), 4.96 (td, *J* = 10.8, 4.4 Hz, 1H), 2.22 – 2.10 (m, 1H), 2.06 – 1.91 (m, 1H), 1.84 – 1.67 (m, 2H), 1.57 (dddd, *J* = 12.4, 10.9, 6.2, 3.0 Hz, 2H), 1.26 (d, *J* = 2.9 Hz, 1H), 1.19 – 1.05 (m, 2H), 0.94 (d, *J* = 1.1 Hz, 3H), 0.93 (d, *J* = 1.6 Hz, 3H), 0.82 (d, *J* = 6.9 Hz, 3H) ppm;

^{13}C NMR (101 MHz, $CDCl_3$) δ = 166.0 (C_q), 156.2 (C_q), 149.5 (CH), 145.0 (C_q), 144.8 (C_q), 142.1 (C_q), 135.7 (CH), 130.4 (CH), 130.2 (C_q), 130.0 (CH), 129.3 (CH), 128.5 (CH), 128.4 (CH), 127.9 (CH), 124.0 (CH), 121.6 (CH), 75.1 (CH), 47.3 (CH), 41.1 (CH_2), 34.4 (CH_2), 31.5 (CH), 26.5 (CH), 23.7 (CH_2), 22.2 (CH_3), 20.9 (CH_3), 16.6 (CH_3) ppm;

IR (ATR): $\tilde{\nu}$ = 2954, 2925, 1708, 1584, 1457, 1264, 1104, 736, 700, 575, 469 cm^{-1};

HR-MS (ESI): *m*/*z* calcd. for $C_{30}H_{34}NO_2^+$ $[M+H]^+$ 440.2584, found 440.2587.

((1*R*,5*S*)-6,6-Dimethylbicyclo[3.1.1]hept-2-en-3-yl)methyl 4-((*Z*)-1-phenyl-2-(pyridin-2-yl)vinyl)benzoate (3x)

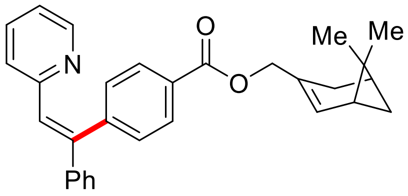

The general procedure was followed using (*E*)-2-styrylpyridine (90.5 mg, 0.50 mmol) and ((1*R*,5*S*)-6,6-dimethylbicyclo[3.1.1]hept-2-en-2-yl)methyl 4-iodobenzoate (276 mg, 0.75 mmol). After 24 h, purification by column chromatography (*n*-hexane/EtOAc 8:1) yielded **3x** (75 mg, 34%) as a pale-yellow sticky liquid.

^{1}H NMR (400 MHz, $CDCl_3$) δ = 8.52 (q, *J* = 1.7 Hz, 1H), 8.07 – 7.94 (m, 2H), 7.47 – 7.24 (m, 8H), 7.19 (s, 1H), 7.01 (ddd, *J* = 7.5, 4.9, 1.2 Hz, 1H), 6.71 (dt, *J* = 8.2, 1.2 Hz, 1H), 5.82 – 5.45 (m, 1H), 4.71 (d, *J* = 1.5 Hz, 2H), 2.50 – 2.09 (m, 5H), 1.31 (s, 3H), 1.24 (d, *J* = 8.7 Hz, 1H), 0.88 (s, 3H) ppm;

^{13}C NMR (101 MHz, $CDCl_3$) δ = 166.4 (C_q), 156.2 (C_q), 149.5 (CH), 145.1 (C_q), 143.1 (C_q), 142.0 (C_q), 135.7 (CH), 130.4 (CH), 130.1 (C_q), 129.4 (CH), 128.5 (CH), 128.4 (CH), 127.9

(CH), 124.0 (CH), 121.9 (CH), 121.6 (CH), 67.8 (CH_2), 67.7 (C_q), 43.9 (CH), 43.8 (CH), 40.9 (CH), 38.3 (C_q), 31.7 (CH_2), 31.5 (CH_2), 26.3 (CH_3), 21.3 (CH_3) ppm;

IR (ATR): $\tilde{\nu}$ = 2919, 1761, 1583, 1435, 1266, 1092, 770, 702, 576, 403 cm^{-1};

HR-MS (ESI): *m*/*z* calcd. for $C_{30}H_{30}NO_2^+$ $[M+H]^+$ 436.2271, found 436.2273.

(*Z*)-4-(1-Phenyl-2-(pyridin-2-yl)vinyl)phenyl (*S*)-2-(6-methoxynaphthalen-2-yl)propanoate (3y)

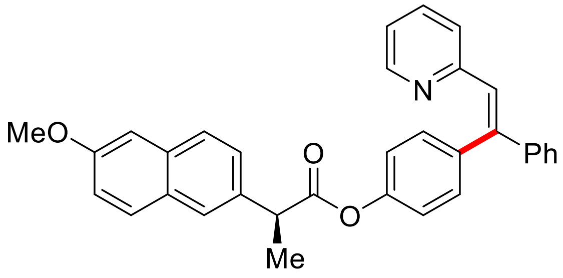

The general procedure was followed using (*E*)-2-styrylpyridine (90.5 mg, 0.50 mmol) and 4-iodophenyl (*S*)-2-(6-methoxynaphthalen-2-yl)propanoate (324 mg, 0.75 mmol). After 24 h, purification by column chromatography (*n*-hexane/EtOAc 4:1) yielded **3y** (133 mg, 55%) as a brown sticky oil.

^{1}H NMR (400 MHz, $CDCl_3$) δ = 8.58 – 8.43 (m, 1H), 7.86 – 7.72 (m, 3H), 7.54 (dd, *J* = 8.5, 1.9 Hz, 1H), 7.41 – 7.29 (m, 6H), 7.18 (td, *J* = 9.0, 2.6 Hz, 5H), 7.06 – 6.91 (m, 3H), 6.72 (d, *J* = 8.1 Hz, 1H), 4.14 (q, *J* = 7.2 Hz, 1H), 3.92 (s, 3H), 1.73 (d, *J* = 7.1 Hz, 3H) ppm;

^{13}C NMR (101 MHz, $CDCl_3$) δ = 173.1 (C_q), 157.8 (C_q), 156.4 (C_q), 150.5 (C_q), 149.3 (CH), 144.9 (C_q), 142.3 (C_q), 137.4 (C_q), 135.6 (CH), 135.1 (C_q), 133.9 (C_q), 131.2 (CH), 129.3 (CH), 129.0 (C_q), 128.9 (CH), 128.3 (CH), 128.2 (CH), 127.8 (CH), 127.5 (CH), 126.2 (CH), 126.1 (CH), 123.8 (CH), 121.8 (CH), 121.3 (CH), 119.2 (CH), 105.6 (CH), 55.3 (CH_3), 45.6 (CH), 18.5 (CH_3) ppm;

IR (ATR): $\tilde{\nu}$ = 2935, 1753, 1604, 1499, 1456, 1265, 1199, 1166, 1126, 1025, 695, 593 cm^{-1};

HR-MS (ESI): *m*/*z* calcd. for $C_{33}H_{28}NO_3^+$ $[M+H]^+$ 486.2064, found 486.2065.

(*Z*)-4-(1-Phenyl-2-(pyridin-2-yl)vinyl)phenyl 2-(1-(4-chlorobenzoyl)-5-methoxy-2-methyl-1H-indol-3-yl)acetate (3z)

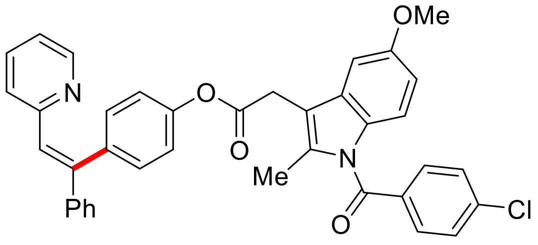

The general procedure was followed using (*E*)-2-styrylpyridine (90.5 mg, 0.50 mmol) and 4-iodophenyl 2-(1-(4-chlorobenzoyl)-5-methoxy-2-methyl-1*H*-indol-3-yl)acetate (409 mg, 0.75 mmol). After 24 h, purification by column chromatography (*n*-hexane/EtOAc 3:1) yielded **3z** (180 mg, 59%) as a pale-yellow sticky liquid.

^{1}H NMR (400 MHz, $CDCl_3$) δ = 8.53 (dt, *J* = 4.8, 1.4 Hz, 1H), 7.73 – 7.65 (m, 2H), 7.51 – 7.45 (m, 2H), 7.38 – 7.29 (m, 6H), 7.22 – 7.18 (m, 2H), 7.16 (s, 1H), 7.11 – 7.05 (m, 3H), 7.01 (ddd, *J* = 7.5, 4.9, 1.2 Hz, 1H), 6.89 (d, *J* = 9.0 Hz, 1H), 6.78 – 6.68 (m, 2H), 3.93 (s, 2H), 3.84 (s, 3H), 2.48 (s, 3H) ppm;

^{13}C NMR (101 MHz, $CDCl_3$) δ = 169.3 (C_q), 168.4 (C_q), 156.4 (C_q), 156.2 (C_q), 150.4 (C_q), 149.2 (CH), 145.1 (C_q), 142.3 (C_q), 139.5 (C_q), 137.6 (C_q), 136.4 (C_q), 135.8 (CH), 133.9 (C_q), 131.4 (CH), 131.3 (CH), 130.9 (C_q), 130.6 (C_q), 129.3 (CH), 128.8 (CH), 128.4 (CH), 128.3 (CH), 127.9 (CH), 124.0 (CH), 121.8 (CH), 121.5 (CH), 115.1 (CH), 112.0 (C_q), 111.8 (CH), 101.4 (CH), 55.9 (CH_3), 30.7 (CH_2), 13.5 (CH_3) ppm;

IR (ATR): $\tilde{\nu}$ = 3056, 2929, 1756, 1683, 1588, 1470, 1360, 1158, 1063, 832, 730, 588 cm^{-1};

HR-MS (ESI): *m*/*z* calcd. for $C_{38}H_{30}N_2O_4Cl^+$ $[M+H]^+$ 613.1889, found 613.1886.

(*E*)-2-(2-(4-Methoxyphenyl)-2-(*p*-tolyl)vinyl)pyridine (3aa)

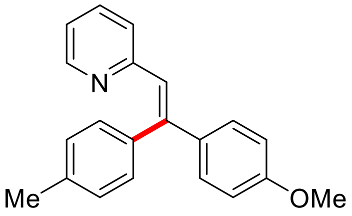

The general procedure was followed using (*E*)-2-(4-methoxystyryl)pyridine (106 mg, 0.50 mmol) and 1-iodo-4-methylbenzene (164 mg, 0.75 mmol). After 24 h, purification by column chromatography (*n*-hexane/EtOAc 8:1) yielded **3aa** (86 mg, 57%) as a pale-yellow liquid.

^{1}H NMR (400 MHz, $CDCl_3$) δ = 8.52 (ddd, *J* = 4.9, 1.9, 1.0 Hz, 1H), 7.38 – 7.23 (m, 3H), 7.22 – 7.12 (m, 2H), 7.10 – 7.05 (m, 3H), 6.95 (ddd, *J* = 7.5, 4.9, 1.1 Hz, 1H), 6.89 – 6.79 (m, 2H), 6.69 (dt, *J* = 8.0, 1.1 Hz, 1H), 3.80 (s, 3H), 2.39 (s, 3H) ppm;

^{13}C NMR (101 MHz, $CDCl_3$) δ = 159.7 (C_q), 157.1 (C_q), 149.2 (CH), 145.5 (C_q), 137.6 (C_q), 137.1 (C_q), 135.4 (C_q), 135.3 (CH), 130.1 (CH), 129.5 (CH), 129.1 (CH), 126.8 (CH), 123.6 (CH), 120.9 (CH), 113.7 (CH), 55.4 (CH_3), 21.4 (CH_3) ppm;

IR (ATR): $\tilde{\nu}$ = 2999, 1573, 1530, 1460, 1429, 1245, 1178, 1030, 824, 736 cm^{-1};

HR-MS (ESI): *m*/*z* calcd. for $C_{21}H_{20}NO_2^+$ $[M+H]^+$ 302.1539, found 302.1542.

(*E*)-4-(2-(Pyridin-2-yl)-1-(*p*-tolyl)vinyl)benzonitrile (3ab)

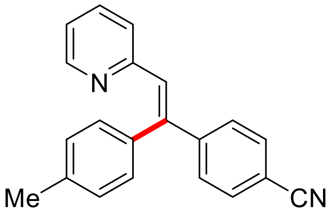

The general procedure was followed using (*E*)-4-(2-(pyridin-2-yl)vinyl)benzonitrile (103 mg, 0.50 mmol) and 1-iodo-4-methylbenzene (164 mg, 0.75 mmol). After 24 h, purification by column chromatography (*n*-hexane/EtOAc 4:1) yielded **3ab** (47 mg, 32%) as a colorless sticky oil.

^{1}**H NMR** (400 MHz, $CDCl_3$) δ 8.49 (ddd, J = 4.9, 1.8, 0.9 Hz, 1H), 7.61 (d, J = 8.3 Hz, 2H), 7.39 (td, J = 7.7, 1.8 Hz, 1H), 7.31 (d, J = 8.3 Hz, 2H), 7.21 – 7.11 (m, 5H), 7.03 (ddd, J = 7.5, 4.9, 1.1 Hz, 1H), 6.73 (dd, J = 8.1, 1.1 Hz, 1H) ppm;

^{13}**C NMR** (101 MHz, $CDCl_3$) δ = 155.9 (C_q), 149.6 (CH), 145.5 (C_q), 144.1 (C_q), 138.8 (C_q), 138.7 (C_q), 135.8 (CH), 132.4 (CH), 131.2 (CH), 129.4 (CH), 128.9 (CH), 127.8 (CH), 124.1 (CH), 121.7 (CH), 118.9 (Cq), 111.5 (C_q), 21.31 (CH_3) ppm;

IR (ATR): $\tilde{\nu}$ = 2921, 2226, 1605, 1507, 1462, 1431, 811, 771, 740, 572, 523, 404 cm^{-1};

HR-MS (ESI): m/z calcd. for $C_{21}H_{17}N_2^+$ $[M+H]^+$ 297.1386, found 297.1387.

(*Z*)-2-(2-(4-Ethylphenyl)-2-(4-methoxyphenyl)vinyl)pyridine (3ac)

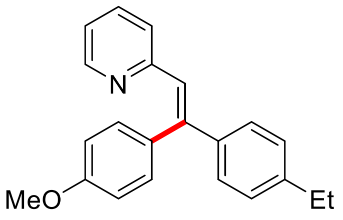

The general procedure was followed using (*E*)-2-(4-ethylstyryl)pyridine (105 mg, 0.50 mmol) and 1-iodo-4-methoxybenzene (175 mg, 0.75 mmol). After 24 h, purification by column chromatography (*n*-hexane/EtOAc 9:1) yielded **3ac** (91 mg, 58%) as a pale-yellow sticky oil.

^{1}**H NMR** (400 MHz, $CDCl_3$) δ = 8.53 (ddd, J = 5.0, 1.8, 1.0 Hz, 1H), 7.35 – 7.27 (m, 3H), 7.18 – 7.09 (m, 5H), 6.99 – 6.93 (m, 1H), 6.91 – 6.85 (m, 2H), 6.74 (dt, J = 8.0, 1.1 Hz, 1H), 3.83 (s, 3H), 2.66 (q, J = 7.6 Hz, 2H), 1.25 (t, J = 7.6 Hz, 3H) ppm;

^{13}**C NMR** (101 MHz, $CDCl_3$) δ = 159.3 (C_q), 157.1 (C_q), 149.3 (CH), 145.5 (C_q), 144.3 (C_q), 140.3 (C_q), 135.3 (CH), 132.3 (C_q), 131.5 (CH), 127.9 (CH), 127.8 (CH), 127.6 (CH), 123.7 (CH), 120.9 (CH), 114.1 (CH), 55.3 (CH_3), 28.6 (CH_2), 15.5 (CH_3) ppm;

IR (ATR): $\tilde{\nu}$ = 2929, 1607, 1580, 1508, 1461, 1244, 1031, 830, 770, 402 cm^{-1};

HR-MS (ESI): m/z calcd. for $C_{22}H_{21}NONa^+$ $[M+Na]^+$ 338.1515, found 338.1520.

(*Z*)-2-(2-(4-(*tert*-Butyl)phenyl)-2-(4-methoxyphenyl)vinyl)pyridine (3ad)

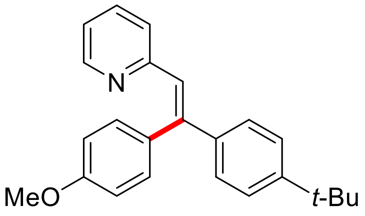

The general procedure was followed using (*E*)-2-(4-(*tert*-butyl)styryl)pyridine (119 mg, 0.50 mmol) and 1-iodo-4-methoxybenzene (175 mg, 0.75 mmol). After 24 h, purification by column chromatography (*n*-hexane/EtOAc 8:1) yielded **3ad** (70 mg, 41%) as a pale-yellow sticky oil.

^{1}**H NMR** (400 MHz, $CDCl_3$) δ = 8.54 (d, J = 1.1 Hz, 1H), 7.40 – 7.27 (m, 5H), 7.13 (dd, J = 6.5, 2.1 Hz, 3H), 6.97 (ddd, J = 7.5, 4.9, 1.2 Hz, 1H), 6.92 – 6.85 (m, 2H), 6.73 (dt, J = 8.1, 1.1 Hz, 1H), 3.84 (s, 3H), 1.33 (s, 9H) ppm;

^{13}**C NMR** (101 MHz, $CDCl_3$) δ = 159.3 (C_q), 157.1 (C_q), 151.3 (C_q), 149.3 (CH), 145.4 (C_q), 139.9 (C_q), 135.4 (CH), 132.3 (C_q), 131.5 (CH), 127.7 (CH), 127.6 (CH), 125.2 (CH), 123.8 (CH), 121.0 (CH), 114.2 (CH), 55.3 (CH_3), 34.7 (C_q), 31.4 (CH_3) ppm;

IR (ATR): $\tilde{\nu}$ = 2959, 1606, 1580, 1508, 1461, 1432, 1241, 1028, 830, 738, 577, 405 cm^{-1};

HR-MS (ESI): *m*/*z* calcd. for $C_{24}H_{26}NO^+$ $[M+H]^+$ 344.2009, found 344.2011.

(*E*)-2-(2-(4-Chlorophenyl)-2-(4-methoxyphenyl)vinyl)pyridine (3ae)

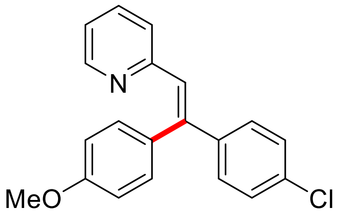

The general procedure was followed using (*E*)-2-(4-chlorostyryl)pyridine (108 mg, 0.50 mmol) and 1-iodo-4-methoxybenzene (175 mg, 0.75 mmol). After 24 h, purification by column chromatography (*n*-hexane/EtOAc 8:1) yielded **3ae** (102 mg, 64%) as a pale-yellow sticky oil.

^{1}**H NMR** (400 MHz, $CDCl_3$) δ = 8.52 (ddd, *J* = 4.8, 1.9, 1.0 Hz, 1H), 7.38 – 7.23 (m, 5H), 7.13 – 7.05 (m, 3H), 6.98 (ddd, *J* = 7.5, 4.9, 1.1 Hz, 1H), 6.90 – 6.83 (m, 2H), 6.75 (dt, *J* = 8.2, 1.1 Hz, 1H), 3.82 (s, 3H) ppm;

^{13}**C NMR** (101 MHz, $CDCl_3$) δ = 159.5 (C_q), 156.7 (C_q), 149.4 (CH), 144.4 (C_q), 141.5 (C_q), 135.4 (CH), 134.0 (C_q), 131.6 (C_q), 131.4 (CH), 129.2 (CH), 128.7 (CH), 128.5 (CH), 123.8 (CH), 121.3 (CH), 114.3 (CH), 55.3 (CH_3) ppm;

IR (ATR): $\tilde{\nu}$ = 2955, 1606, 1574, 1508, 1460, 1290, 1242, 1034, 771, 742, 518, 407 cm^{-1};

HR-MS (ESI): *m*/*z* calcd. for $C_{20}H_{17}NOCl^+$ $[M+H]^+$ 322.0993, found 322.0987.

(*E*)-2-(2-(4-Bromophenyl)-2-(4-methoxyphenyl)vinyl)pyridine (3af)

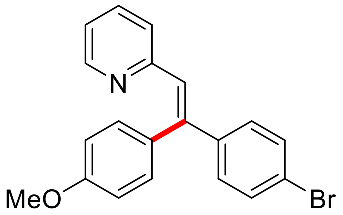

The general procedure was followed using (*E*)-2-(4-bromostyryl)pyridine (130 mg, 0.50 mmol) and 1-iodo-4-methoxybenzene (175 mg, 0.75 mmol). After 24 h, purification by column chromatography (*n*-hexane/EtOAc 8:1) yielded **3af** (95 mg, 52%) as a pale-yellow sticky oil.

^{1}**H NMR** (400 MHz, $CDCl_3$) δ = 8.52 (ddd, *J* = 4.9, 1.9, 1.0 Hz, 1H), 7.46 – 7.40 (m, 2H), 7.32 (td, *J* = 7.8, 1.9 Hz, 1H), 7.24 – 7.18 (m, 2H), 7.10 – 7.05 (m, 3H), 6.99 (ddd, *J* = 7.5, 4.9, 1.1 Hz, 1H), 6.90 – 6.84 (m, 2H), 6.75 (dt, *J* = 8.1, 1.1 Hz, 1H), 3.83 (s, 3H) ppm.

^{13}**C NMR** (101 MHz, $CDCl_3$) δ = 159.5 (C_q), 156.6 (C_q), 149.3 (CH), 144.6 (C_q), 142.0 (C_q), 135.7 (CH), 131.6 (CH), 131.5 (C_q), 131.4 (CH), 129.6 (CH), 128.7 (CH), 123.9 (CH), 122.3 (C_q), 121.4 (CH), 114.3 (CH), 55.3 (CH_3) ppm;

IR (ATR): $\tilde{\nu}$ = 2931, 1600, 1579, 1510, 1464, 1209, 1245, 1032, 838, 778, 531, 403 cm^{-1};

HR-MS (ESI): *m/z* calcd. for $C_{20}H_{16}NOBrNa^+$ $[M+Na]^+$ 388.0307, found 388.0301.

(*E*)-2-(2-(3-ethoxyphenyl)-2-(4-methoxyphenyl)vinyl)pyridine (3ag)

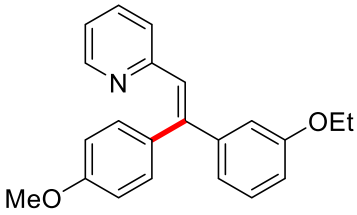

The general procedure was followed using (*E*)-2-(3-ethoxystyryl)pyridine (112 mg, 0.50 mmol) and 1-iodo-4-methoxybenzene (175 mg, 0.75 mmol). After 24 h, purification by column chromatography (*n*-hexane/EtOAc 4:1) yielded **3ag** (136 mg, 76%) as a pale-yellow sticky oil.

^{1}H NMR (400 MHz, $CDCl_3$) δ = 8.53 (ddd, *J* = 4.9, 1.9, 1.0 Hz, 1H), 7.30 (td, *J* = 7.8, 1.9 Hz, 1H), 7.21 (t, *J* = 7.9 Hz, 1H), 7.11 (d, *J* = 8.6 Hz, 3H), 7.01 – 6.93 (m, 2H), 6.91 (t, *J* = 2.1 Hz, 1H), 6.89 – 6.81 (m, 3H), 6.75 (dt, *J* = 8.1, 1.1 Hz, 1H), 3.99 (q, *J* = 7.0 Hz, 2H), 3.82 (s, 3H), 1.38 (t, *J* = 7.0 Hz, 3H) ppm;

^{13}C NMR (101 MHz, $CDCl_3$) δ = 159.3 (C_q), 158.9 (C_q), 156.9 (C_q), 149.3 (CH), 145.6 (C_q), 144.4 (C_q), 135.4 (CH), 132.0 (C_q), 131.5 (CH), 129.2 (CH), 128.5 (CH), 123.8 (CH), 121.1 (CH), 120.5 (CH), 114.3 (CH), 114.2 (CH), 114.1 (CH), 63.5 (CH_2), 55.3 (CH_3), 14.9 (CH_3) ppm;

IR (ATR): $\tilde{\nu}$ = 2978, 1599, 1572, 1508, 1432, 1244, 1173, 838, 777, 695, 533 cm^{-1};

HR-MS (ESI): *m/z* calcd. for $C_{22}H_{21}NO_2Na^+$ $[M+Na]^+$ 354.1465, found 354.1468.

(*E*)-2-(2-(3-Fluorophenyl)-2-(4-methoxyphenyl)vinyl)pyridine (3ah)

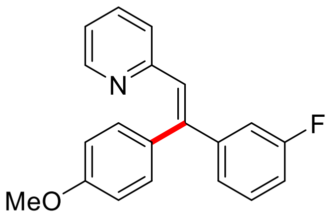

The general procedure was followed using (*E*)-2-(3-fluorostyryl)pyridine (100 mg, 0.50 mmol) and 1-iodo-4-methoxybenzene (175 mg, 0.75 mmol). After 24 h, purification by column chromatography (*n*-hexane/EtOAc 9:1) yielded **3ah** (111 mg, 73%) as a pale-yellow solid.

M.p.: 117 °C;

^{1}H NMR (400 MHz, $CDCl_3$) δ = 8.54 (ddd, *J* = 4.9, 1.8, 0.9 Hz, 1H), 7.38 – 7.24 (m, 2H), 7.17 (ddd, *J* = 7.8, 1.7, 1.0 Hz, 1H), 7.10 (dd, J = 6.6, 2.1 Hz, 3H), 7.06 – 6.95 (m, 3H), 6.92 – 6.85 (m, 2H), 6.76 (dt, *J* = 8.0, 1.1 Hz, 1H), 3.83 (s, 3H) ppm;

^{13}C NMR (101 MHz, $CDCl_3$) δ = 162.9 (C_q, d, $^1J_{C-F}$ = 245.3 Hz), 159.5 (C_q), 156.5 (C_q), 149.3 (CH), 145.3 (C_q), 144.5 (C_q), 135.6 (C_q), 131.5 (CH, d, $^3J_{C-F}$ = 2.7 Hz), 129.7 (CH), 129.6 (CH), 129.2 (CH), 123.9 (CH), 123.6 (CH), 121.4 (CH), 114.9 (CH, d, $^2J_{C-F}$ = 21.8 Hz), 114.4 (CH), 55.3 (CH_3) ppm;

^{19}F NMR (377 MHz, $CDCl_3$) δ = -113.45 ppm;

IR (ATR): $\tilde{\nu}$ = 2924, 1608, 1575, 1509, 1460, 1245, 1033, 838, 780, 694, 523, 405 cm^{-1};

HR-MS (ESI): *m*/*z* calcd. for $C_{20}H_{16}NOFNa^+$ $[M+Na]^+$ 328.1108, found 328.1114.

(*E*)-2-(2-(4-Methoxyphenyl)-2-(thiophen-2-yl)vinyl)pyridine (3ai)

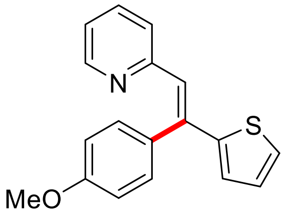

The general procedure was followed using (*E*)-2-(2-(thiophen-2-yl)vinyl)pyridine (94 mg, 0.50 mmol) and 1-iodo-4-methoxybenzene (175 mg, 0.75 mmol). After 24 h, purification by column chromatography (*n*-hexane/EtOAc 8:1) yielded **3ai** (70 mg, 48%) as a pale-yellow sticky oil.

^{1}H NMR (400 MHz, $CDCl_3$) δ = 8.52 (d, *J* = 4.8 Hz, 1H), 7.33 – 7.17 (m, 5H), 7.00 – 6.89 (m, 4H), 6.84 (dd, *J* = 3.7, 1.2 Hz, 1H), 6.64 (dd, *J* = 8.2, 1.4 Hz, 1H), 3.85 (s, 3H) ppm;

^{13}C NMR (101 MHz, $CDCl_3$) δ = 159.6 (C_q), 156.2 (C_q), 149.3 (CH), 147.7 (C_q), 139.2 (C_q), 135.4 (CH), 131.2 (C_q), 131.0 (CH), 127.6 (CH), 127.0 (CH), 126.8 (CH), 126.0 (CH), 123.6 (CH), 121.1 (CH), 114.4 (CH), 55.4 (CH_3) ppm;

IR (ATR): $\tilde{\nu}$ = 2955, 1606, 1574, 1509, 1461, 1292, 1245, 1030, 839, 701, 536, 406 cm^{-1};

HR-MS (ESI): *m*/*z* calcd. for $C_{18}H_{15}NOSNa^+$ $[M+Na]^+$ 316.0767, found 316.0763.

2-((1*Z*,3*E*)-4-Phenyl-2-(*p*-tolyl)buta-1,3-dien-1-yl)pyridine (3aj)

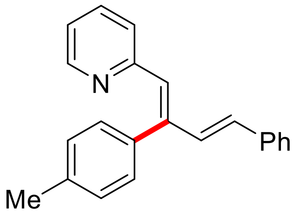

The general procedure was followed using 2-((1*E*,3*E*)-4-phenylbuta-1,3-dien-1-yl)pyridine (104 mg, 0.50 mmol) and 1-iodo-4-methylbenzene (175 mg, 0.75 mmol). After 24 h, purification by column chromatography (*n*-hexane/EtOAc 8:1) yielded **3aj** (71 mg, 48%) as a pale-yellow sticky oil.

^{1}H NMR (300 MHz, $CDCl_3$) δ = 8.60 – 8.42 (m, 1H), 7.43 – 7.30 (m, 5H), 7.25 (td, *J* = 6.3, 3.8 Hz, 3H), 7.19 – 7.10 (m, 3H), 6.96 (dd, *J* = 7.5, 5.0 Hz, 1H), 6.90 (s, 1H), 6.54 (d, *J* = 8.0 Hz, 1H), 6.25 (d, *J* = 15.9 Hz, 1H), 2.44 (s, 3H) ppm;

^{13}C NMR (75 MHz, $CDCl_3$) δ = 156.1 (C_q), 149.3 (CH), 144.8 (C_q), 137.4 (C_q), 137.2 (C_q), 135.2 (CH), 134.6 (C_q), 133.9 (CH), 133.1 (CH), 132.5 (CH), 129.7 (CH), 129.2 (CH), 128.6 (CH), 127.8 (CH), 126.7 (CH), 123.4 (CH), 121.1 (CH), 21.4. (CH_3) ppm;

IR (ATR): $\tilde{\nu}$ = 3025, 2923, 1573, 1457, 963, 820, 747, 692, 557, 488 cm^{-1};

HR-MS (ESI): *m*/*z* calcd. for $C_{22}H_{19}NNa^+$ $[M+Na]^+$ 320.1410, found 320.1418.

(*Z*)-2-(2-(4-Methoxyphenyl)prop-1-en-1-yl)pyridine (3ak)

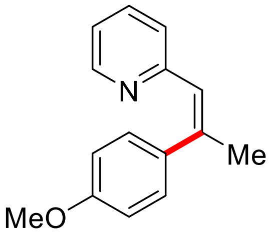

The general procedure was followed using (*E*)-2-(prop-1-en-1-yl)pyridine (59.8 mg, 0.50 mmol) 1-iodo-4-methoxybenzene (175.5 mg, 0.75 mmol). After 24 h, purification by column chromatography (*n*-hexane/EtOAc 9:1) yielded **3ak** (36.1 mg, 32%) as a pale-yellow oil.

^{1}H NMR (400 MHz, $CDCl_3$) δ = 8.49 (ddd, *J* = 4.8, 1.8, 0.9 Hz, 1H), 7.28 (td, *J* = 7.8, 1.7 Hz, 1H), 7.13 – 7.06 (m, 2H), 6.95 (ddd, *J* = 7.4, 4.9, 1.2 Hz, 1H), 6.86 – 6.78 (m, 2H), 6.71 (dt, *J* = 8.1, 1.1 Hz, 1H), 6.59 (d, *J* = 1.6 Hz, 1H), 3.80 (s, 3H), 2.22 (d, *J* = 1.5 Hz, 3H) ppm;

^{13}C NMR (101 MHz, $CDCl_3$) δ = 159.0 (C_q), 157.2 (C_q), 149.2 (CH), 142.4 (C_q), 135.3 (CH), 133.8 (C_q), 129.3 (CH), 127.4 (CH), 123.7 (CH), 120.1 (CH), 114.1 (CH), 55.3 (CH_3), 27.2 (CH_3) ppm;

IR (ATR): $\tilde{v}$ = 3033, 2836, 1607, 1562, 1510, 1464, 1432, 1245, 1176, 1150, 1032 cm^{-1};

HR-MS (ESI): m/z calcd for $C_{15}H_{16}NO^+$ $[M+H]^+$ 226.1226, found 226.1237.

(*E*)-2-(2-(4-Methoxyphenyl)-2-(*p*-tolyl)vinyl)thiazole (3al) and (*Z*)-2-(2-(4-methoxyphenyl)-2-(*p*-tolyl)vinyl)thiazole (3al')

The general procedure was followed using (*E*)-4-(4-methoxystyryl)thiazole (94 mg, 0.50 mmol) and 1-iodo-4-methylbenzene (164 mg, 0.75 mmol). After 24 h, purification by column chromatography (*n*-hexane/EtOAc 8:1) yielded **3al** (65 mg, 42%) and **3al'** (45 mg, 23%) separately as pale-yellow soild.

For **3al**:

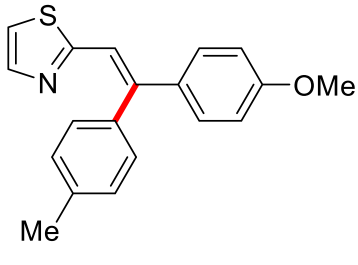

M.p.: 106 °C;

^{1}H NMR (400 MHz, $CDCl_3$) δ = 7.71 (d, *J* = 3.2 Hz, 1H), 7.52 (d, *J* = 1.0 Hz, 1H), 7.29 (d, *J* = 8.3 Hz, 2H), 7.21 – 7.11 (m, 4H), 7.07 – 7.02 (m, 3H), 3.90 (s, 3H), 2.35 (s, 3H) ppm;

^{13}C NMR (101 MHz, $CDCl_3$) δ = 166.4 (C_q), 160.2 (C_q), 146.3 (C_q), 141.9 (CH), 138.7 (C_q), 138.2 (C_q), 131.2 (CH), 130.7 (C_q), 129.3 (CH), 127.2 (CH), 121.2 (CH), 119.5 (CH), 115.3 (CH), 55.4 (CH_3), 21.3 (CH_3) ppm;

IR (ATR): $\tilde{v}$ = 2925,1604, 1507, 1464, 1284, 1245, 1177, 1029, 826, 724, 520, 488 cm^{-1};

HR-MS (ESI): *m*/*z* calcd. for $C_{19}H_{17}NOSNa^+$ $[M+Na]^+$ 330.0923, found 330.0919.

For **3al'**:

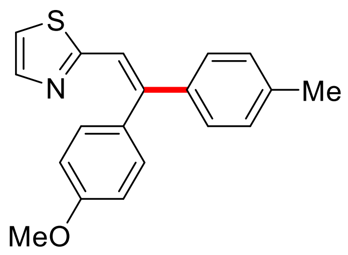

M.p.: 126 °C

^{1}H NMR (400 MHz, $CDCl_3$) δ = 7.70 (d, *J* = 3.3 Hz, 1H), 7.47 (d, *J* = 1.0 Hz, 1H), 7.36 – 7.31 (m, 4H), 7.15 (d, *J* = 8.0 Hz, 2H), 7.02 (dd, *J* = 3.3, 1.0 Hz, 1H), 6.89 – 6.83 (m, 2H), 3.81 (s, 3H), 2.47 (s, 3H) ppm;

^{13}C NMR (101 MHz, $CDCl_3$) δ = 166.5 (C_q), 160.1 (C_q), 146.3 (C_q), 141.7 (CH), 138.7 (C_q), 135.7 (C_q), 133.5 (CH), 130.6 (C_q), 129.7 (CH), 128.6 (CH), 120.1 (CH), 119.3 (CH), 114.0 (CH), 55.5 (CH_3), 21.6 (CH_3) ppm;

IR (ATR): $\tilde{\nu}$ = 2927,1599, 1509, 1465, 1247, 1180, 1031, 830, 727, 529, 484 cm^{-1};

HR-MS (ESI): *m*/*z* calcd. for $C_{19}H_{17}NOSNa^+$ $[M+Na]^+$ 330.0923, found 330.0925.

1-(2-(4-Methoxyphenyl)-2-phenylvinyl)-1*H*-pyrazole (3am)

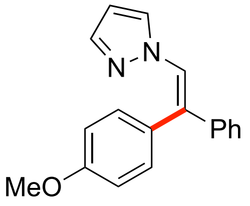

The general procedure was followed using (*E*)-1-styryl-1*H*-pyrazole (42.6 mg, 0.25 mmol), 1-iodo-4-methyoxybenzene (87.8 mg, 0.375 mmol), $[Ru(OAc)_2(p\text{-cymene})]$ (8.8 mg, 25 µmol), Na_2CO_3 (53.0 mg, 0.5 mmol) and DMA/1,4-dioxane (3:1, 0.75 mL). After 24 h, purification by column chromatography (toluene/EtOAc 20:1) and preparative gel permeation chromatography, the corresponding product was obtained as colorless solid (38.0 mg, 55%) as a non-separable mixture of the (*Z*)- and (*E*)-isomer.

Note: Analysis by ^{1}H NMR spectroscopy showed a ratio *Z*/*E* = 54:46.

M.p.: 73–75 °C

^{1}H NMR (300 MHz, $CDCl_3$) δ = 7.47 (d, *J* = 1.8 Hz, 1H), 7.46 (d, *J* = 1.8 Hz, 1H), 7.39 (s, 1H), 7.37 (s, 1H), 7.33 – 7.26 (m, 3H), 7.22 (s, 5H), 7.18 – 7.09 (m, 4H), 7.04 (d, *J* = 8.7 Hz, 2H), 6.87 – 6.82 (m, 3H), 6.78 (d, *J* = 8.8 Hz, 2H), 6.73 (d, *J* = 2.6 Hz, 1H), 6.02 (t, *J* = 2.2 Hz, 1H), 5.98 (t, *J* = 2.2 Hz, 1H), 3.76 (s, 3H), 3.73 (s, 3H) ppm;

^{13}C NMR (75 MHz, $CDCl_3$) δ = 159.6 (C_q), 159.5 (C_q), 140.6 (C_q), 140.1 (CH), 140.0 (CH), 138.3 (C_q), 132.8 (C_q), 131.2 (CH), 131.2 (C_q), 131.1 (C_q), 130.1 (C_q), 130.0 (CH), 129.3 (CH), 129.2 (CH), 129.2 (CH), 128.7 (CH), 128.5 (CH), 128.2 (CH), 127.9 (CH), 127.6 (CH), 125.4 (CH), 124.3 (CH), 114.6 (CH), 114.0 (CH), 106.6 (CH), 106.5 (CH), 55.4 (CH_3), 55.4 (CH_3) ppm;

IR (ATR): $\tilde{\nu}$ = 1606, 1510, 1438, 1393, 1247, 1177, 1031, 832, 751, 700 cm^{-1};

HR-MS (ESI): *m*/*z* calcd. for $C_{18}H_{16}N_2ONa^+$ $[M+Na]^+$ 299.1155, found 299.1153.

5 Comparison Chemoselectivity Thermal vs. Photochemical Conditions

To evaluate beneficial effects of our photochemical approach in comparison to conventional thermal heating, two analog reactions were conducted.

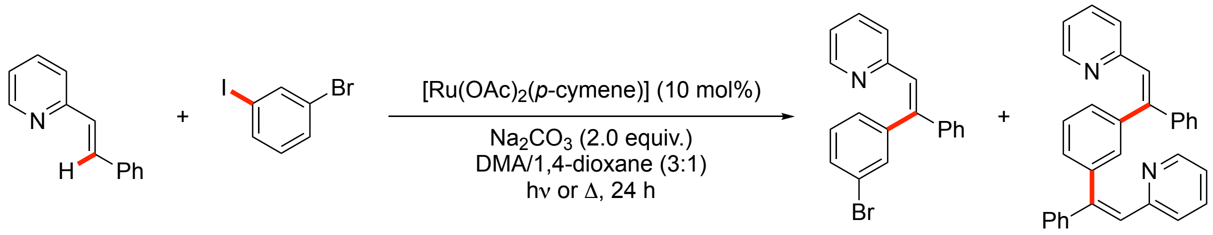

1a (2.2 equiv) (0.5 mmol) **3an** **3an’**

Photochemical Conditions

The general procedure was followed with modified amounts of the coupling agents as described in the following: (*E*)-2-styrylpyridine (199 mg, 1.10 mmol, 2.2 equiv.) and 1-bromo-3-iodobenzene (141 mg, 0.50 mmol, 1.0 equiv.). After 24 h, purification by column chromatography (*n*-hexane/EtOAc) yielded **3an** (63.8 mg, 38%) as brown sticky oil. The formation of **3an’** was not observed.

Thermal Conditions

A 10 mL glass vial was charged with (*E*)-2-styrylpyridine (199 mg, 1.10 mmol, 2.2 equiv.), 1-bromo-3-iodobenzene (141 mg, 0.50 mmol, 1.0 equiv.), [Ru(OAc)$_2$(*p*-cymene)] (18 mg, 50.0 µmol, 10 mol %), and Na_2CO_3 (106 mg, 1.00 mmol, 2.0 equiv.). The vial was capped with a septum and wrapped with parafilm. The vial was evacuated and purged with N_2 three times. The mixture of DMA and 1,4-dioxane (3:1, 1.5 mL) was then added and the resulting reaction mixture was stirred at 120 °C. After 24 h, the resulting mixture was cooled down to room temperature, filtered through a pad of silica gel and washed with diethyl ether. The filtrate was concentrated in vacuo. Purification of the residue by column chromatography (*n*-hexane/EtOAc) yielded a **3an** (33.6 mg, 20%) as brown oil and **3an’** (152.2 mg, 70%) as reddish oil.

Characterization Data

(*Z*)-2-(2-(3-Bromophenyl)-2-phenylvinyl)pyridine (3an)

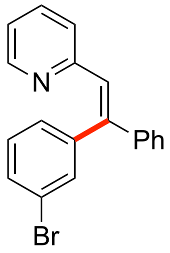

^{1}H NMR (400 MHz, CDCl$_3$) δ = 8.54 (ddd, *J* = 4.9, 1.9, 1.0 Hz, 1H), 7.48 (ddd, *J* = 7.9, 2.1, 1.2 Hz, 1H), 7.40 – 7.30 (m, 7H), 7.22 (t, *J* = 7.8 Hz, 1H), 7.17 – 7.13 (m, 2H), 7.03 (ddd, *J* = 7.5, 4.8, 1.1 Hz, 1H), 6.74 (dt, *J* = 8.1, 1.1 Hz, 1H) ppm;

^{13}C NMR (101 MHz, CDCl$_3$) δ = 156.1 (C$_q$), 149.5 (CH), 144.2 (C$_q$), 142.1 (C$_q$), 141.9 (C$_q$), 135.5 (CH), 132.9 (CH), 130.3 (CH), 129.4 (CH), 129.0 (CH), 128.4 (CH), 128.3 (CH), 127.8 (CH), 123.8 (CH), 122.7 (C$_q$), 121.5 (CH) ppm;

IR (ATR): $\tilde{\nu}$ = 3060, 3031, 3001, 2944, 1582, 1532, 1492, 1445, 1292, 1151, 1070, 992 cm^{-1};

HR-MS (ESI): *m*/*z* calcd. for $C_{19}H_{15}BrN^+$ [M+H]$^+$ 336.0382, found 336.0392.

1,3-Bis((*Z*)-1-phenyl-2-(pyridin-2-yl)vinyl)benzene (3an')

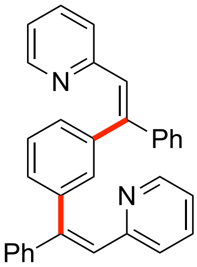

^{1}H NMR (400 MHz, CDCl$_3$) δ = 8.51 (ddd, *J* = 4.8, 1.9, 1.0 Hz, 2H), 7.41 – 7.33 (m, 3H), 7.35 – 7.24 (m, 10H), 7.20 (dd, *J* = 7.6, 1.7 Hz, 2H), 7.13 (s, 2H), 7.10 (t, *J* = 1.7 Hz, 1H), 7.01 (ddd, *J* = 7.5, 4.9, 1.2 Hz, 2H), 6.85 (dt, *J* = 8.2, 1.1 Hz, 2H) ppm;

^{13}C NMR (101 MHz, CDCl$_3$) δ = 156.5 (C$_q$), 149.4 (CH), 145.4 (C$_q$), 142.2 (C$_q$), 140.6 (C$_q$), 135.2 (CH), 131.7 (CH), 129.6 (CH), 129.4 (CH), 128.7 (CH), 128.2 (CH), 128.1 (CH), 127.7 (CH), 124.0 (CH), 121.3 (CH) ppm;

IR (ATR): $\tilde{\nu}$ = 3052, 3024, 3000, 2925, 1643, 1582, 1492, 1432, 1293, 1150, 1032, 990 cm^{-1};

HR-MS (ESI): *m*/*z* calcd. for $C_{32}H_{25}N_2^+$ [M+H]$^+$ 437.2012, found 437.2021.

6 Comparison Ruthenium-Catalysts

During the optimization of the reaction, an initially similar catalytic efficacy was observed for the well-defined [Ru(OAc)$_2$(*p*-cymene)] complex and the [RuCl$_2$(*p*-cymene)]$_2$ complex in combination with substoichiometric amounts of acetate additives (*vide supra*, Table S3). A more detailed investigation on the activity of these two catalytic systems was performed analog to the general procedure, showing superior activities for [Ru(OAc)$_2$(*p*-cymene)] as catalyst in this reaction for various substrates.

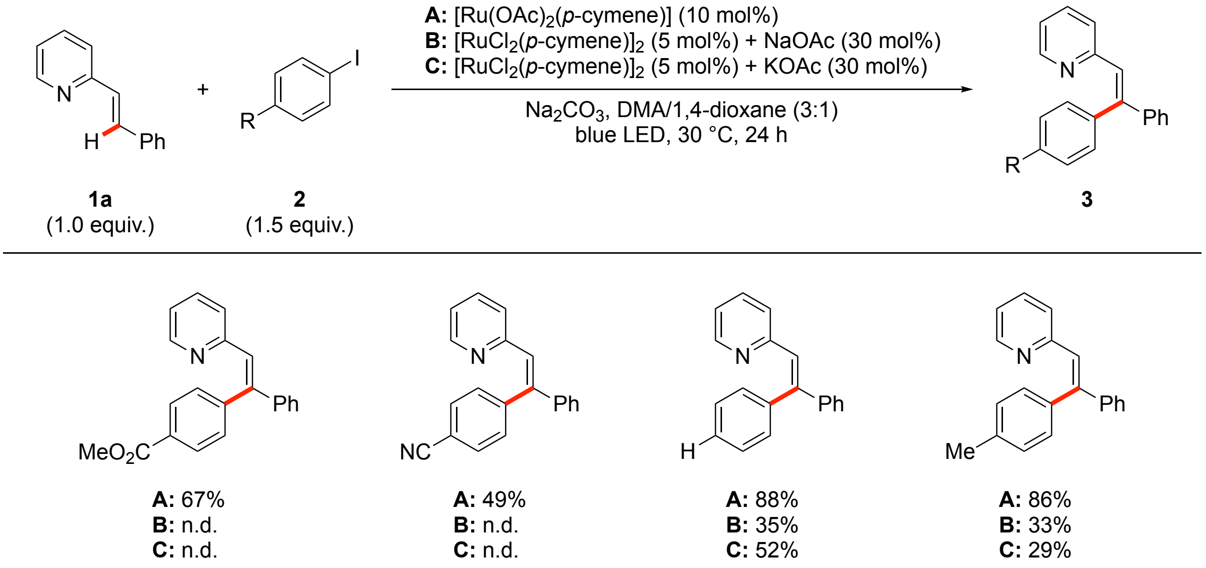

7 Mechanistic Studies

7.1 Reaction with Cyclometallated Ru-Complex as Catalyst

Synthesis of Ru-I

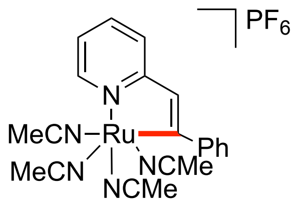

Ru-I

The synthesis of ruthenacycle **Ru-I** followed an adapted procedure reported in the literature.[3] An oven dried 10 mL Ace pressure tube equipped with a stirring bar was transferred to a nitrogen-filled glove box, then $[RuCl_2(p\text{-cymene})]_2$ (245 mg, 0.4 mmol, 0.5 equiv.), KOAc (117.8 mg, 1.2 mmol, 1.5 equiv.), KPF_6 (295 mg, 1.6 mmol, 2 equiv.), (*E*)-2-styrylpyridine (145 mg, 0.8 mmol, 1 equiv.) and MeCN (5 mL, 0.16 M) were added. The tube was sealed, transferred out of the box, placed in an oil bath at 100 °C and stirred for 24 h. Upon completion, the reaction crude was loaded on an aluminium oxide (Al_2O_3, neutral) column conditioned with CH_2Cl_2 and quickly eluted with MeCN/CH_2Cl_2 (1:1) using N_2 in replacement of compressed air collecting the yellow/orange band. The solution was concentrated under reduced pressure and then quickly precipitated with Et_2O affording the desired cyclometallated Ru-complex **Ru-I** as a yellow/orange solid (112.8 mg, 24%). The complex was promptly transferred to a glove box as it decomposes turning green if exposed to air over time.

^{1}H NMR (400 MHz, CD_3CN) δ = 8.72 (ddd, *J* = 5.6, 1.6, 0.7 Hz, 1H), 7.58 (ddd, *J* = 8.0, 7.4, 1.6 Hz, 1H), 7.36 – 7.30 (m, 4H), 7.27 (ddd, *J* = 8.0, 1.4, 0.9 Hz, 1H), 7.24 – 7.18 (m, 1H), 6.93 (ddd, *J* = 7.3, 5.7, 1.5 Hz, 1H), 6.91 (s, 1H), 2.13 (s, 3H), 2.12 (s, 6H), 1.96 (s, 3H) ppm;

^{13}C NMR (101 MHz, CD_3CN) δ 214.3, 171.6, 153.2, 152.6, 136.8, 130.3, 128.5, 126.4, 126.2, 124.2, 122.6, 120.0, 118.6, 4.0, 3.9.

^{19}F NMR (337 MHz, CD_3CN) δ = -72.0 (s), -73.8 (s) ppm;

^{31}P NMR (162 MHz, CD_3CN) δ = -146.8 (hept, *J* = 711 Hz) ppm.

Ru-I in arylation reaction

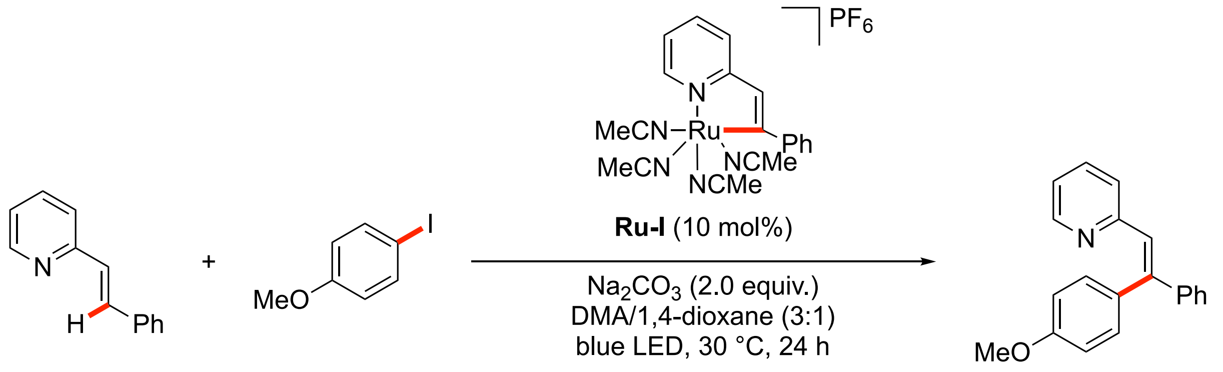

1a
(0.5 mmol)

2a
(1.5 equiv.)

3a

The reaction with a cyclometallated Ru-complex as catalyst followed the general procedure using (*E*)-2-styrylpyridine (90.5 mg, 0.50 mmol) and 1-iodo-4-methoxybenzene (176 mg, 0.75 mmol), and **Ru-I** (22.3 mg, 50 µmol, 10 mol%) instead of [Ru(OAc)$_2$(*p*-cymene)]. Besides a) the photochemical conditions with blue LED irradiation, two additional reactions were performed in the dark at b) ambient temperature and at c) 30 °C. The crude reaction mixtures were analyzed by ^{1}H NMR spectroscopy and in case of the photochemical reaction a), the product has been purified by column chromatography.

Table S6. Effect of the blue light irradiation on the yield of **3b**.

Condition	Condition details	Yield of 3b (%)
a)	Blue LED, 30 °C	77
b)	In dark, 25 °C	(14)
c)	In dark, 30 °C	(21)

Conditions: **1a** (0.5 mmol), **2a** (0.45 mmol), **Ru-I** (10 mol%), Na_2CO_3 (2 equiv.), N_2, 24 h, DMA:1,4-dioxane (3:1, 1.5 mL), isolated yield. Yield in the parentheses was determined by ^{1}H-NMR using CH_2Br_2 as the internal standard.

Note: The reaction of 1 equiv. **Ru-I** with 1.5 equiv. **2a** in DMA/1,4-dioxane (3:1) did not furnish the corresponding product **3a** after 24 h under blue LED irradiation.

7.2 Radical Scavenger Experiments

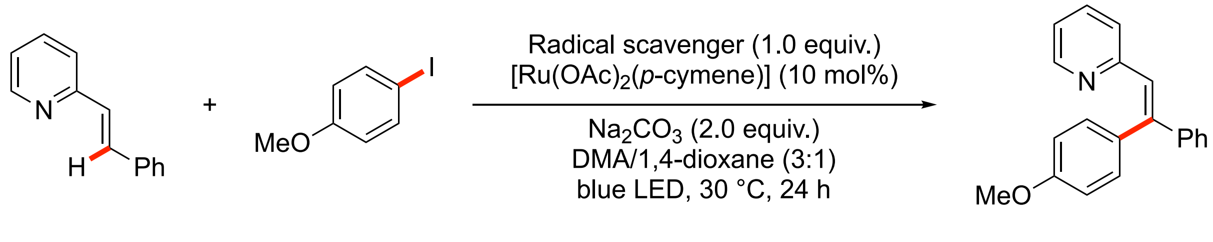

To evaluate whether a radical pathway is likely, the general procedure was followed using (*E*)-2-styrylpyridine (90.5 mg, 0.50 mmol) and 1-iodo-4-methoxybenzene (176 mg, 0.75 mmol), and a radical scavenger (1.0 equiv.) was added. After 24 h, the crude reaction mixture was analyzed by HR-MS and ^{1}H NMR spectroscopy with CH_2Br_2 as internal standard.

Table S7. Observed yield for the radical scavenger experiments.

Radical Scavenger	^{1}H NMR yield of 3a
TEMPO	12%
BHT	30%
Glavinoxyl	0%
DPPH	0%

Conditions: **1a** (0.5 mmol), **2a** (0.75 mmol), $[Ru(OAc)_2(p\text{-cymene})]$ (10 mol%), Na_2CO_3 (2 equiv.), N_2, 24 h, DMA:1,4-dioxane (3:1, 1.5 mL). TEMPO = (2,2,6,6-Tetramethylpiperidin-1-yl)oxy. BHT = Butylated hydroxytoluene. DPPH = 2,2-Diphenyl-1-picrylhydrazyl.

The following species were detected by ESI MS:

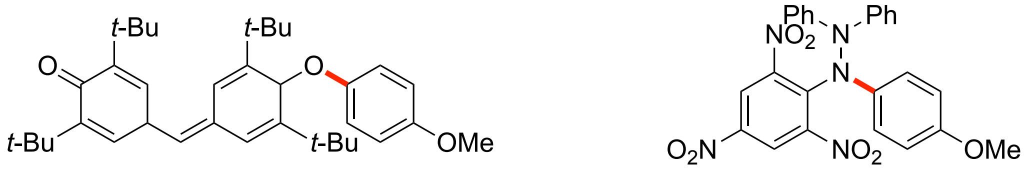

Glavinoxyl adduct
m/*z* calcd. for $C_{36}H_{51}O_3^+$ $[M+H]^+$: 531.38
found 531.35

DPPH adduct
m/*z* calcd. for $C_{25}H_{19}N_5O_7Na^+$ $[M+Na]^+$: 524.12
found 524.10

7.3 On/Off Experiment

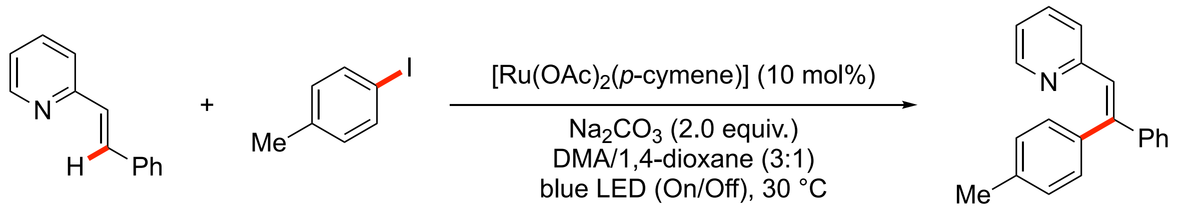

1a **3b**

A 10 mL glass vial was charged with (*E*)-2-styrylpyridine (181 mg, 1 mmol, 1.0 equiv.), 1-iodo-4-methylbenzene (218 mg, 1.5 mmol, 1.5 equiv.), [Ru(OAc)$_2$(*p*-cymene)] (36 mg, 100 µmol, 10 mol%), 1,3,5-trimethoxybenzene (1 mmol, 168 mg) as internal standard, and Na_2CO_3 (212 mg, 2.00 mmol, 2 equiv.). The vial was capped with a septum and wrapped with parafilm. The vial was evacuated and purged with N_2 three times, and the mixture was stirred with a N_2 ballon under visible light irradiation. After the time intervals indicated below, the light was turned off or on, respectively. Additionally, after every interval, an aliquot of 100 µL was collected using a syringe, which was filtered through a short plug of silica gel and washed with EtOAc. After removal of the solvent, the residue was analyzed by ^{1}H NMR spectroscopy.

Table S8. Effect of the blue light irradiation on the yield of **3b**.

Time (h)	Light	Yield of 3b (%)
2	On	14
4	Off	15
6	On	27
8	Off	29
10	On	44
11	Off	45
12	On	56

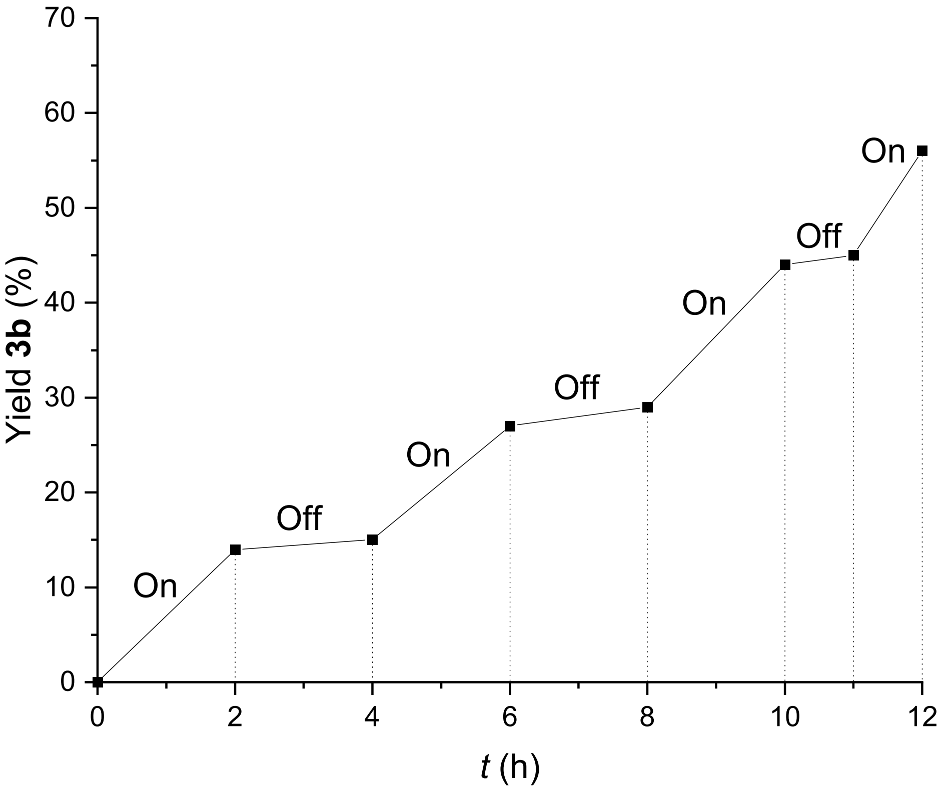

Figure S1. Graphical representation of the yield evolution during the on/off experiment.

7.4 UV/Vis Spectroscopy

For analysis of the reagents and reagent combinations via UV/Vis spectroscopy, the following sample solutions were prepared in a N_2-filled glovebox as follows:

1. Substrate **1a** (0.10 mmol) in 5.0 mL of DMA
2. Substrate **2a** (0.10 mmol) in 5.0 mL of DMA
3. [Ru(OAc)$_2$(*p*-cymene)] (0.010 mmol) in 5.0 mL of DMA
4. Substrate **1a** (0.10 mmol) + [Ru(OAc)$_2$(*p*-cymene)] (0.010 mmol) in 5.0 mL of DMA
5. Substrate **2a** (0.10 mmol) + [Ru(OAc)$_2$(*p*-cymene)] (0.010 mmol) in 5.0 mL of DMA
6. [**Ru-I]** (0.010 mmol) in 5.0 mL of DMA
7. Substrate **1a** (0.10 mmol) + [**Ru-I]** (0.010 mmol) + KOAc (0.020 mmol) in 5.0 mL of DMA
8. Substrate **1a** (0.10 mmol) + [**Ru-I]** (0.010 mmol) in 5.0 mL of DMA
9. [**Ru-I]** (0.010 mmol) + KOAc (0.020 mmol) in 5.0 mL of DMA
10. **3a** (0.010 mmol) in 5.0 mL of DMA

Then, solutions **1**, **2**, **4** and **5** were removed from the glovebox and stirred under 450 nm LEDs for 24 h. The solutions were introduced into the glovebox, and 200 µL of each solution were collected to a cuvette and diluted to the final volume of 3.0 mL. The cuvettes were removed from the glovebox, and the samples were subjected to UV/Vis absorption measurements.
Solutions **3**, **6** and **10** were stirred for 10 min. Then, 200 µL of the solutions were filled in the cuvette and diluted to the final volume of 3.0 mL. The cuvettes were removed from the glovebox, and the samples were subjected to UV/Vis absorption measurements.
Solutions **7**, **8**, and **9** were stirred for 24 h at room temperature in the glovebox, and 200 µL of the solutions were filled in the cuvette and diluted to the final volume of 3.0 mL. The cuvettes were removed from the glovebox, and the samples were subjected to UV/Vis absorption measurements.

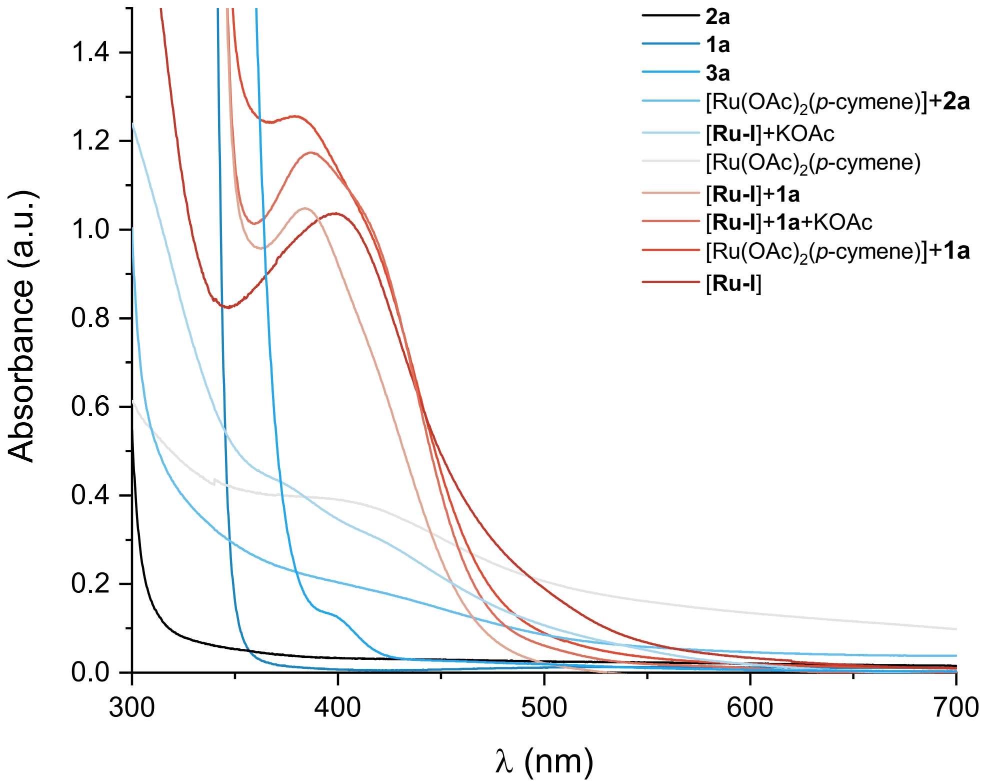

Figure S2. UV/Vis spectra of reagents and ruthenium complexes.

7.5 Determination of Quantum Yield

The quantum yield of the reaction has been determined using the ferrioxalate actinometer. Here, the procedure was used as described by G. Gauglitz and S. Hubig and was carried out in the dark.[4]

Initially three solutions were prepared as described as follows:

1) Potassium ferrioxalate trihydrate (245.5 mg) and 0.5 M solution H_2SO_4 in water (5 mL) were diluted with water to a total volume of 50 mL.
2) 1,10-Phenantroline (166.6 mg) was dissolved in 100 mL of water. To enable full solvation, the mixture was sonicated for 10 min at 40 °C.
3) A buffered, aqueous solution was prepared by diluting NaOAc (3.486 g) and H_2SO_4 (0.5 mL) with water to a total volume of 50 mL.

Next, 3 mL of solution 1) (V_1) were filled in a 10 mL glass vial which was stirred vigorously for 10 s. Afterwards 1 mL (V_2) of the irradiated solution was treated with 4 mL of solution 2) and 0.5 mL of solution 3). The resulting mixture was diluted with water to a total volume of 10 mL (V_3) and the solution was stored in the dark for 1 h. This process was repeated two additional times, and the solutions were placed in different positions of the photochemical setup to evaluate the position-sensitivity of the light intensity to determine an average quantum yield. Moreover, reference solutions were prepared in an analog procedure but without irradiation. Finally, UV/Vis analysis of the samples (optical pathlength l = 1 cm) was performed to determine the absorption A(510 nm) at 510 nm.

The following absorbances A(510 nm) were determined:

Table S9: Absorption A(510 nm) for irradiated and non-irradiated ferrioxalate solutions.

	**A_1(510 nm)**	**A_2(510 nm)**	**A_3(510 nm)**	**øA(510 nm)**
Irradiated	4.0457	3.7725	3.3503	3.7223
Non-Irradiated	0.0348	0.0380	0.0592	0.0440

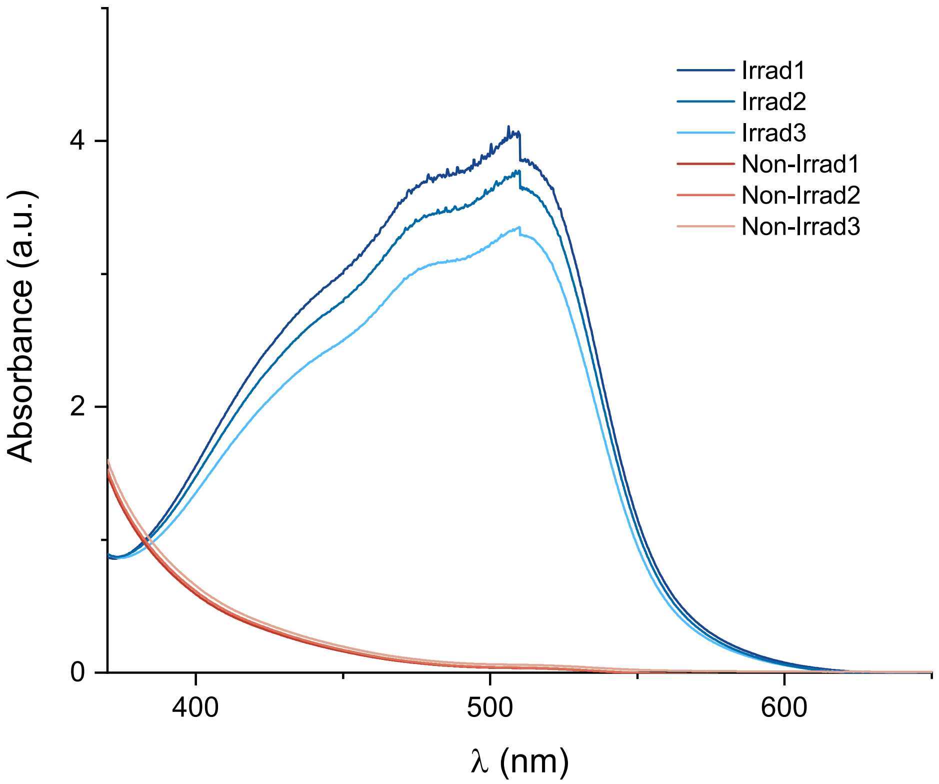

Figure S3. UV/Vis spectra of irradiated (Irrad) and non-irradiated (Non-Irrad) samples for the ferrioxalate actinometer.

The photon flux can be determined using the following equation:

$$q_{n,\mathrm{p}} = \frac{\Delta A \cdot V_1 \cdot V_3}{\Phi(\lambda) \cdot \epsilon(510\ \mathrm{nm}) \cdot V_2 \cdot l \cdot t}$$

Based on the determined differences in absorbance obtained from Table S9, the quantum yield of the ferrioxalate actinometer $\Phi(\lambda)$ at 458 nm (0.01 M) (Note: it is assumed that the literature value for the quantum yield at 458 nm is a good approximation for the quantum yield at 450 nm) $\Phi(458\ \mathrm{nm}) = 1.12$[3], and $\epsilon(510\ \mathrm{nm}) = 11100\ \mathrm{L\ mol^{-1}\ cm^{-1}}$, the photon flux is:

$$q_{n,\mathrm{p}} = \frac{(3.7223 - 0.0440) \cdot 3 \cdot 10^{-3}\ \mathrm{L} \cdot 10 \cdot 10^{-3}\ \mathrm{L}}{1.12 \cdot 1110000\ \mathrm{L\ mol^{-1}\ m^{-1}} \cdot 1 \cdot 10^{-3}\ \mathrm{L} \cdot 1 \cdot 10^{-2}\ \mathrm{m} \cdot 10\ \mathrm{s}}$$

$$q_{n,\mathrm{p}} = 8.8762 \cdot 10^{-7}\ \mathrm{mol\ s^{-1}}$$

The quantum yield for the Ru-catalyzed arylation reaction can now be determined as the quotient of the product formation rate, which was extracted from the initial rate in the interval from 0 to 2 h from Figure S1, and the photon flux $q_{n,\mathrm{p}}$:

$$\text{Quatum Yield} = \frac{\text{product formation rate}}{q_{n,\mathrm{p}}}$$

$$\text{Quatum Yield} = \frac{1.9444 \cdot 10^{-8}\text{mol s}^{-1}}{8.8762 \cdot 10^{-7}\text{ mol s}^{-1}} = 0.0219 = 2\%$$

8 Isomerization Study

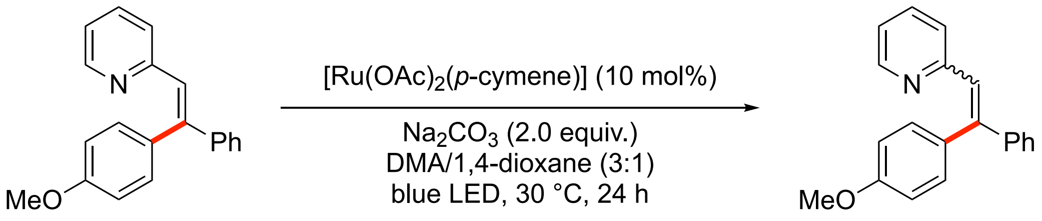

To evaluate the origin of the product isomerization for some of the products, a series of experiments was conducted. In a typical procedure, (*Z*)-2-(2-(4-methoxyphenyl)-2-phenylvinyl)pyridine **3a** (0.1 mmol, 28.7 mg) and [Ru(OAc)$_2$(*p*-cymene)] (10 mol%) were dissolved in a mixture of DMA and 1,4-dioxane (3:1, 1.5 mL). Then, the vial was removed from the glovebox and stirred under visible light irradiation with the wavelength of 450 nm. After 24 h, the resulting mixture was filtered through a pad of silica gel and washed with diethyl ether. The filtrate was concentrated in vacuo. The residue has been analyzed by ^{1}H NMR to determine the yield and the degree of isomerization using dibromomethane (1.0 equiv.) as internal standard.

Similar experiments were conducted with variations as follows:

Table S9. Isomerization study.

Deviation from above	Yield (*Z*)-3a	Yield (*E*)-3a	(*Z*)/(*E*) Ratio
---	32%	47%	41:59
No [Ru(OAc)$_2$(*p*-cymene)]	55%	40%	58:42
No light	89%	0%	100:0
120 °C instead of light	92%	0%	100:0

Based on these results, we conclude that the isomerization of (*Z*)-**3a** is a purely light-induced process, probably being caused by an excitation of the double bond, leading to a triplet state. Free rotation along the C–C bond then allows the isomerization.

9 References

[1] J. L. R. William, R. E. Adel, J. M. Carlson, G. A. Reynolds, D. G. Borden, J. A. Ford, *J. Org. Chem.* **1963**, *28*, 387–390.

[2] D. Zell, S. Warratz, D. Gelman, S. J. Garden, L. Ackermann, *Chem. Eur. J.* **2016**, *22*, 1248–1252.

[3] Gang. W. Wang, M. Wheatley, M. Simonetti, D. M. Cannas, I. Larrosa, *Chem* **2020**, *6*, 1459–1468.

[4] H. J. Kuhn, S. E. Braslavsky, R. Schmidt, Chemical actinometry (IUPAC Technical Report). *Pure Appl. Chem.* **2004**, *76*, 2105–2146.

10 NMR Spectra

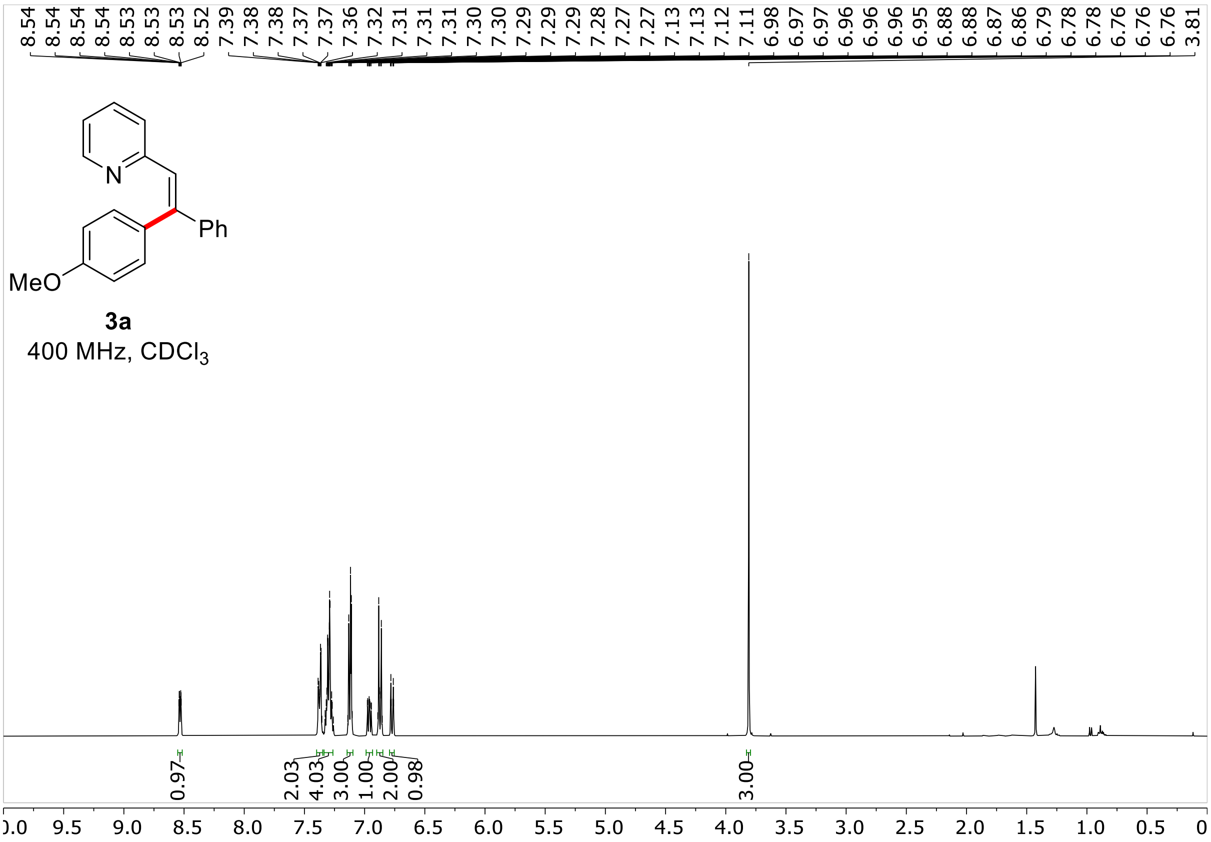

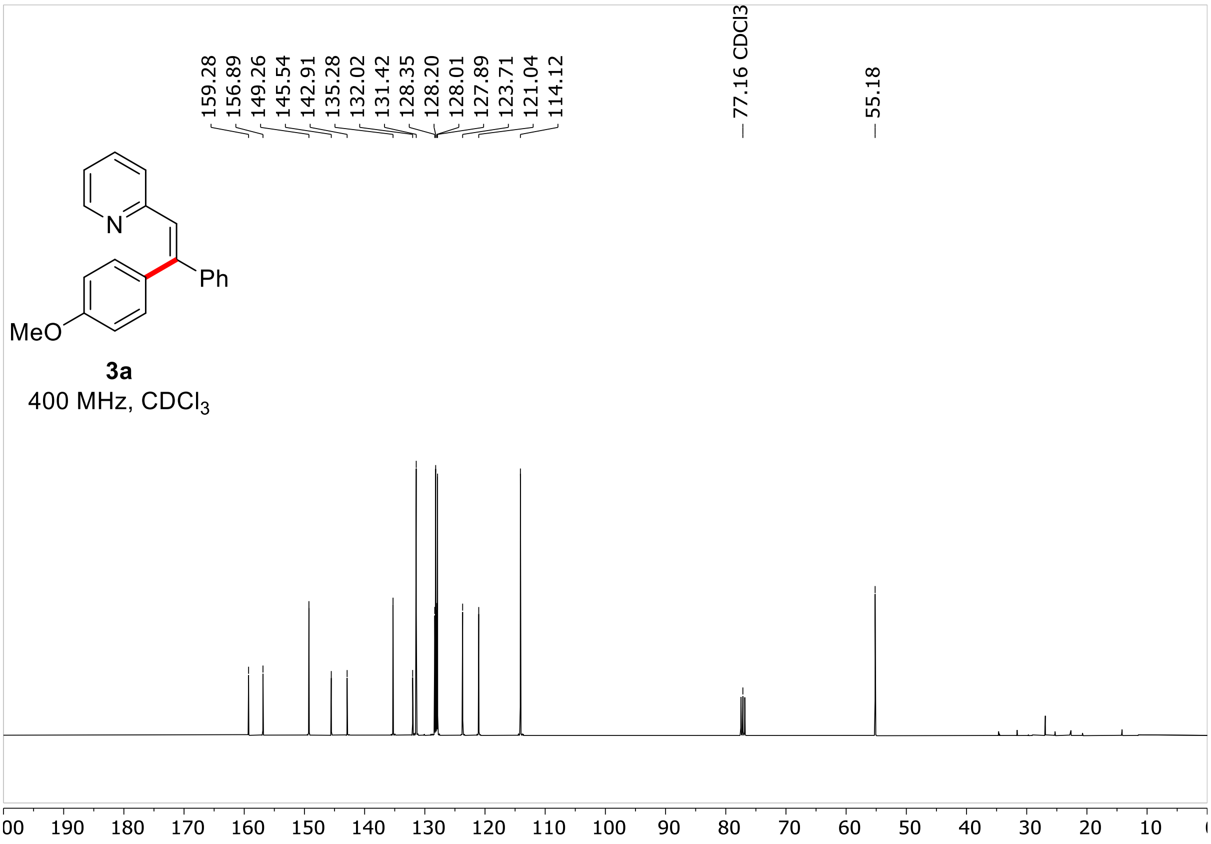

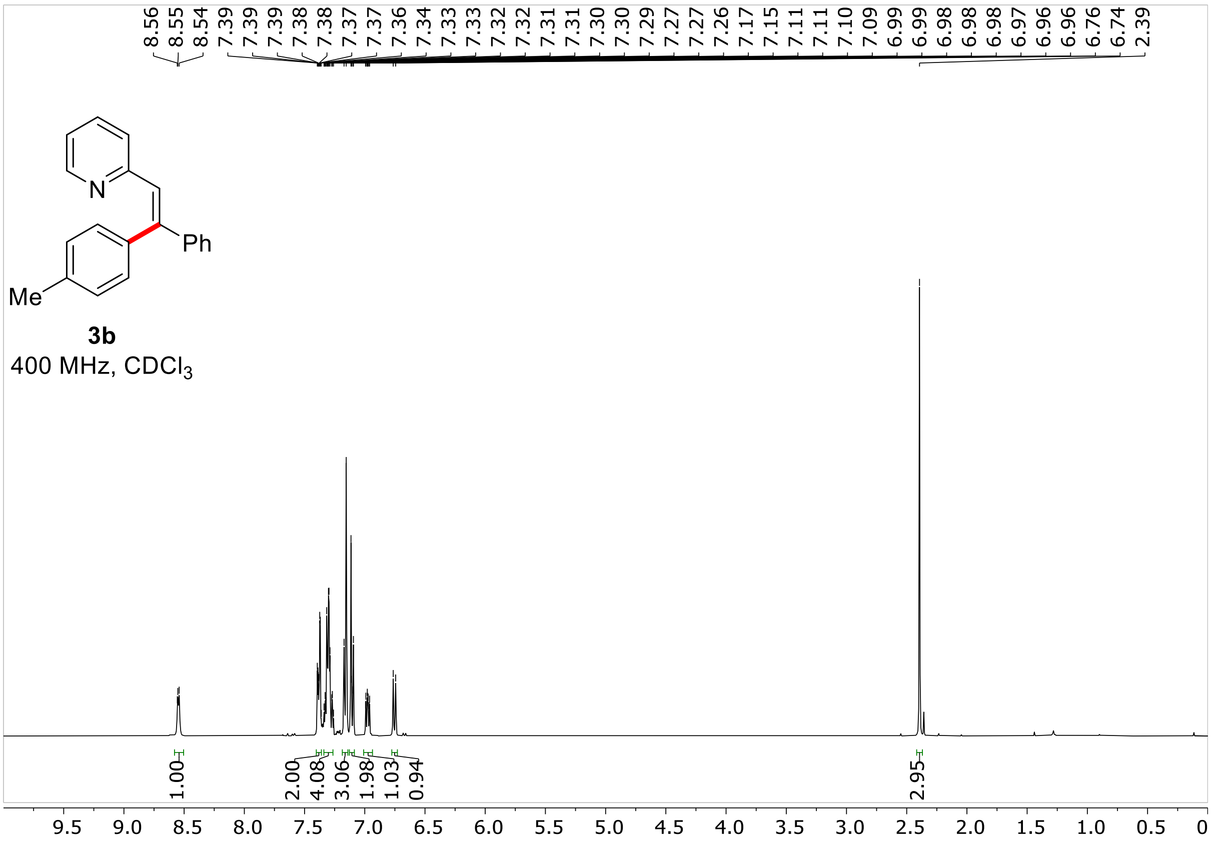
8.56 8.55 8.54 7.39 7.39 7.39 7.38 7.38 7.37 7.37 7.36 7.34 7.33 7.33 7.32 7.32 7.31 7.31 7.30 7.30 7.29 7.27 7.27 7.26 7.17 7.15 7.11 7.11 7.10 7.09 6.99 6.99 6.98 6.98 6.98 6.97 6.96 6.96 6.76 6.74 2.39
N
Ph
Me
3b
400 MHz, CDCl3
1.00 2.00 4.08 3.06 1.98 1.03 0.94 2.95
9.5 9.0 8.5 8.0 7.5 7.0 6.5 6.0 5.5 5.0 4.5 4.0 3.5 3.0 2.5 2.0 1.5 1.0 0.5 0

z

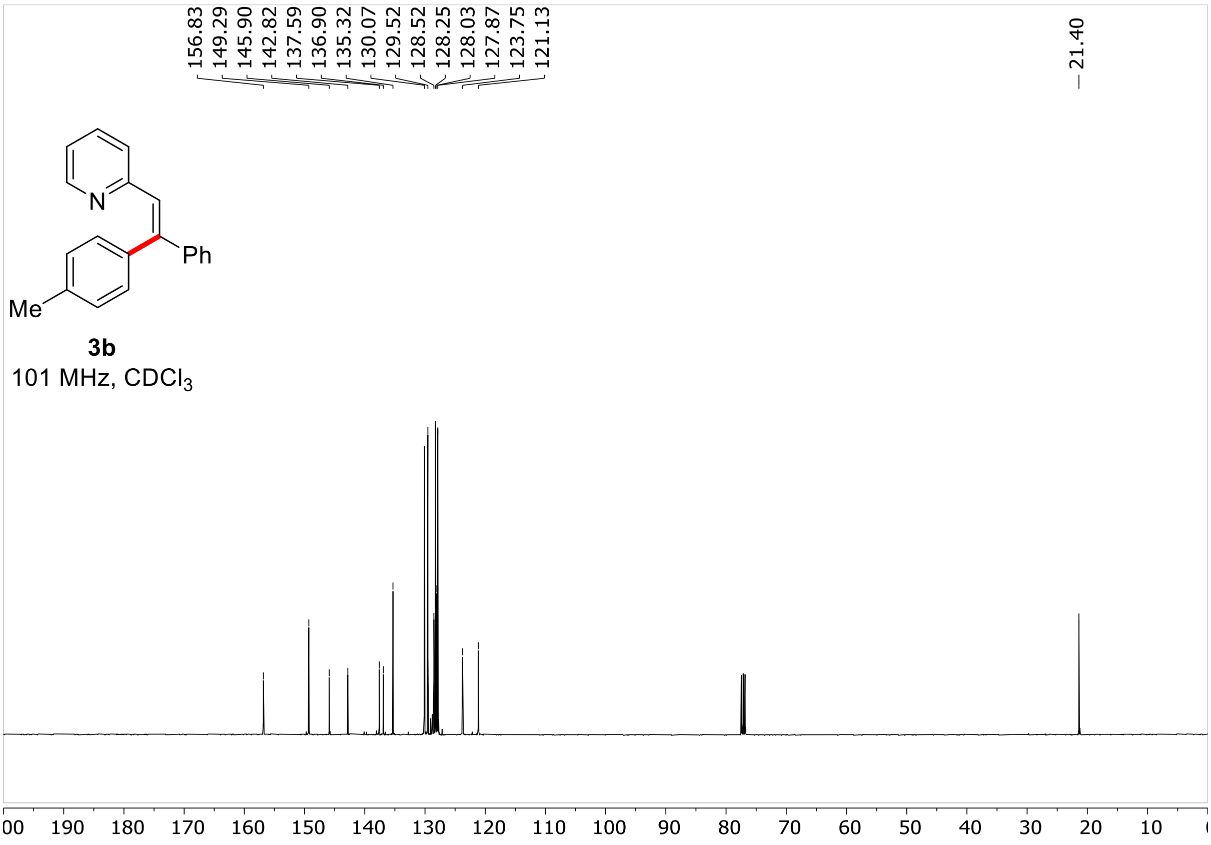
156.83 149.29 145.90 142.82 137.59 136.90 135.32 130.07 129.52 128.52 128.25 128.03 127.87 123.75 121.13
21.40
N
Ph
Me
3b
101 MHz, CDCl3
190 180 170 160 150 140 130 120 110 100 90 80 70 60 50 40 30 20 10

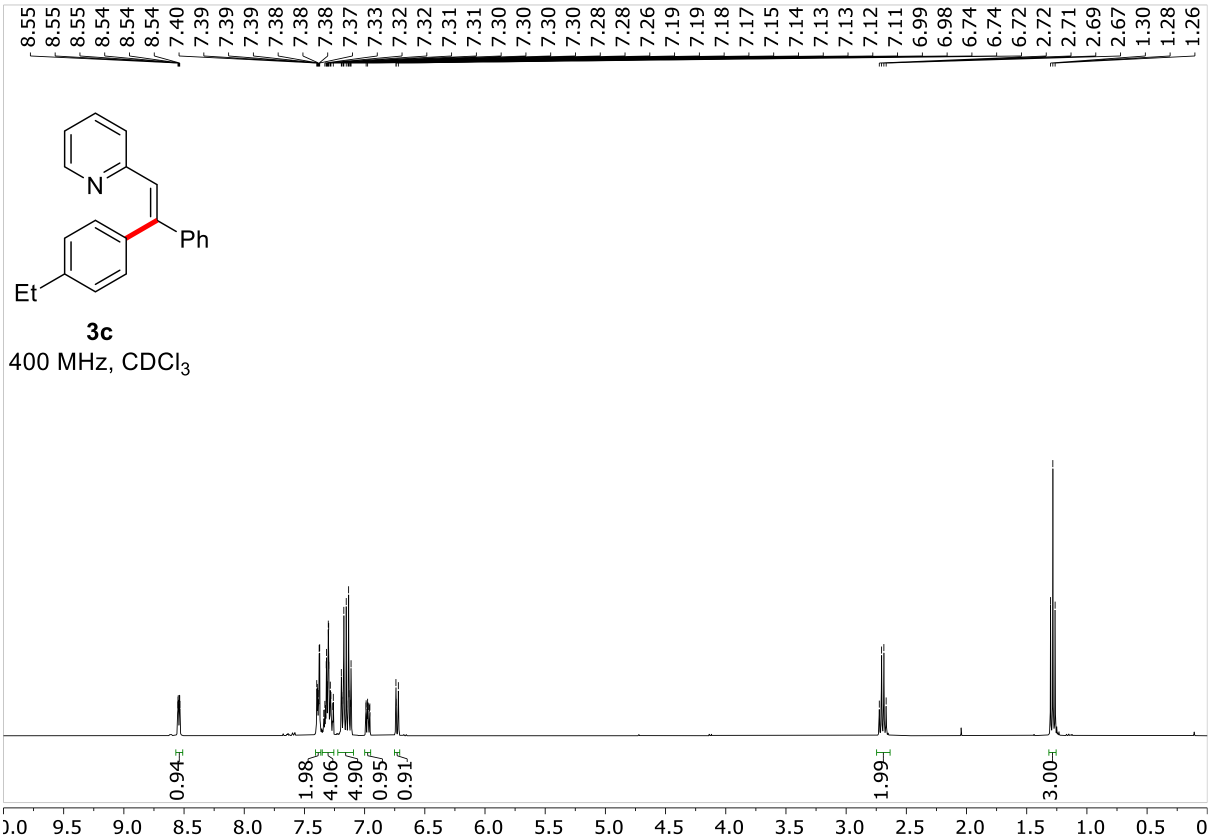
8.55
8.55
8.55
8.54
8.54
8.54
7.40
7.39
7.39
7.39
7.38
7.38
7.38
7.37
7.33
7.32
7.32
7.31
7.31
7.30
7.30
7.30
7.30
7.28
7.28
7.26
7.19
7.19
7.18
7.17
7.15
7.14
7.13
7.13
7.12
7.11
6.99
6.98
6.74
6.74
6.72
2.72
2.71
2.69
2.67
1.30
1.28
1.26
N
Ph
Et
3c
400 MHz, CDCl3
0.94
1.98
4.06
4.90
0.95
0.91
1.99
3.00
9.5
9.0
8.5
8.0
7.5
7.0
6.5
6.0
5.5
5.0
4.5
4.0
3.5
3.0
2.5
2.0
1.5
1.0
0.5
0

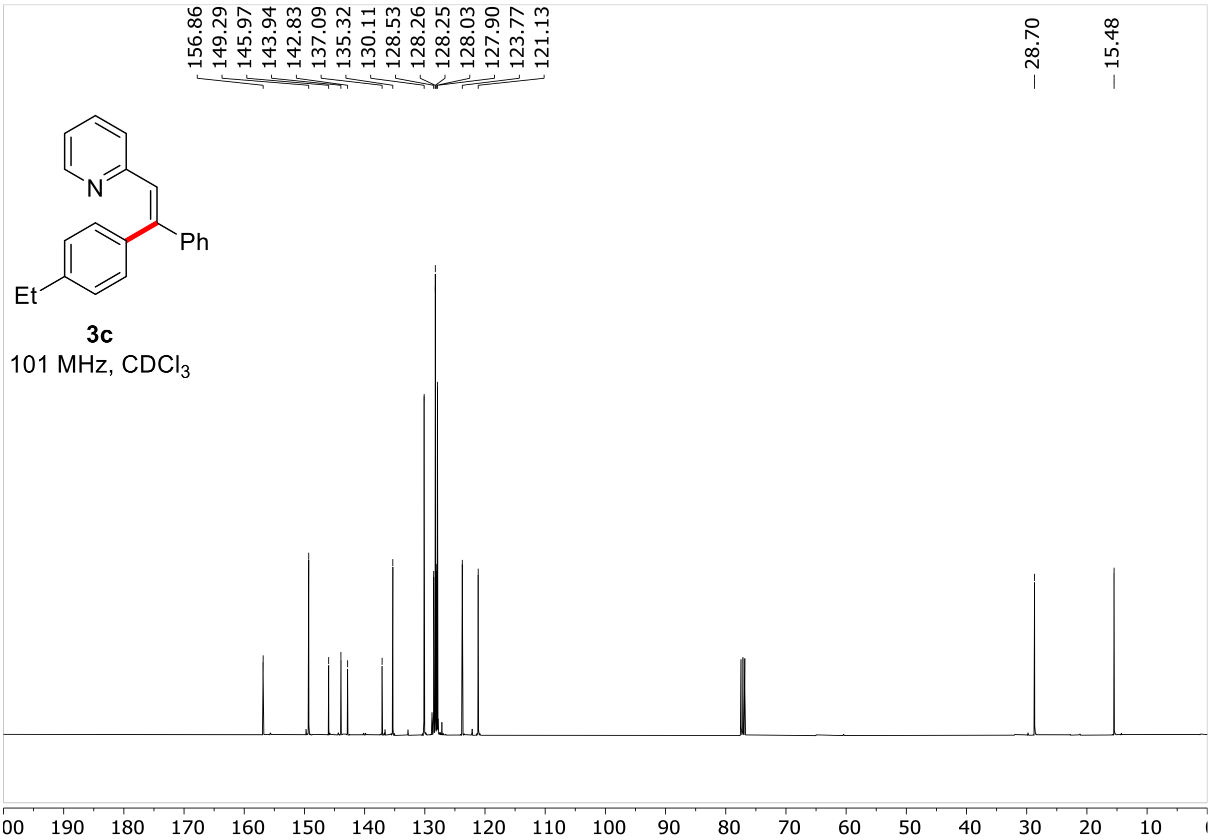
156.86
149.29
145.97
143.94
142.83
137.09
135.32
130.11
128.53
128.26
128.25
128.03
127.90
123.77
121.13
28.70
15.48
N
Ph
Et
3c
101 MHz, CDCl3
190
180
170
160
150
140
130
120
110
100
90
80
70
60
50
40
30
20
10

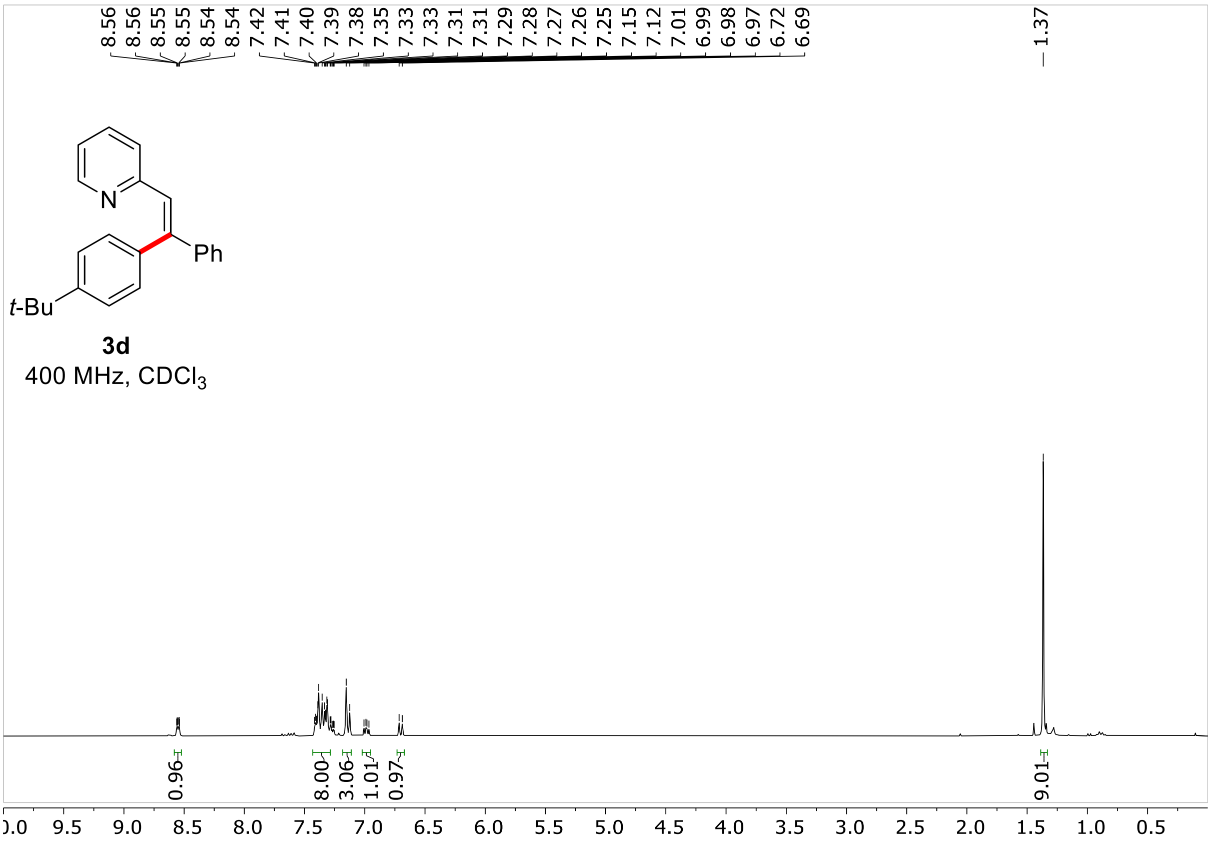

8.56 8.56 8.55 8.55 8.54 8.54 7.42 7.41 7.40 7.39 7.38 7.35 7.33 7.33 7.31 7.31 7.29 7.28 7.27 7.26 7.25 7.15 7.12 7.01 6.99 6.98 6.97 6.72 6.69
1.37
N
Ph
t-Bu
3d
400 MHz, CDCl3
0.96
8.00
3.06
1.01
0.97
9.01

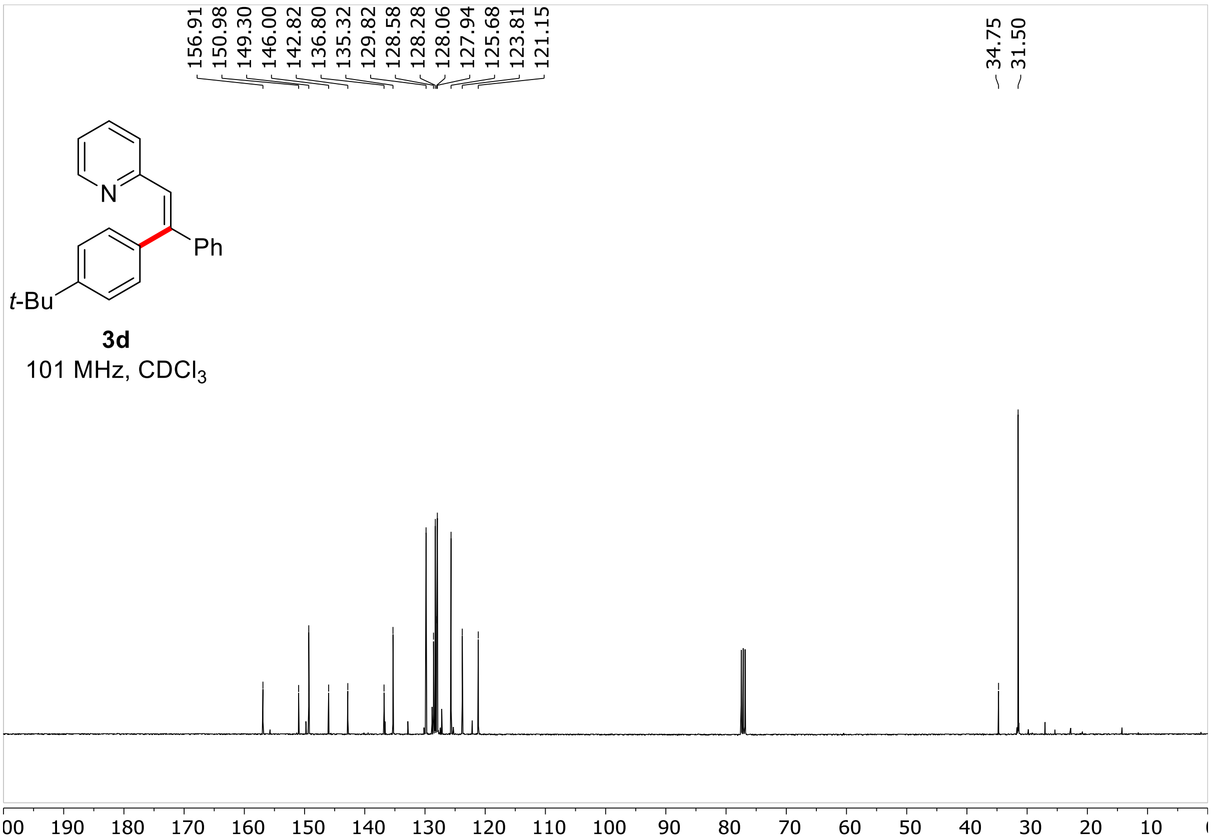

156.91 150.98 149.30 146.00 142.82 136.80 135.32 129.82 128.58 128.28 128.06 127.94 125.68 123.81 121.15
34.75
31.50
N
Ph
t-Bu
3d
101 MHz, CDCl3

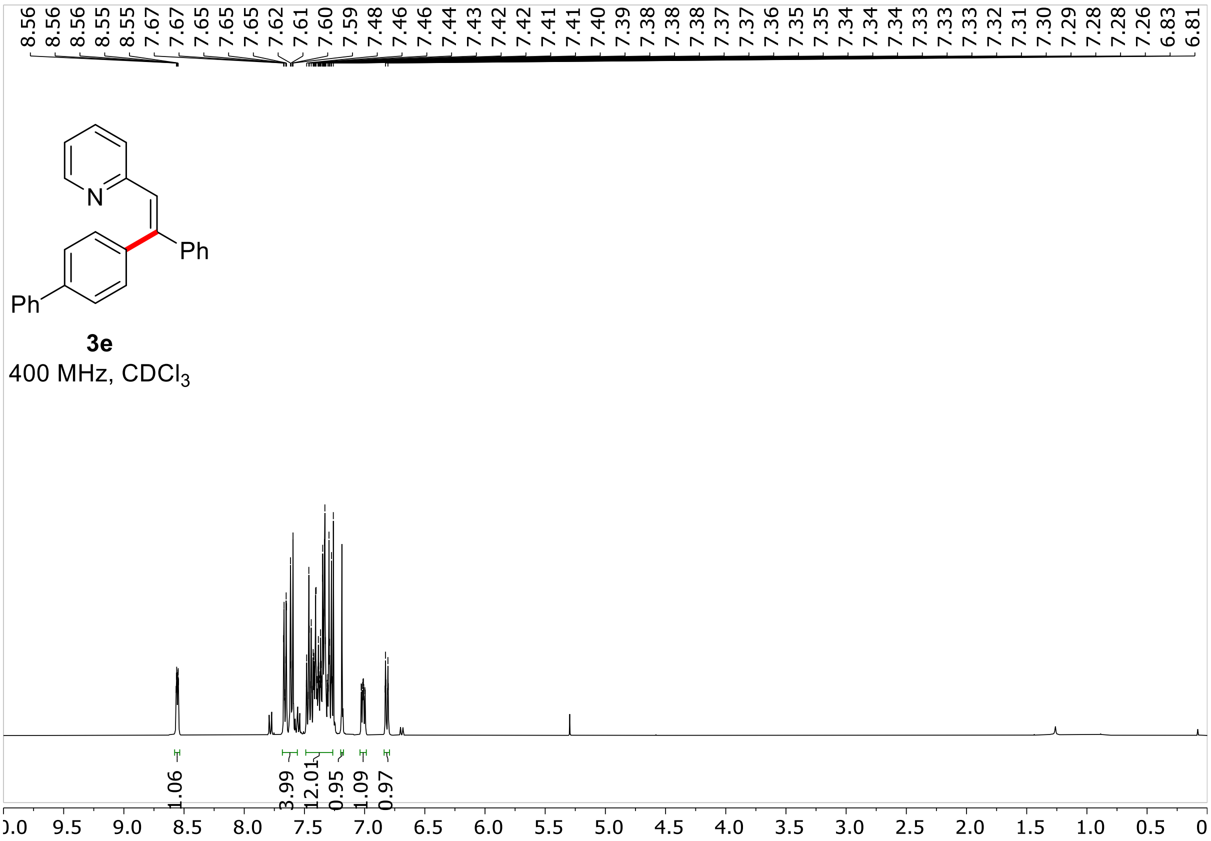
8.56 8.56 8.56 8.55 8.55 7.67 7.67 7.65 7.65 7.65 7.62 7.61 7.60 7.59 7.48 7.46 7.46 7.44 7.43 7.42 7.42 7.41 7.41 7.40 7.39 7.38 7.38 7.38 7.37 7.37 7.36 7.35 7.35 7.34 7.34 7.34 7.33 7.33 7.33 7.32 7.31 7.30 7.29 7.28 7.28 7.26 6.83 6.81
N
Ph
Ph
3e
400 MHz, CDCl3
1.06
3.99
12.01
0.95
1.09
0.97
9.5 9.0 8.5 8.0 7.5 7.0 6.5 6.0 5.5 5.0 4.5 4.0 3.5 3.0 2.5 2.0 1.5 1.0 0.5 0

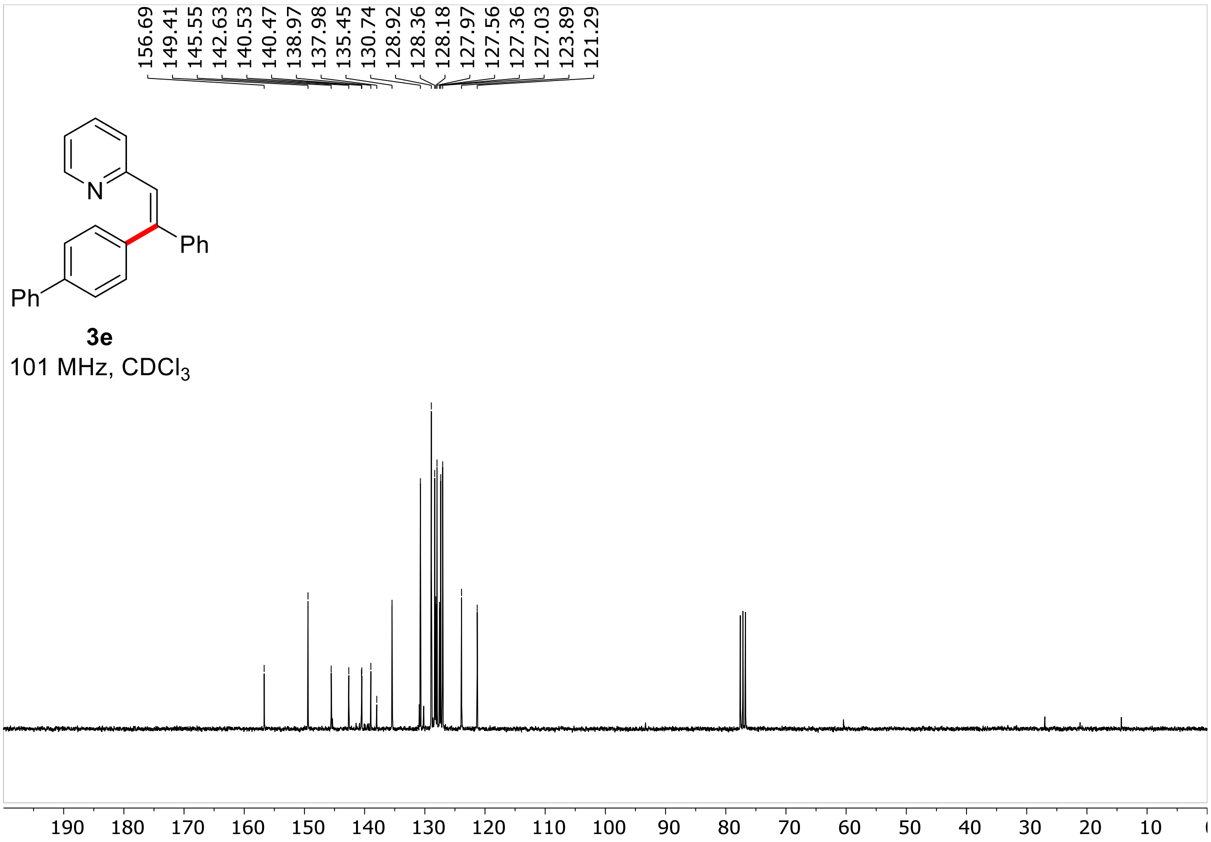
156.69 149.41 145.55 142.63 140.53 140.47 138.97 137.98 135.45 130.74 128.92 128.36 128.18 127.97 127.56 127.36 127.03 123.89 121.29
N
Ph
Ph
3e
101 MHz, CDCl3
190 180 170 160 150 140 130 120 110 100 90 80 70 60 50 40 30 20 10

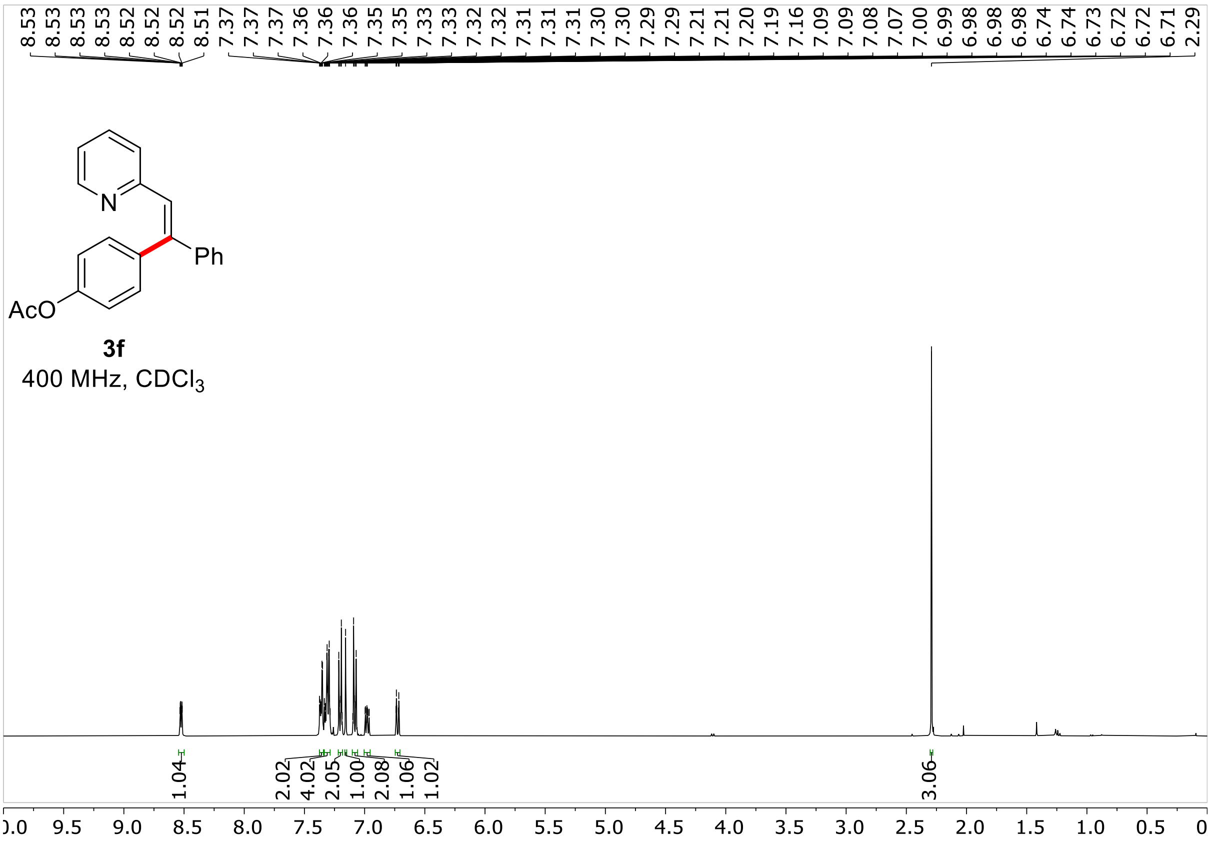
3f
400 MHz, CDCl3

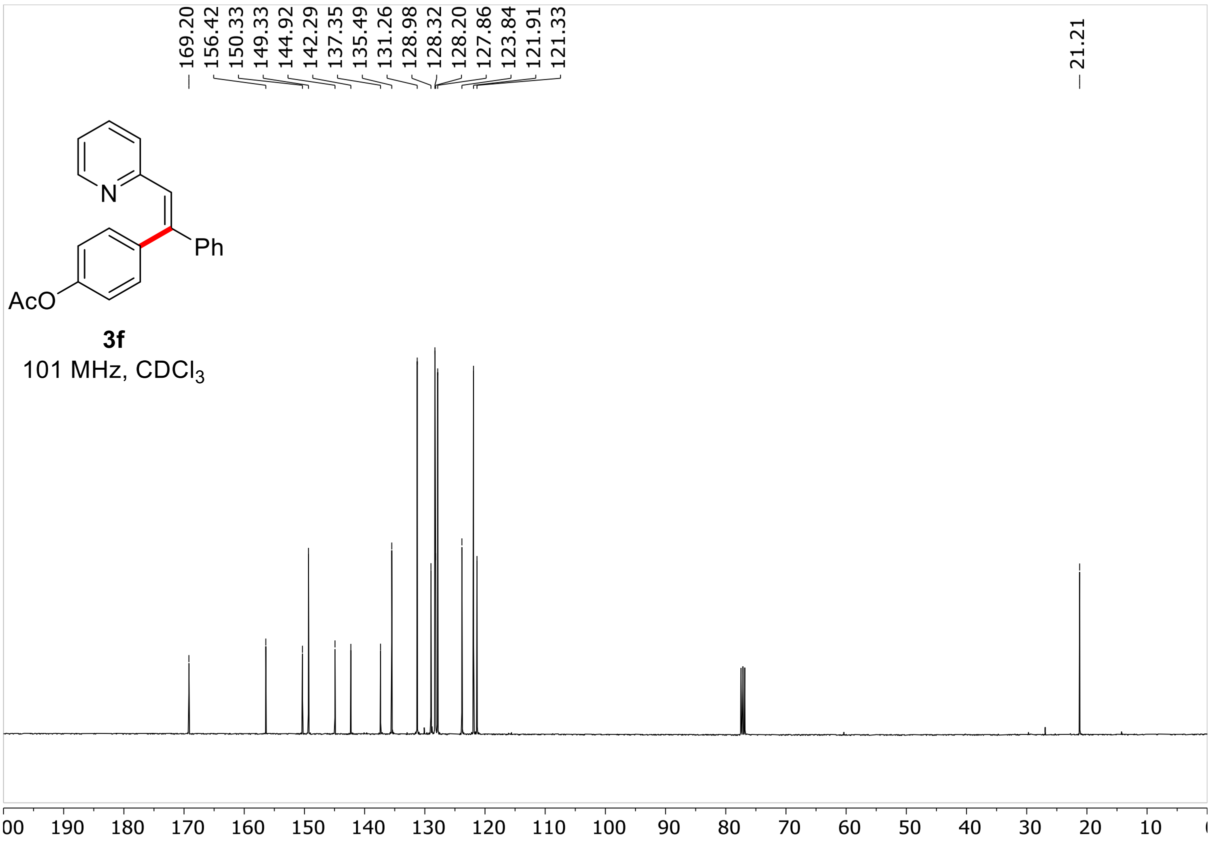
3f
101 MHz, CDCl3

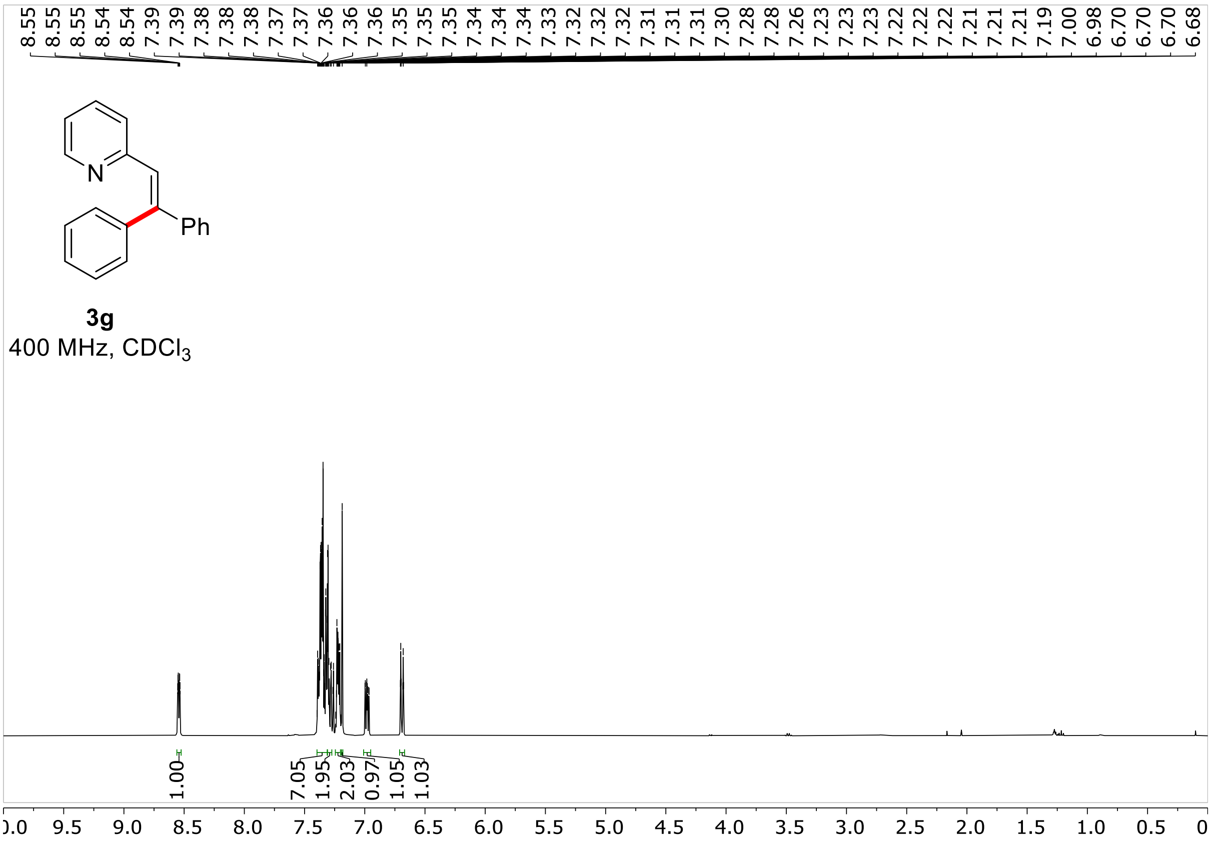
8.55
8.55
8.55
8.54
8.54
7.39
7.39
7.38
7.38
7.38
7.37
7.36
7.36
7.36
7.35
7.35
7.35
7.34
7.34
7.34
7.33
7.32
7.32
7.32
7.31
7.31
7.31
7.30
7.28
7.28
7.26
7.23
7.23
7.23
7.22
7.22
7.22
7.21
7.21
7.21
7.19
7.00
6.98
6.70
6.70
6.70
6.68
N
Ph
3g
400 MHz, $CDCl_3$
1.00
7.05
1.95
2.03
0.97
1.05
1.03

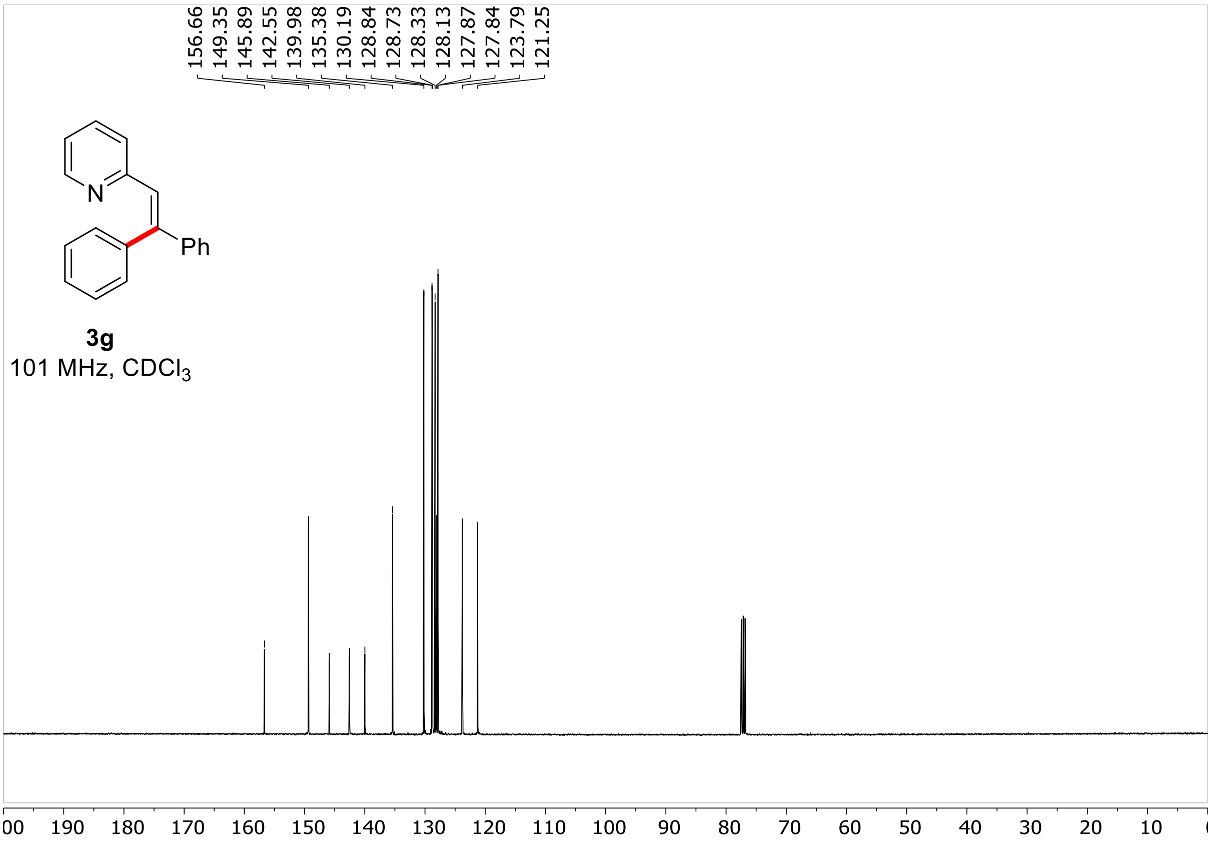
156.66
149.35
145.89
142.55
139.98
135.38
130.19
128.84
128.73
128.33
128.13
127.87
127.84
123.79
121.25
N
Ph
3g
101 MHz, $CDCl_3$

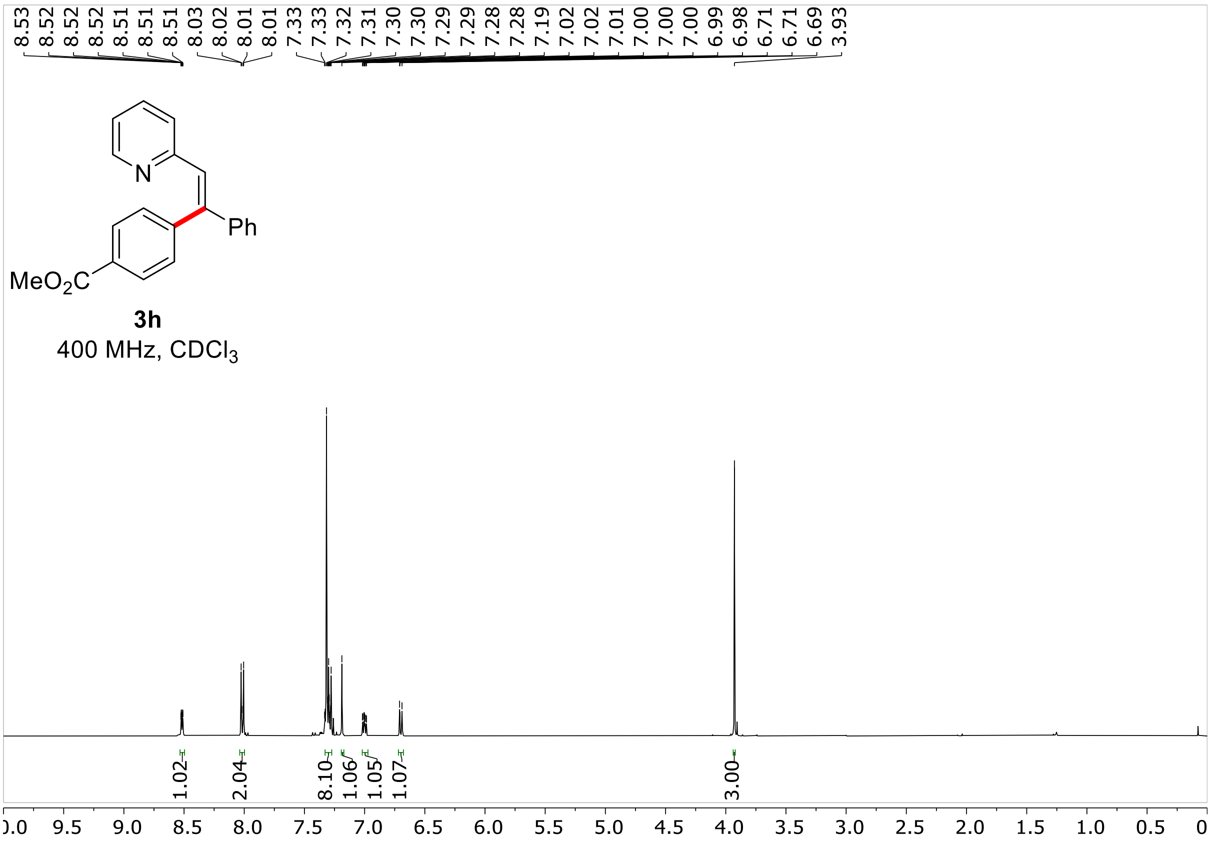
8.53 8.52 8.52 8.52 8.51 8.51 8.51 8.03 8.02 8.01 8.01 7.33 7.33 7.32 7.31 7.30 7.30 7.29 7.29 7.28 7.28 7.19 7.02 7.02 7.01 7.00 7.00 7.00 6.99 6.98 6.71 6.71 6.69 3.93
N
Ph
MeO2C
3h
400 MHz, CDCl3
1.02
2.04
8.10
1.06
1.05
1.07
3.00

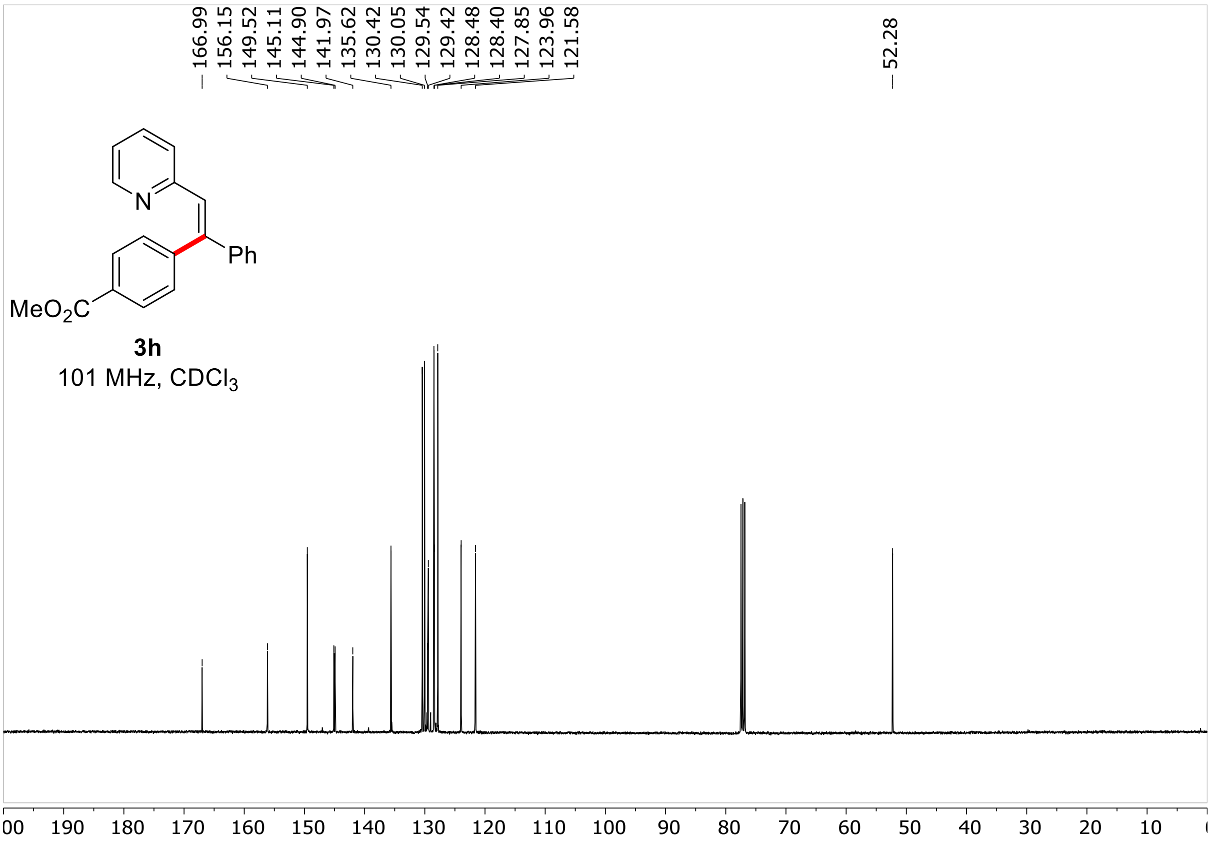
166.99 156.15 149.52 145.11 144.90 141.97 135.62 130.42 130.05 129.54 129.42 128.48 128.40 127.85 123.96 121.58
52.28
N
Ph
MeO2C
3h
101 MHz, CDCl3

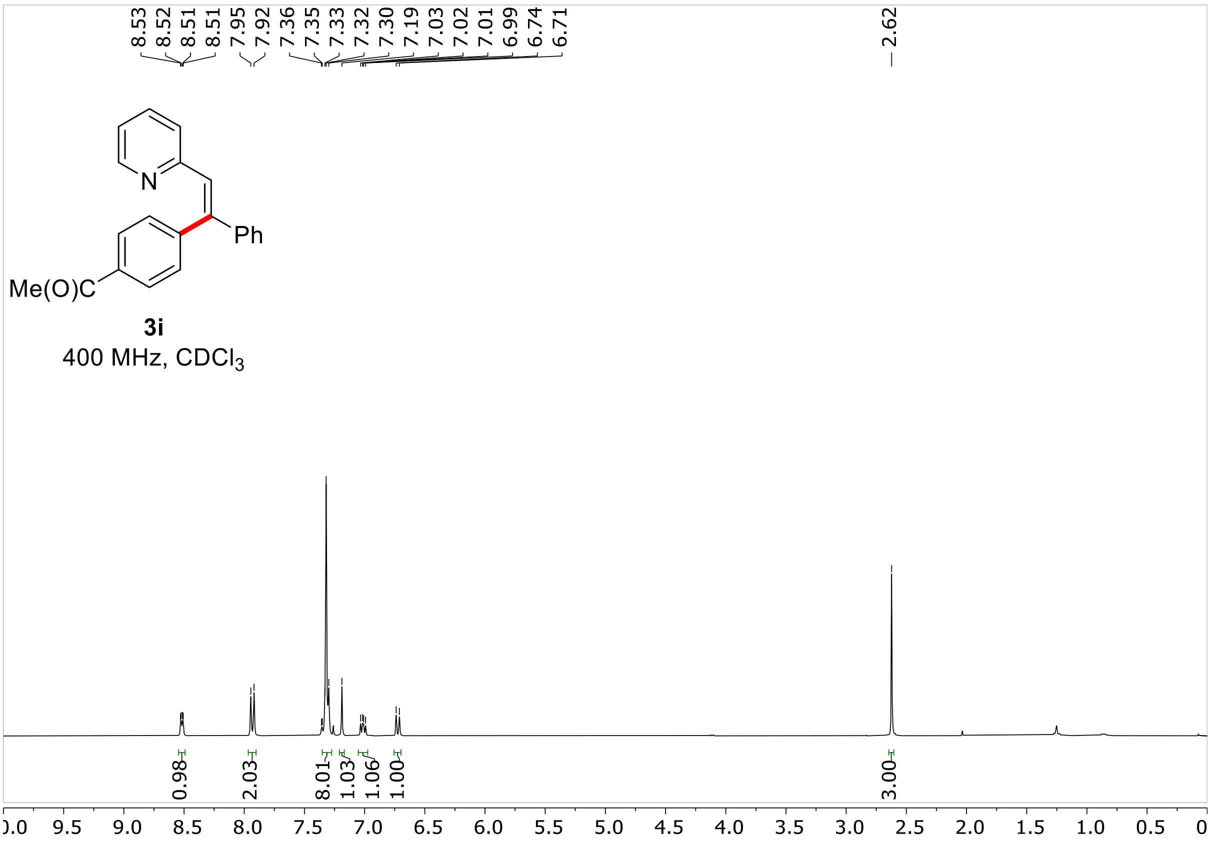
8.53
8.52
8.51
8.51
7.95
7.92
7.36
7.35
7.33
7.32
7.30
7.19
7.03
7.02
7.01
6.99
6.74
6.71
2.62
N
Ph
Me(O)C
3i
400 MHz, CDCl3
0.98
2.03
8.01
1.03
1.06
1.00
3.00

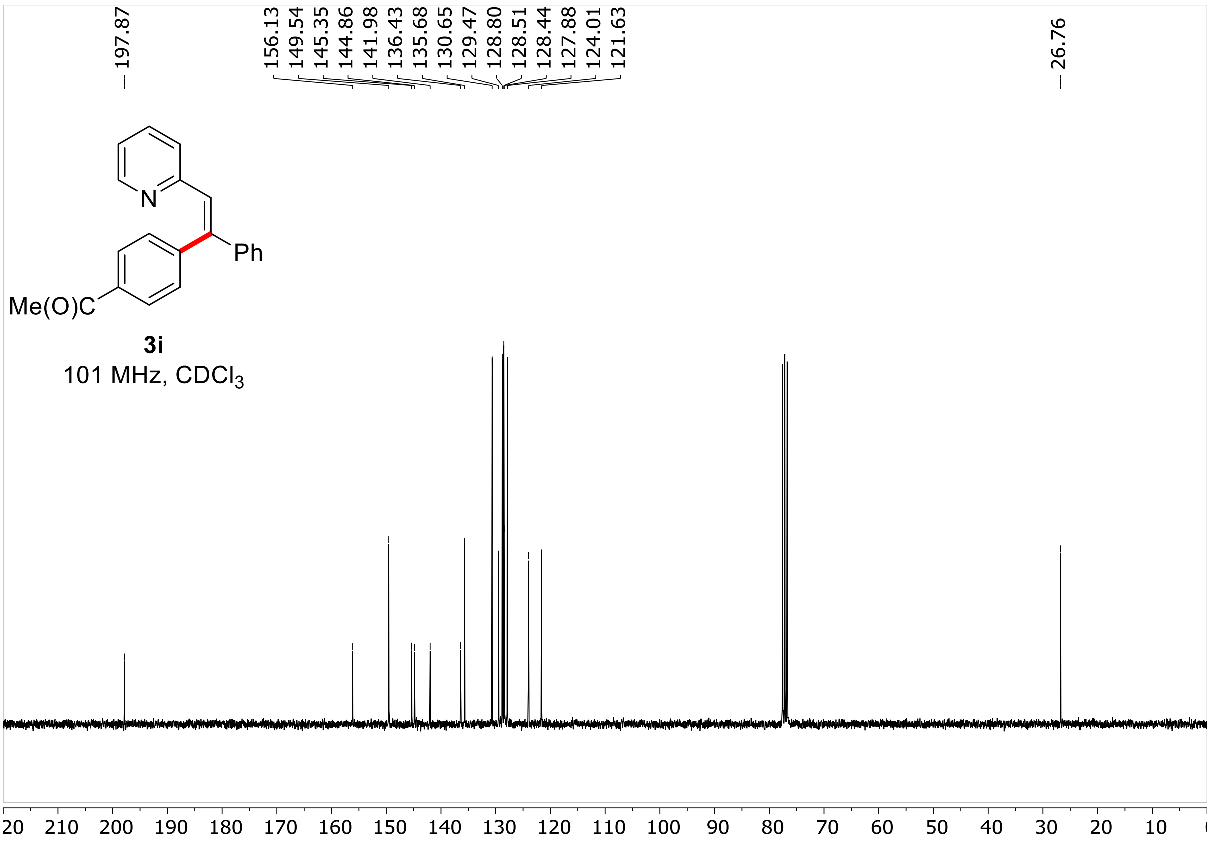
197.87
156.13
149.54
145.35
144.86
141.98
136.43
135.68
130.65
129.47
128.80
128.51
128.44
127.88
124.01
121.63
26.76
N
Ph
Me(O)C
3i
101 MHz, CDCl3

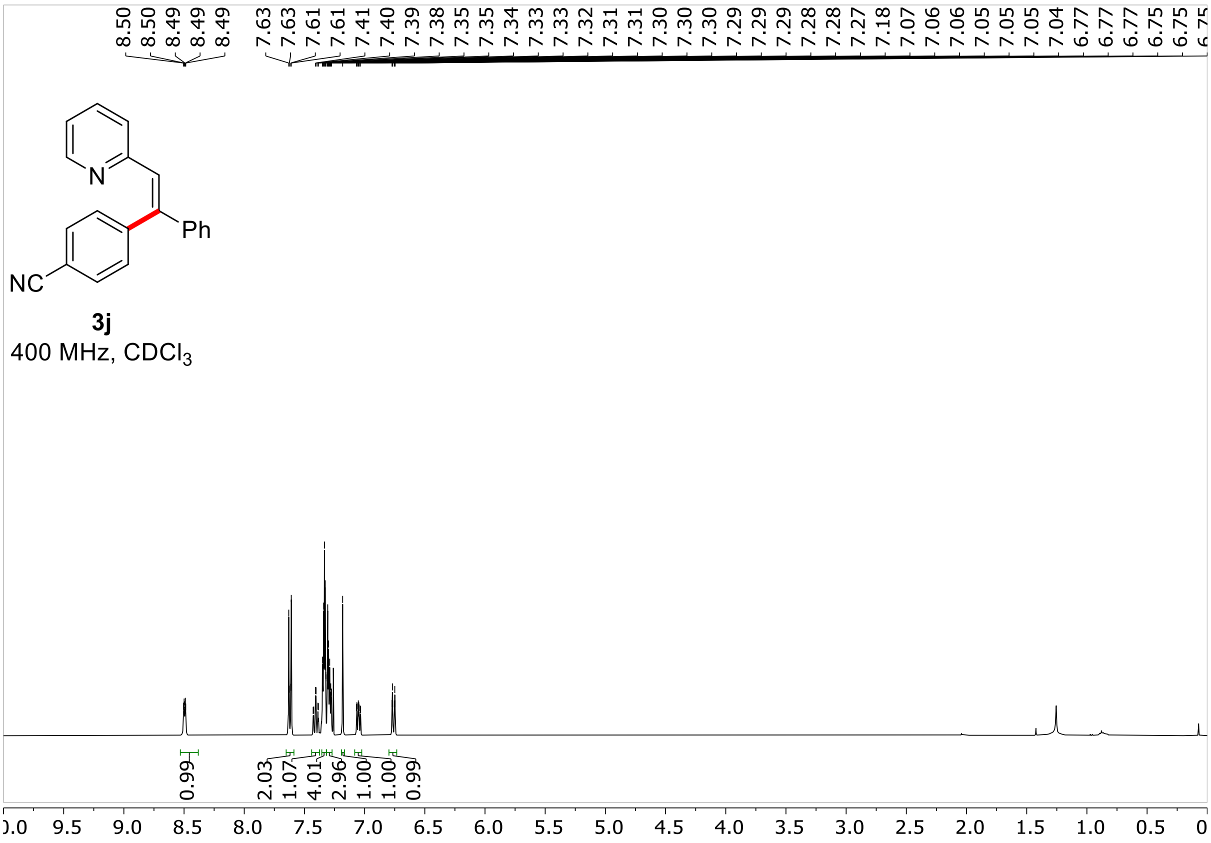
8.50 8.50 8.49 8.49 8.49 7.63 7.63 7.61 7.61 7.41 7.40 7.39 7.38 7.35 7.35 7.34 7.33 7.33 7.32 7.31 7.31 7.30 7.30 7.30 7.29 7.29 7.29 7.28 7.28 7.27 7.18 7.07 7.06 7.06 7.05 7.05 7.05 7.04 6.77 6.77 6.77 6.75 6.75
N
Ph
NC
3j
400 MHz, $CDCl_3$
0.99 2.03 1.07 4.01 2.96 1.00 1.00 0.99

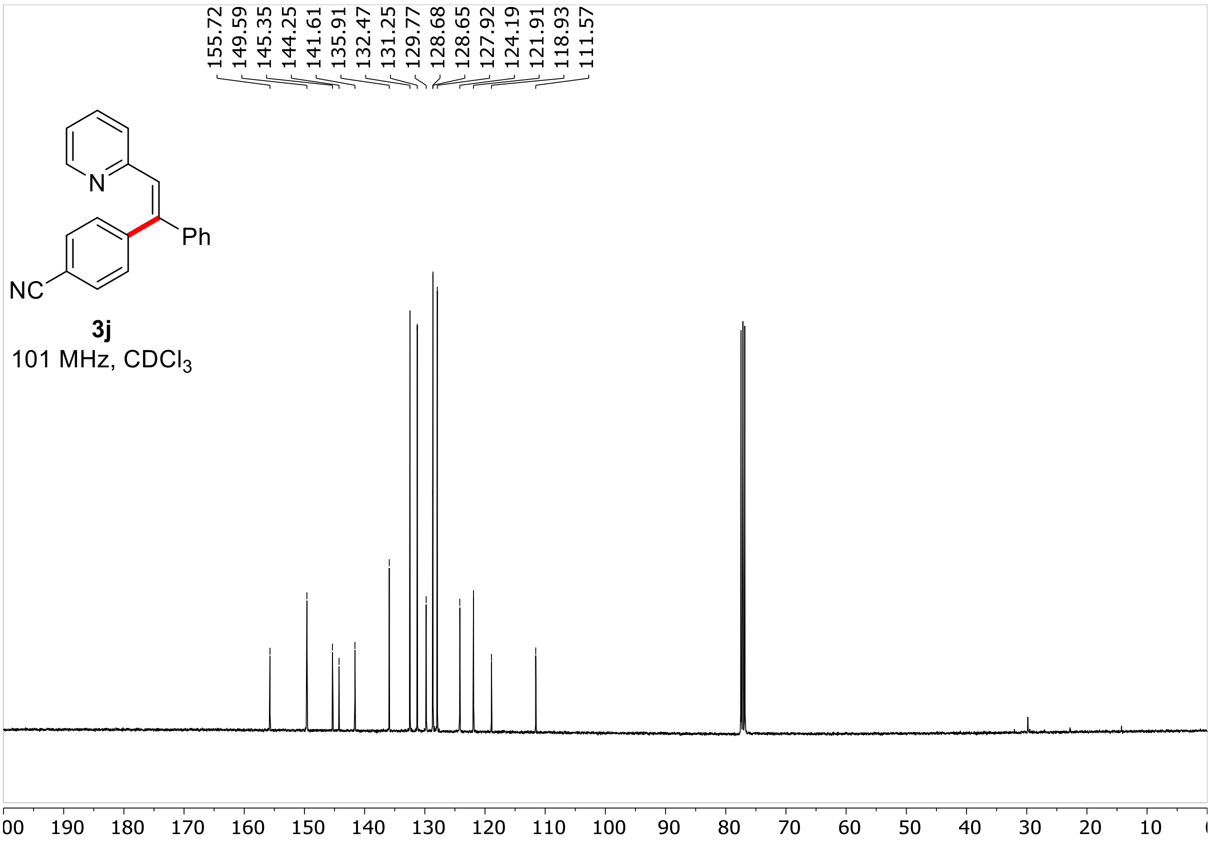
155.72 149.59 145.35 144.25 141.61 135.91 132.47 131.25 129.77 128.68 128.65 127.92 124.19 121.91 118.93 111.57
N
Ph
NC
3j
101 MHz, $CDCl_3$

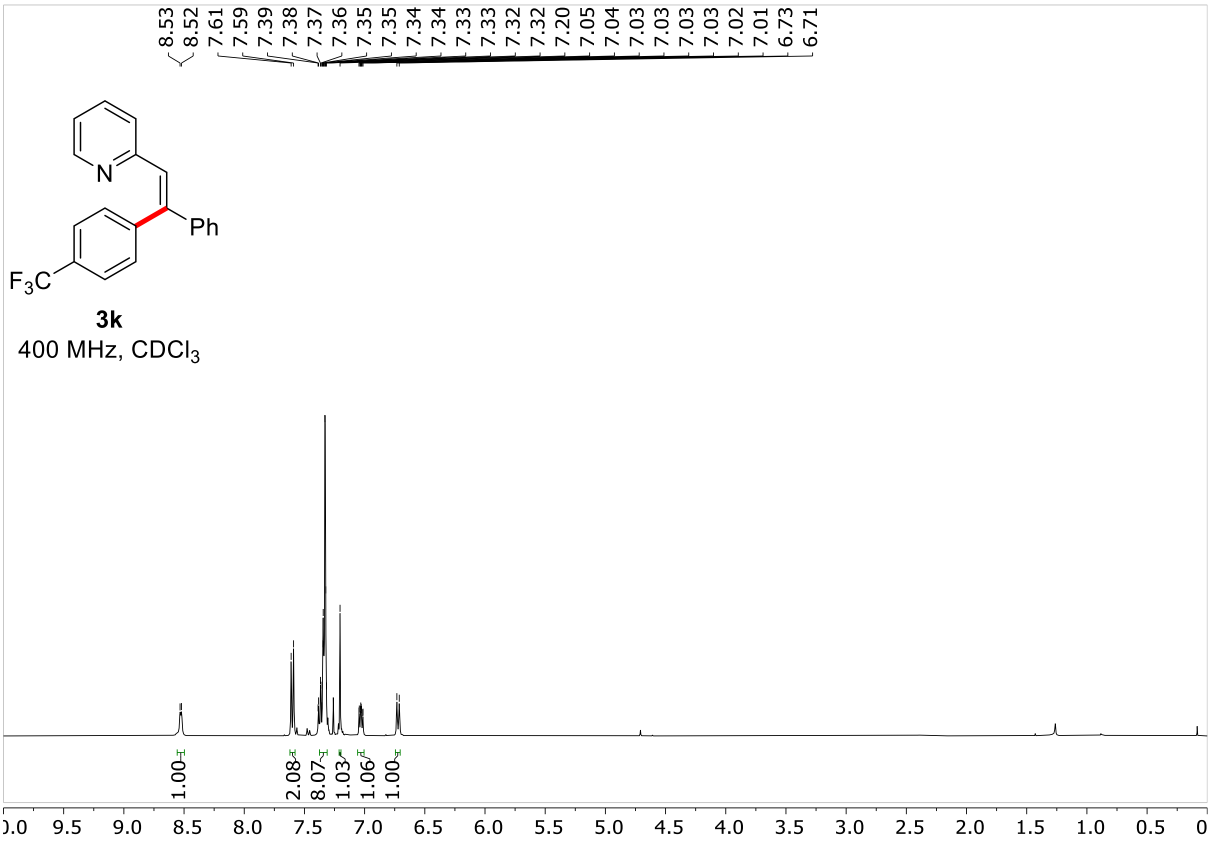
8.53
8.52
7.61
7.59
7.39
7.38
7.37
7.36
7.35
7.35
7.34
7.34
7.33
7.33
7.32
7.32
7.20
7.05
7.04
7.03
7.03
7.03
7.02
7.01
6.73
6.71
N
Ph
F3C
3k
400 MHz, CDCl3
1.00
2.08
8.07
1.03
1.06
1.00

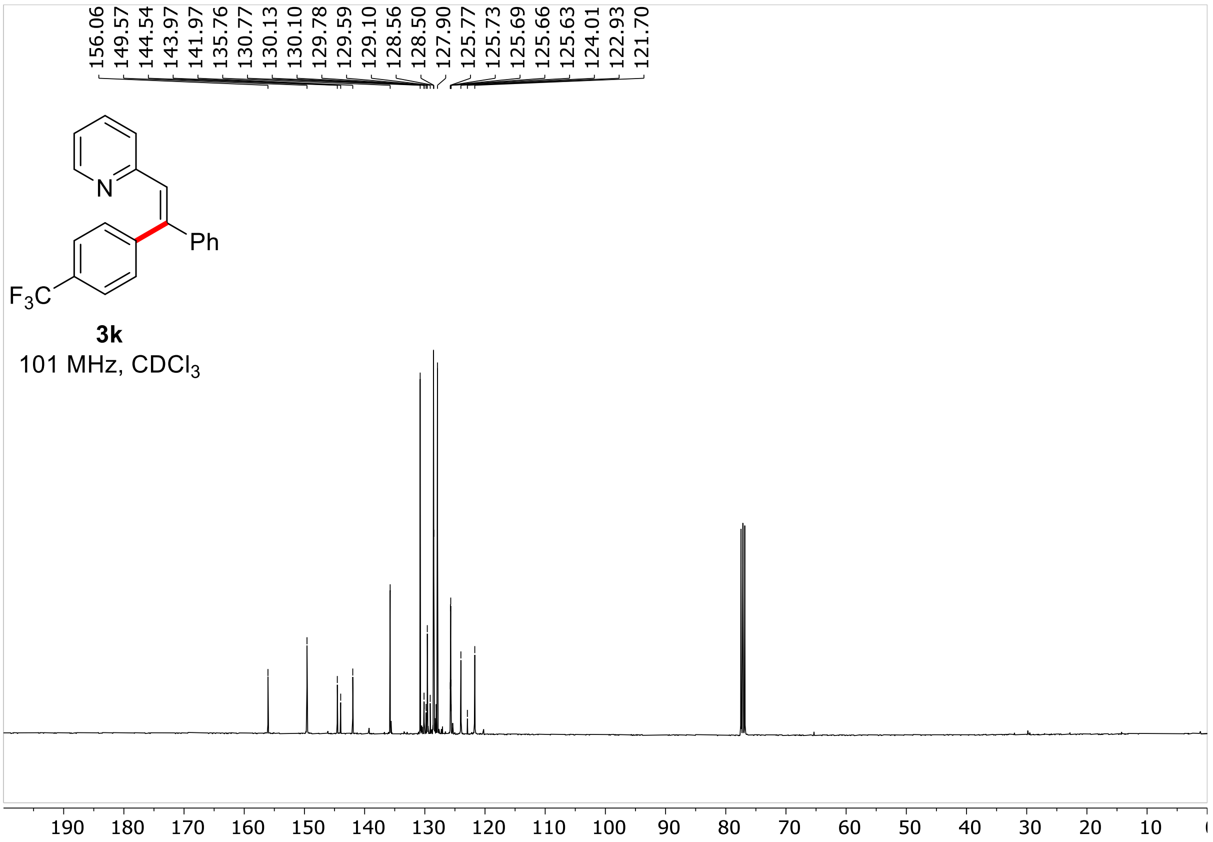
156.06
149.57
144.54
143.97
141.97
135.76
130.77
130.13
130.10
129.78
129.59
129.10
128.56
128.50
127.90
125.77
125.73
125.69
125.66
125.63
124.01
122.93
121.70
N
Ph
F3C
3k
101 MHz, CDCl3

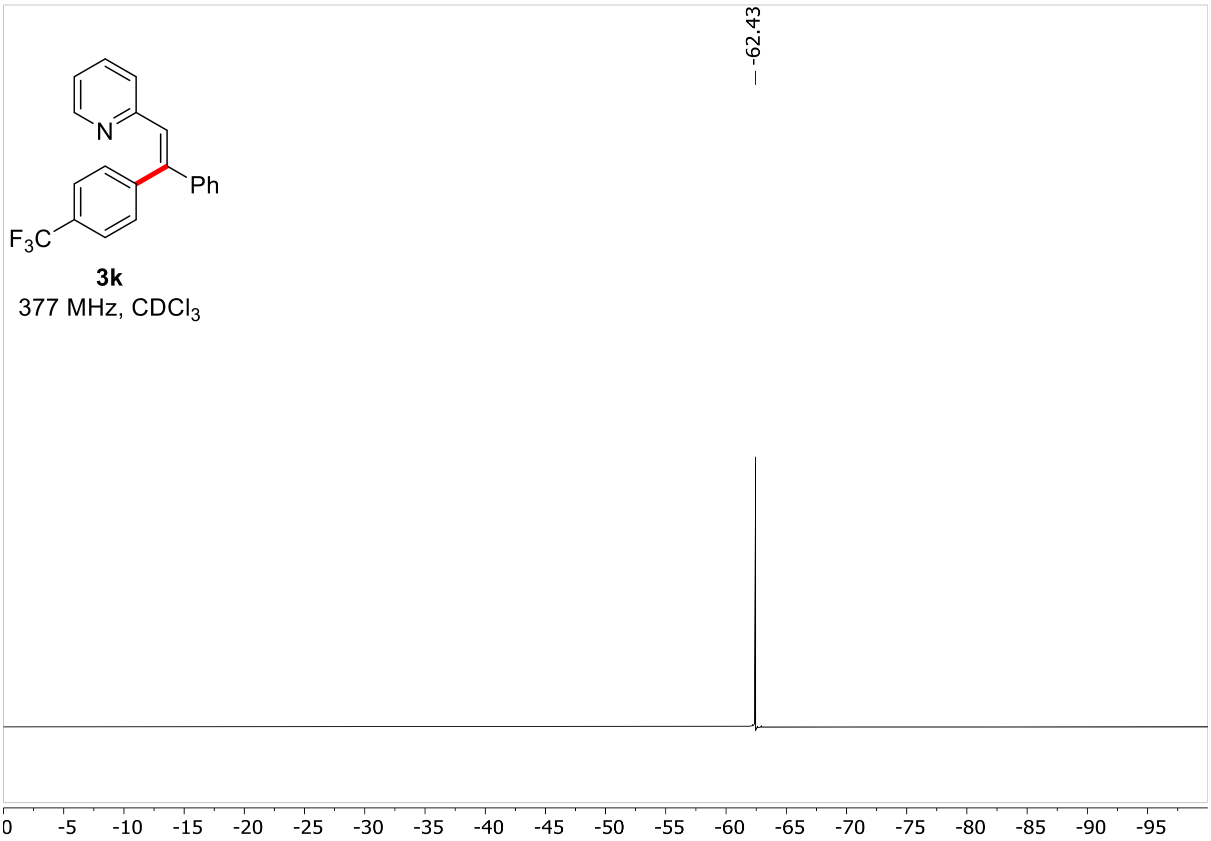

3k
377 MHz, $CDCl_3$

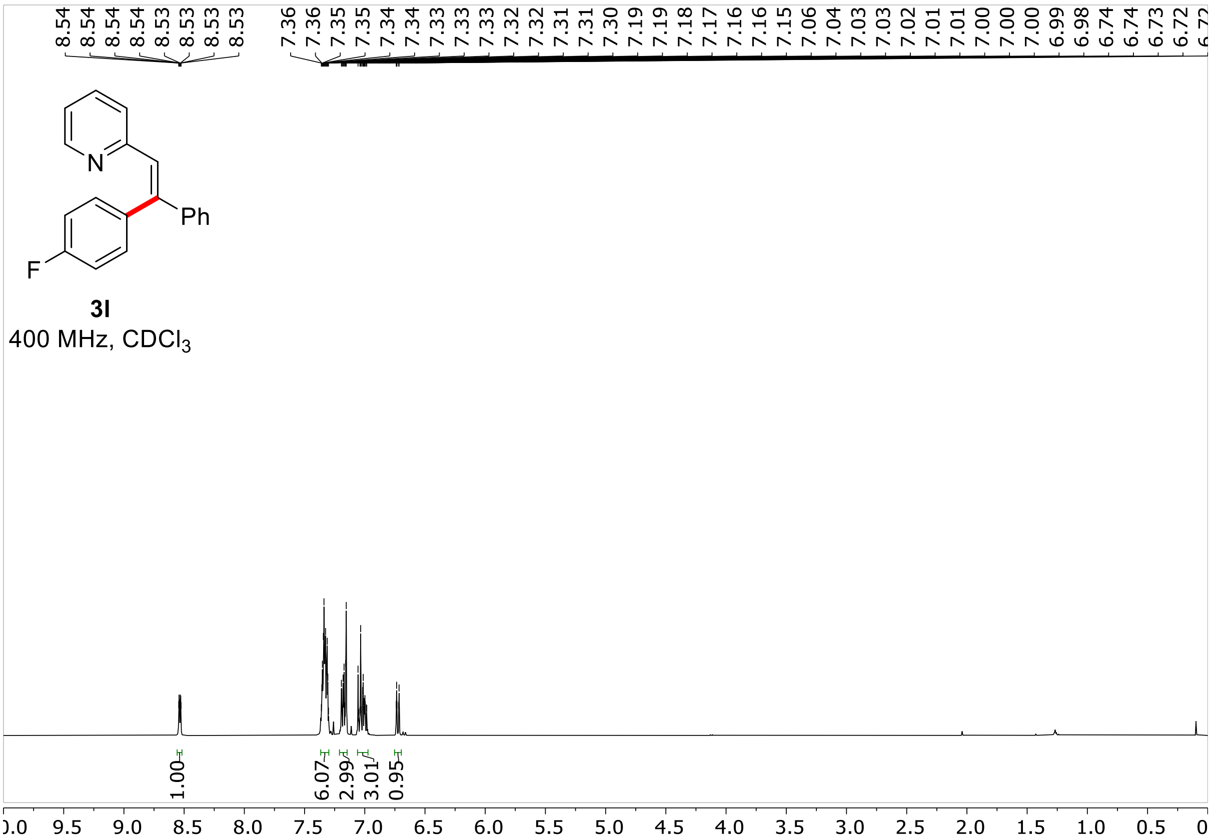
8.54 8.54 8.54 8.54 8.53 8.53 8.53 8.53
7.36 7.36 7.35 7.35 7.34 7.34 7.33 7.33 7.33 7.32 7.32 7.31 7.31 7.30 7.19 7.19 7.18 7.17 7.16 7.16 7.15 7.06 7.04 7.03 7.03 7.02 7.01 7.01 7.00 7.00 7.00 6.99 6.98 6.74 6.74 6.73 6.72
N
Ph
F
3l
400 MHz, CDCl$_3$
1.00
6.07
2.99
3.01
0.95

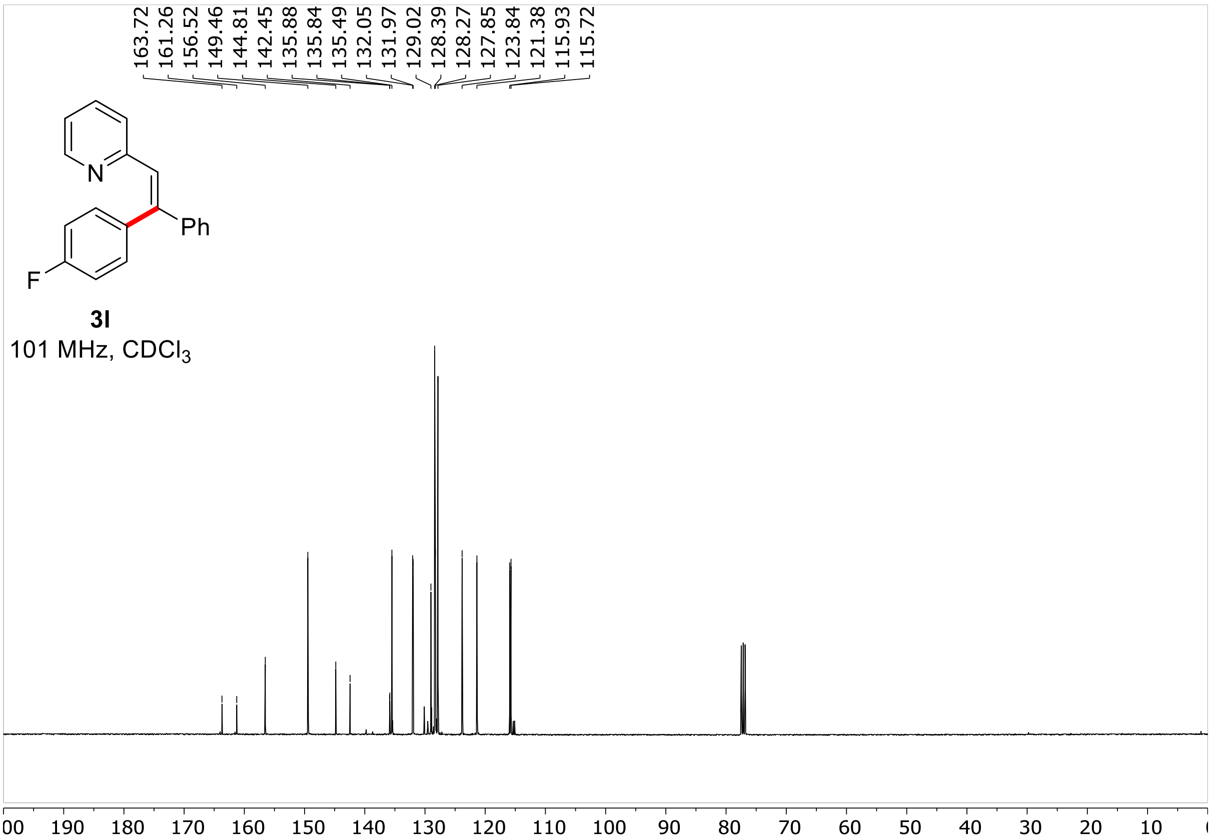
163.72 161.26 156.52 149.46 144.81 142.45 135.88 135.84 135.49 132.05 131.97 129.02 128.39 128.27 127.85 123.84 121.38 115.93 115.72
N
Ph
F
3l
101 MHz, CDCl$_3$

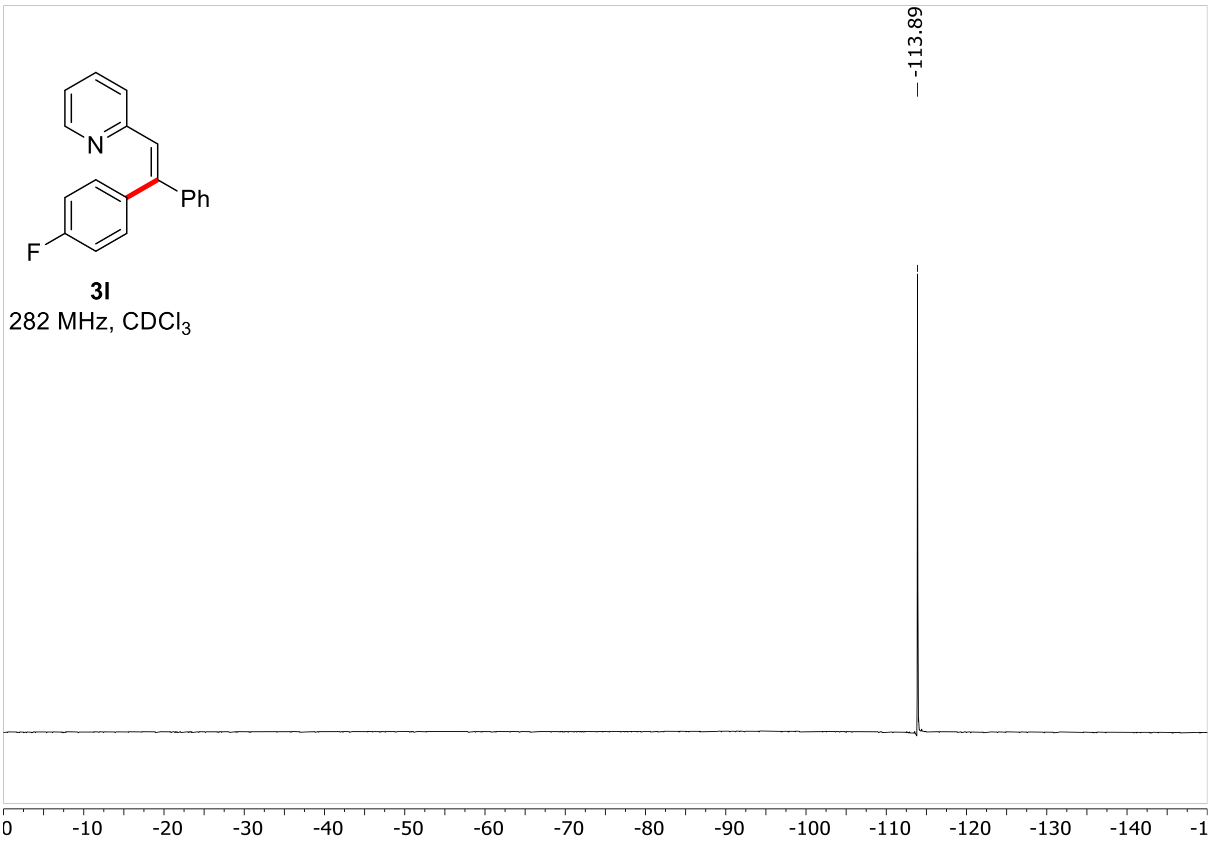
-113.89
N
Ph
F
3l
282 MHz, CDCl3
0
-10
-20
-30
-40
-50
-60
-70
-80
-90
-100
-110
-120
-130
-140
-1

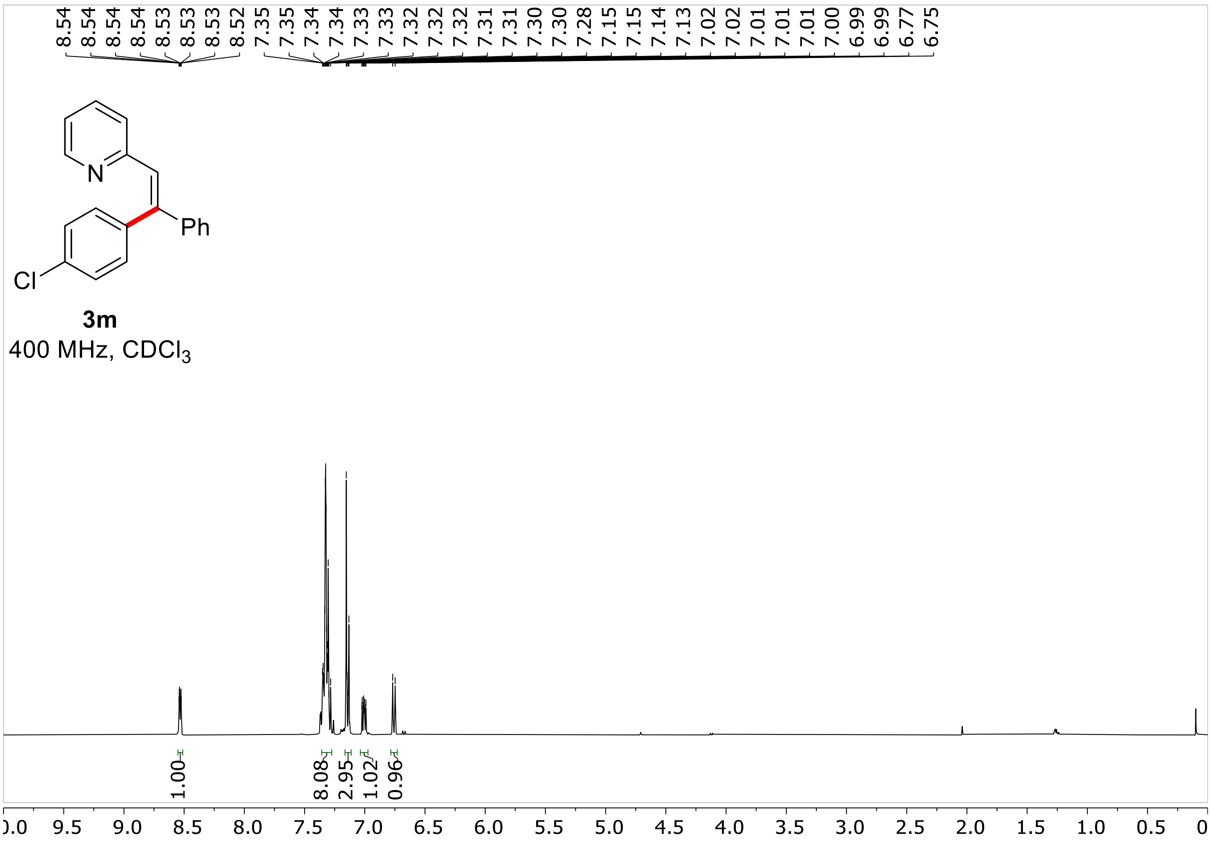
8.54 8.54 8.54 8.54 8.53 8.53 8.53 8.52 7.35 7.35 7.34 7.34 7.33 7.33 7.32 7.32 7.32 7.31 7.31 7.30 7.30 7.28 7.15 7.15 7.14 7.13 7.02 7.02 7.01 7.01 7.01 7.00 6.99 6.99 6.77 6.75
N
Ph
Cl
3m
400 MHz, CDCl3
1.00
8.08
2.95
1.02
0.96

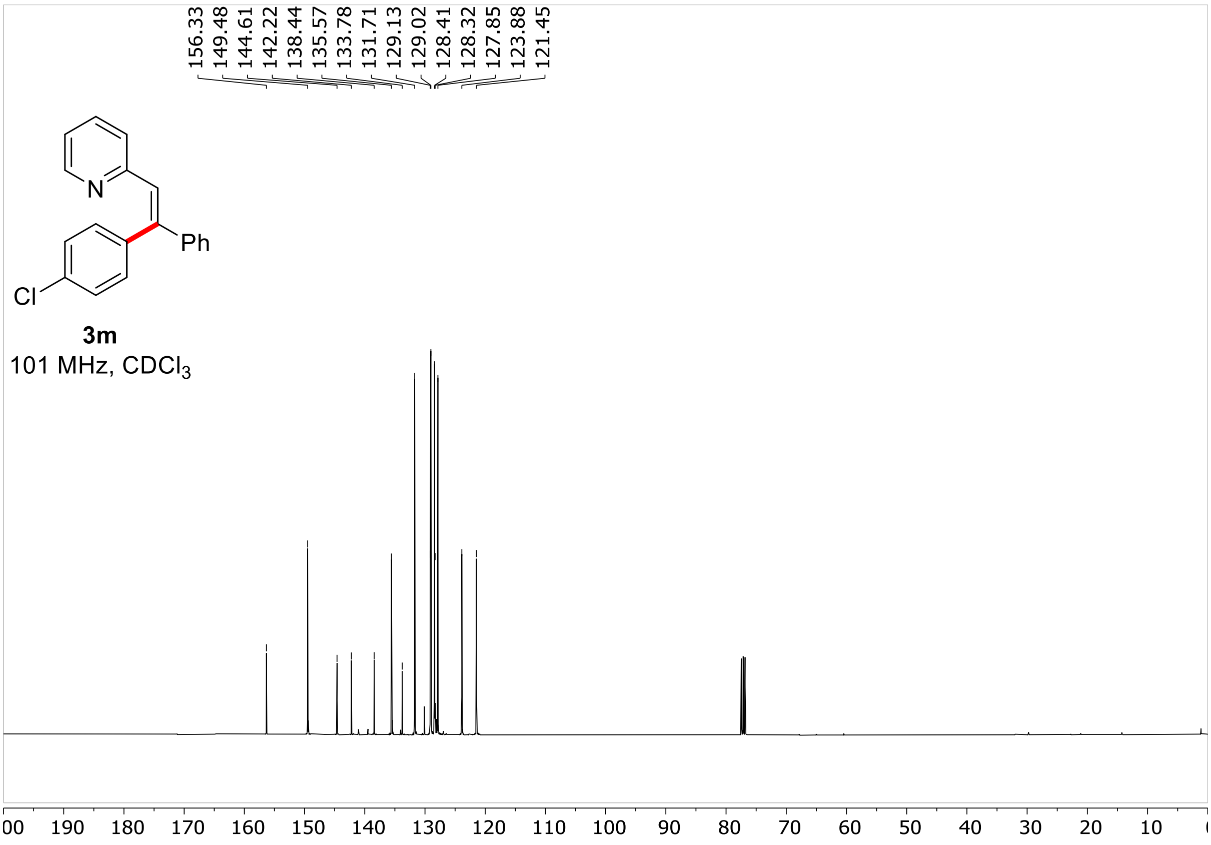
156.33 149.48 144.61 142.22 138.44 135.57 133.78 131.71 129.13 129.02 128.41 128.32 127.85 123.88 121.45
N
Ph
Cl
3m
101 MHz, CDCl3

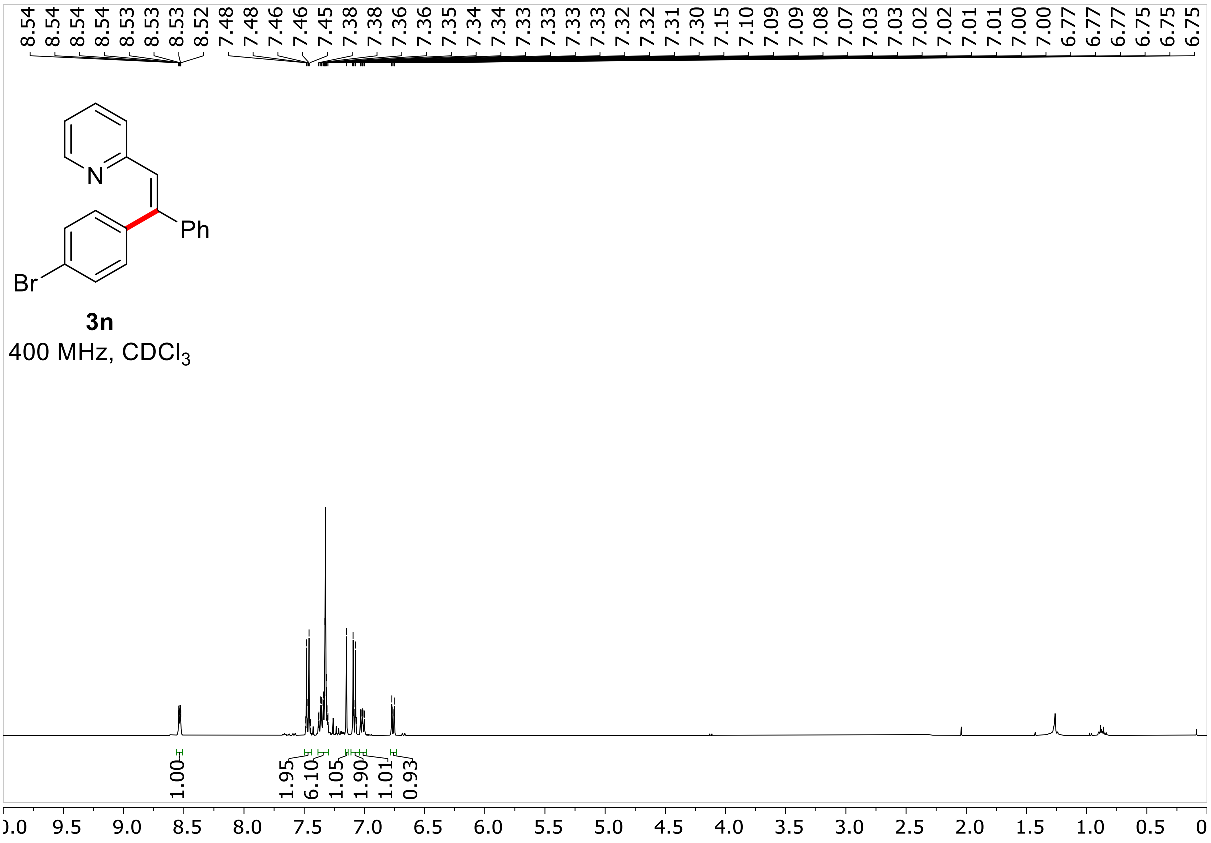
3n
400 MHz, CDCl3

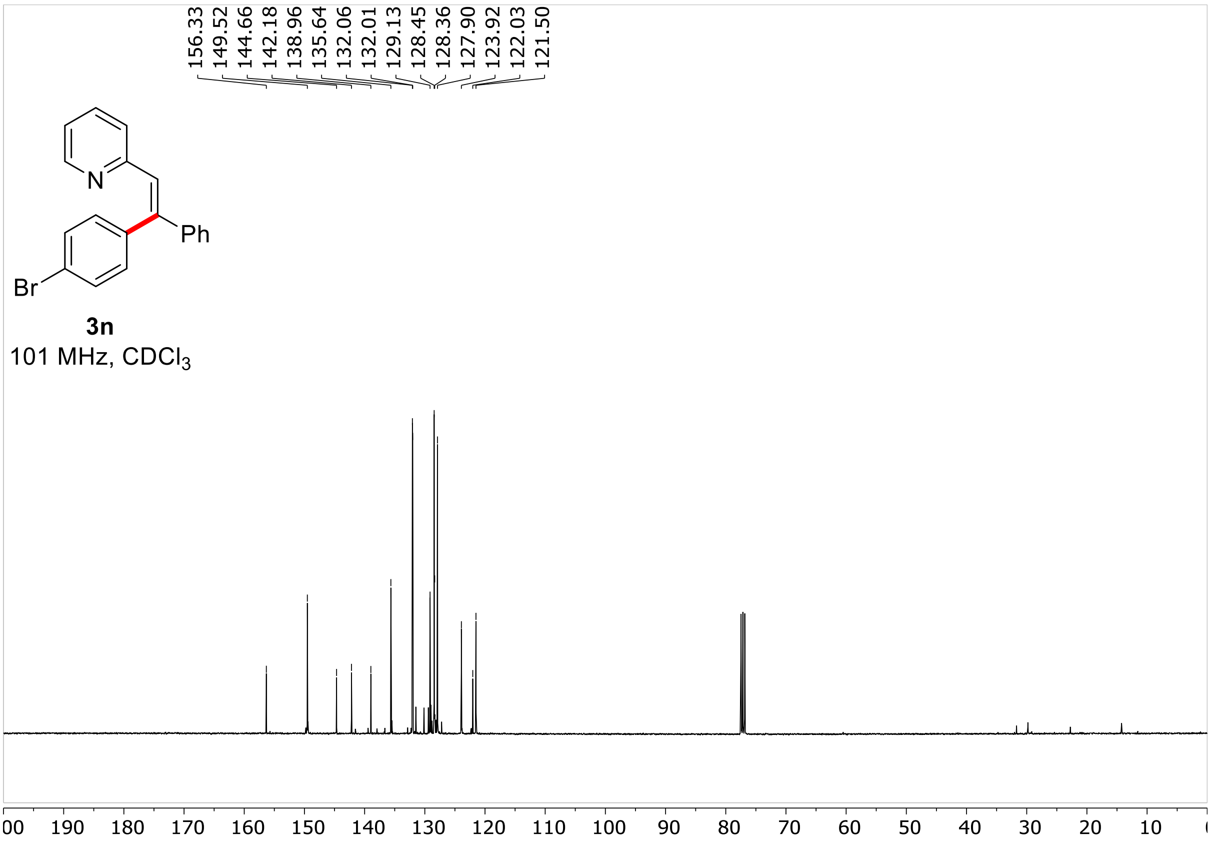
3n
101 MHz, CDCl3

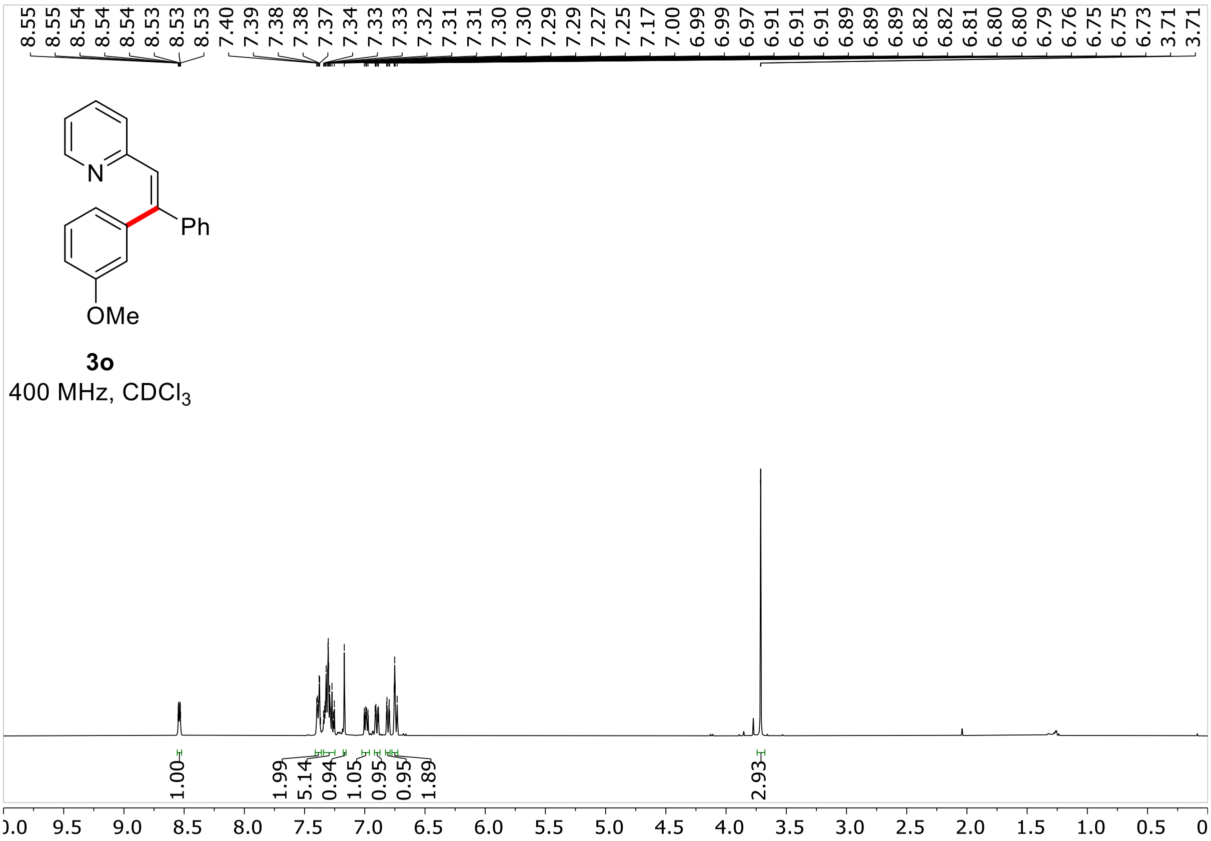

3o
400 MHz, $CDCl_3$

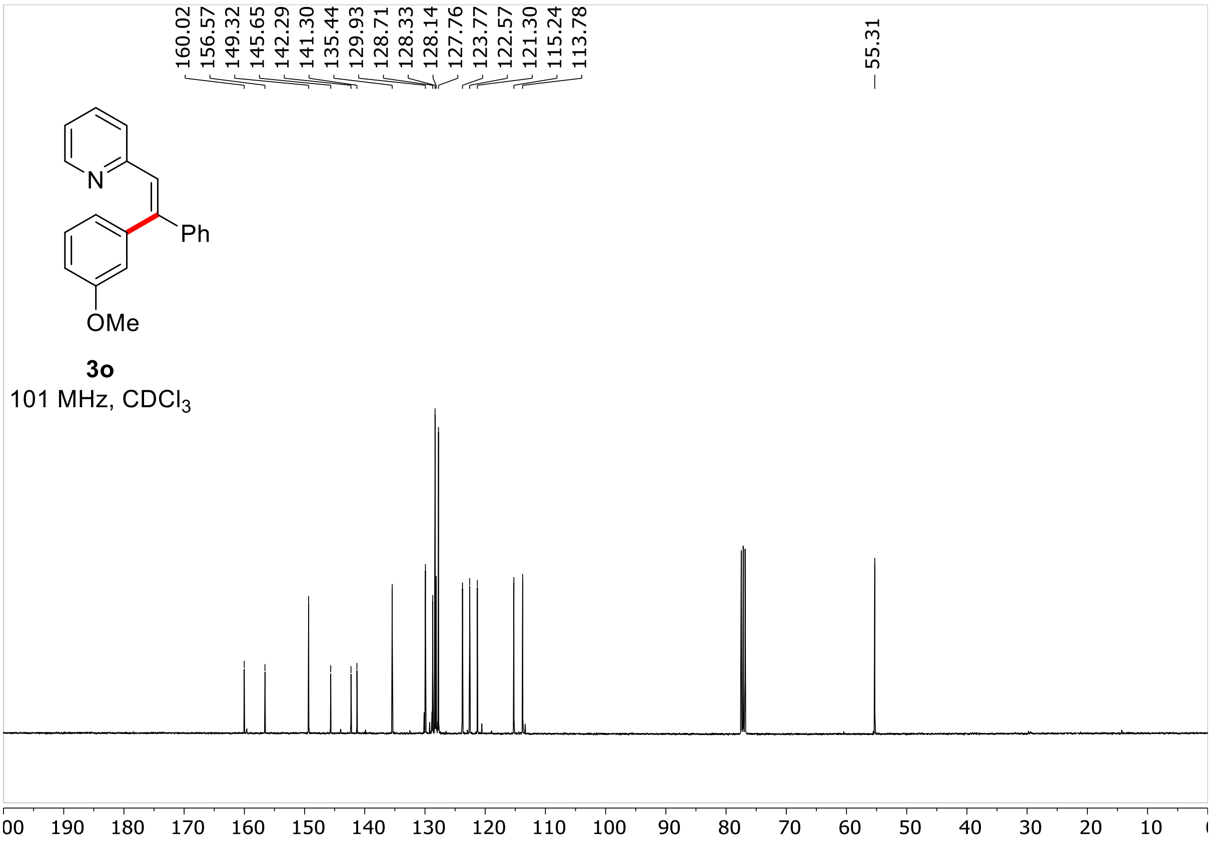

3o
101 MHz, $CDCl_3$

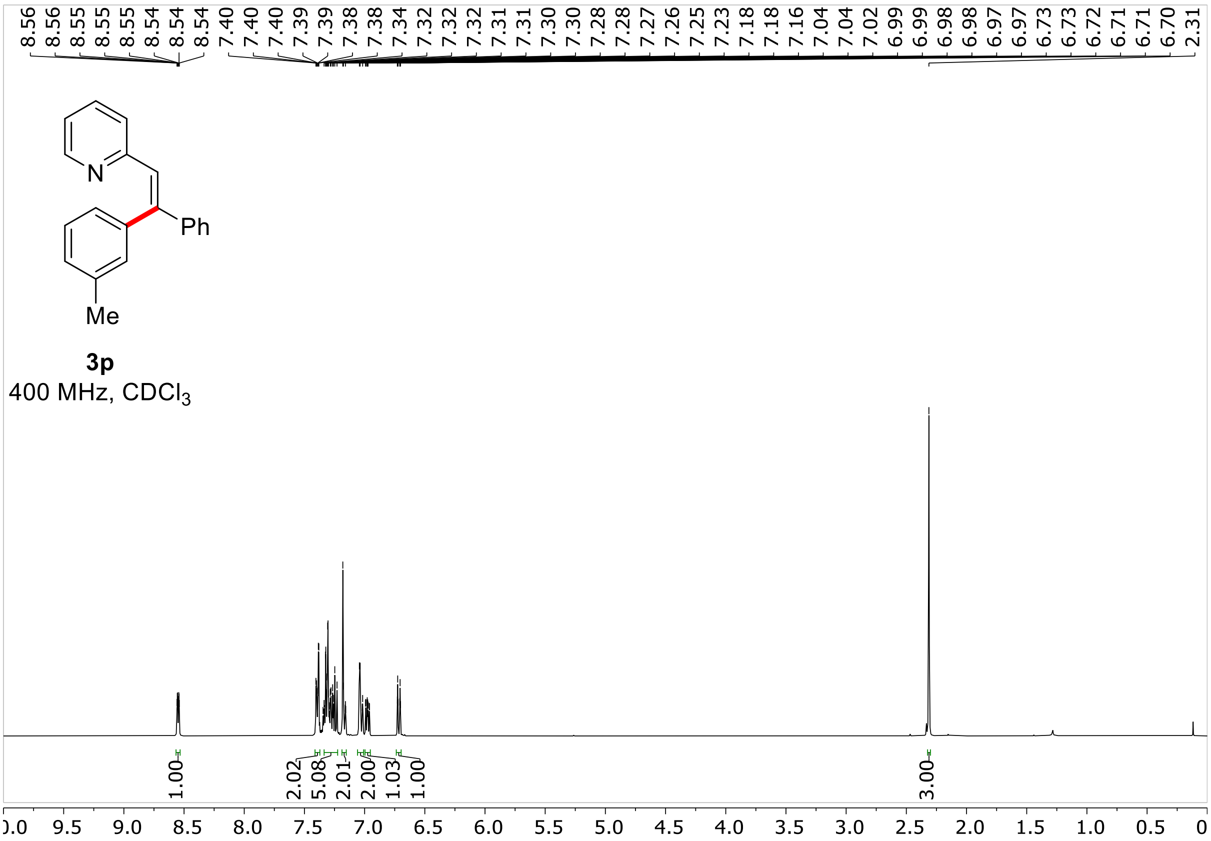
8.56 8.56 8.55 8.55 8.55 8.54 8.54 8.54 7.40 7.40 7.39 7.39 7.38 7.38 7.34 7.32 7.32 7.32 7.31 7.31 7.30 7.30 7.28 7.28 7.27 7.26 7.25 7.23 7.18 7.18 7.16 7.04 7.04 7.02 6.99 6.99 6.98 6.98 6.97 6.97 6.73 6.73 6.72 6.71 6.71 6.70 2.31
N
Ph
Me
3p
400 MHz, $CDCl_3$
1.00
2.02
5.08
2.01
2.00
1.03
1.00
3.00

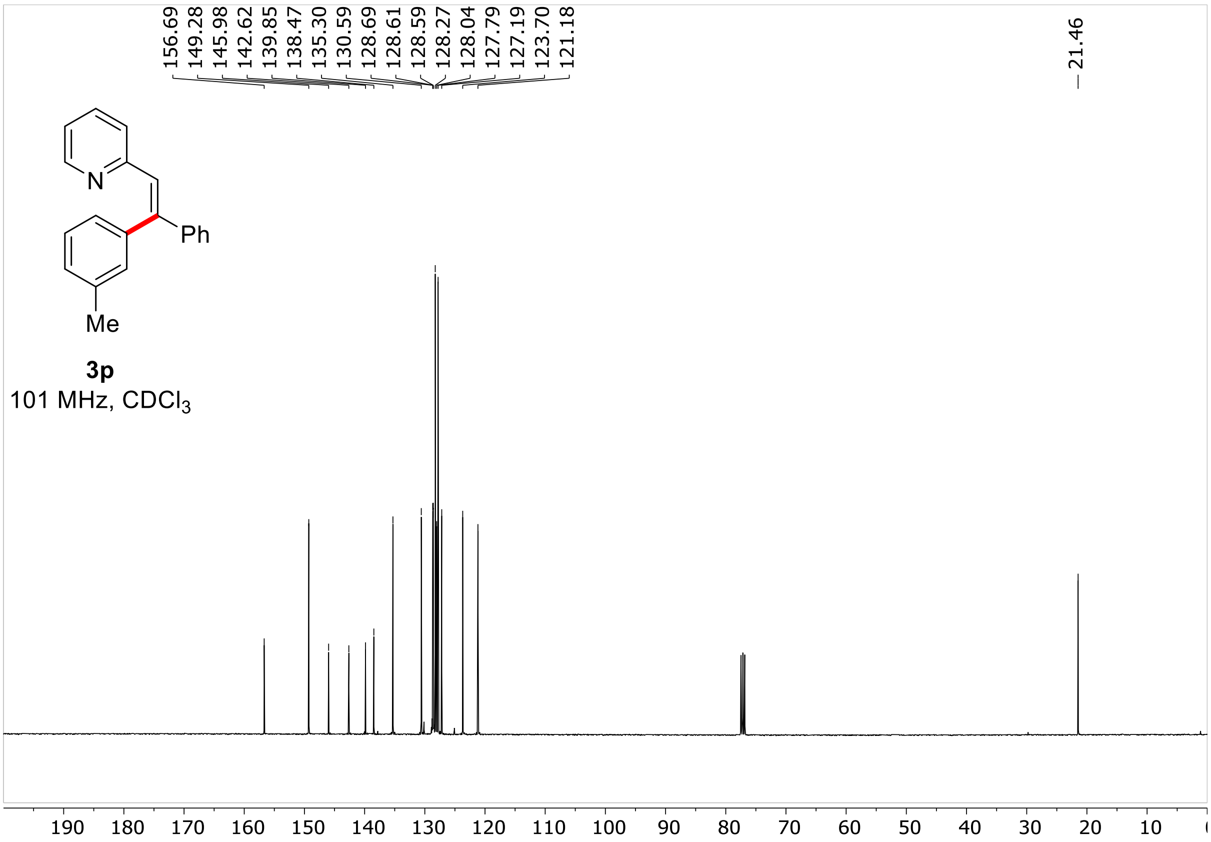
156.69 149.28 145.98 142.62 139.85 138.47 135.30 130.59 128.69 128.61 128.59 128.27 128.04 127.79 127.19 123.70 121.18
21.46
N
Ph
Me
3p
101 MHz, $CDCl_3$

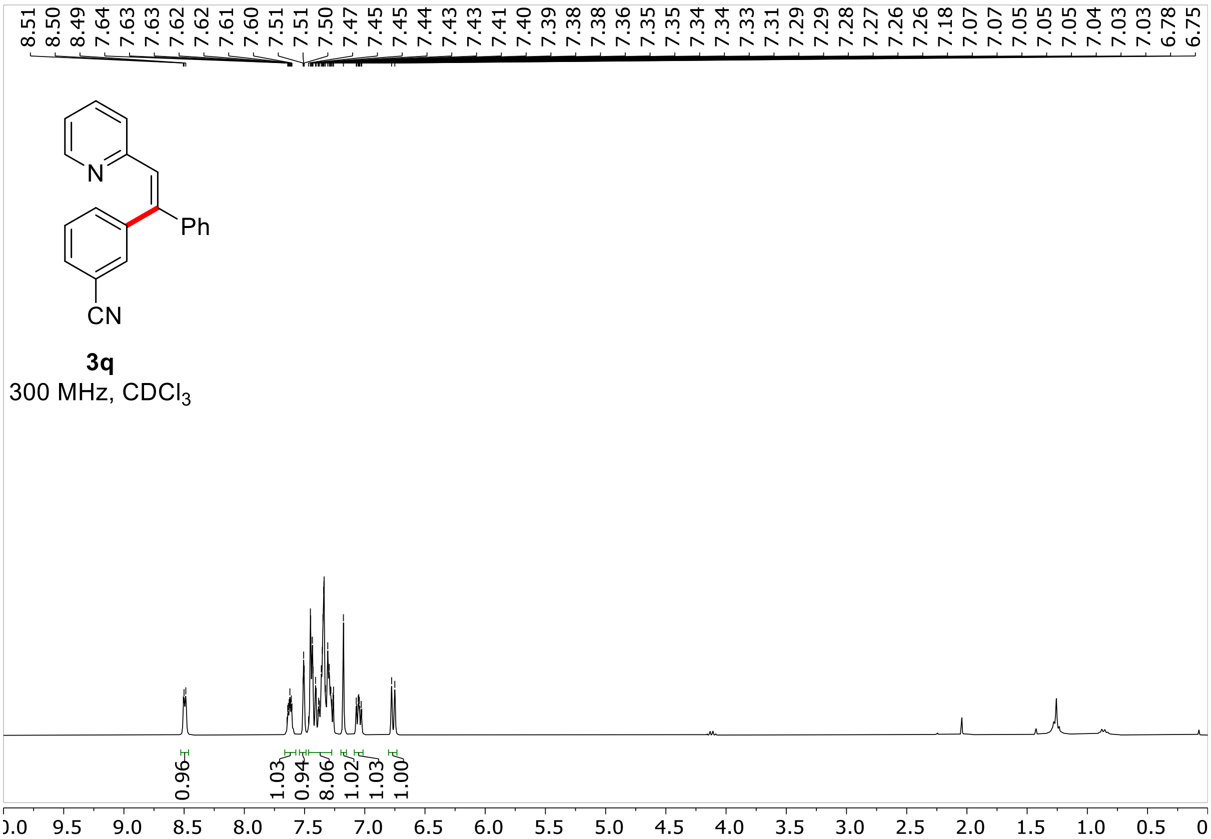
8.51 8.50 8.49 7.64 7.63 7.63 7.62 7.62 7.61 7.60 7.51 7.51 7.50 7.47 7.45 7.45 7.44 7.43 7.43 7.41 7.40 7.39 7.38 7.38 7.36 7.35 7.35 7.34 7.34 7.33 7.31 7.29 7.29 7.28 7.27 7.26 7.26 7.18 7.07 7.07 7.05 7.05 7.04 7.03 7.03 6.78 6.75
N
Ph
CN
3q
300 MHz, CDCl3
0.96 1.03 0.94 8.06 1.02 1.03 1.00

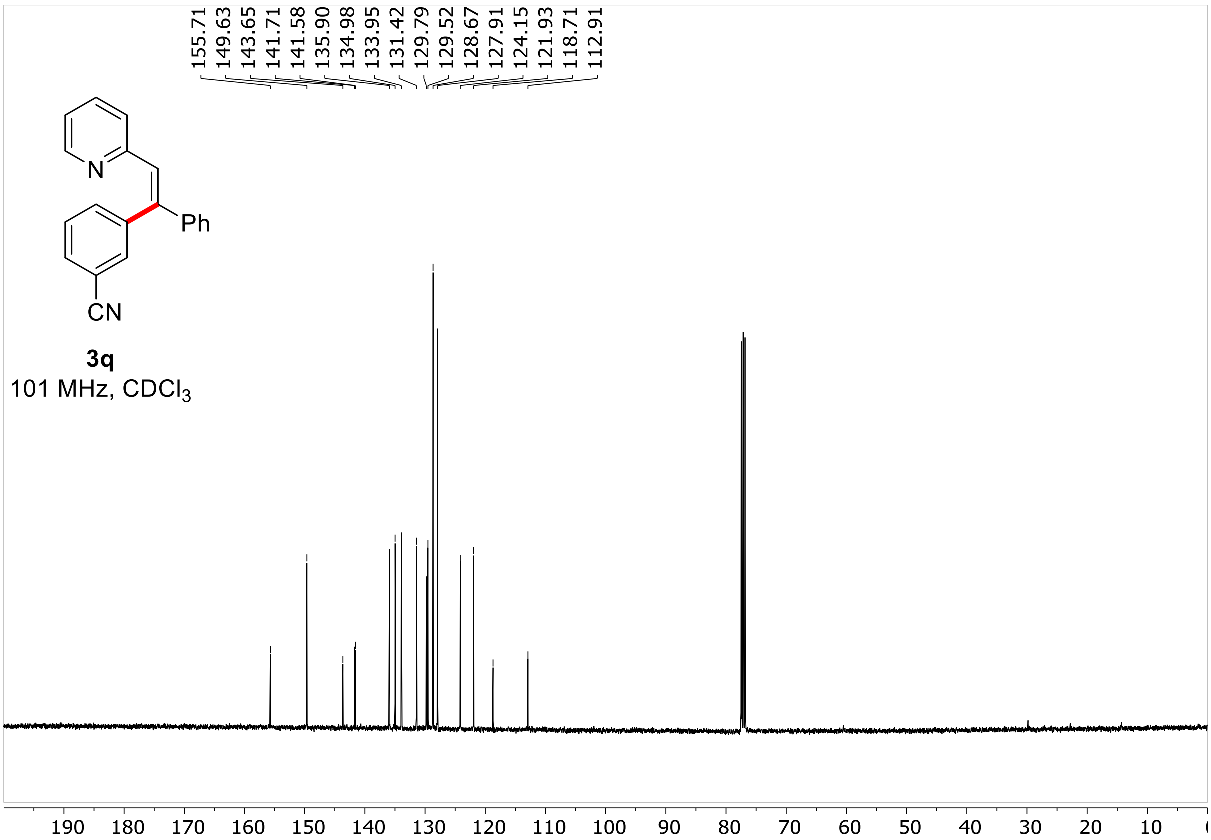
155.71 149.63 143.65 141.71 141.58 135.90 134.98 133.95 131.42 129.79 129.52 128.67 127.91 124.15 121.93 118.71 112.91
N
Ph
CN
3q
101 MHz, CDCl3

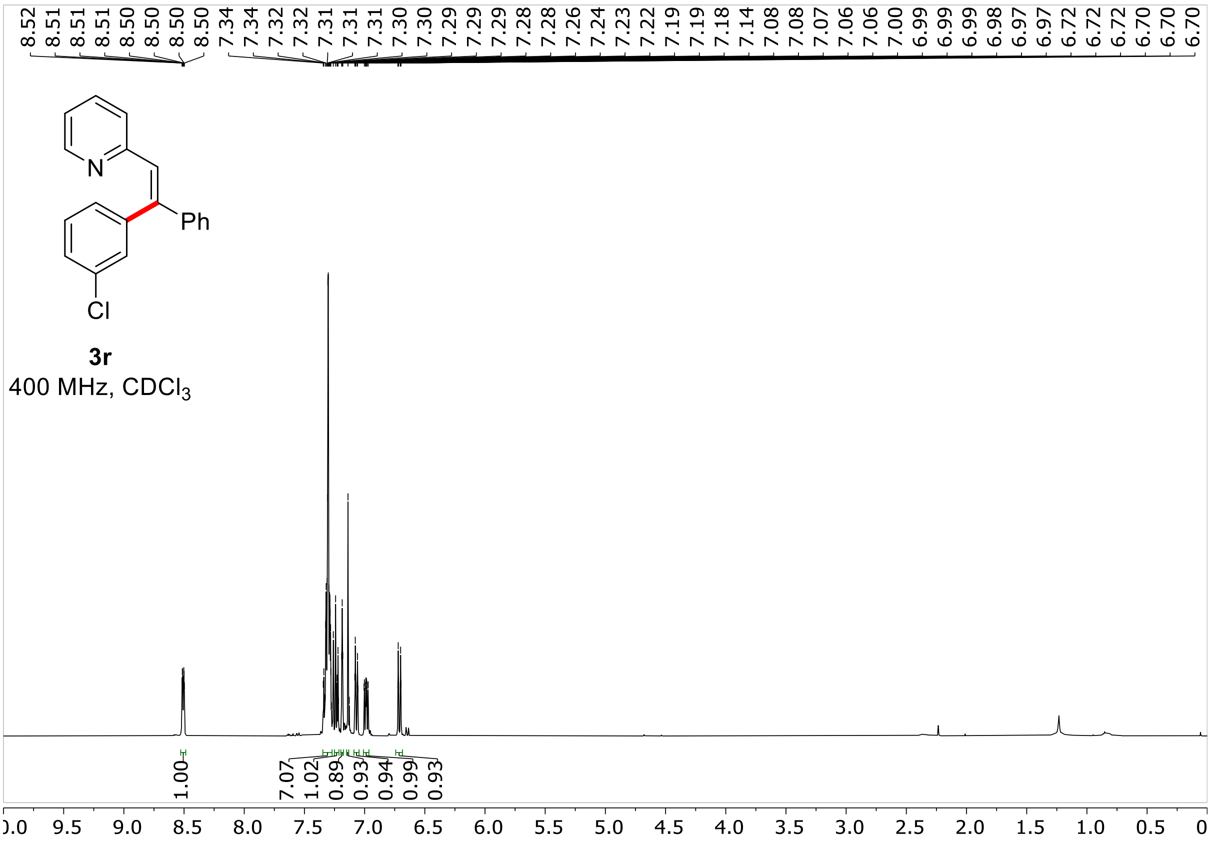
3r
400 MHz, CDCl3

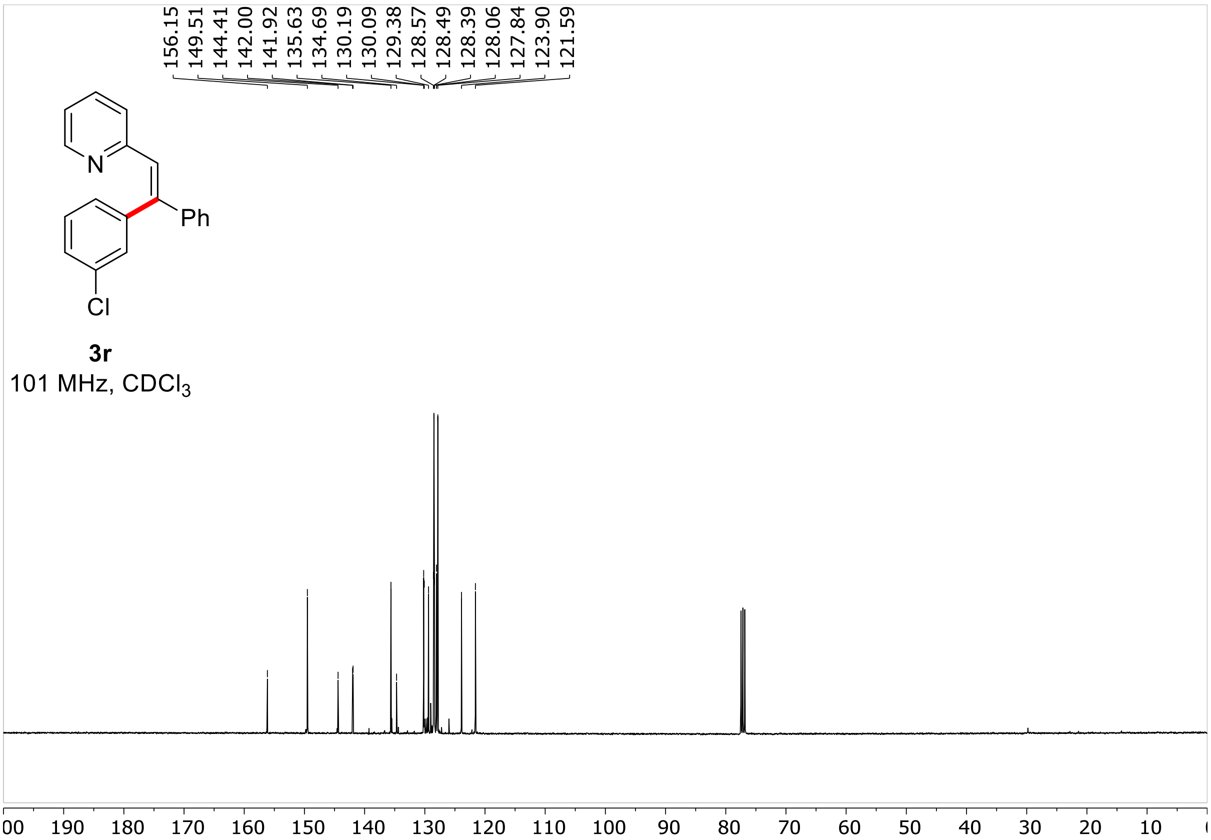
3r
101 MHz, CDCl3

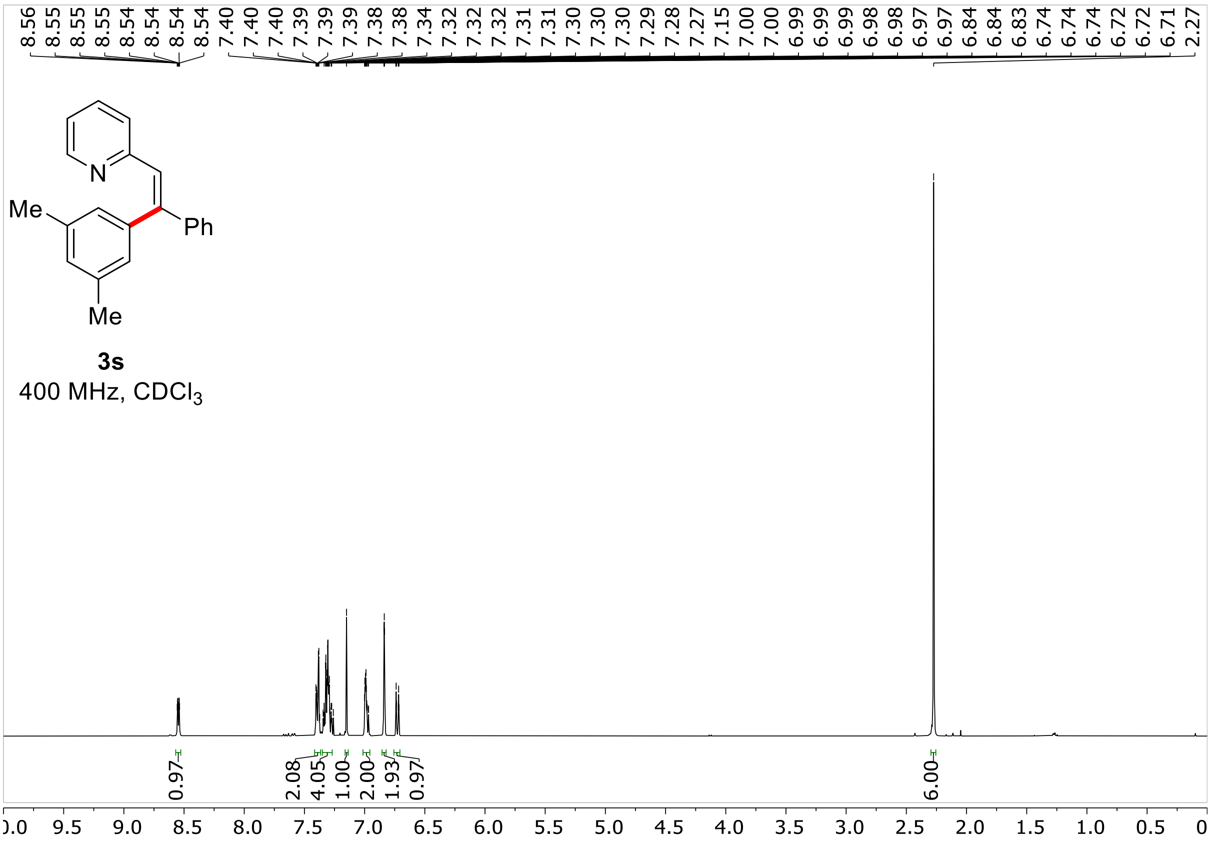
8.56
8.55
8.55
8.55
8.54
8.54
8.54
8.54
7.40
7.40
7.39
7.39
7.39
7.38
7.38
7.34
7.32
7.32
7.32
7.31
7.31
7.30
7.30
7.30
7.29
7.28
7.27
7.15
7.00
7.00
6.99
6.99
6.99
6.98
6.98
6.97
6.97
6.84
6.84
6.83
6.74
6.74
6.74
6.72
6.72
6.71
2.27
Me
N
Ph
Me
3s
400 MHz, CDCl3
0.97
2.08
4.05
1.00
2.00
1.93
0.97
6.00

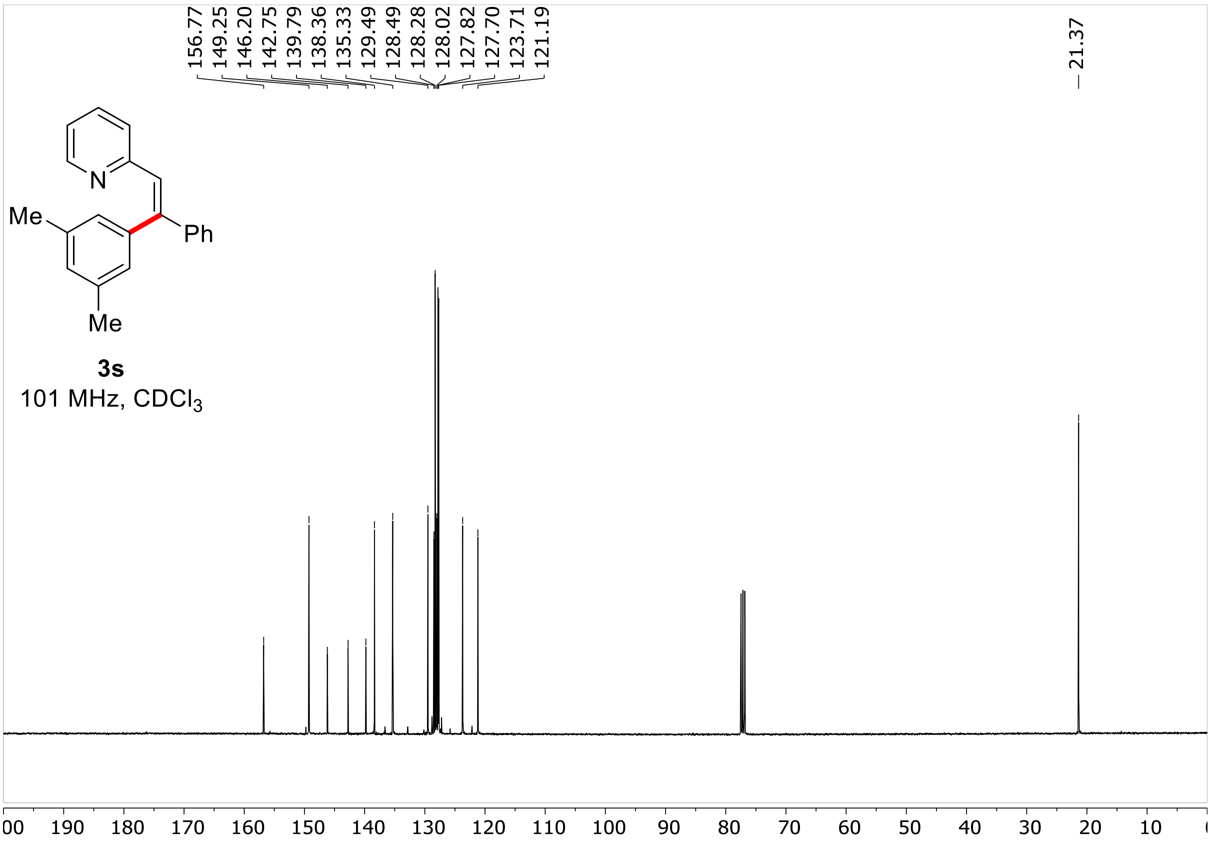
156.77
149.25
146.20
142.75
139.79
138.36
135.33
129.49
128.49
128.28
128.02
127.82
127.70
123.71
121.19
21.37
Me
N
Ph
Me
3s
101 MHz, CDCl3

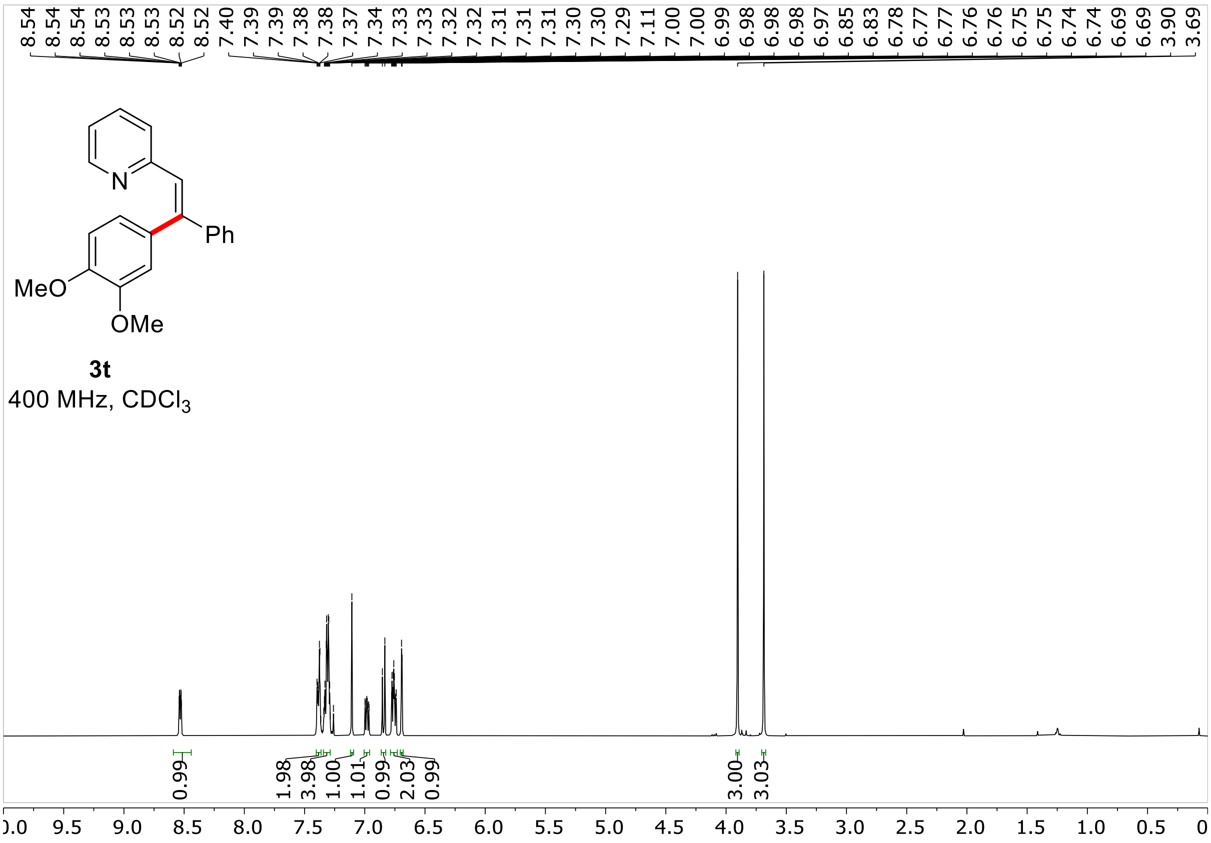

8.54
8.54
8.54
8.53
8.53
8.53
8.52
8.52
7.40
7.39
7.39
7.38
7.38
7.37
7.34
7.33
7.33
7.32
7.32
7.31
7.31
7.31
7.30
7.30
7.29
7.11
7.00
7.00
6.99
6.98
6.98
6.98
6.97
6.85
6.83
6.78
6.77
6.77
6.76
6.76
6.75
6.75
6.74
6.74
6.69
6.69
3.90
3.69
N
Ph
MeO
OMe
3t
400 MHz, CDCl3
0.99
1.98
3.98
1.00
1.01
0.99
2.03
0.99
3.00
3.03
0.0
9.5
9.0
8.5
8.0
7.5
7.0
6.5
6.0
5.5
5.0
4.5
4.0
3.5
3.0
2.5
2.0
1.5
1.0
0.5
0

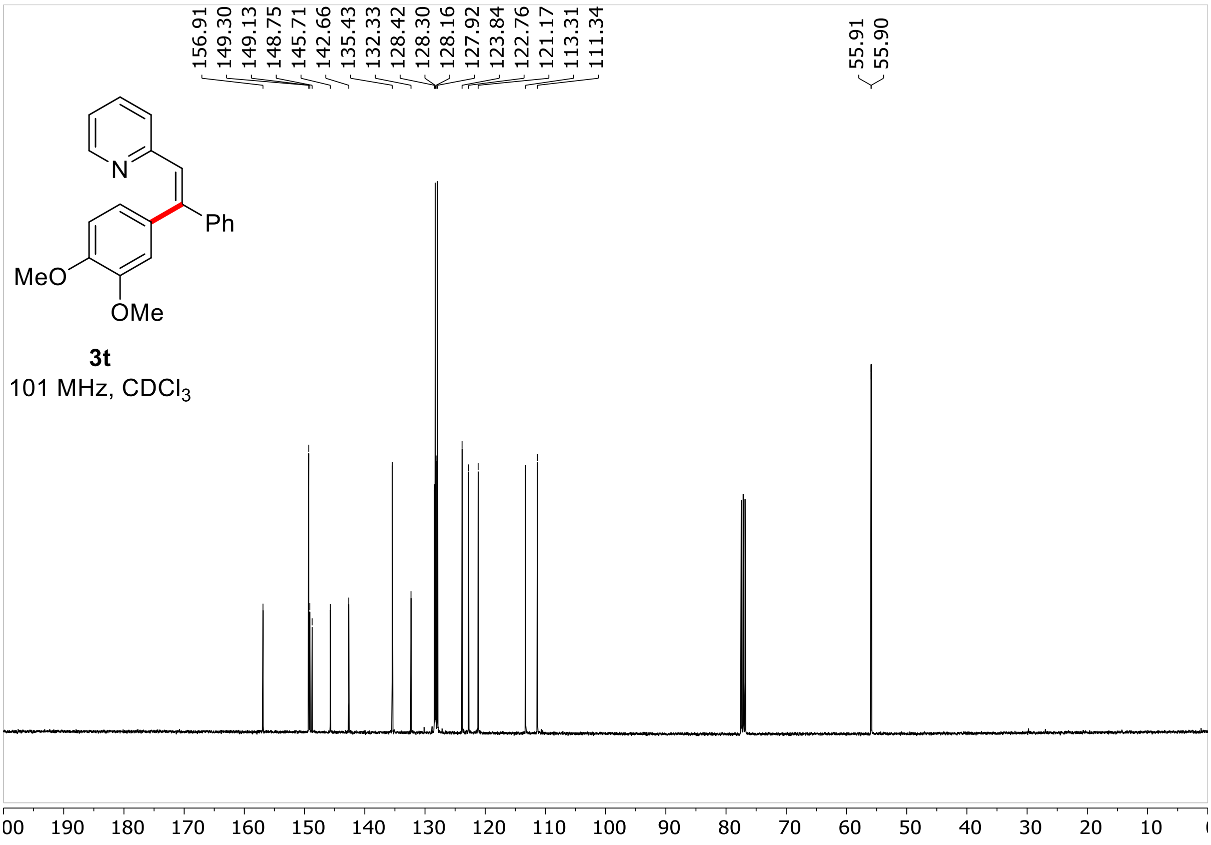

156.91
149.30
149.13
148.75
145.71
142.66
135.43
132.33
128.42
128.30
128.16
127.92
123.84
122.76
121.17
113.31
111.34
55.91
55.90
N
Ph
MeO
OMe
3t
101 MHz, CDCl3
00
190
180
170
160
150
140
130
120
110
100
90
80
70
60
50
40
30
20
10

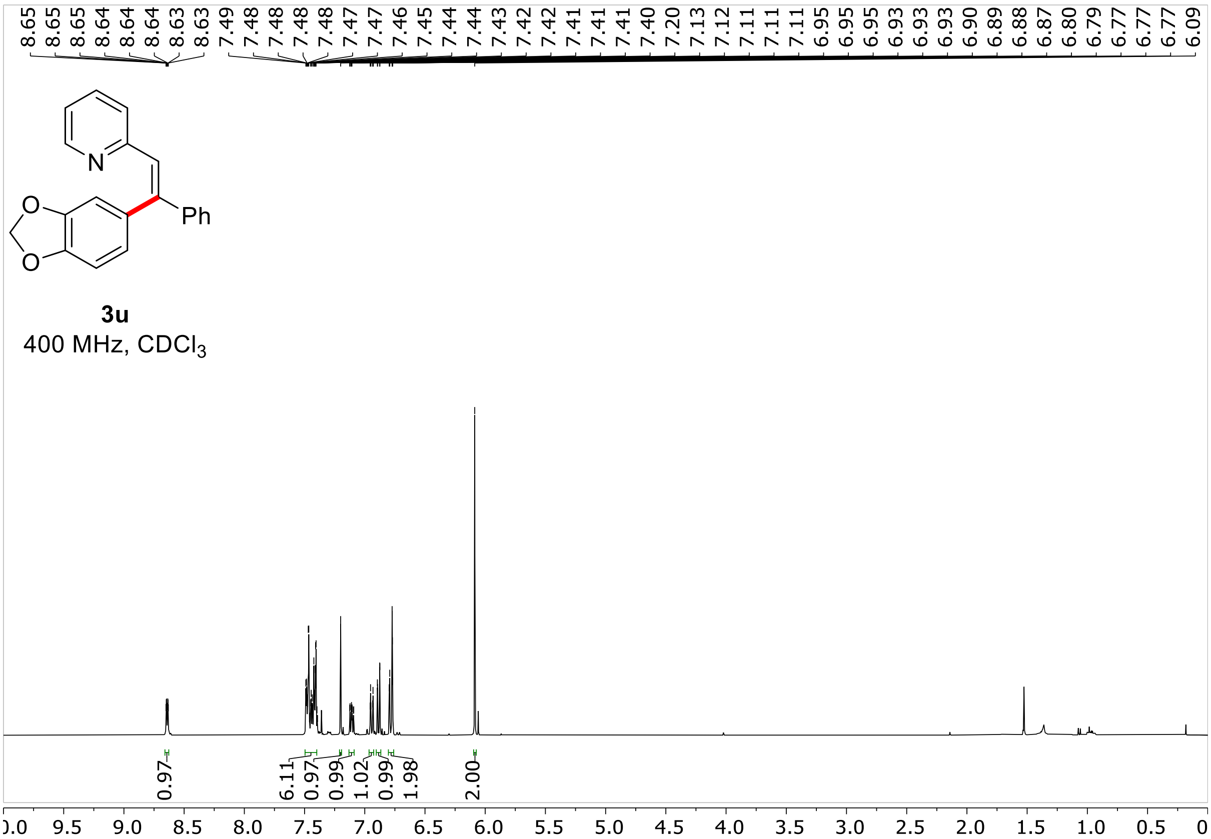
8.65 8.65 8.65 8.64 8.64 8.64 8.63 8.63 7.49 7.48 7.48 7.48 7.47 7.47 7.46 7.45 7.44 7.44 7.43 7.42 7.42 7.41 7.41 7.41 7.40 7.20 7.13 7.12 7.11 7.11 7.11 6.95 6.95 6.95 6.93 6.93 6.93 6.90 6.89 6.88 6.87 6.80 6.79 6.77 6.77 6.77 6.09
N
O
O
Ph
3u
400 MHz, CDCl3
0.97 6.11 0.97 0.99 1.02 0.99 1.98 2.00
0.0 9.5 9.0 8.5 8.0 7.5 7.0 6.5 6.0 5.5 5.0 4.5 4.0 3.5 3.0 2.5 2.0 1.5 1.0 0.5 0

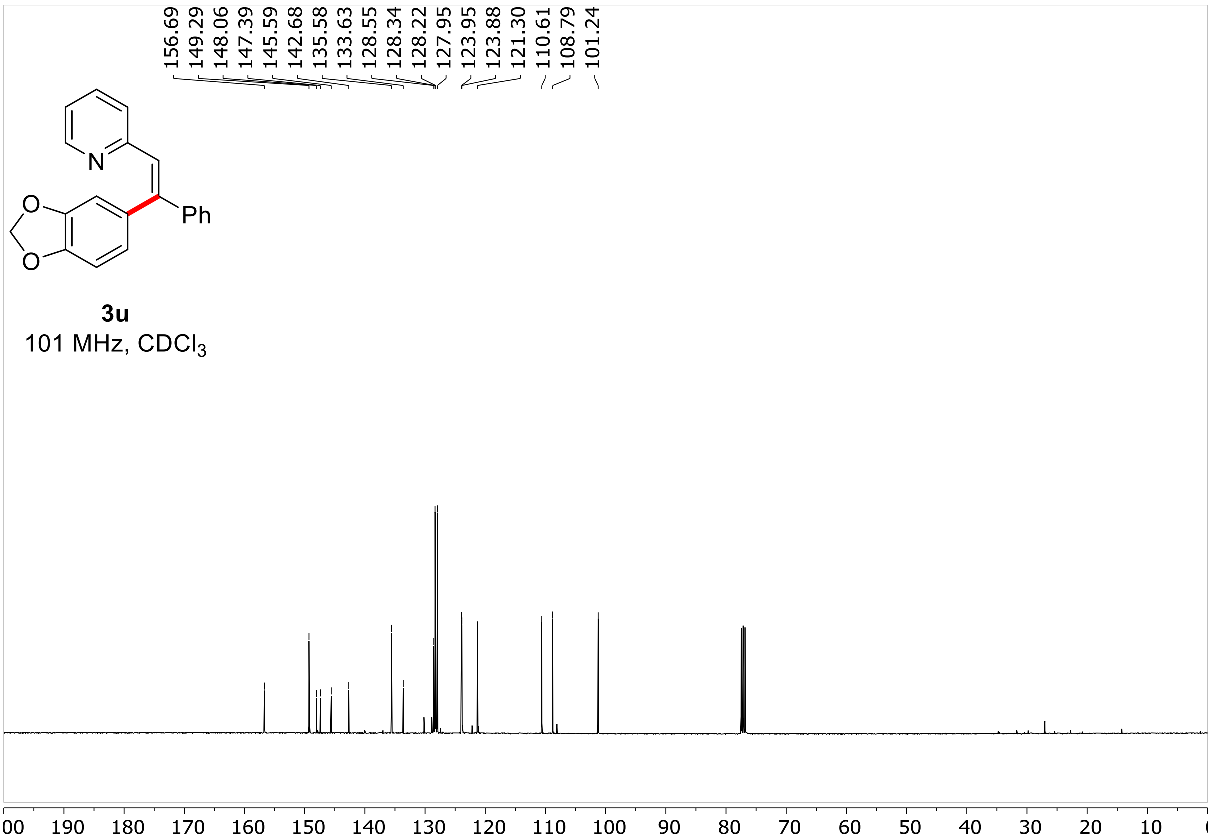
156.69 149.29 148.06 147.39 145.59 142.68 135.58 133.63 128.55 128.34 128.22 127.95 123.95 123.88 121.30 110.61 108.79 101.24
N
O
O
Ph
3u
101 MHz, CDCl3
00 190 180 170 160 150 140 130 120 110 100 90 80 70 60 50 40 30 20 10

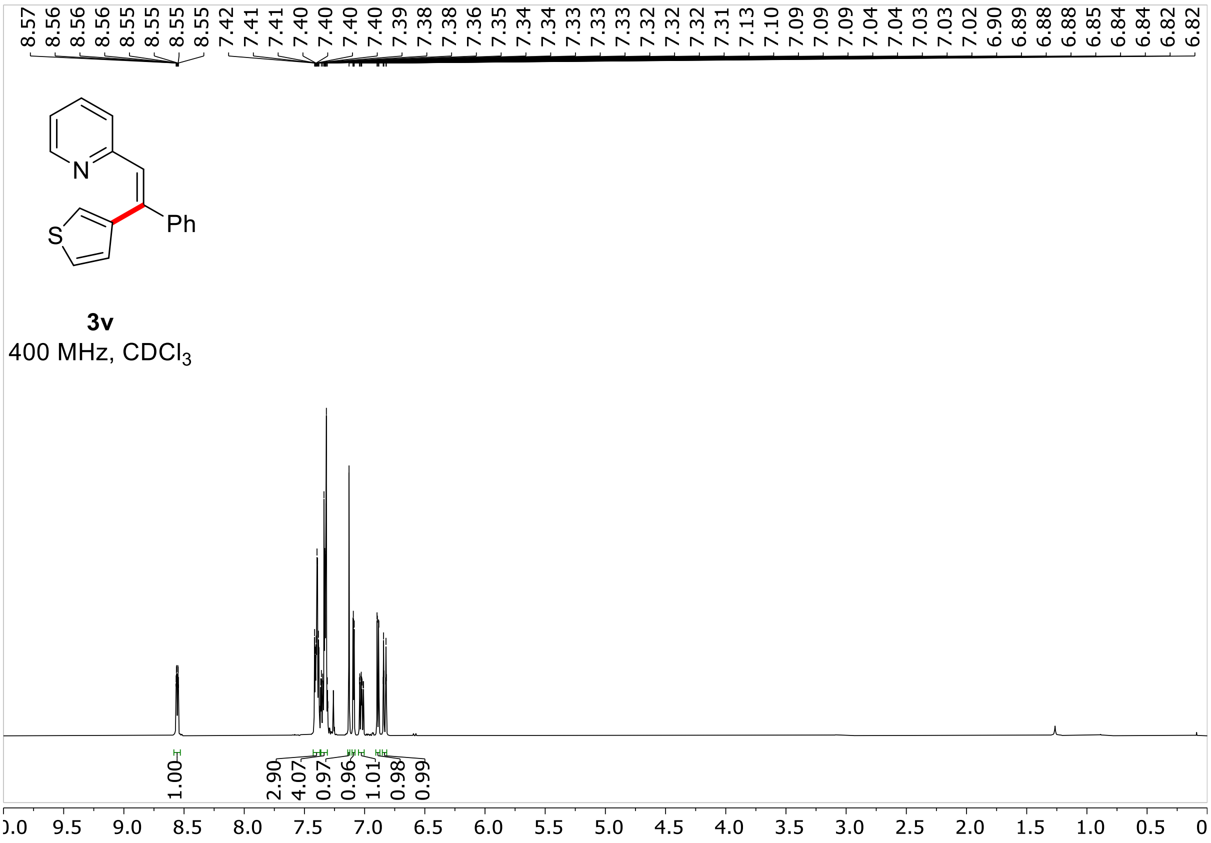
8.57 8.56 8.56 8.56 8.55 8.55 8.55 8.55 7.42 7.41 7.41 7.40 7.40 7.40 7.40 7.39 7.38 7.38 7.36 7.35 7.34 7.34 7.33 7.33 7.33 7.32 7.32 7.32 7.31 7.13 7.10 7.09 7.09 7.04 7.04 7.03 7.03 7.02 6.90 6.89 6.88 6.85 6.84 6.84 6.82 6.82
N
S
Ph
3v
400 MHz, CDCl3
1.00
2.90
4.07
0.97
0.96
1.01
0.98
0.99

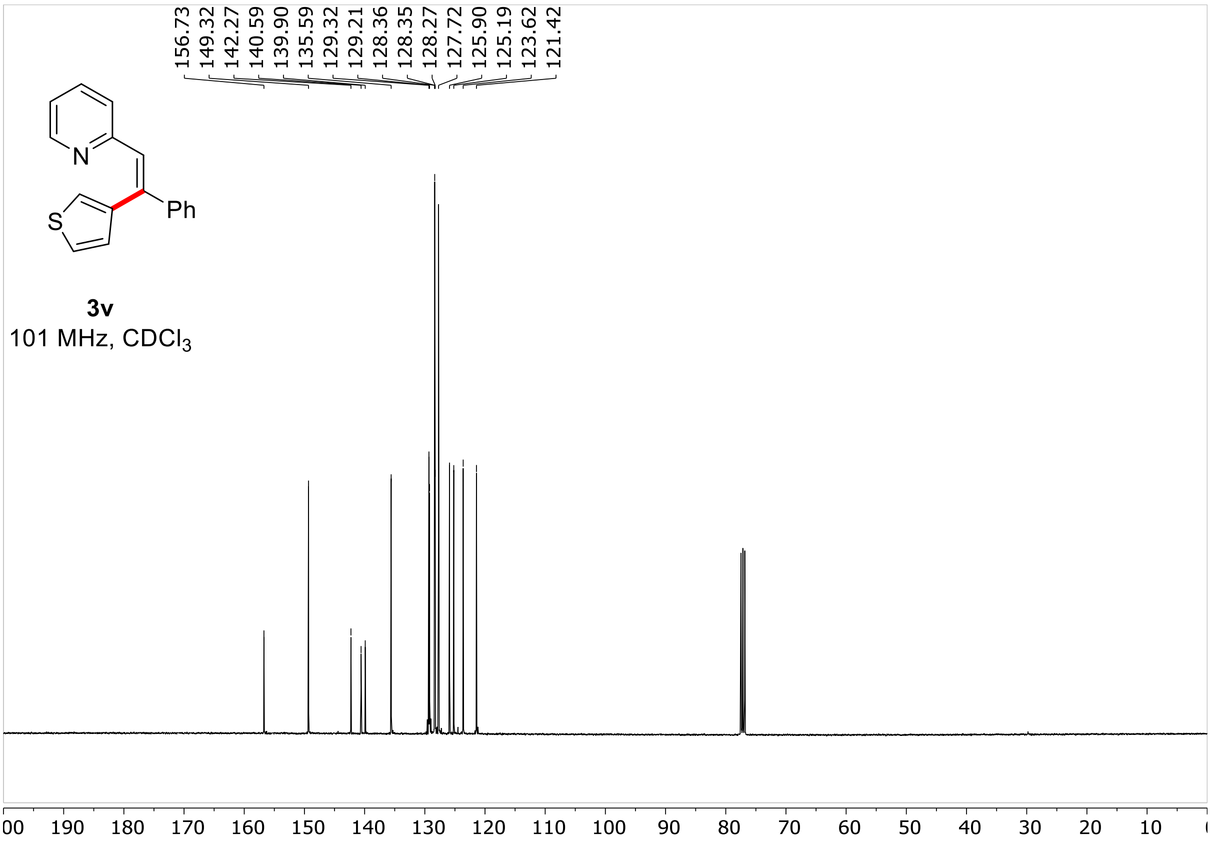
156.73 149.32 142.27 140.59 139.90 135.59 129.32 129.21 128.36 128.35 128.27 127.72 125.90 125.19 123.62 121.42
N
S
Ph
3v
101 MHz, CDCl3

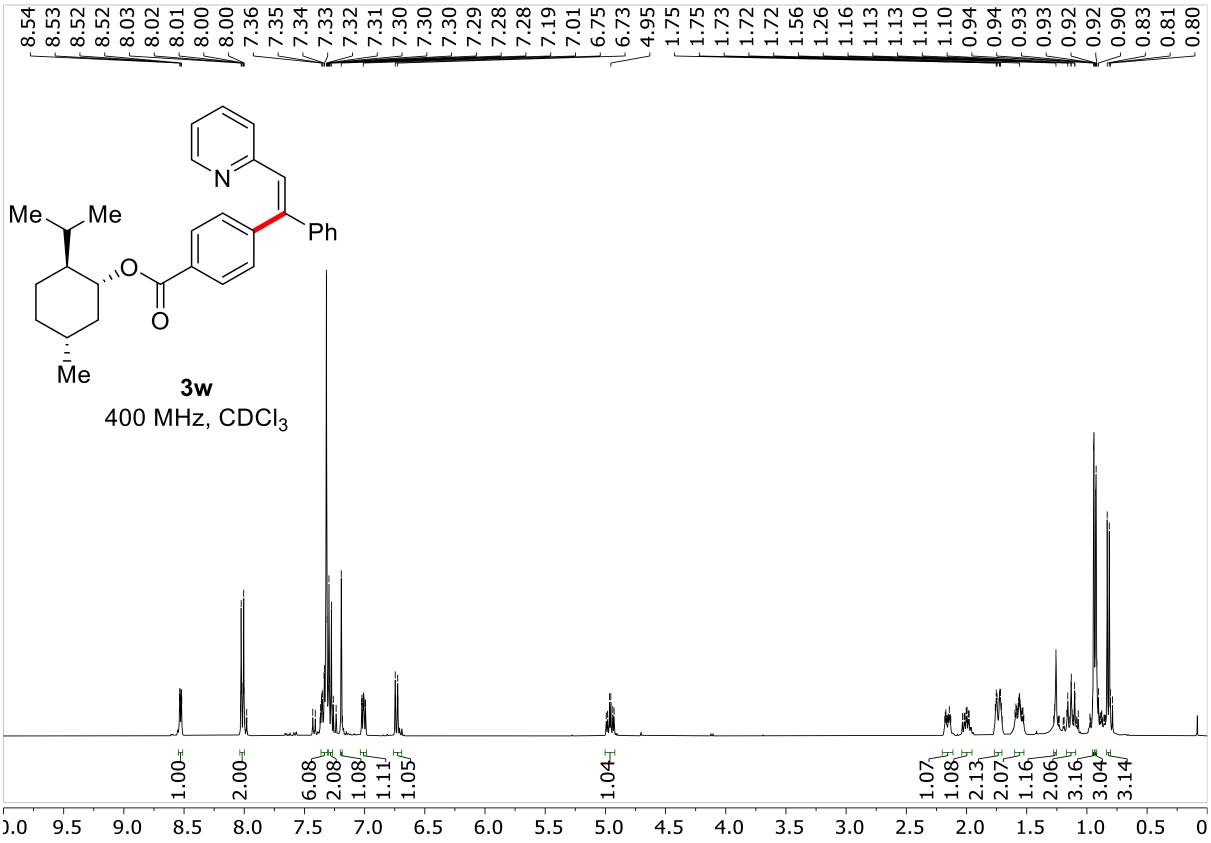
8.54 8.53 8.52 8.52 8.03 8.02 8.01 8.00 8.00 7.36 7.35 7.34 7.33 7.32 7.31 7.30 7.30 7.30 7.29 7.28 7.28 7.19 7.01 6.75 6.73 4.95 1.75 1.75 1.73 1.72 1.72 1.56 1.26 1.16 1.13 1.13 1.10 1.10 0.94 0.94 0.93 0.93 0.92 0.92 0.90 0.83 0.81 0.80
Me
Me
N
Ph
O
O
Me
3w
400 MHz, CDCl3
1.00 2.00 6.08 2.08 1.08 1.11 1.05 1.04 1.07 1.08 2.13 2.07 1.16 2.06 3.16 3.04 3.14
9.5 9.0 8.5 8.0 7.5 7.0 6.5 6.0 5.5 5.0 4.5 4.0 3.5 3.0 2.5 2.0 1.5 1.0 0.5 0

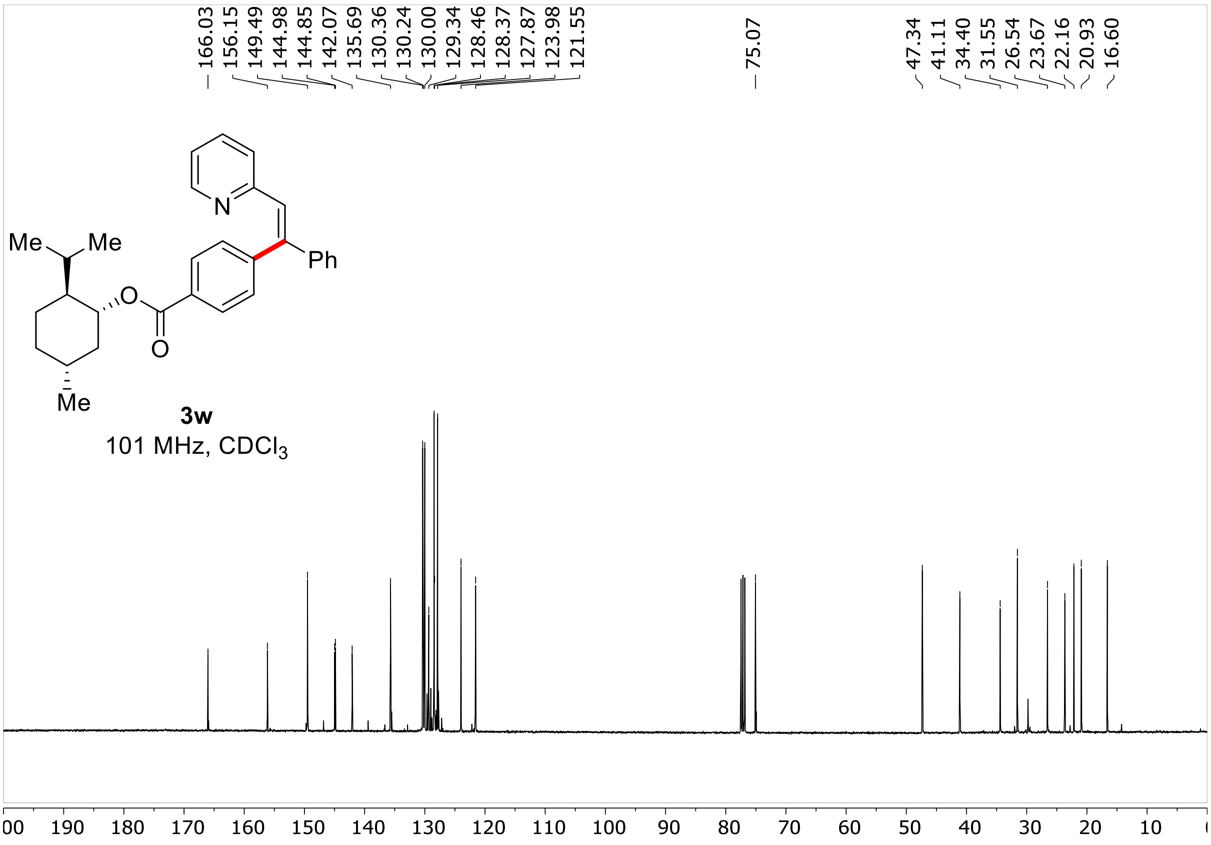
166.03 156.15 149.49 144.98 144.85 142.07 135.69 130.36 130.24 130.00 129.34 128.46 128.37 127.87 123.98 121.55 75.07 47.34 41.11 34.40 31.55 26.54 23.67 22.16 20.93 16.60
Me
Me
N
Ph
O
O
Me
3w
101 MHz, CDCl3
190 180 170 160 150 140 130 120 110 100 90 80 70 60 50 40 30 20 10

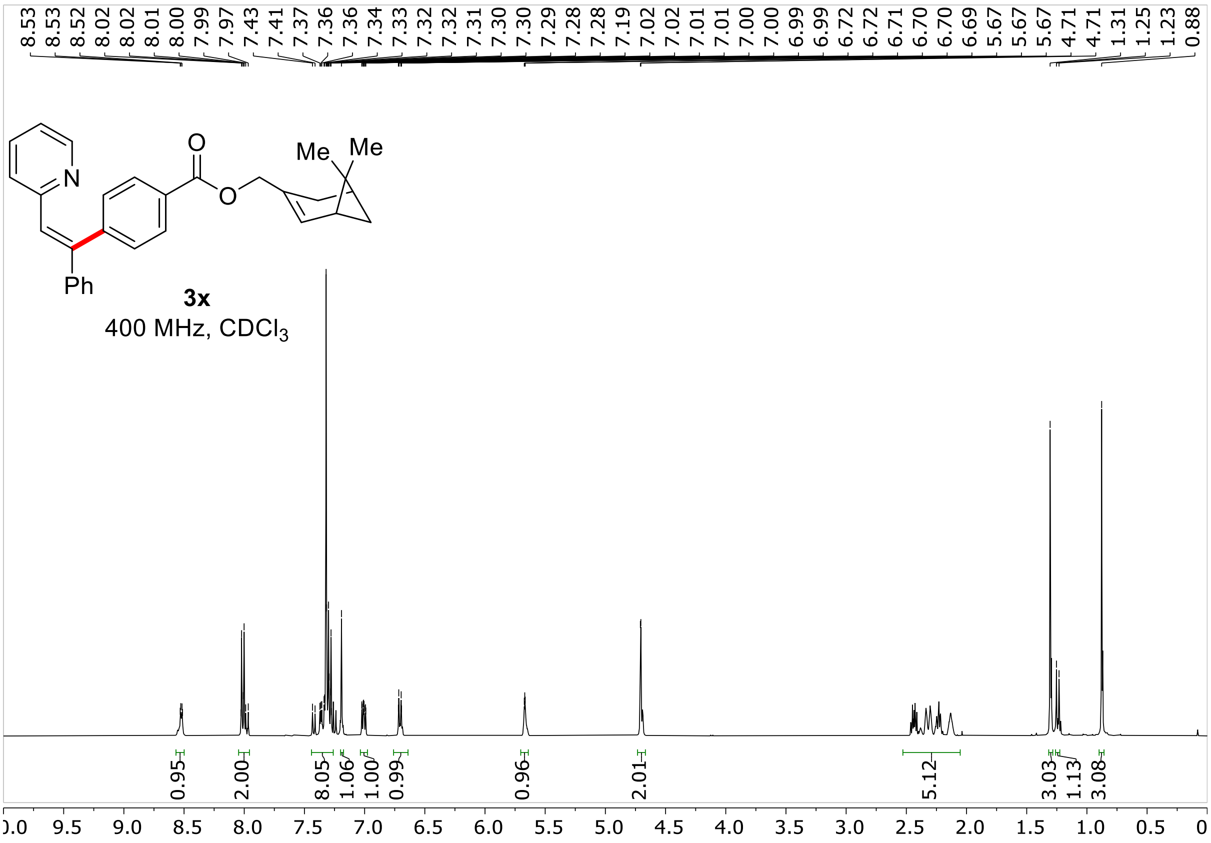
8.53
8.53
8.52
8.02
8.02
8.01
8.00
7.99
7.97
7.43
7.41
7.37
7.36
7.36
7.34
7.33
7.32
7.32
7.31
7.30
7.30
7.29
7.28
7.28
7.19
7.02
7.02
7.01
7.01
7.00
7.00
6.99
6.99
6.72
6.72
6.71
6.70
6.70
6.69
5.67
5.67
5.67
4.71
4.71
4.71
1.31
1.25
1.23
0.88
Me
Me
N
O
O
Ph
3x
400 MHz, CDCl3
0.95
2.00
8.05
1.06
1.00
0.99
0.96
2.01
5.12
3.03
1.13
3.08

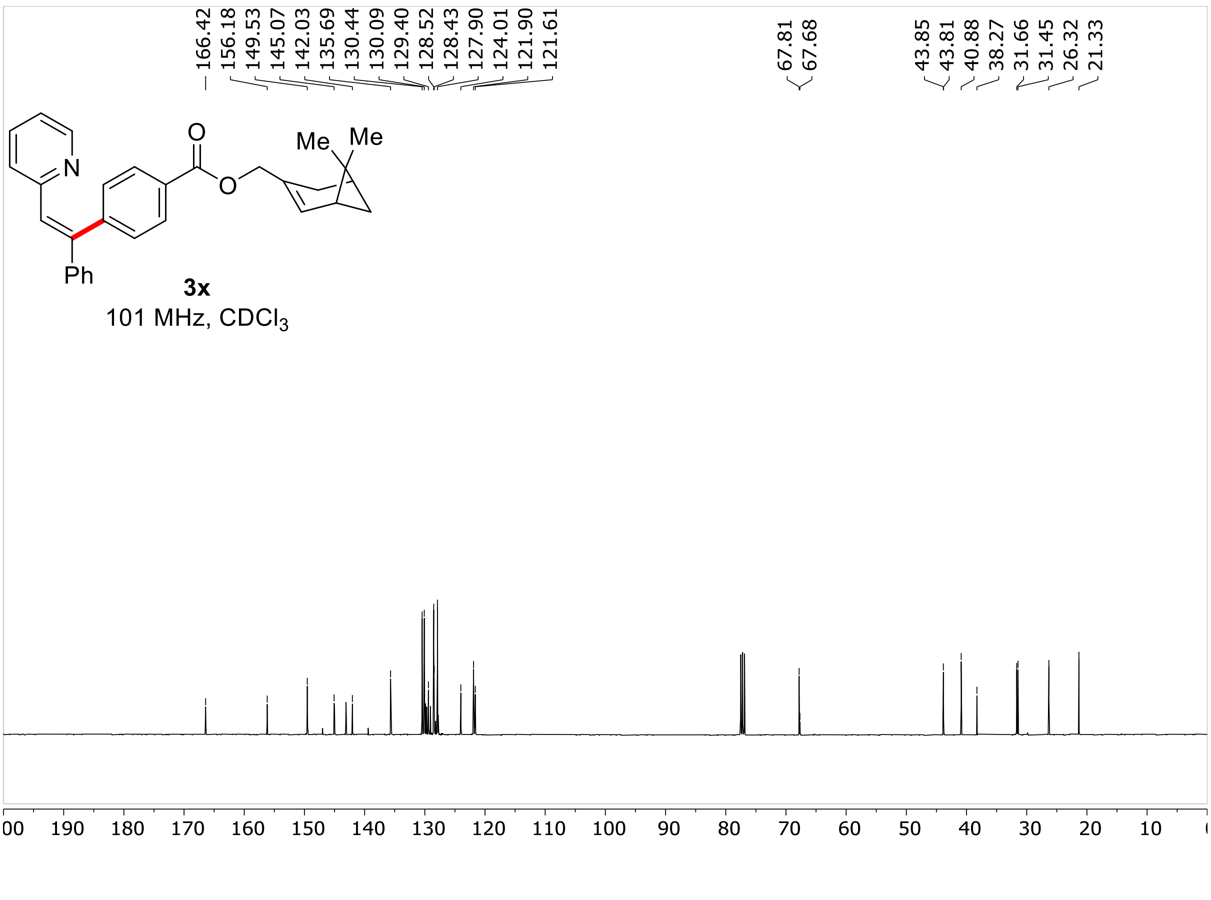
166.42
156.18
149.53
145.07
142.03
135.69
130.44
130.09
129.40
128.52
128.43
127.90
124.01
121.90
121.61
67.81
67.68
43.85
43.81
40.88
38.27
31.66
31.45
26.32
21.33
Me
Me
N
O
O
Ph
3x
101 MHz, CDCl3

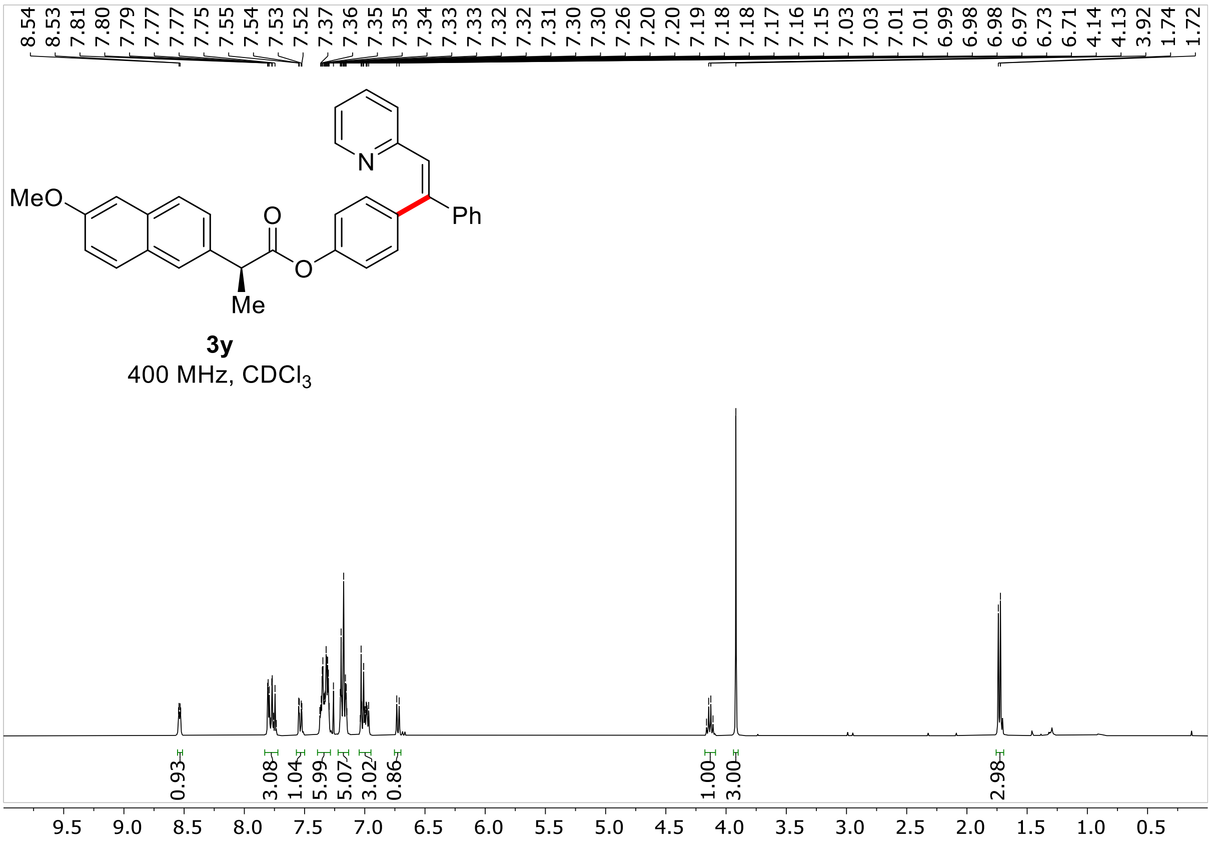

8.54 8.53 7.81 7.80 7.79 7.77 7.77 7.75 7.55 7.54 7.53 7.52 7.37 7.36 7.35 7.35 7.34 7.33 7.33 7.32 7.32 7.31 7.30 7.30 7.26 7.20 7.20 7.19 7.18 7.18 7.17 7.16 7.15 7.03 7.03 7.01 7.01 6.99 6.98 6.98 6.97 6.73 6.71 4.14 4.13 3.92 1.74 1.72
N
MeO
O
O
Ph
Me
3y
400 MHz, $CDCl_3$
0.93 3.08 1.04 5.99 5.07 3.02 0.86 1.00 3.00 2.98
9.5 9.0 8.5 8.0 7.5 7.0 6.5 6.0 5.5 5.0 4.5 4.0 3.5 3.0 2.5 2.0 1.5 1.0 0.5

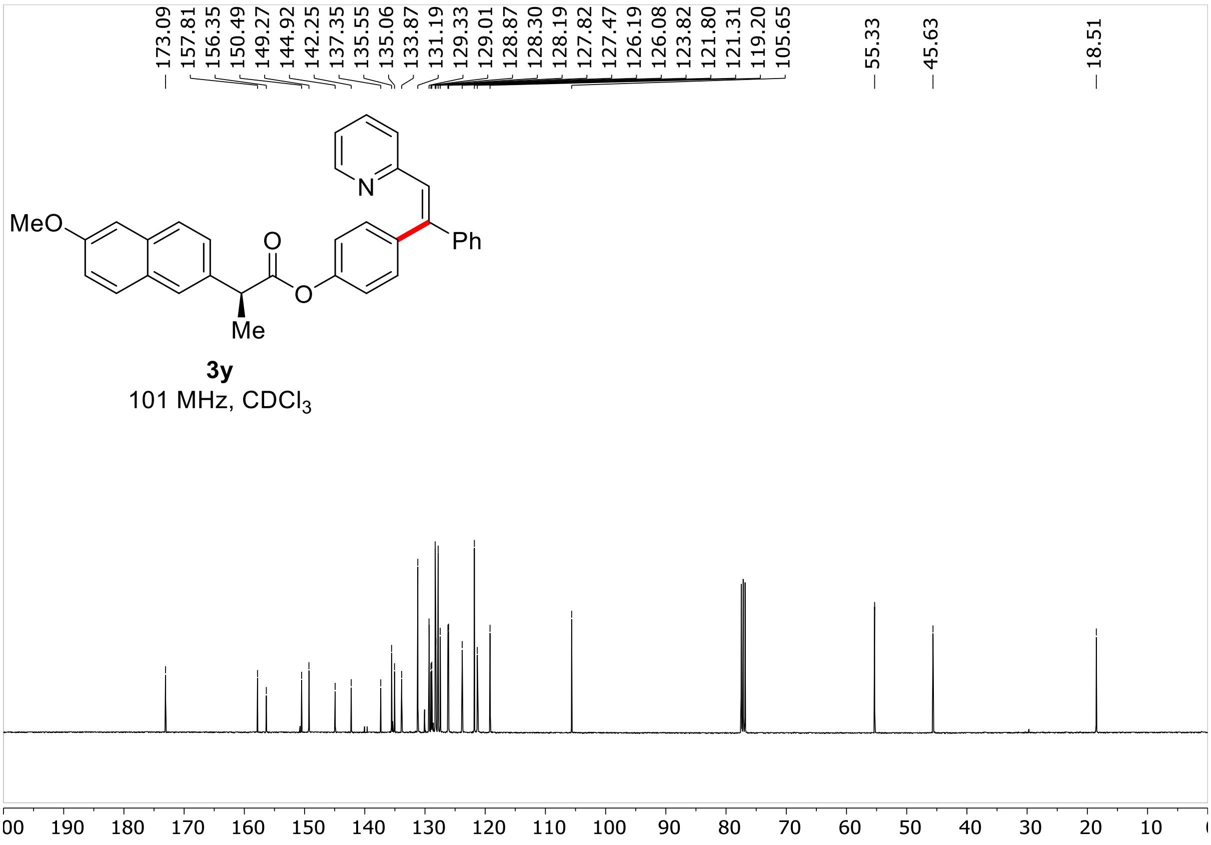

173.09 157.81 156.35 150.49 149.27 144.92 142.25 137.35 135.55 135.06 133.87 131.19 129.33 129.01 128.87 128.30 128.19 127.82 127.47 126.19 126.08 123.82 121.80 121.31 119.20 105.65 55.33 45.63 18.51
N
MeO
O
O
Ph
Me
3y
101 MHz, $CDCl_3$
00 190 180 170 160 150 140 130 120 110 100 90 80 70 60 50 40 30 20 10

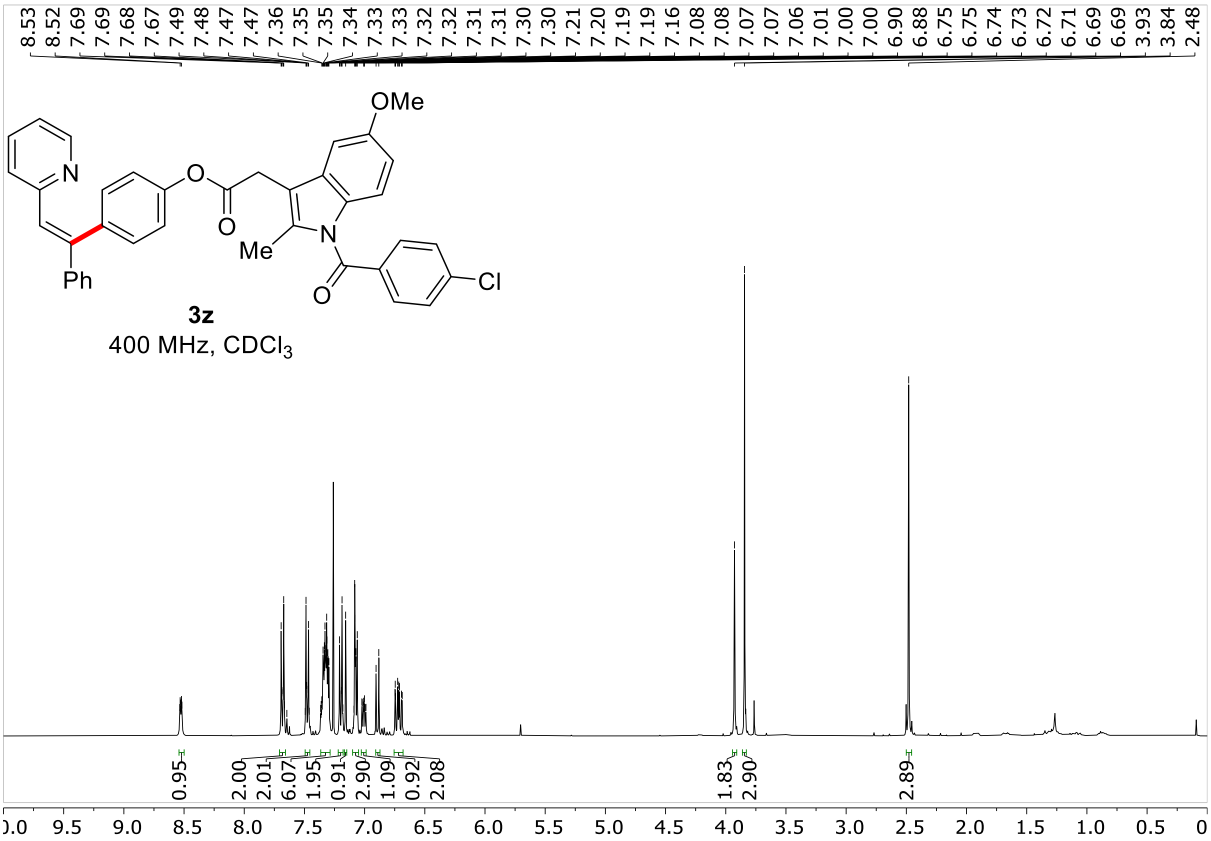
8.53
8.52
7.69
7.69
7.68
7.67
7.49
7.48
7.47
7.47
7.36
7.35
7.35
7.34
7.33
7.33
7.32
7.32
7.31
7.31
7.30
7.30
7.21
7.20
7.19
7.19
7.16
7.08
7.08
7.07
7.07
7.06
7.01
7.00
7.00
6.90
6.88
6.75
6.75
6.74
6.73
6.72
6.71
6.69
6.69
3.93
3.84
2.48
OMe
N
O
O
Me
N
Ph
O
Cl
3z
400 MHz, CDCl3
0.95
2.00
2.01
6.07
1.95
0.91
2.90
1.09
0.92
2.08
1.83
2.90
2.89

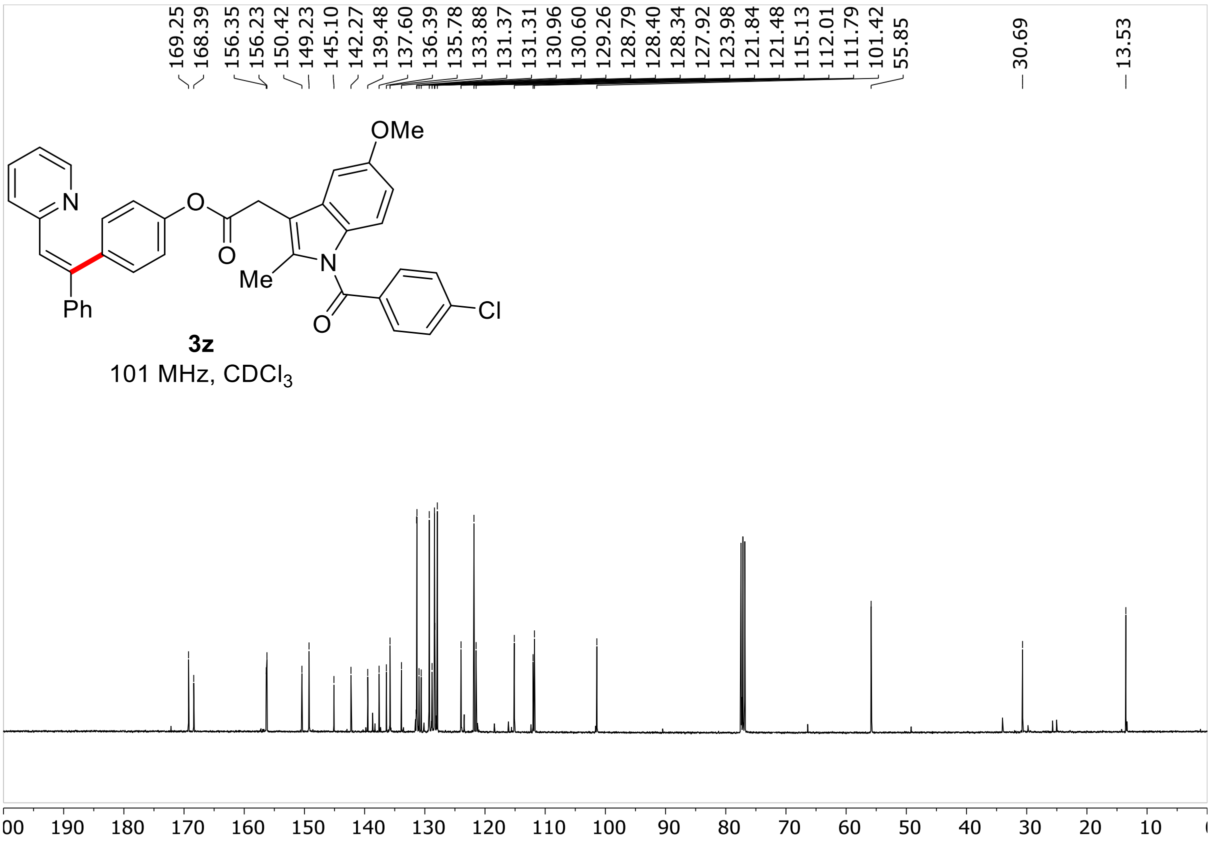
169.25
168.39
156.35
156.23
150.42
149.23
145.10
142.27
139.48
137.60
136.39
135.78
133.88
131.37
131.31
130.96
130.60
129.26
128.79
128.40
128.34
127.92
123.98
121.84
121.48
115.13
112.01
111.79
101.42
55.85
30.69
13.53
OMe
N
O
O
Me
N
Ph
O
Cl
3z
101 MHz, CDCl3

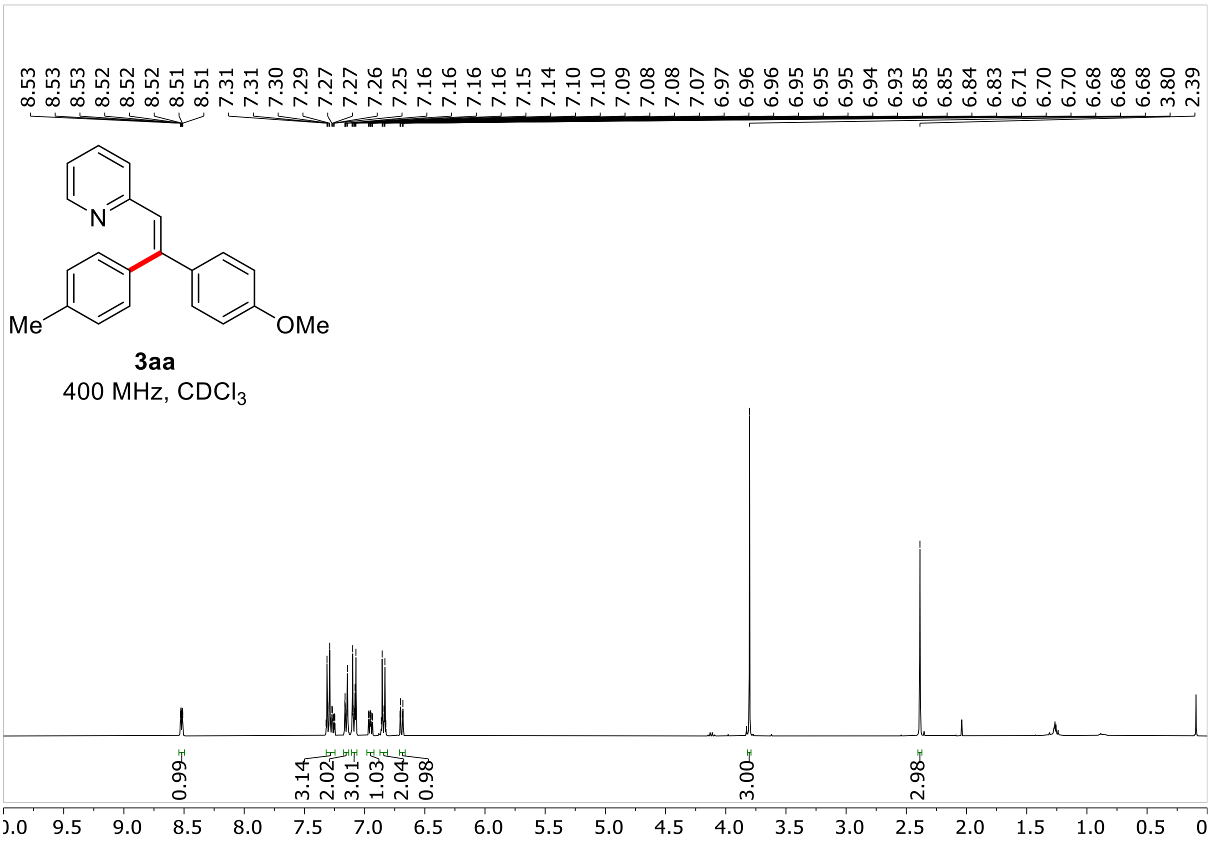
8.53 8.53 8.53 8.52 8.52 8.52 8.51 8.51 7.31 7.31 7.30 7.29 7.27 7.27 7.26 7.25 7.16 7.16 7.16 7.16 7.15 7.14 7.10 7.10 7.09 7.08 7.08 7.07 6.97 6.96 6.96 6.95 6.95 6.94 6.93 6.85 6.85 6.84 6.83 6.71 6.70 6.68 6.68 6.68 3.80 2.39
N
Me
OMe
3aa
400 MHz, CDCl3
0.99
3.14
2.02
3.01
1.03
2.04
0.98
3.00
2.98
0.0 9.5 9.0 8.5 8.0 7.5 7.0 6.5 6.0 5.5 5.0 4.5 4.0 3.5 3.0 2.5 2.0 1.5 1.0 0.5 0

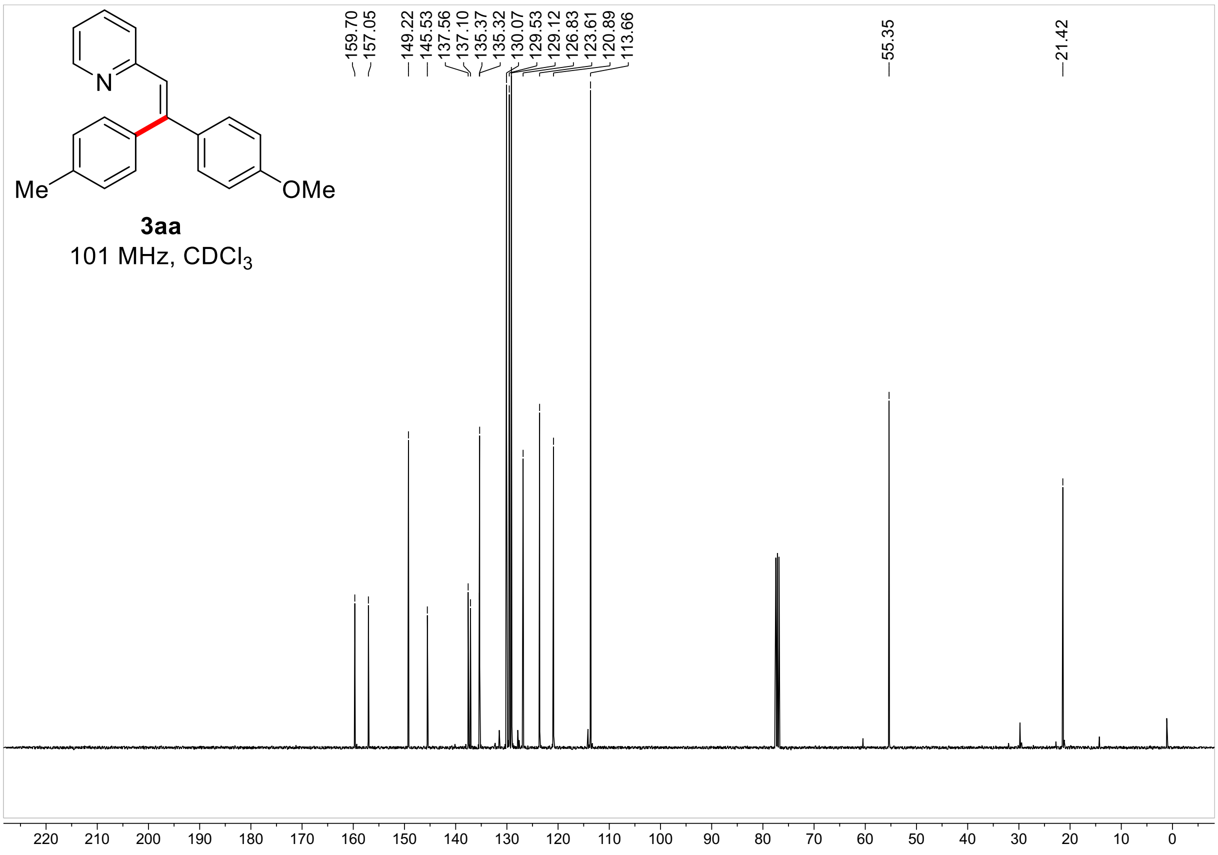
159.70 157.05 149.22 145.53 137.56 137.10 135.37 135.32 130.07 129.53 129.12 126.83 123.61 120.89 113.66
55.35
21.42
N
Me
OMe
3aa
101 MHz, CDCl3
220 210 200 190 180 170 160 150 140 130 120 110 100 90 80 70 60 50 40 30 20 10 0

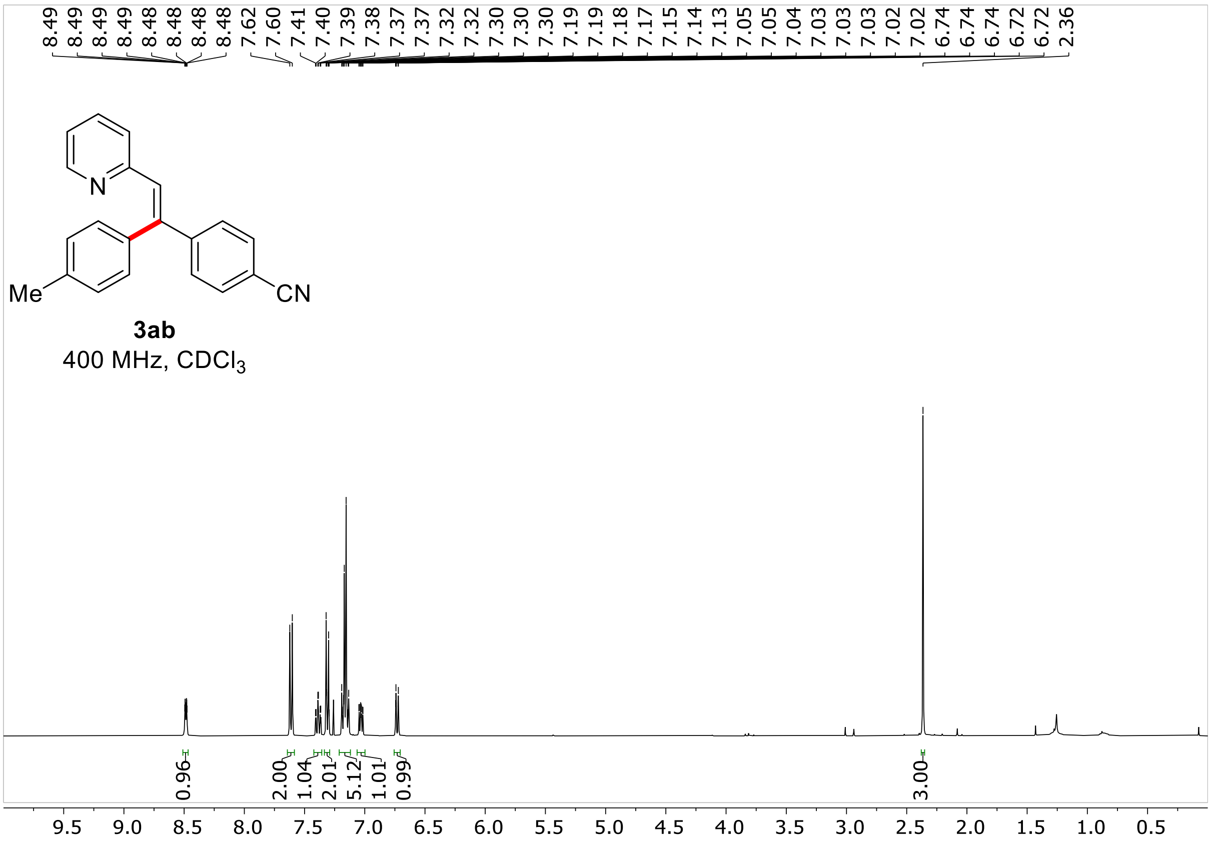
8.49 8.49 8.49 8.49 8.48 8.48 8.48 8.48 7.62 7.60 7.41 7.40 7.39 7.38 7.37 7.37 7.32 7.32 7.30 7.30 7.30 7.19 7.19 7.18 7.17 7.15 7.14 7.13 7.05 7.05 7.04 7.03 7.03 7.03 7.02 7.02 6.74 6.74 6.74 6.72 6.72 2.36
N
Me
CN
3ab
400 MHz, CDCl3
0.96
2.00
1.04
2.01
5.12
1.01
0.99
3.00
9.5 9.0 8.5 8.0 7.5 7.0 6.5 6.0 5.5 5.0 4.5 4.0 3.5 3.0 2.5 2.0 1.5 1.0 0.5

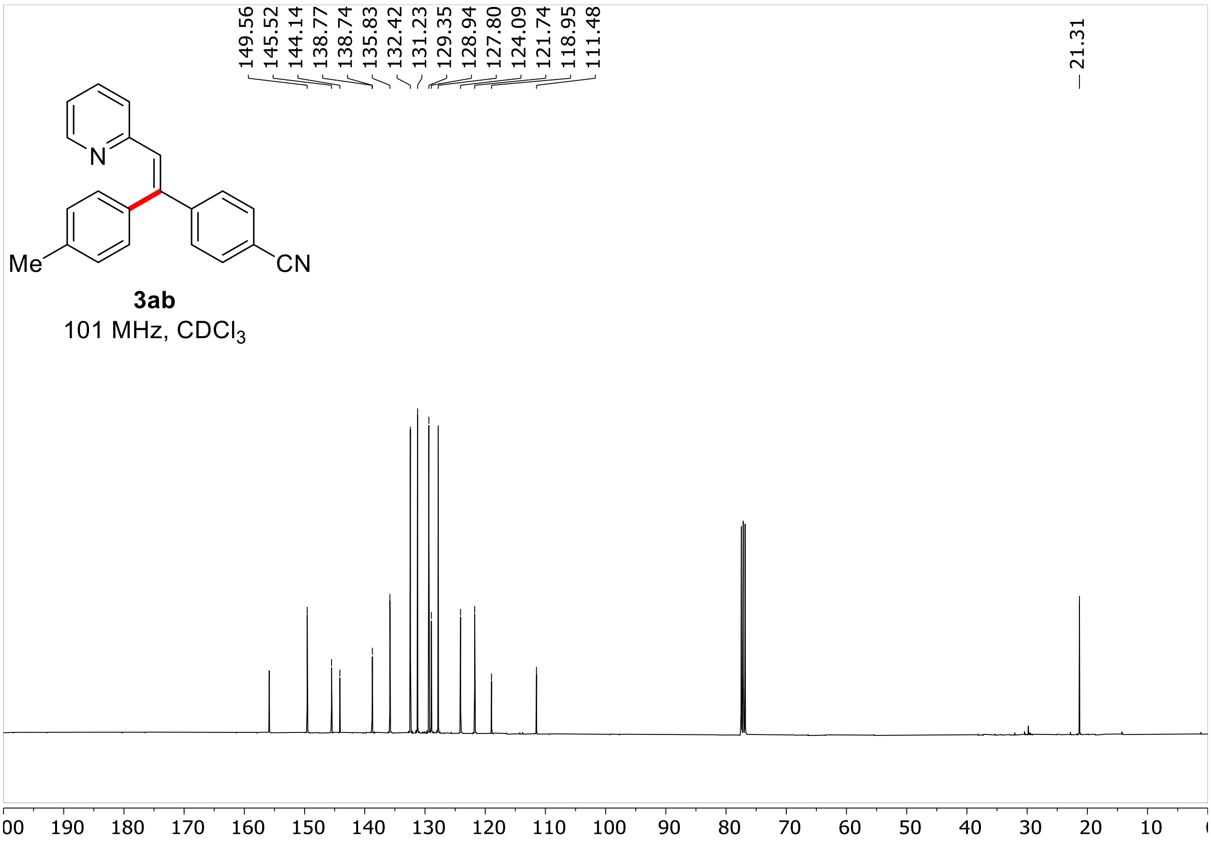
149.56 145.52 144.14 138.77 138.74 135.83 132.42 131.23 129.35 128.94 127.80 124.09 121.74 118.95 111.48
21.31
N
Me
CN
3ab
101 MHz, CDCl3
190 180 170 160 150 140 130 120 110 100 90 80 70 60 50 40 30 20 10

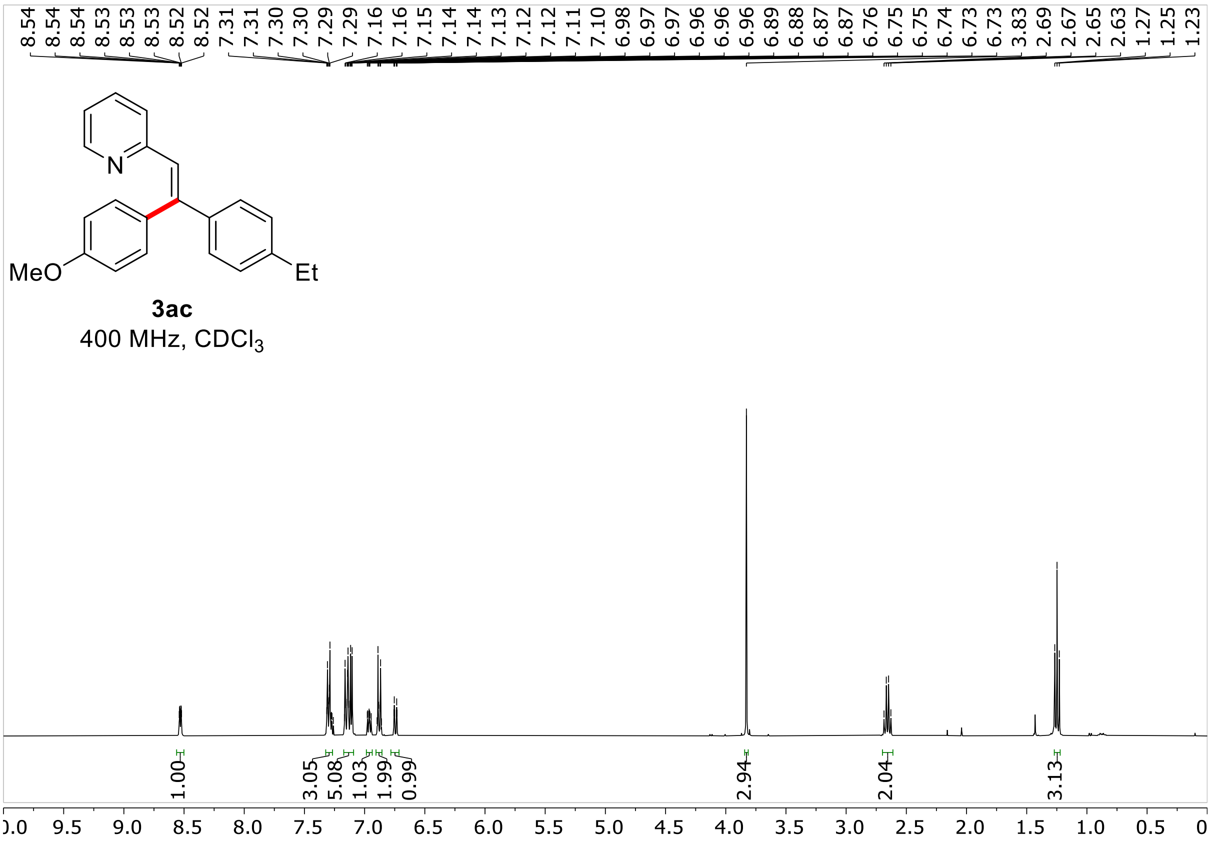
8.54 8.54 8.54 8.53 8.53 8.53 8.52 8.52 7.31 7.31 7.30 7.29 7.29 7.16 7.16 7.15 7.14 7.14 7.13 7.12 7.12 7.11 7.10 6.98 6.97 6.97 6.96 6.96 6.96 6.89 6.88 6.87 6.76 6.75 6.75 6.74 6.73 6.73 3.83 2.69 2.67 2.65 2.63 1.27 1.25 1.23
N
MeO
Et
3ac
400 MHz, CDCl3
1.00
3.05
5.08
1.03
1.99
0.99
2.94
2.04
3.13

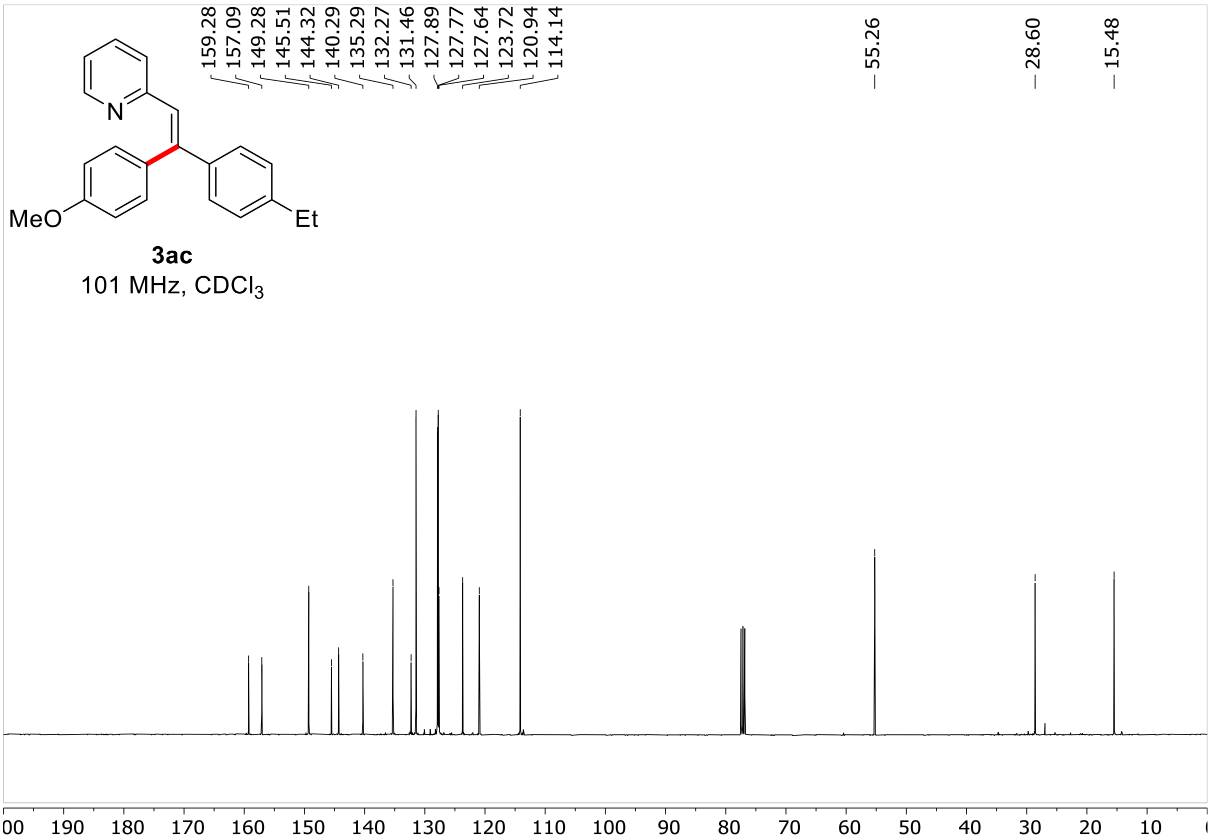
159.28 157.09 149.28 145.51 144.32 140.29 135.29 132.27 131.46 127.89 127.77 127.64 123.72 120.94 114.14
55.26
28.60
15.48
N
MeO
Et
3ac
101 MHz, CDCl3

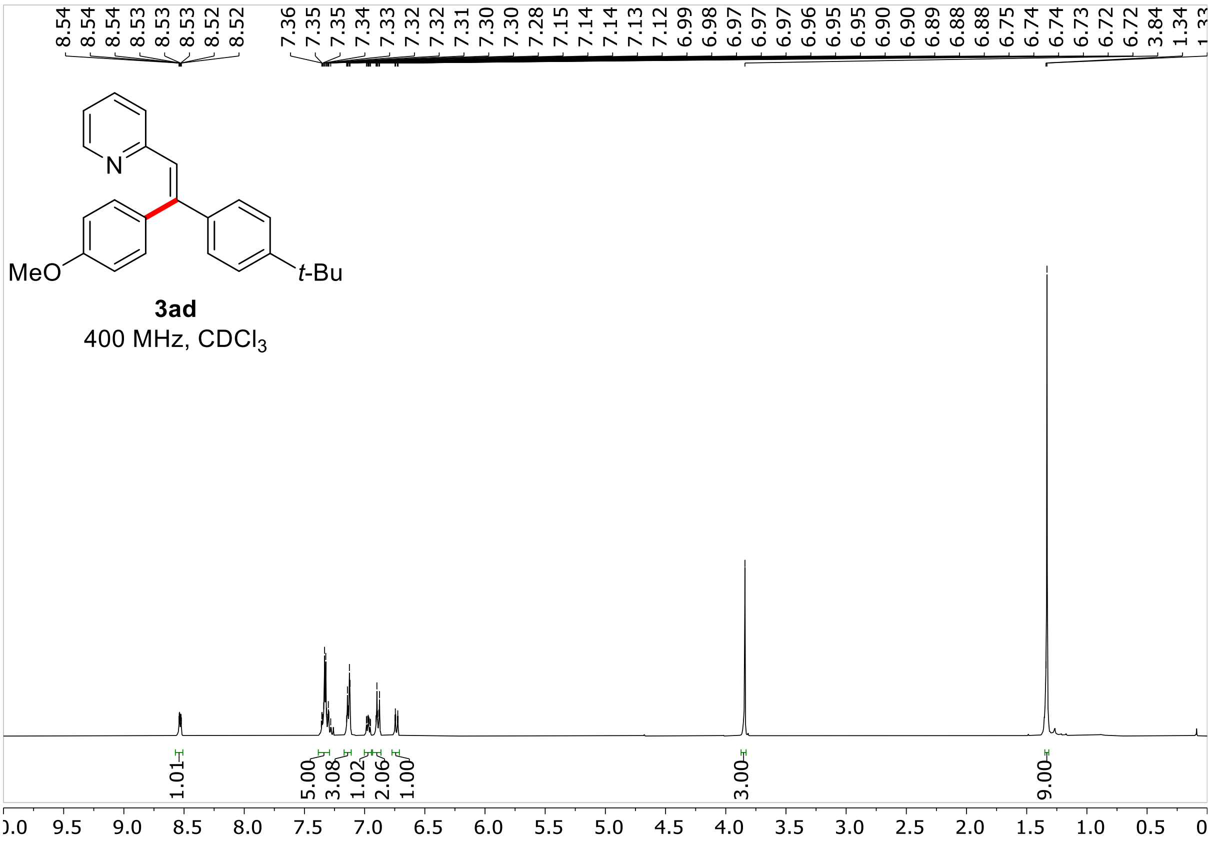
8.54 8.54 8.54 8.53 8.53 8.53 8.52 8.52
7.36 7.35 7.35 7.34 7.33 7.32 7.32 7.31 7.30 7.30 7.28 7.15 7.14 7.14 7.13 7.12 6.99 6.98 6.97 6.97 6.97 6.96 6.95 6.95 6.90 6.90 6.89 6.88 6.88 6.75 6.74 6.74 6.73 6.72 6.72 3.84 1.34
N
MeO
t-Bu
3ad
400 MHz, CDCl3
1.01
5.00 3.08 1.02 2.06 1.00
3.00
9.00
9.5 9.0 8.5 8.0 7.5 7.0 6.5 6.0 5.5 5.0 4.5 4.0 3.5 3.0 2.5 2.0 1.5 1.0 0.5 0

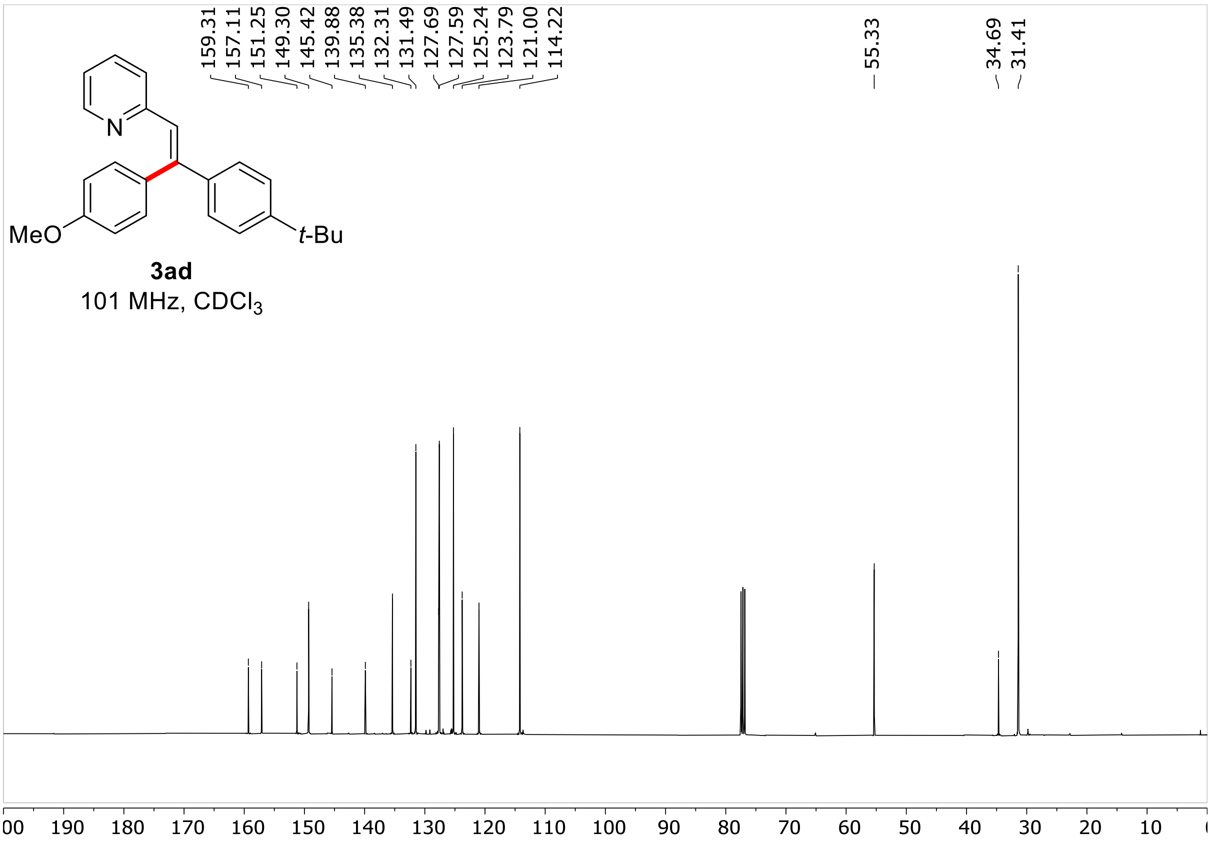
159.31 157.11 151.25 149.30 145.42 139.88 135.38 132.31 131.49 127.69 127.59 125.24 123.79 121.00 114.22
55.33
34.69 31.41
N
MeO
t-Bu
3ad
101 MHz, CDCl3
190 180 170 160 150 140 130 120 110 100 90 80 70 60 50 40 30 20 10

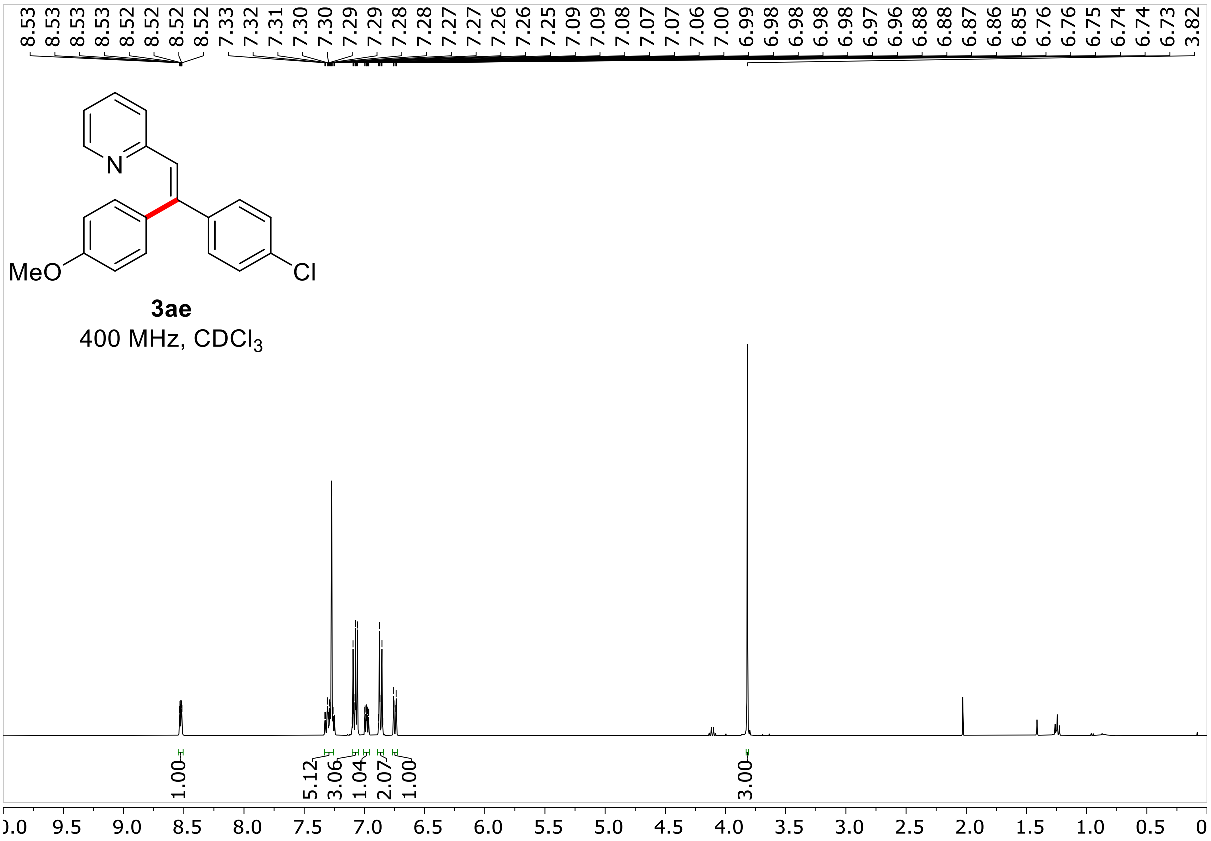
8.53 8.53 8.53 8.53 8.52 8.52 8.52 8.52 7.33 7.32 7.31 7.30 7.30 7.29 7.29 7.28 7.28 7.27 7.27 7.26 7.26 7.25 7.09 7.09 7.08 7.07 7.07 7.06 7.00 6.99 6.98 6.98 6.98 6.98 6.97 6.96 6.88 6.88 6.87 6.86 6.85 6.76 6.76 6.75 6.74 6.74 6.73 3.82
N
MeO
Cl
3ae
400 MHz, CDCl3
1.00
5.12
3.06
1.04
2.07
1.00
3.00

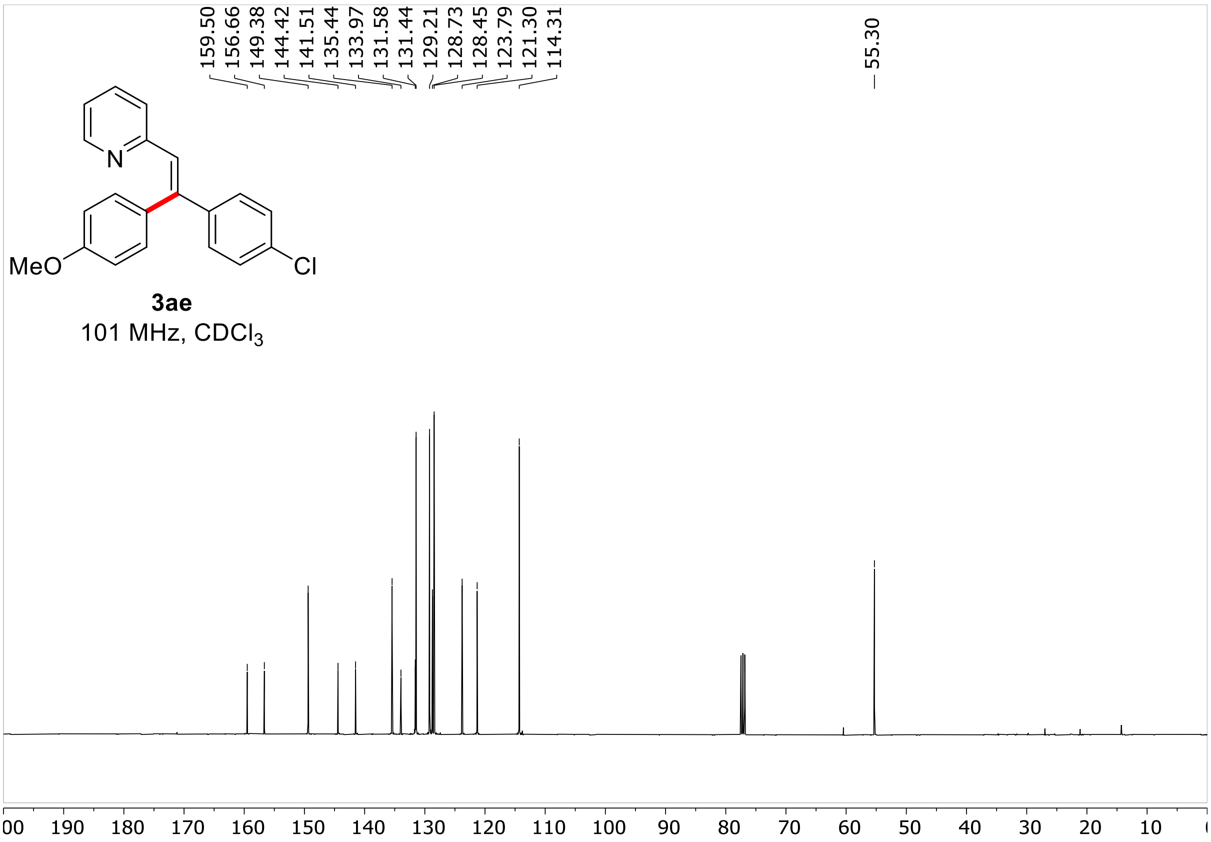
159.50 156.66 149.38 144.42 141.51 135.44 133.97 131.58 131.44 129.21 128.73 128.45 123.79 121.30 114.31
55.30
N
MeO
Cl
3ae
101 MHz, CDCl3

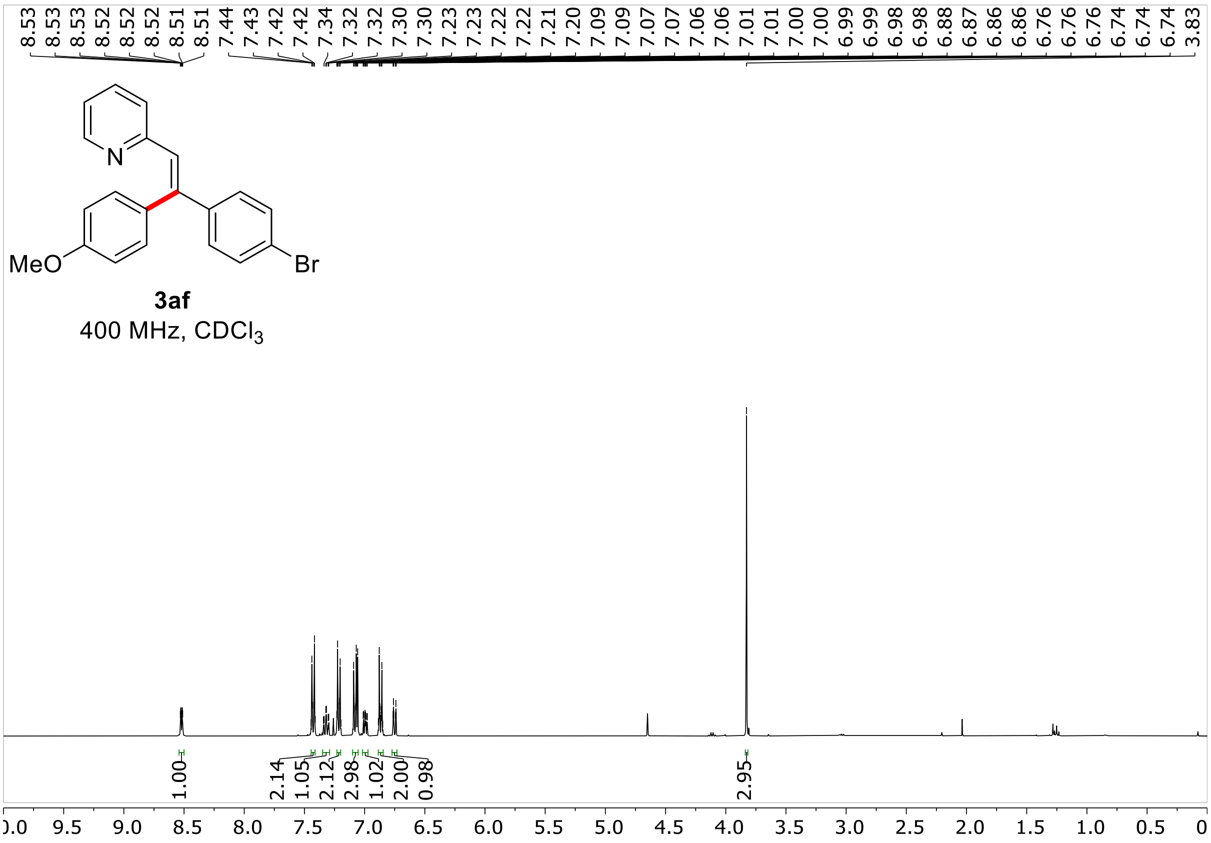

8.53
8.53
8.53
8.52
8.52
8.52
8.51
8.51
7.44
7.43
7.42
7.42
7.34
7.32
7.32
7.30
7.30
7.23
7.23
7.22
7.22
7.21
7.20
7.09
7.09
7.07
7.07
7.06
7.06
7.01
7.01
7.00
7.00
6.99
6.99
6.98
6.98
6.88
6.87
6.86
6.86
6.76
6.76
6.74
6.74
6.74
3.83
MeO
Br
N
3af
400 MHz, CDCl3
1.00
2.14
1.05
2.12
2.98
1.02
2.00
0.98
2.95
9.5
9.0
8.5
8.0
7.5
7.0
6.5
6.0
5.5
5.0
4.5
4.0
3.5
3.0
2.5
2.0
1.5
1.0
0.5
0

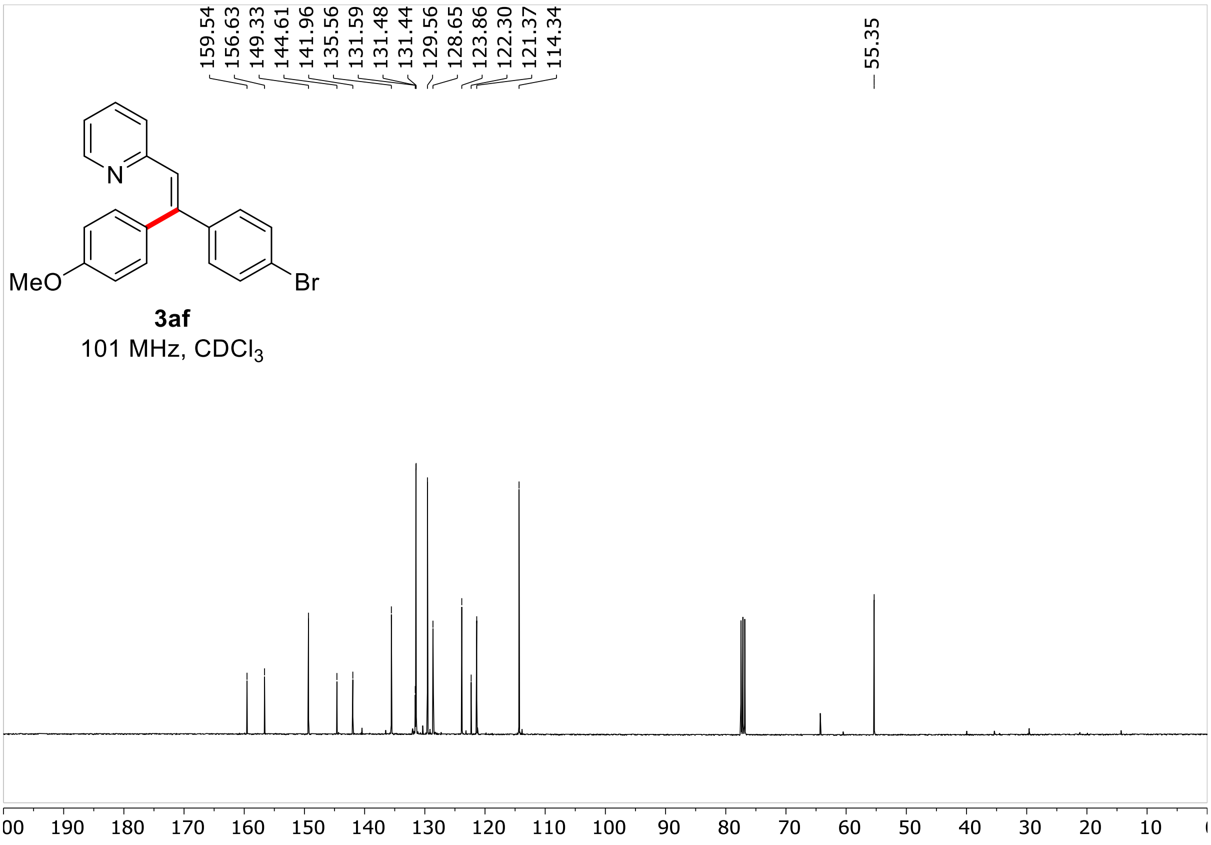

159.54
156.63
149.33
144.61
141.96
135.56
131.59
131.48
131.44
129.56
128.65
123.86
122.30
121.37
114.34
55.35
MeO
Br
N
3af
101 MHz, CDCl3
190
180
170
160
150
140
130
120
110
100
90
80
70
60
50
40
30
20
10

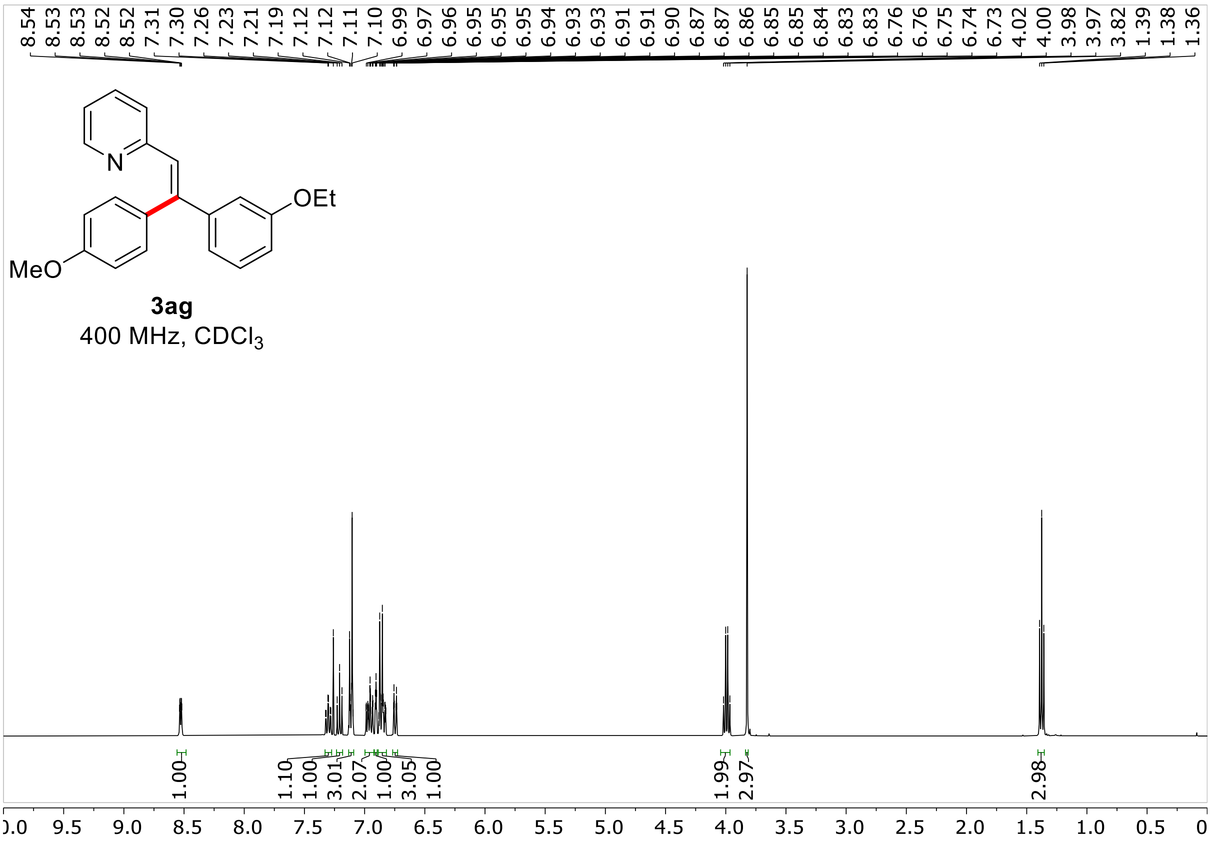
8.54
8.53
8.53
8.52
8.52
7.31
7.30
7.26
7.23
7.21
7.19
7.12
7.12
7.11
7.10
6.99
6.97
6.96
6.95
6.95
6.94
6.93
6.93
6.91
6.91
6.90
6.87
6.87
6.86
6.85
6.85
6.84
6.83
6.83
6.76
6.76
6.75
6.74
6.73
4.02
4.00
3.98
3.97
3.82
1.39
1.38
1.36
N
OEt
MeO
3ag
400 MHz, CDCl3
1.00
1.10
1.00
3.01
2.07
1.00
3.05
1.00
1.99
2.97
2.98
9.5
9.0
8.5
8.0
7.5
7.0
6.5
6.0
5.5
5.0
4.5
4.0
3.5
3.0
2.5
2.0
1.5
1.0
0.5
0

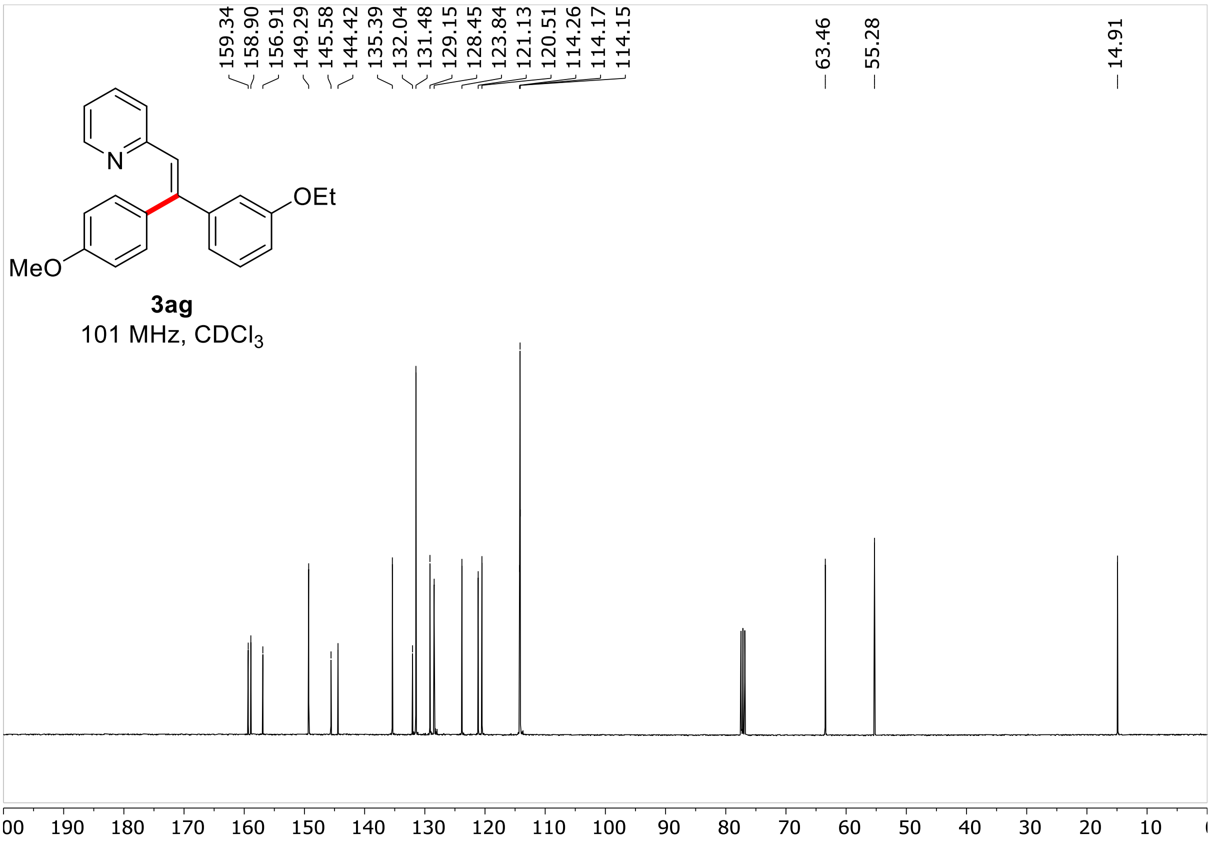
159.34
158.90
156.91
149.29
145.58
144.42
135.39
132.04
131.48
129.15
128.45
123.84
121.13
120.51
114.26
114.17
114.15
63.46
55.28
14.91
N
OEt
MeO
3ag
101 MHz, CDCl3
190
180
170
160
150
140
130
120
110
100
90
80
70
60
50
40
30
20
10

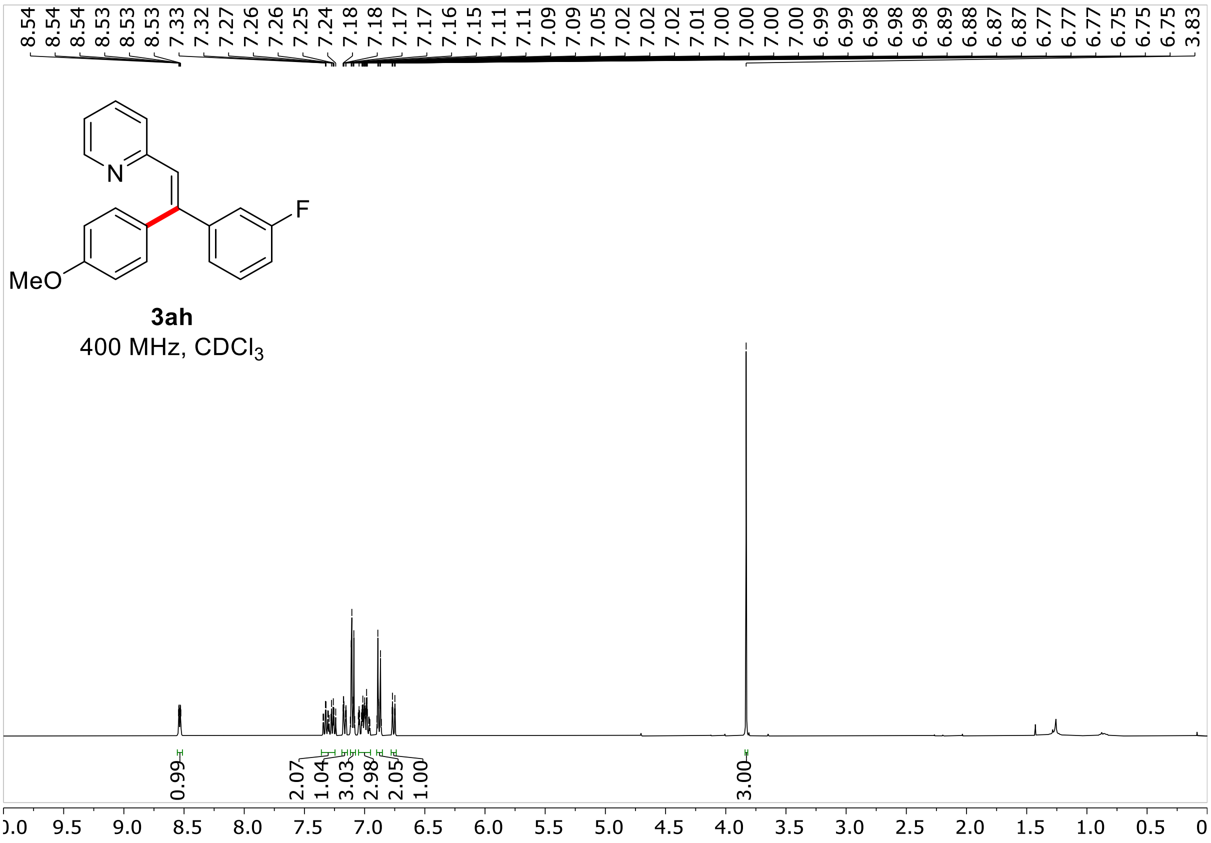
8.54 8.54 8.54 8.53 8.53 8.53 7.33 7.32 7.27 7.26 7.26 7.25 7.24 7.18 7.18 7.17 7.17 7.16 7.15 7.11 7.11 7.09 7.09 7.05 7.02 7.02 7.02 7.01 7.00 7.00 7.00 6.99 6.99 6.98 6.98 6.98 6.89 6.88 6.87 6.87 6.77 6.77 6.75 6.75 6.75 3.83
N
F
MeO
3ah
400 MHz, CDCl3
0.99
2.07 1.04 3.03 2.98 2.05 1.00
3.00
9.5 9.0 8.5 8.0 7.5 7.0 6.5 6.0 5.5 5.0 4.5 4.0 3.5 3.0 2.5 2.0 1.5 1.0 0.5 0

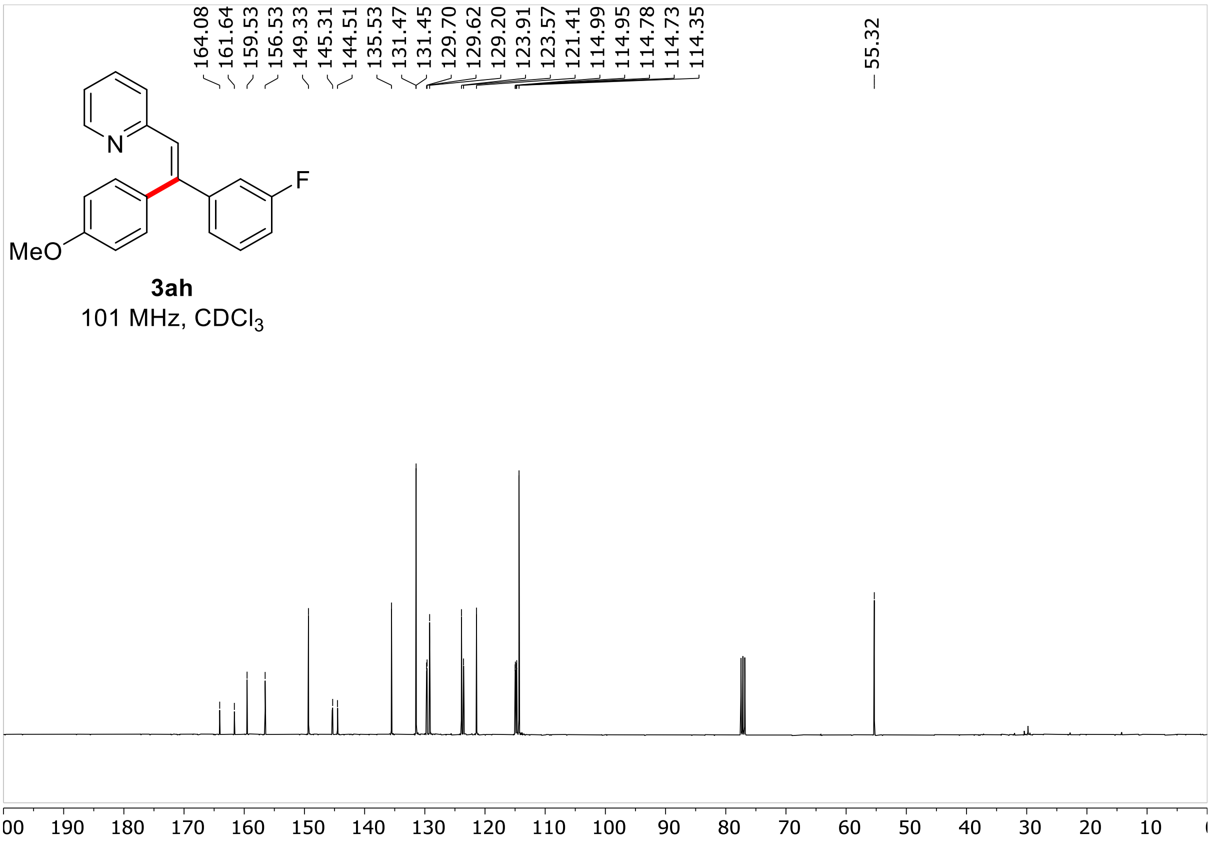
164.08 161.64 159.53 156.53 149.33 145.31 144.51 135.53 131.47 131.45 129.70 129.62 129.20 123.91 123.57 121.41 114.99 114.95 114.78 114.73 114.35
55.32
N
F
MeO
3ah
101 MHz, CDCl3
190 180 170 160 150 140 130 120 110 100 90 80 70 60 50 40 30 20 10

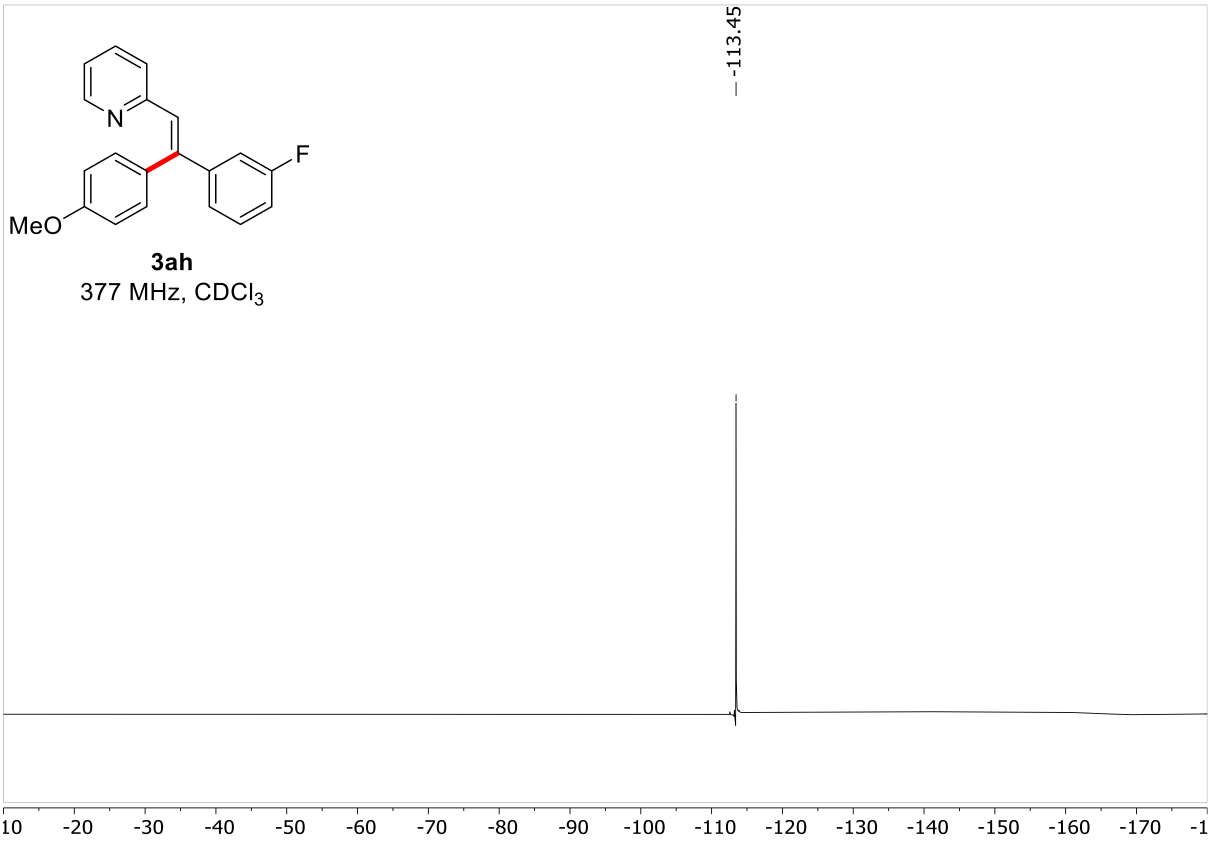

MeO
N
F
3ah
377 MHz, CDCl3
-113.45
10
-20
-30
-40
-50
-60
-70
-80
-90
-100
-110
-120
-130
-140
-150
-160
-170
-1

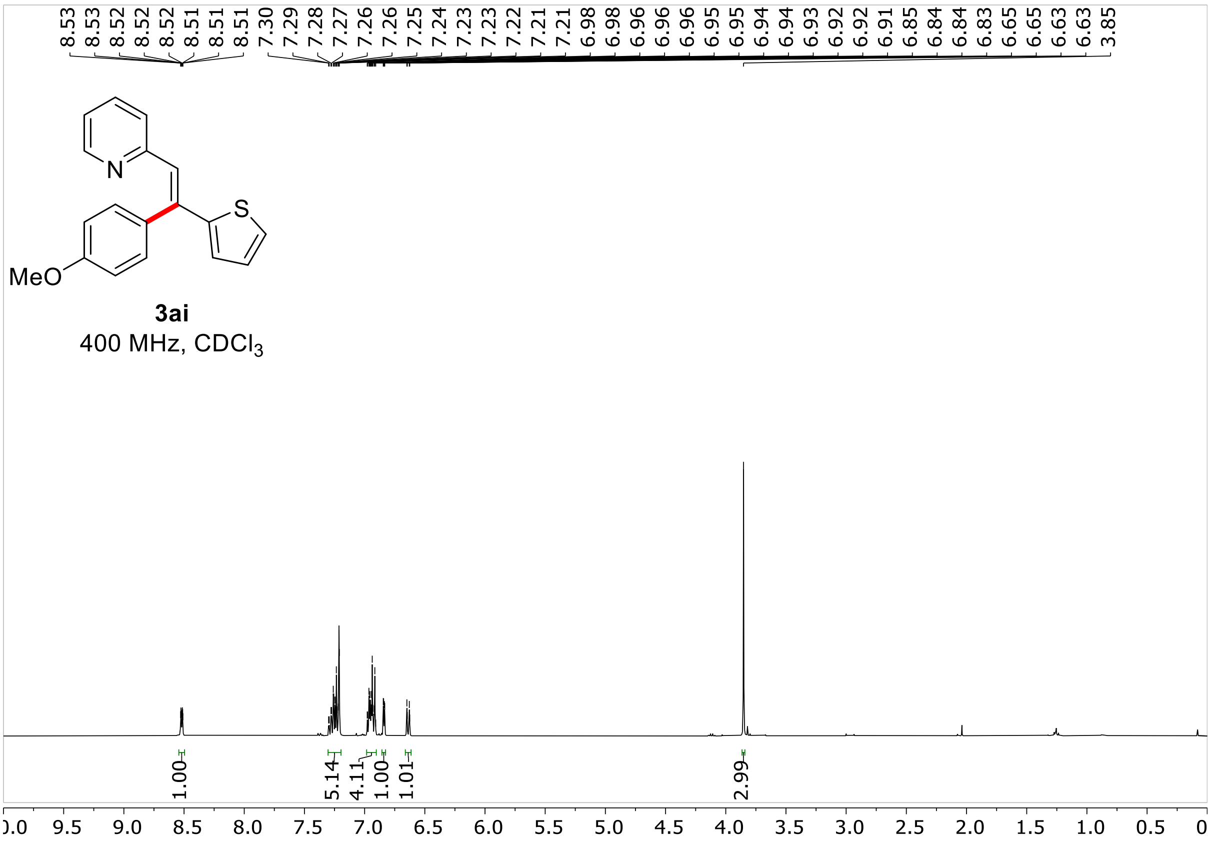
MeO
N
S
3ai
400 MHz, CDCl3

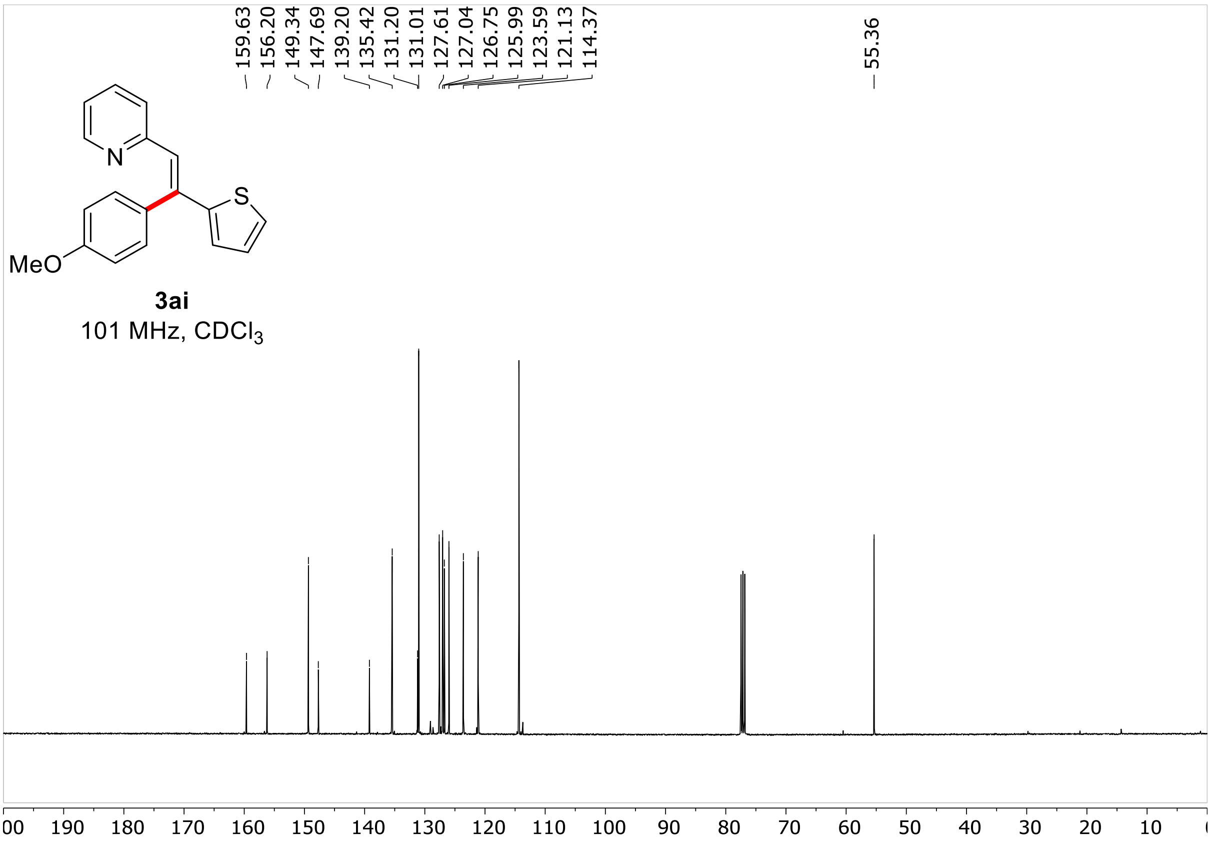
MeO
N
S
3ai
101 MHz, CDCl3

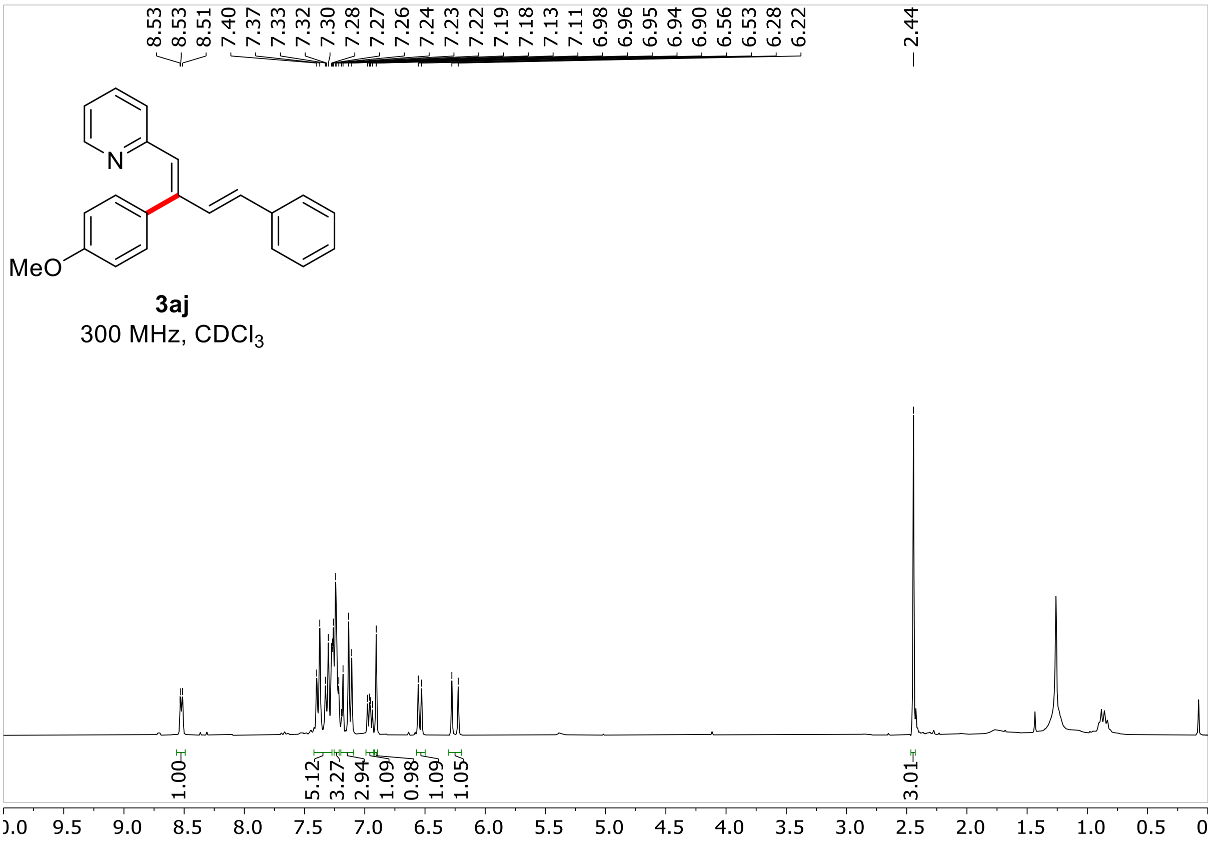
8.53
8.53
8.51
7.40
7.37
7.33
7.32
7.30
7.28
7.27
7.26
7.24
7.23
7.22
7.19
7.18
7.13
7.11
6.98
6.96
6.95
6.94
6.90
6.56
6.53
6.28
6.22
2.44
N
MeO
3aj
300 MHz, CDCl3
1.00
5.12
3.27
2.94
1.09
0.98
1.09
1.05
3.01
9.5 9.0 8.5 8.0 7.5 7.0 6.5 6.0 5.5 5.0 4.5 4.0 3.5 3.0 2.5 2.0 1.5 1.0 0.5 0

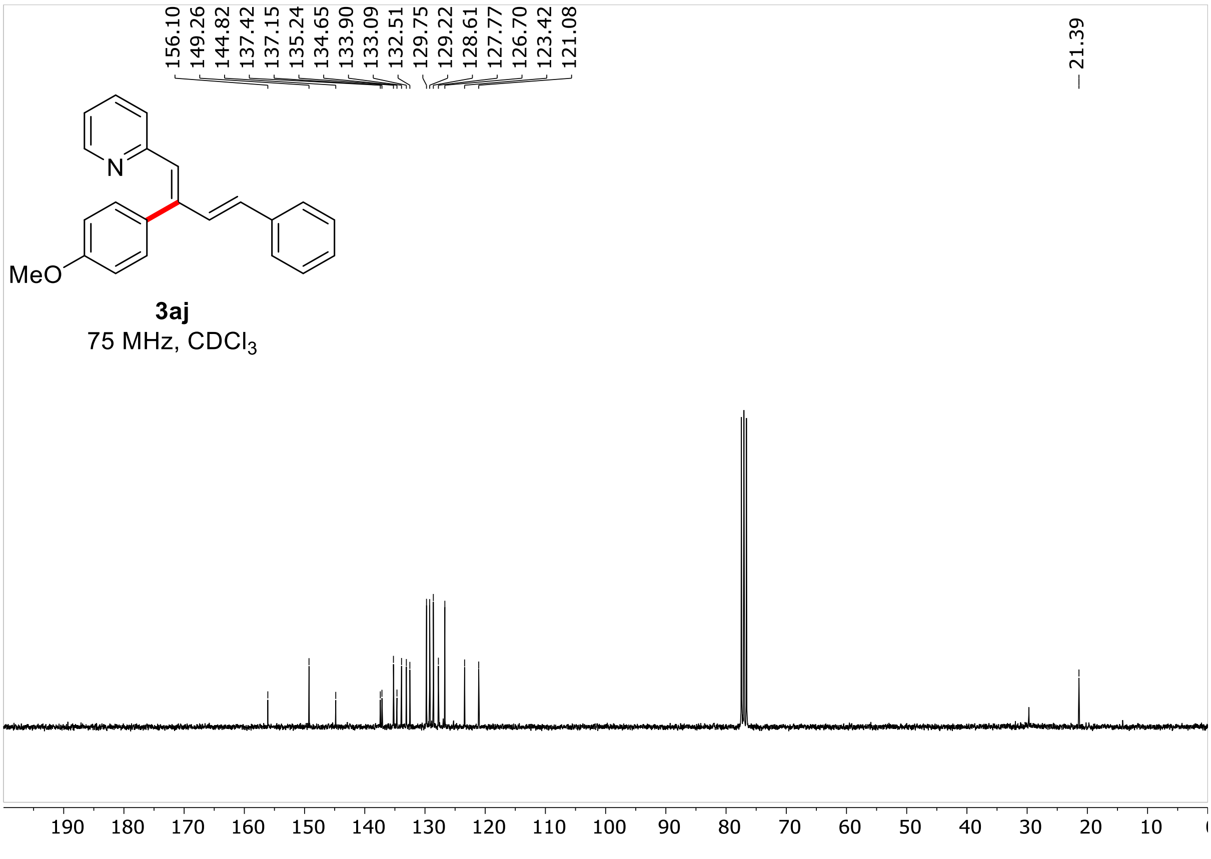
156.10
149.26
144.82
137.42
137.15
135.24
134.65
133.90
133.09
132.51
129.75
129.22
128.61
127.77
126.70
123.42
121.08
21.39
N
MeO
3aj
75 MHz, CDCl3
190 180 170 160 150 140 130 120 110 100 90 80 70 60 50 40 30 20 10

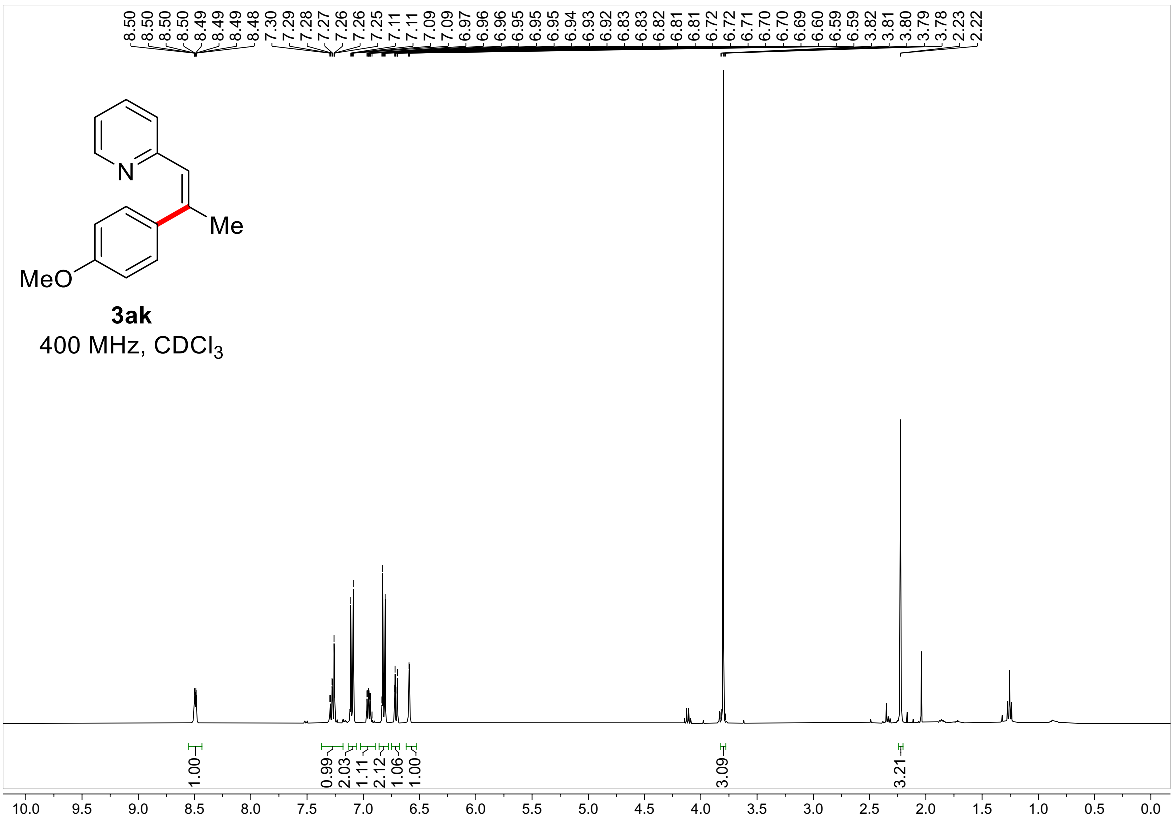

8.50 8.50 8.50 8.50 8.49 8.49 8.49 8.48 7.30 7.29 7.28 7.27 7.26 7.26 7.25 7.11 7.11 7.09 7.09 6.97 6.96 6.96 6.95 6.95 6.94 6.93 6.92 6.83 6.83 6.82 6.81 6.81 6.72 6.72 6.71 6.70 6.70 6.69 6.60 6.59 6.59 3.82 3.81 3.80 3.79 3.78 2.23 2.22
N
Me
MeO
3ak
400 MHz, CDCl3
1.00
0.99
2.03
1.11
2.12
1.06
1.00
3.09
3.21
10.0 9.5 9.0 8.5 8.0 7.5 7.0 6.5 6.0 5.5 5.0 4.5 4.0 3.5 3.0 2.5 2.0 1.5 1.0 0.5 0.0

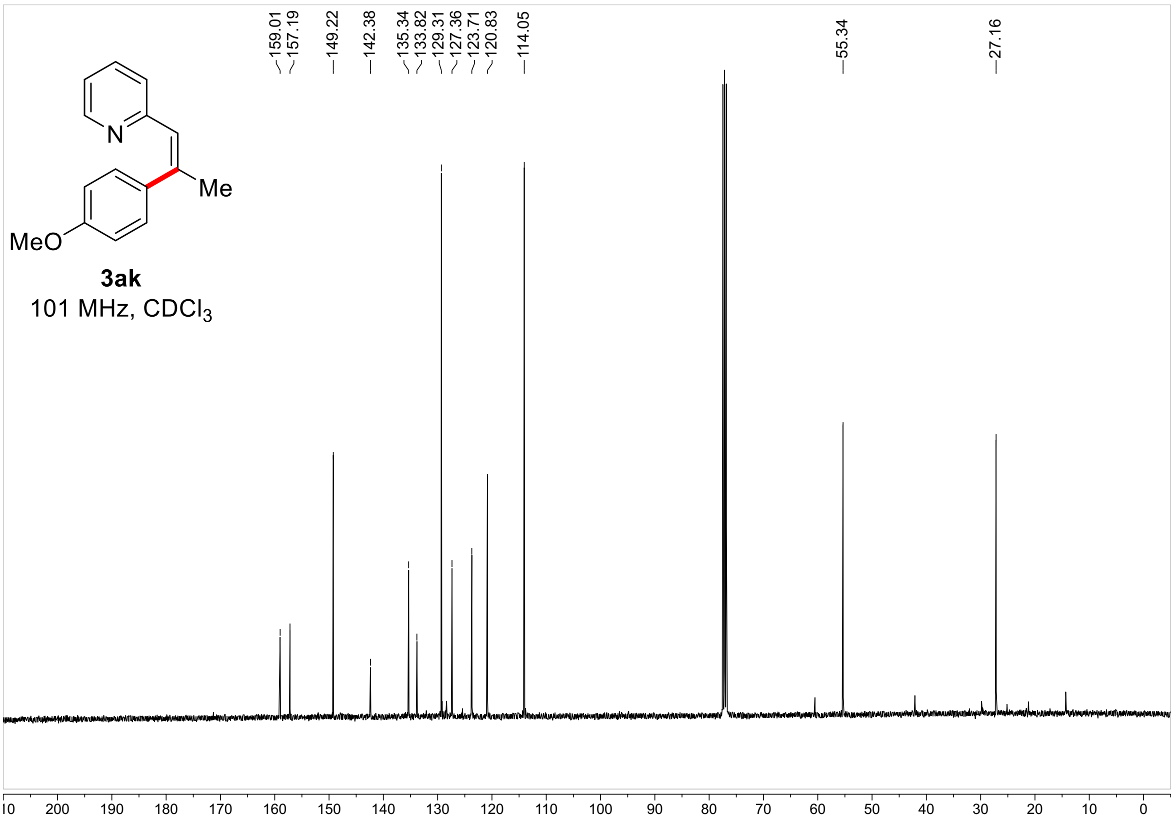

159.01 157.19 149.22 142.38 135.34 133.82 129.31 127.36 123.71 120.83 114.05 55.34 27.16
N
Me
MeO
3ak
101 MHz, CDCl3
200 190 180 170 160 150 140 130 120 110 100 90 80 70 60 50 40 30 20 10 0

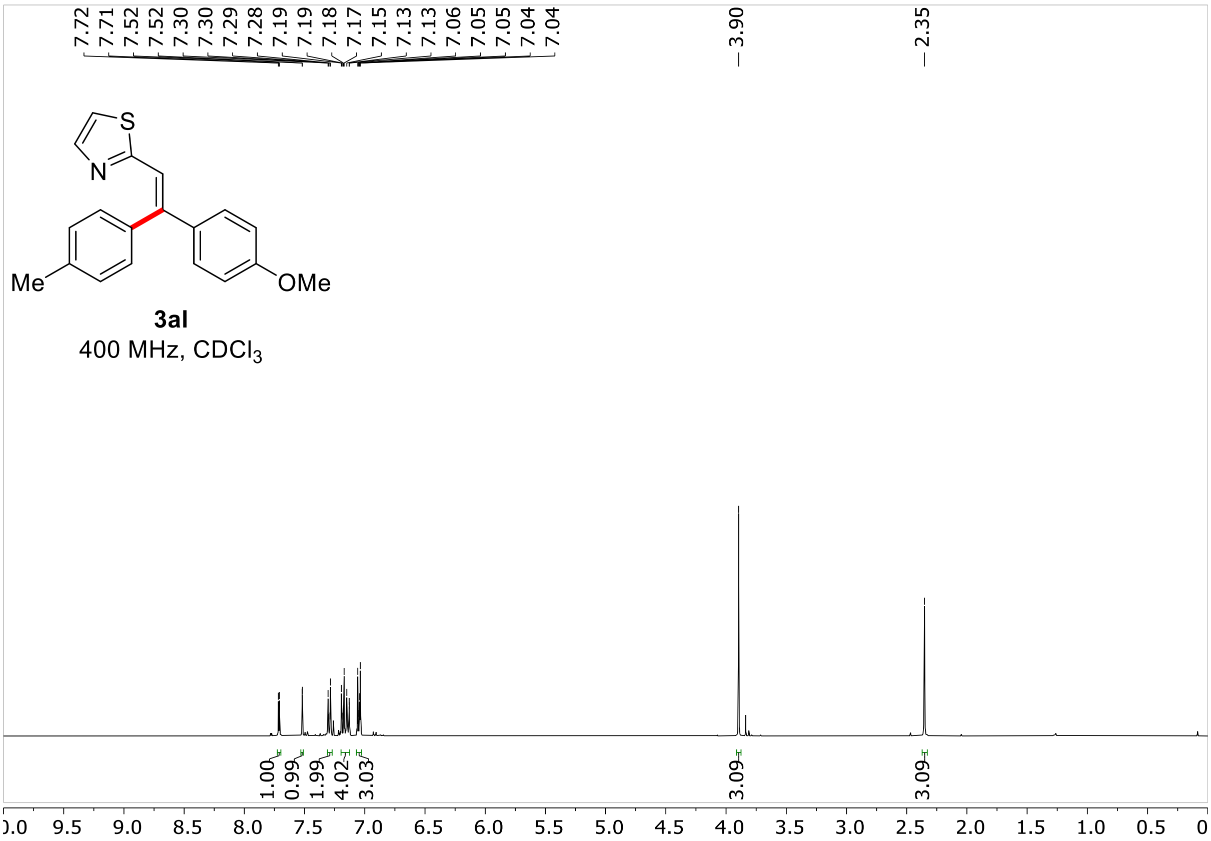
7.72
7.71
7.52
7.52
7.30
7.30
7.29
7.28
7.19
7.19
7.18
7.17
7.15
7.13
7.13
7.06
7.05
7.05
7.04
7.04
3.90
2.35
S
N
Me
OMe
3al
400 MHz, CDCl3
1.00
0.99
1.99
4.02
3.03
3.09
3.09
9.5
9.0
8.5
8.0
7.5
7.0
6.5
6.0
5.5
5.0
4.5
4.0
3.5
3.0
2.5
2.0
1.5
1.0
0.5
0

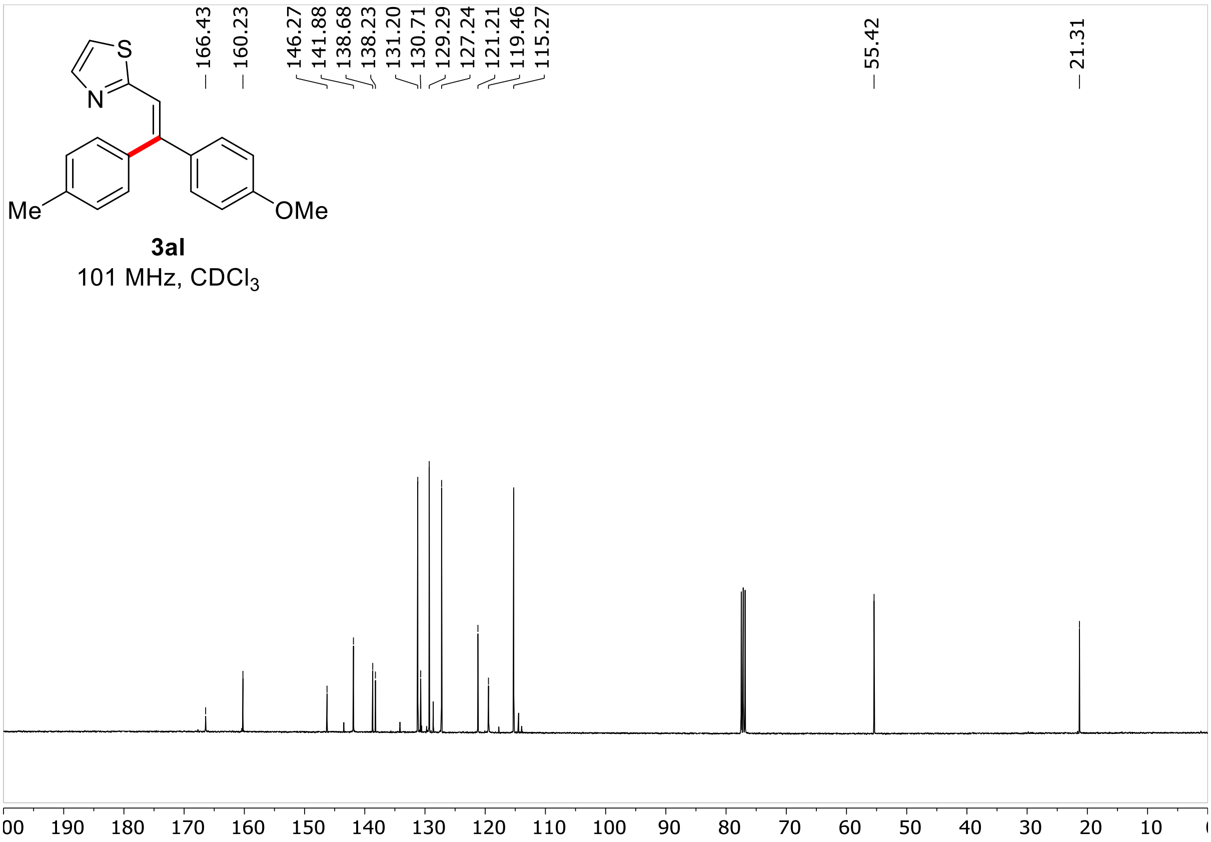
166.43
160.23
146.27
141.88
138.68
138.23
131.20
130.71
129.29
127.24
121.21
119.46
115.27
55.42
21.31
S
N
Me
OMe
3al
101 MHz, CDCl3
190
180
170
160
150
140
130
120
110
100
90
80
70
60
50
40
30
20
10

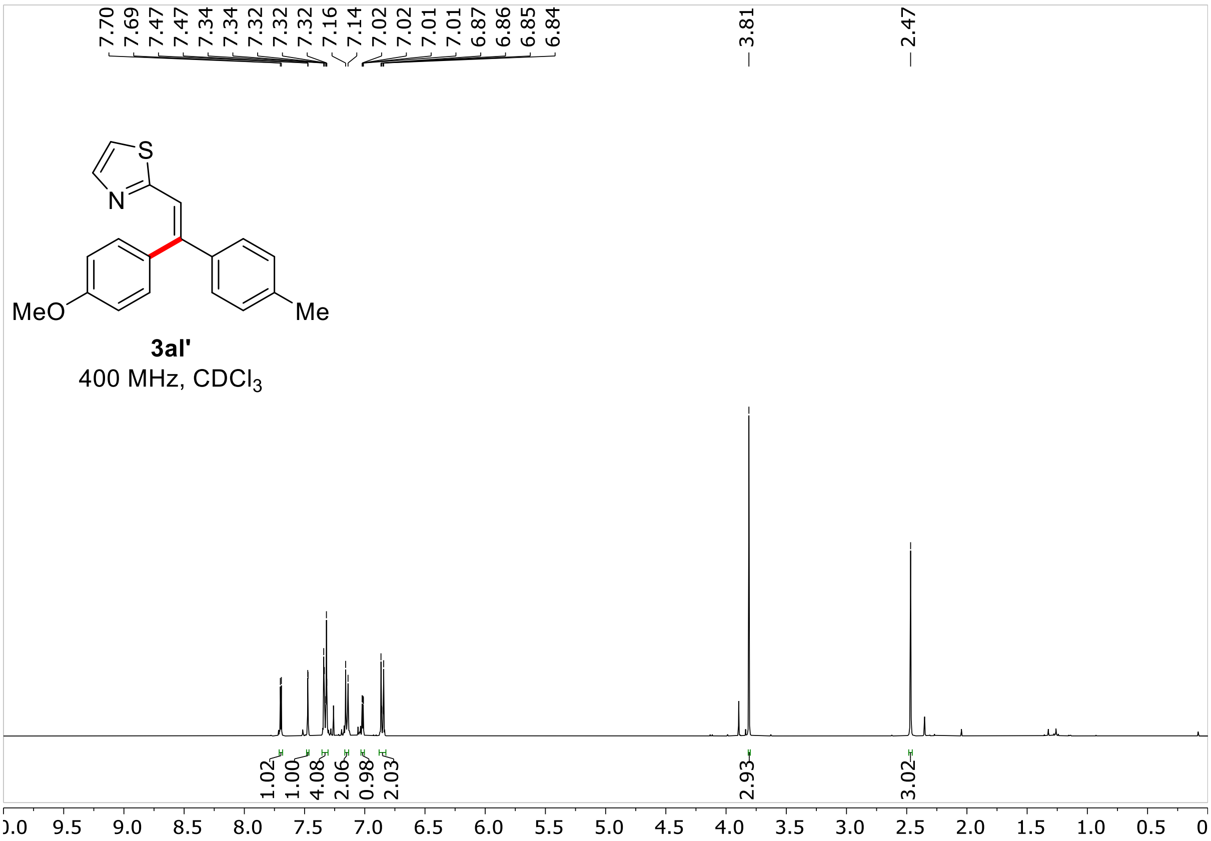
7.70
7.69
7.47
7.47
7.34
7.34
7.32
7.32
7.32
7.16
7.14
7.02
7.02
7.01
7.01
6.87
6.86
6.85
6.84
3.81
2.47
S
N
MeO
Me
3al'
400 MHz, CDCl3
1.02
1.00
4.08
2.06
0.98
2.03
2.93
3.02
9.5
9.0
8.5
8.0
7.5
7.0
6.5
6.0
5.5
5.0
4.5
4.0
3.5
3.0
2.5
2.0
1.5
1.0
0.5
0

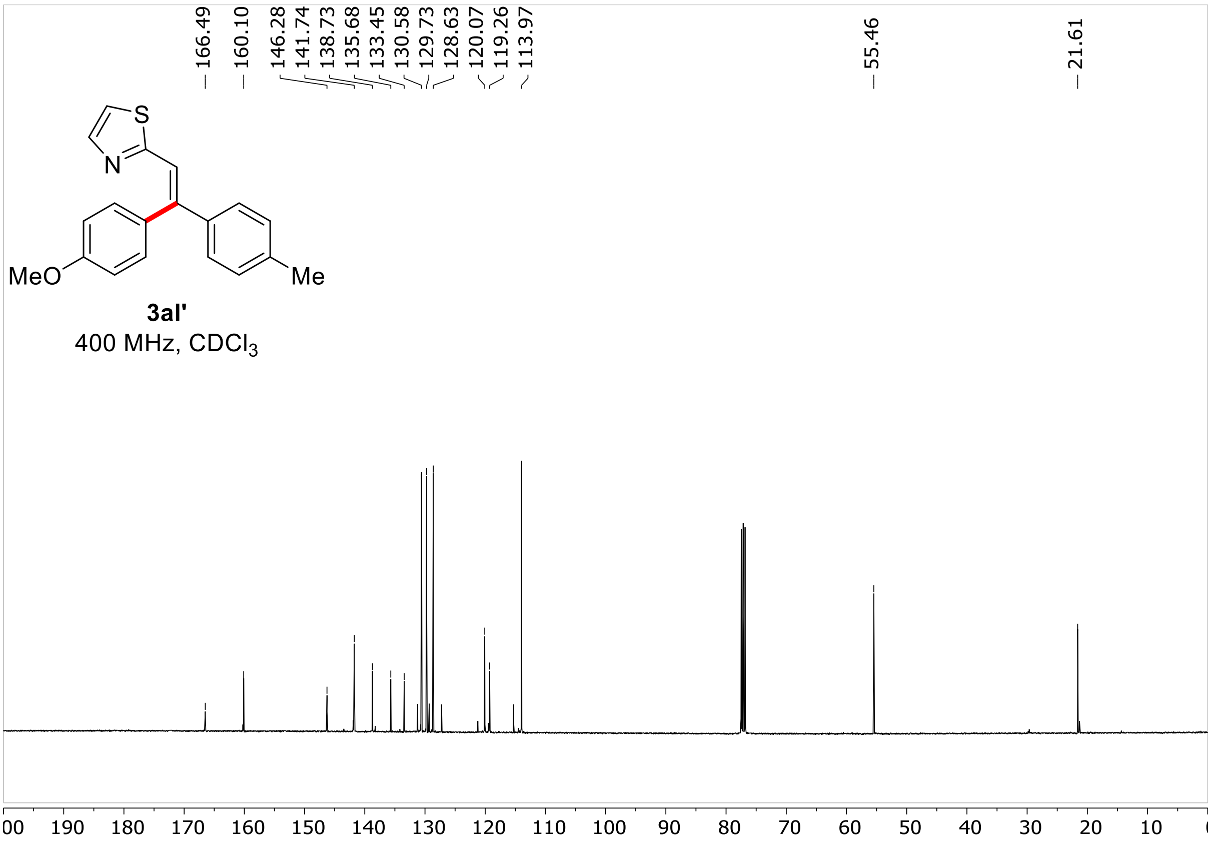
166.49
160.10
146.28
141.74
138.73
135.68
133.45
130.58
129.73
128.63
120.07
119.26
113.97
55.46
21.61
S
N
MeO
Me
3al'
400 MHz, CDCl3
190
180
170
160
150
140
130
120
110
100
90
80
70
60
50
40
30
20
10

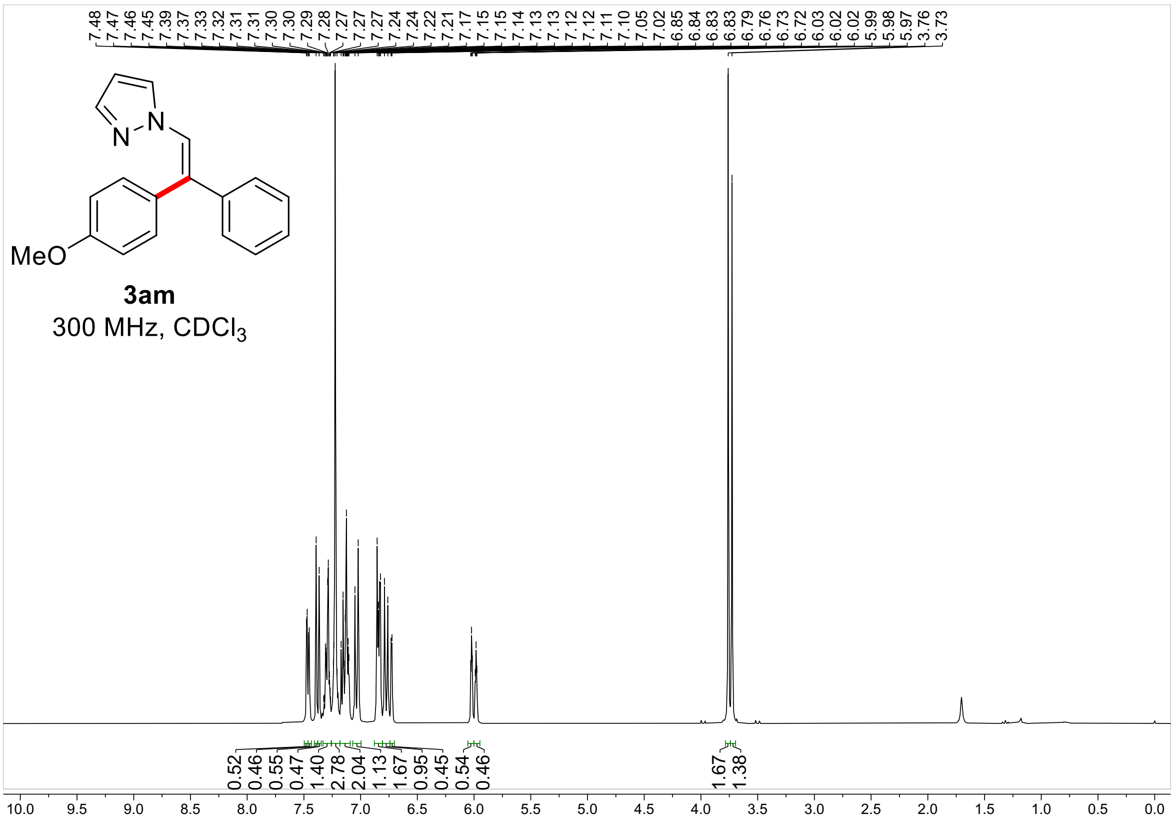

MeO
3am
300 MHz, CDCl3

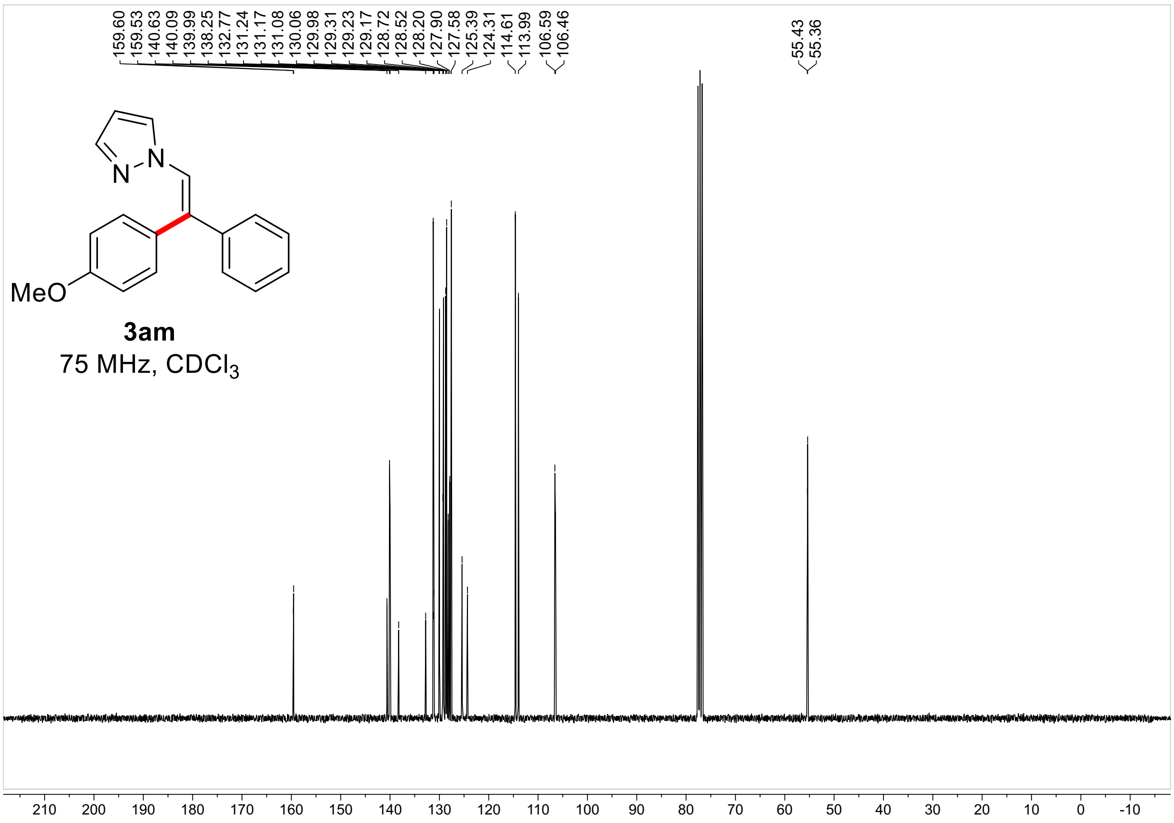

MeO
3am
75 MHz, CDCl3

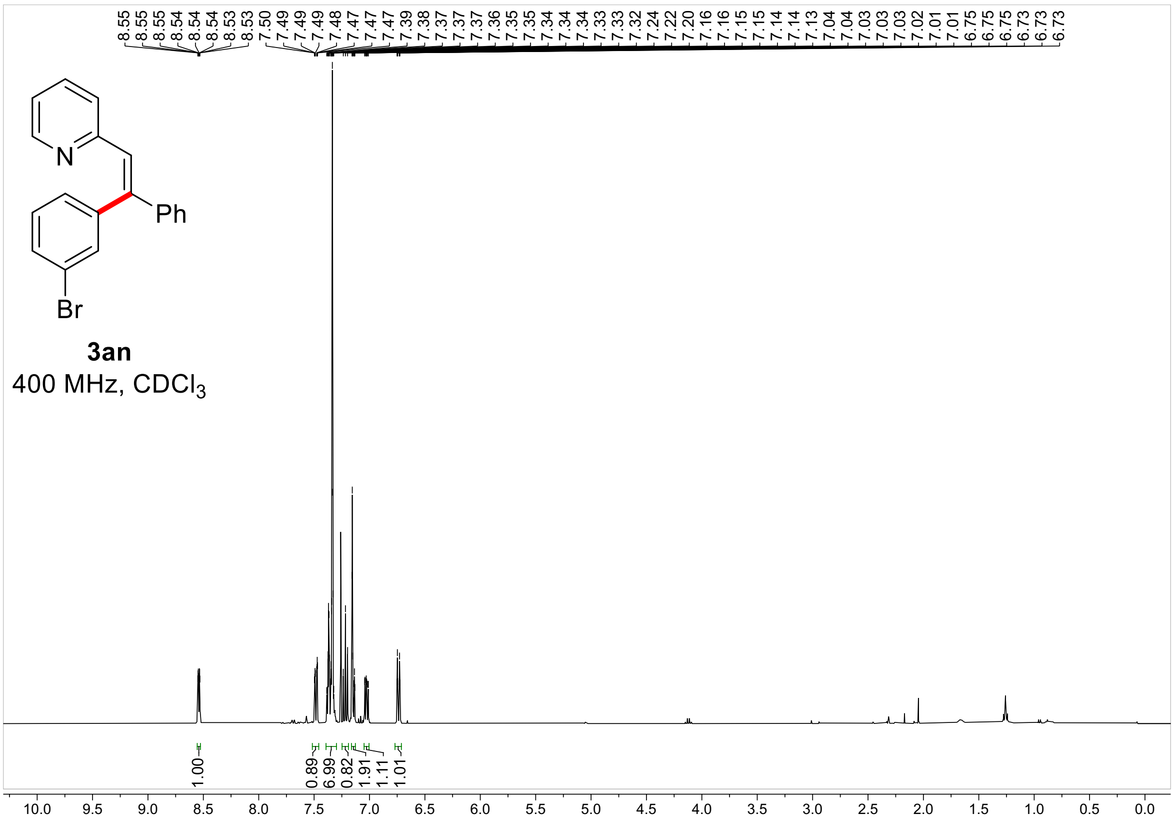
8.55 8.55 8.55 8.54 8.54 8.54 8.53 8.53 7.50 7.49 7.49 7.48 7.47 7.47 7.47 7.39 7.38 7.37 7.37 7.37 7.36 7.35 7.35 7.34 7.34 7.33 7.33 7.32 7.24 7.22 7.20 7.16 7.16 7.15 7.15 7.14 7.13 7.04 7.04 7.03 7.03 7.02 7.01 7.01 6.75 6.75 6.73 6.73 6.73
N
Ph
Br
3an
400 MHz, CDCl3
1.00
0.89 6.99 0.82 1.91 1.11 1.01
10.0 9.5 9.0 8.5 8.0 7.5 7.0 6.5 6.0 5.5 5.0 4.5 4.0 3.5 3.0 2.5 2.0 1.5 1.0 0.5 0.0

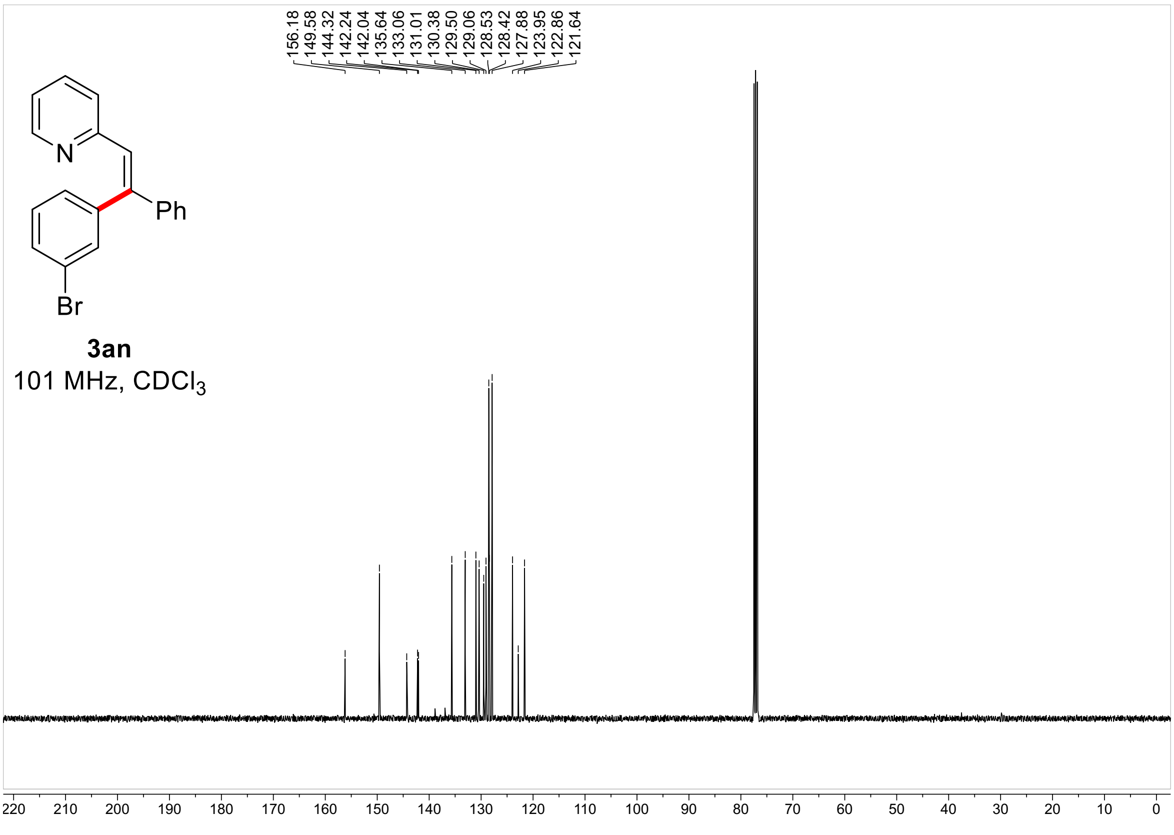
156.18 149.58 144.32 142.24 142.04 135.64 133.06 131.01 130.38 129.50 129.06 128.53 128.42 127.88 123.95 122.86 121.64
N
Ph
Br
3an
101 MHz, CDCl3
220 210 200 190 180 170 160 150 140 130 120 110 100 90 80 70 60 50 40 30 20 10 0

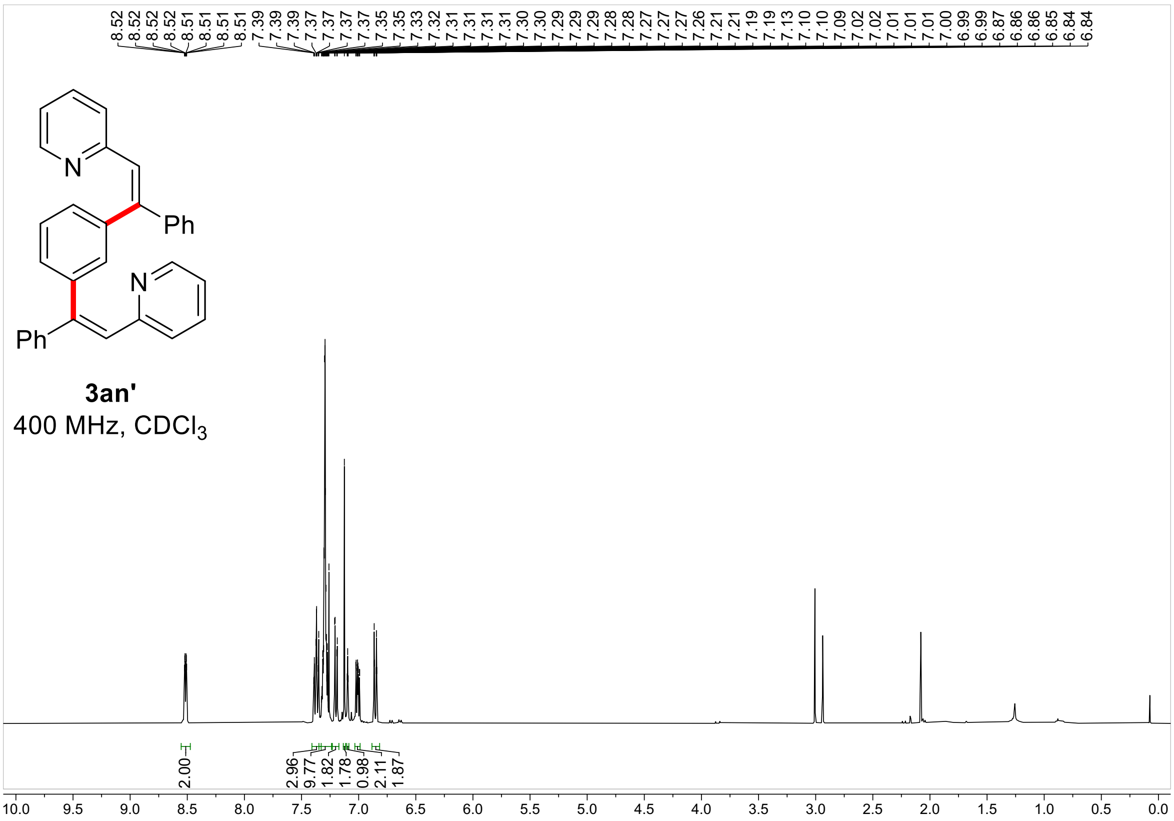
3an'
400 MHz, CDCl3

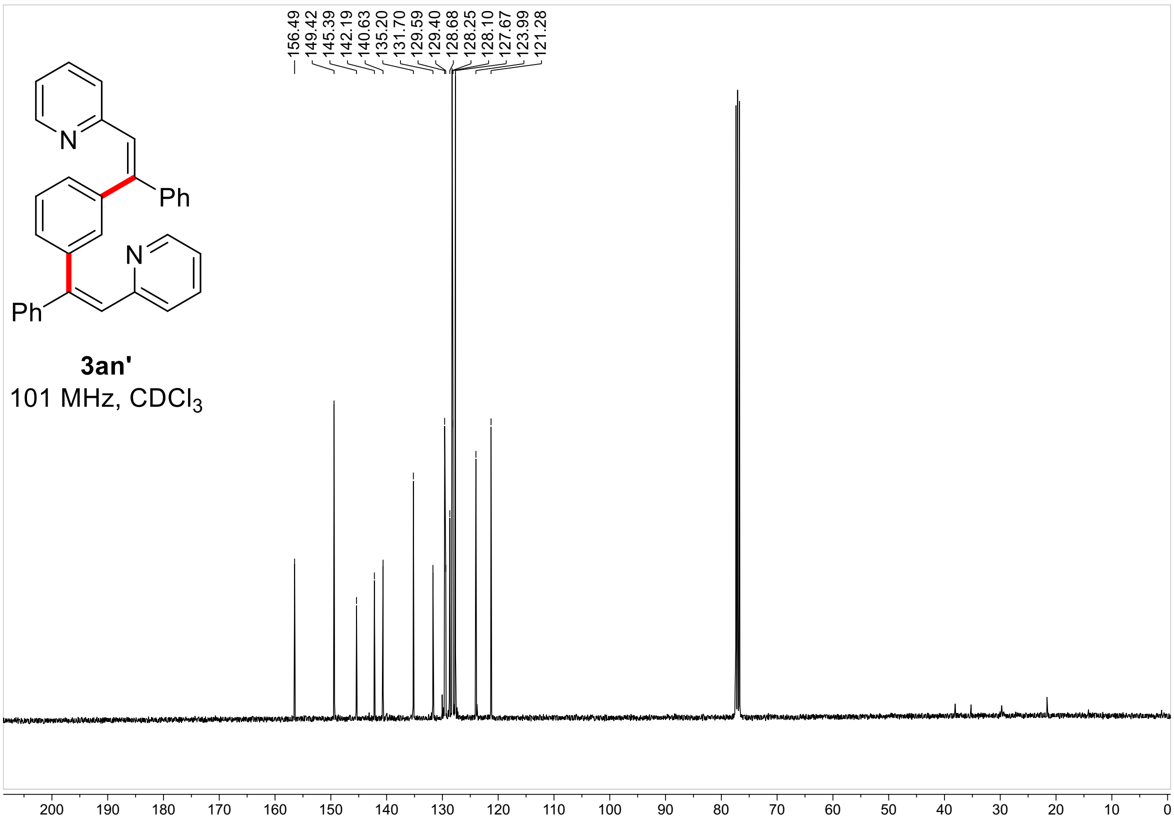
3an'
101 MHz, CDCl3
156.49
149.42
145.39
142.19
140.63
135.20
131.70
129.59
129.40
128.68
128.25
128.10
127.67
123.99
121.28

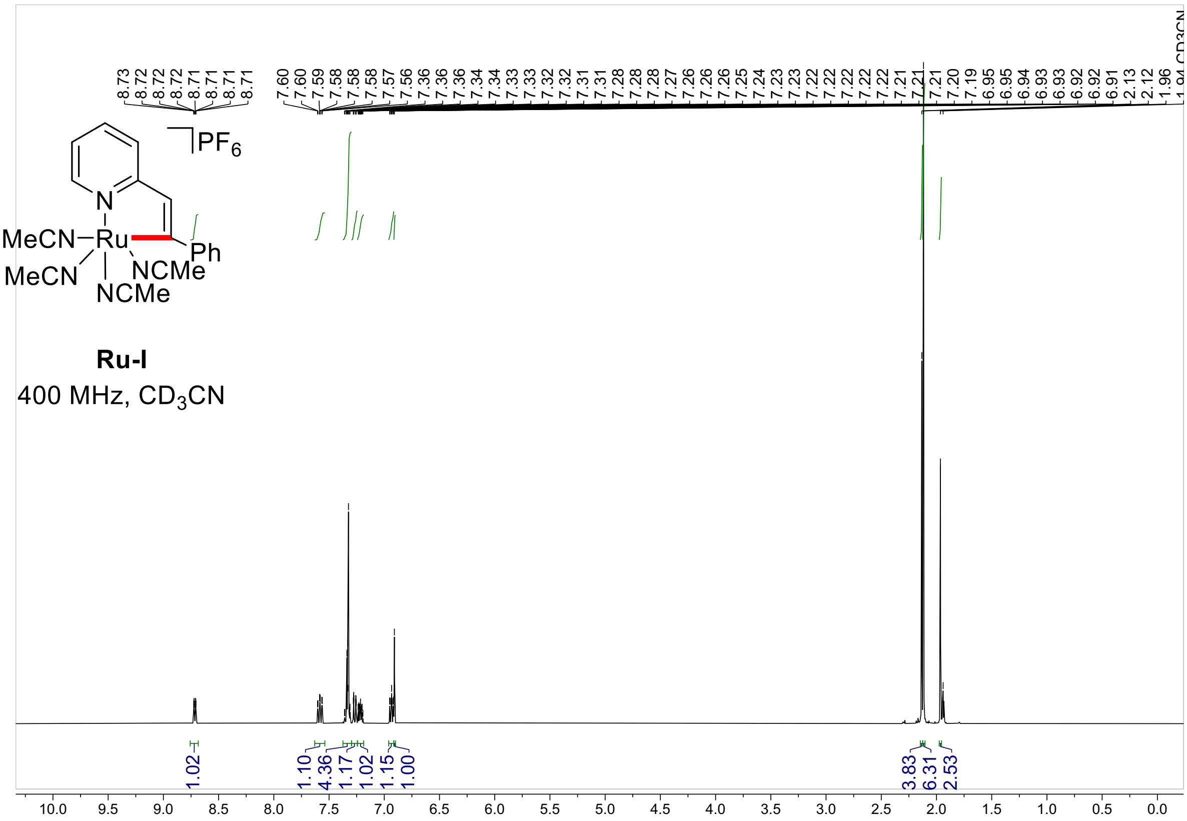

PF6
MeCN
Ru
N
Ph
NCMe
Ru-I
400 MHz, CD3CN

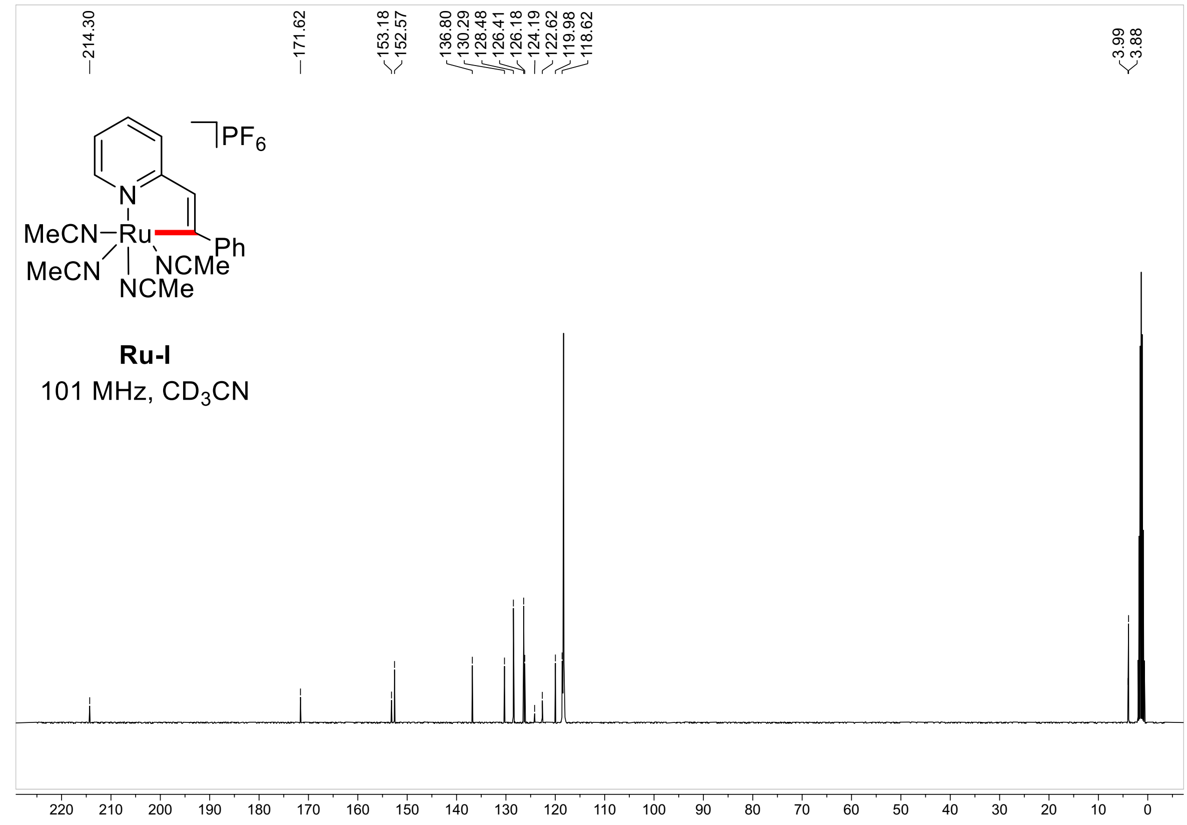

PF6
MeCN
Ru
N
Ph
NCMe
Ru-I
101 MHz, CD3CN

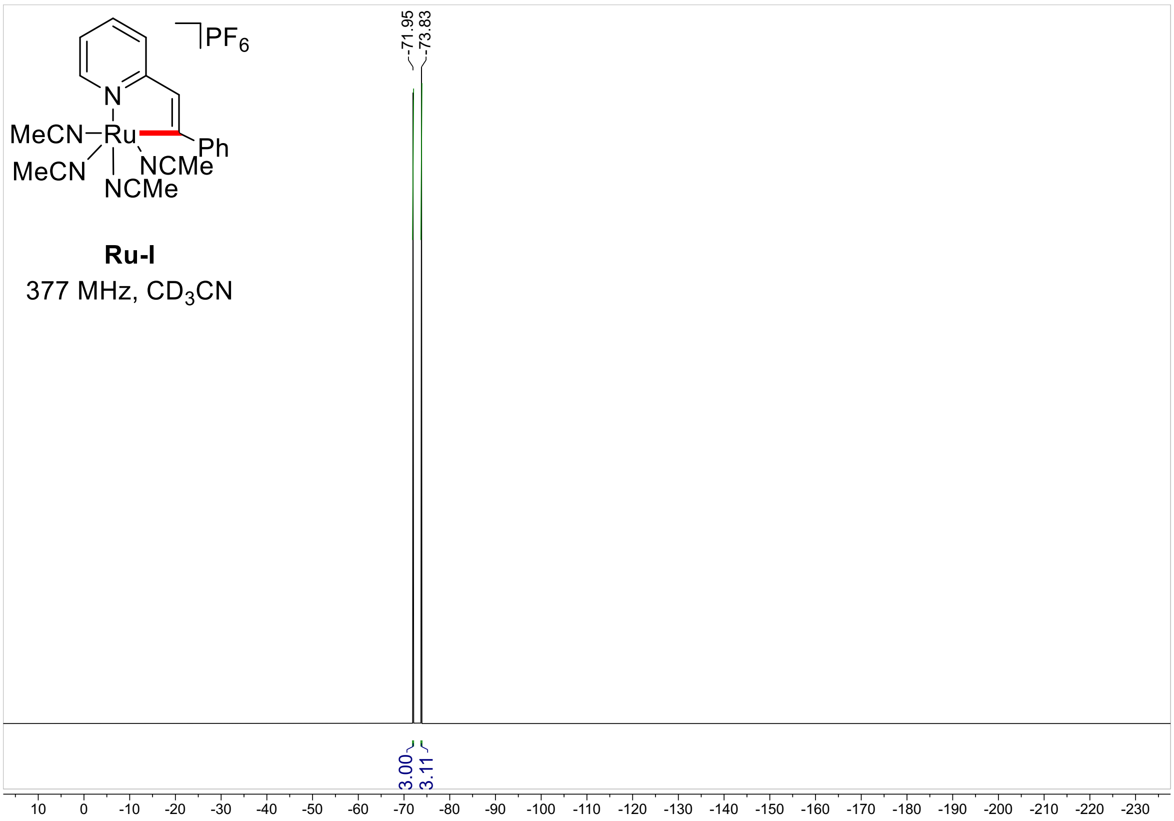
PF6
MeCN
Ru
NCMe
Ph
Ru-I
377 MHz, CD3CN
-71.95
-73.83
3.00
3.11
10 0 -10 -20 -30 -40 -50 -60 -70 -80 -90 -100 -110 -120 -130 -140 -150 -160 -170 -180 -190 -200 -210 -220 -230

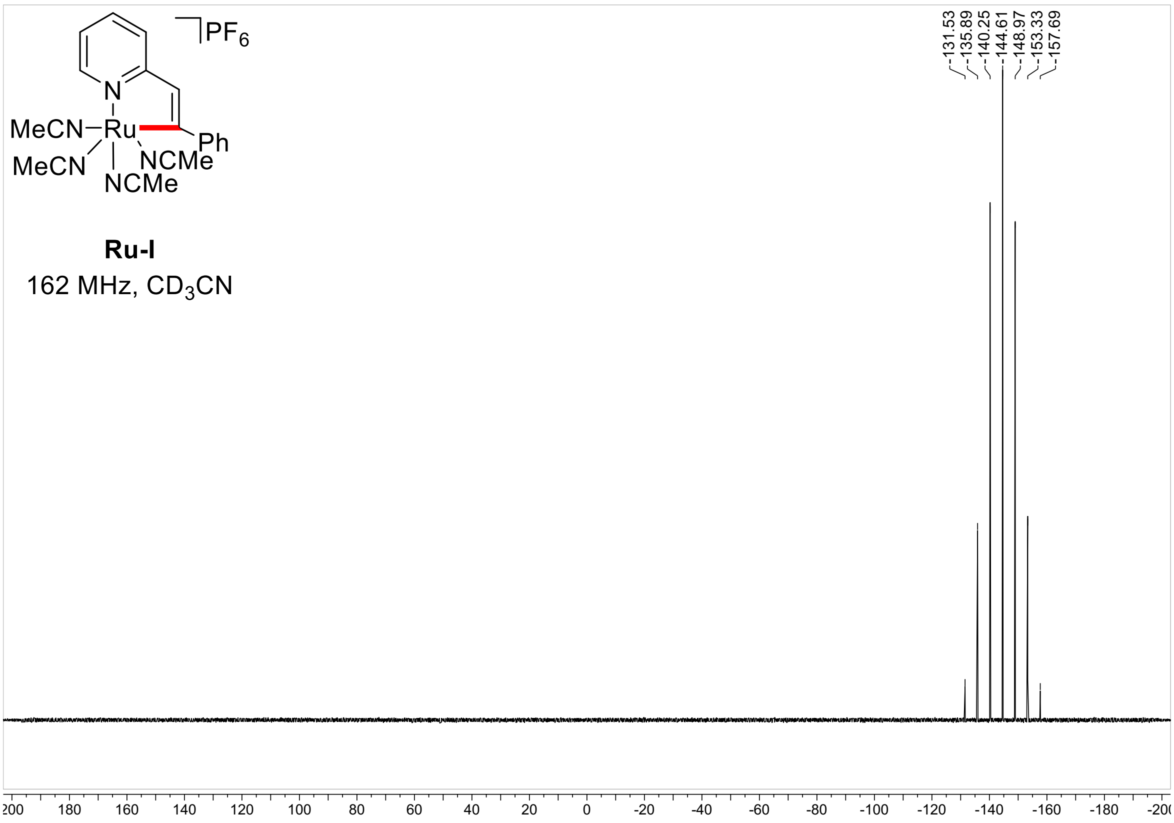
PF6
MeCN
Ru
NCMe
Ph
Ru-I
162 MHz, CD3CN
-131.53
-135.89
-140.25
-144.61
-148.97
-153.33
-157.69
200 180 160 140 120 100 80 60 40 20 0 -20 -40 -60 -80 -100 -120 -140 -160 -180 -200
